# BRF110, an Orally Active Nurr1-RXRα-Selective Rexinoid, Enhances BDNF Expression without Elevating Triglycerides

**DOI:** 10.1021/acs.jmedchem.4c03046

**Published:** 2025-02-13

**Authors:** Xenophon Asvos, Mohamed A. El Mubarak, Theodoros Karampelas, Theodoros Rampias, Constantin Tamvakopoulos, Gregory B. Sivolapenko, Athanasios Papakyriakou, Stavros Topouzis, Demetrios K. Vassilatis, Demosthenes Fokas

**Affiliations:** † Department of Materials Science and Engineering, University of Ioannina, Ioannina 45110, Greece; ‡ Department of Pharmacy, University of Patras, Patras 26504, Greece; § Center for Clinical Research, Experimental Surgery, and Translational Research, Biomedical Research Foundation of the Academy of Athens, Athens 11527, Greece; ∥ Institute of Biosciences and Applications, National Centre for Scientific Research “Demokritos”, Athens 15341, Greece

## Abstract

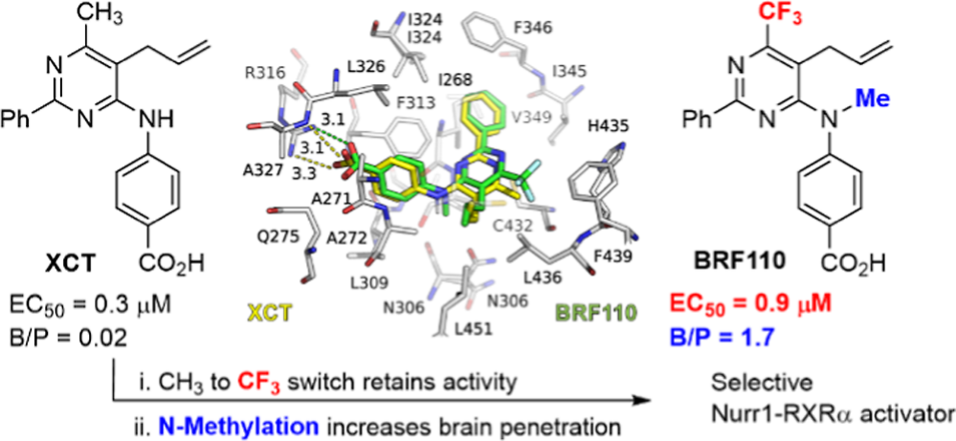

We report the discovery of a Nurr1-RXRα heterodimer-selective rexinoid which emerged from the structural modification of aminopyrimidine XCT0135908. Although XCT0135908 demonstrated high selectivity for the Nurr1-RXRα heterodimer over other RXRα dimerization partners, its poor in vivo stability and limited brain penetration hindered its utility. Structure–activity relationship (SAR) studies alongside bioactivity evaluations of a diverse series of substituted pyrimidines led to BRF110, a brain-penetrant compound retaining the selective activation of the Nurr1-RXRα heterodimer. BRF110, as XCT0135908, protects dopaminergic cells against the Parkinson’s disease-related toxin MPP^+^ and increases BDNF transcription in mice. Notably, BRF110, in contrast to the market-approved pan-RXR agonist bexarotene, did not elevate triglyceride levels, indicating that enhanced heterodimer selectivity can mitigate off-target in vivo side effects of rexinoids. These findings highlight the potential of heterodimer-selective scaffolds as a strategy for improving the therapeutic profile of rexinoids, addressing significant challenges in the clinical development of RXR-targeting molecules.

## Introduction

Retinol, retinal, and retinoic acid are essential vitamin A metabolites with diverse functions in development, differentiation, homeostasis, and cell fate regulation.^1^ The actions of retinoic acid are mediated by two nuclear receptor families, RARs and RXRs (α, β, and γ), for which it serves as a high-affinity ligand.^2^ The pleiotropic signaling pathways controlled by RXRs necessitate the formation of heterodimers with various nuclear receptors delineating their diverse cellular functions. RXR agonists, known as rexinoids, induce apoptosis in cancer cells,^3,4^ modulate lipid and glucose metabolism, and have progressed in clinical development for cancer and metabolic diseases,^5^ neuropsychiatric disorders,^6−8^ and neurodegenerative disorders.^9^ However, rexinoids are known to suppress the thyroid hormone axis^10^ and to cause rapid triglyceride increases.^11^ This is attributed to a single compound activating several RXR heterodimers.

In silico RXRα shape-based comparisons of available crystal structures suggested that different variations of the ligand-binding pocket (LBP) are induced by agonist binding or antagonist binding or by the RXRα heterodimer partner.^12^ Therefore, the development of synthetic rexinoids selectively stabilizing a specific RXR heterodimer is plausible and it potentially could reduce the undesired side effects.

Nuclear receptor-related 1 protein (Nurr1), a member of the Nur77/NGFI-B subfamily of nuclear receptors, is indispensable for the differentiation and survival of midbrain dopaminergic (mDA) neurons.^13,14^ Notably, mutations resulting in decreased Nurr1 expression have been encountered in both familial and sporadic cases of Parkinson’s disease (PD).^15,16^ Furthermore, the age-related decline in Nurr1 levels^17^ and the heightened vulnerability of Nurr1± mice to PD-associated injuries^18−20^ highlight the exceptional potential of targeting Nurr1 as an innovative therapeutic strategy for PD.^21^ The crystal structure of Nurr1 revealed a ligand-binding domain (LBD) lacking a binding cavity, potentially rendering it inaccessible to small molecules.^22^ Nurr1’s conformation is similar to that of constitutively active nuclear receptors, and its activity is regulated by its expression levels, further complicating the understanding of small-molecule activation of the Nurr1 signaling pathway.^23−28^

However, among the reported Nurr1 agonists, only a handful of ligands directly bind and modulate Nurr1 according to protein NMR studies.^29^ The antimalarial agents amodiaquine and chloroquine stand out since they physically bind to Nurr1 LBD and activate Nurr1 transcription, in conjunction with their Nurr1-independent transcriptional effects.^24,29^ Further work on the 4-amino-7-chloroquinoline core of amodiaquine, employing fragment-based and scaffold hopping strategies, led to the discovery of novel chemotypes that directly modulate Nurr1.^30,31^ Apart from the chloroquinoline-based Nurr1 agonists, a nanomolar agonist which directly binds to Nurr1 LBD has recently emerged from the elaboration of a dihydroorotate dehydrogenase inhibitor.^32^ Also, direct activation of Nurr1 may occur through the covalent binding of the dopamine metabolite 5,6-dihydroxyindole (DHI) in a previously unreported binding pocket. DHI binds directly to Nurr1-LBD forming a covalent adduct with Cys566 and stimulates the transcription of Nurr1 target genes underlying dopamine homeostasis.^33^ In a similar fashion, prostaglandin A1 (PGA1) couples covalently to Nurr1-LBD by forming a Michael adduct with Cys566 and stimulates its transcriptional function.^34^ This mode of binding to Nurr1-LBD led to the structure-guided design of indole-based noncovalent binders with markedly enhanced potency than DHI, opening a new avenue to Nurr1 agonist development.^35^

In mDA neurons, Nurr1 forms heterodimers with retinoid X receptors alpha (RXRα) and gamma (RXRγ).^36,37^ Activation of RXRα by rexinoid ligands has been shown to increase the survival of Nurr1-expressing neurons under various conditions, including serum deprivation, hypoxia, and exposure to the PD-associated toxin 6-hydroxy dopamine (6-OHDA).^37,38^ Thus, utilizing the dimerization partner RXR to activate Nurr1 holds promise for neuroprotection in PD treatment. However, the proclivity of RXRs to interact with a wide spectrum of nuclear receptors^1^ presents a hurdle in the design of selective rexinoids for the Nurr1-RXRα heterodimer as employing nonselective ligands can lead to unintended side effects.

**Figure 1:**
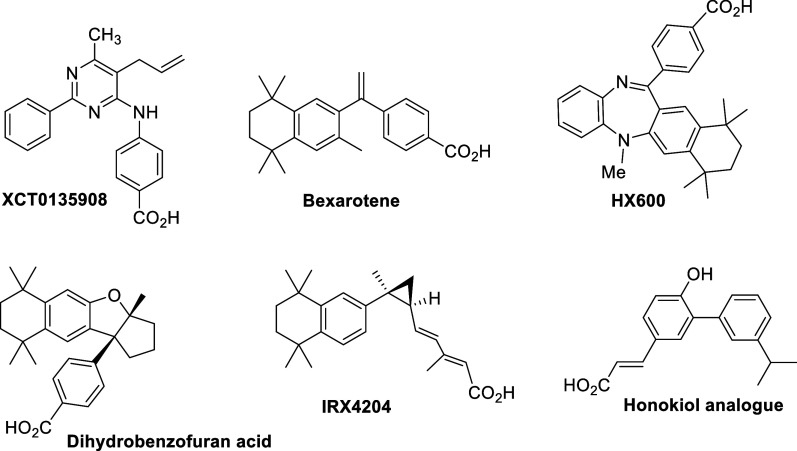
Structures of known RXR ligands that activate the Nurr1-RXRα heterodimer.

Most synthetic RXR ligands^39^ that activate the Nurr1-RXRα heterodimer, compared to other nuclear receptor dimers, trace their origins to bexarotene (Targretin), an FDA-approved antineoplastic agent that can penetrate the blood–brain barrier (BBB) (Figure 1).^40^ However, bexarotene activates all three isoforms of RXR (α, β, γ) and their heterodimers,^41−44^ leading to dose-dependent side effects including hypothyroidism,^45,46^ hyperlipidemia,^47,48^ and cutaneous toxicity.^44^ Although bexarotene has shown promising results in rescuing mDA neurons and improving behavioral function in PD and Alzheimer’s disease models,^49,50^ these findings have not been consistently replicated.^51,52^ Analogues of bexarotene, such as HX600, dihydrobenzofuran acid, and cyclopropyldienoic acid IRX4204, have also demonstrated activation of the Nurr1-RXRα heterodimer, improving movement and reducing mDA neuron loss in rodent models of PD.^53−56^ Despite the drawbacks of bexarotene, ongoing efforts focus on designing novel bexarotene analogues with minimal crossover effects on other nuclear receptors for the treatment of cancer^57,58^ and neurodegenerative disorders.^59^

A synthetic honokiol analogue featuring a biaryl scaffold selectively activates the Nurr1-RXRα heterodimer over the RXRα-RXRα homodimer by 25-fold.^60^ Its activity regarding the activation of other RXR heterodimers is not provided. However, honokiol regulates cholesterol metabolism and homeostasis by directly activating RXRβ-LXR in murine astrocytes and neuronal culture.^61^ In addition, it enhances glucose uptake through RXR-PPARγ signaling by directly binding to PPARγ,^62^ indicating off-target effects.

In contrast, XCT0135908 (XCT), a nonbexarotene-based aminopyrimidine compound identified from a chemical library, remarkably activates selectively the Nurr1-RXRα heterodimer by a factor of 37-fold compared to other RXRα heterodimerization partners.^37,63^ XCT exhibits promising neuroprotective properties in a rat neuron in vitro PD model,^38^ but it does not permeate the brain and is unstable in vivo.^64^ Herein, we describe the medicinal chemistry modifications based on the XCT scaffold and the SAR studies leading to the discovery of BRF110, a neuroprotective compound which retains the remarkable selectively for the Nurr1-RXRα heterodimer, is stable in vivo, effectively crosses the blood–brain barrier, and is devoid of hypertriglyceridemic side effects.

## Results and Discussion

### Design of Nurr1-RXRα Activators

XCT, even though its molecular size (MW) and lipophilicity (clog *P*) are in the preferred range,^65^ it does not reach the brain and it is essentially inactive in vivo.^64^ The observed poor brain penetration of XCT (B/P = 0.02) is probably associated with the increased polar surface area (PSA) due to the presence of two hydrogen bond donor (HBD) groups, a secondary amine group, and a highly polar and negatively charged carboxylic acid functionality at physiological pH. Thus, reduction of the PSA of the molecule may improve the brain penetration of XCT.^66^

Capping of the polar carboxyl group in XCT as an ester prodrug would reduce the PSA and facilitate the passive diffusion of the masked XCT into the brain. Should ester hydrolysis take place once the prodrug crosses the BBB, then release of the active XCT acid will occur in the brain. Indeed, capping of the carboxyl group in XCT as the ethyl ester enhanced its brain penetration (B/P = 1.9 at 2 h) but with complete loss of activity (EC_50_ > 100 μM) in a Nurr1-RXRα heterodimer cell-based assay, confirming the involvement of the carboxyl group in a primary interaction in the RXRα LBP. Attempts to design a series of XCT ester prodrugs with good plasma stability that could penetrate the BBB via passive diffusion or a specific transporter mechanism and could in turn gradually release XCT were not fruitful.

**Figure 2:**
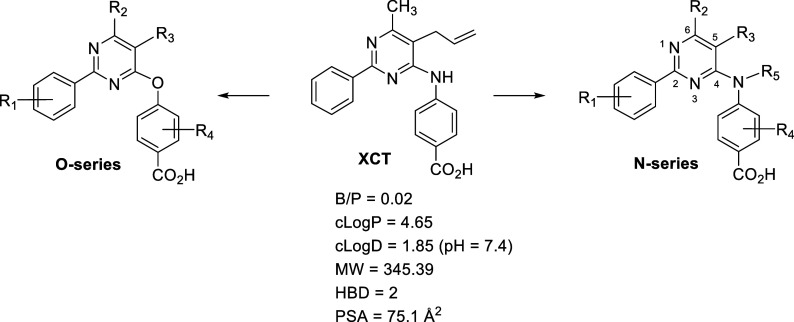
Design of structurally diverse amino and phenoxypyrimidines for SAR and brain penetration studies.

Alkylation of the secondary amine nitrogen of XCT or replacement of that nitrogen with an oxygen atom could serve as an alternative strategy to reduce the number of HBDs as well as the total PSA and provide entry to active compounds with increased brain penetration, should these structural changes not alter the active binding conformation. Therefore, in the absence of any SAR data and in the search of active and brain-penetrant compounds for our target validation studies, we embarked on the synthesis of a series of structurally diverse aminopyrimidines (N-series) and phenoxypyrimidines (O-series) in the hope to identify the structural features that influence activity and brain penetration (Figure 2).

### Chemistry

**Scheme 1:**
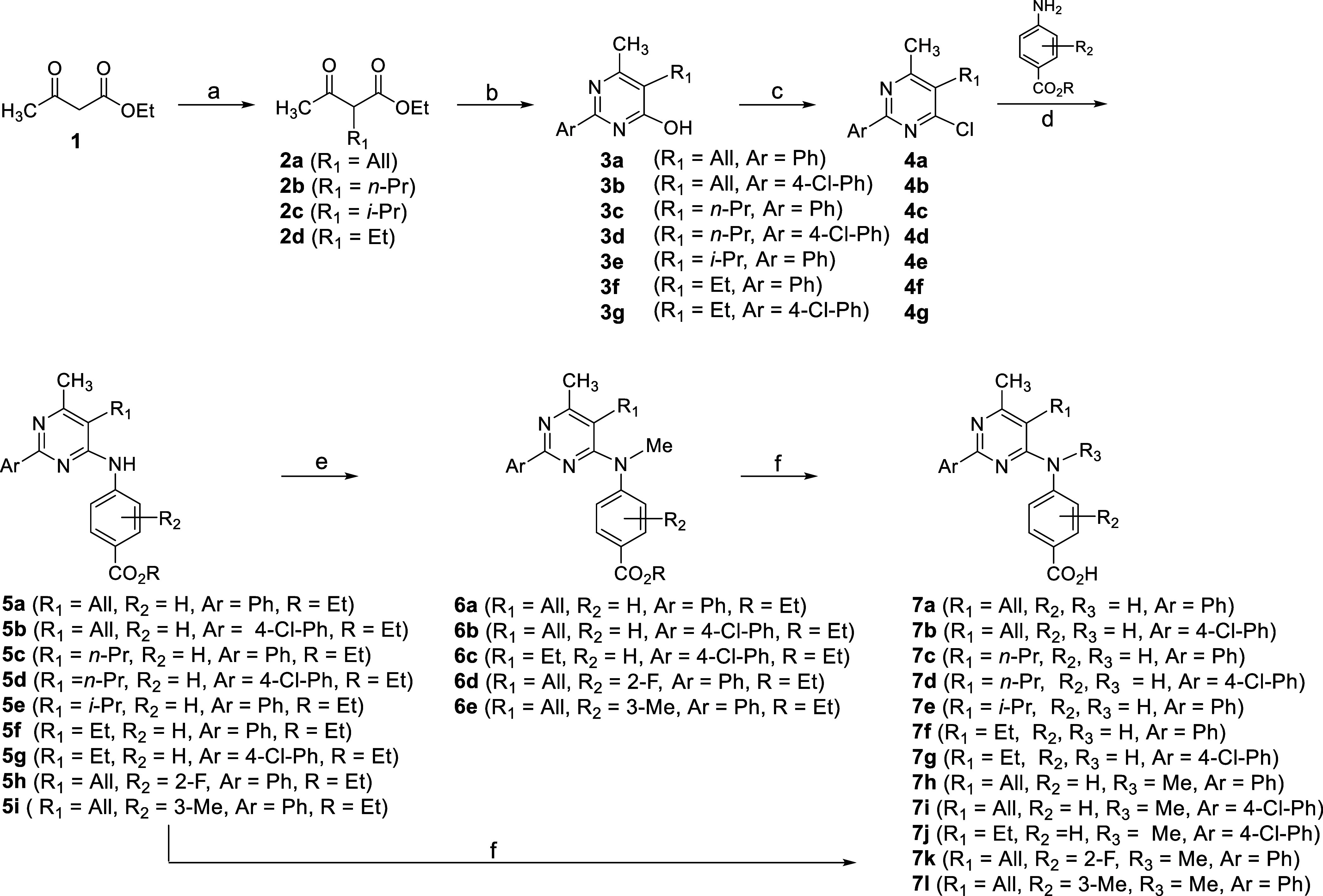
Strategy for the Synthesis of Aminopyrimidines **7** Reagents and conditions: (a) (i) NaOEt, EtOH, KI (cat), rt, then allyl bromide (for **2a**), reflux for 17 h, 51%; (ii) NaH, THF, 0 °C, then *n*-propyl bromide (for **2b**), 0 °C to rt, 16 h, 57%; (iii) *t*-BuOK, THF, *t*-BuOH (cat), 0 °C for 30 min, then R_1_-X (isopropyl bromide for **2c** and ethyl iodide for **2d**), 70 °C for 12 h, 35–49%; (b) Ar(CH = NH)NH_2_·HCl, NaOEt, EtOH, reflux, 16 h, 29–85%; (c) POCl_3_, 80 °C, 6 h, 59–81%; (d) EtOH, concd. HCl (cat), 60 °C, 24 h, 36–57%; (e) DMF, Cs_2_CO_3_, MeI, rt, 18 h, 56–83%; (f) EtOH–1N LiOH (4:1), rt, 16 h, 57–90%.

A series of aminopyrimidines **7a**–**g** possessing a methyl group at the C6 carbon of the pyrimidine ring, as in XCT, were initially prepared starting from β-ketoester **1** (Scheme 1). Alkylation of **1** with an alkyl halide under standard conditions gave ketoesters **2a**–**d**, which underwent a cyclization reaction with a benzamidine to afford pyrimidinols **3a**–**g**. Subsequent treatment with POCl_3_ resulted in chloropyrimidines **4a**–**g**, which underwent a substitution reaction with a 4-aminobenzoate substrate to afford aminopyrimidines **5a**–**i**. Then, hydrolysis of the ester group in **5a**–**g** produced acids **7a**–**g**, respectively. In order to assess whether alkylation of the secondary nitrogen in aminopyrimidines affects activity and/or enhances brain penetration, *N*-methylated analogues **7h**–**l** were prepared accordingly from the hydrolysis of esters **6a**–**e**, which were in turn derived by the methylation of compounds **5a**, **5b**, **5g**, **5h**, and **5i** respectively.^67^

**Scheme 2:**
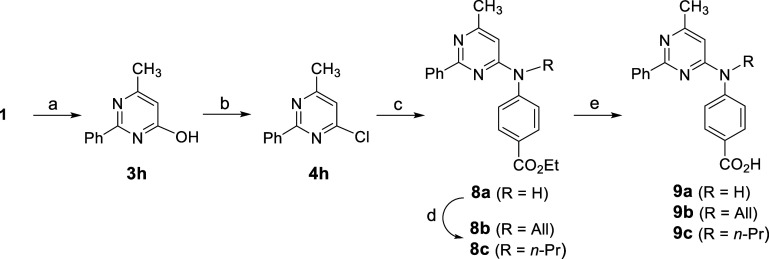
Entry to Aminopyrimidine Analogues Devoid of a C5 Substituent Reagents and conditions: (a) Ph(CH = NH)NH_2_·HCl, NaOEt, EtOH, reflux, 16 h, 30%; (b) POCl_3_, 80 °C, 6 h, 59%; (c) ethyl 4-aminobenzoate, EtOH, concd. HCl (cat), 60 °C, 24 h, 20%; (d) DMF, Cs_2_CO_3_, R-X, rt, 18 h, 64–83%; (e) EtOH–1N LiOH (4:1), rt, 16 h, 47–69%.

Truncated analogue **9a** as well as *N*-alkylated analogues **9b**–**c**, in which the C5 alkyl substituent of the pyrimidine ring was transposed to the secondary nitrogen center, were prepared in order to assess the role of the substituent at the pyrimidine C5 carbon (Scheme 2). Chlorination of pyrimidinol **3h**, derived by the condensation of β-ketoester **1** with benzamidine, afforded chloride **4h** which was smoothly converted to aminopyrimidine **8a**. Then, ester hydrolysis produced analogue **9a**. N-Alkylation of **8a** with allyl and propyl bromide generated the corresponding compounds **8b** and **8c**, which were hydrolyzed to give aminopyrimidine analogues **9b** and **9c**, respectively.

**Scheme 3:**
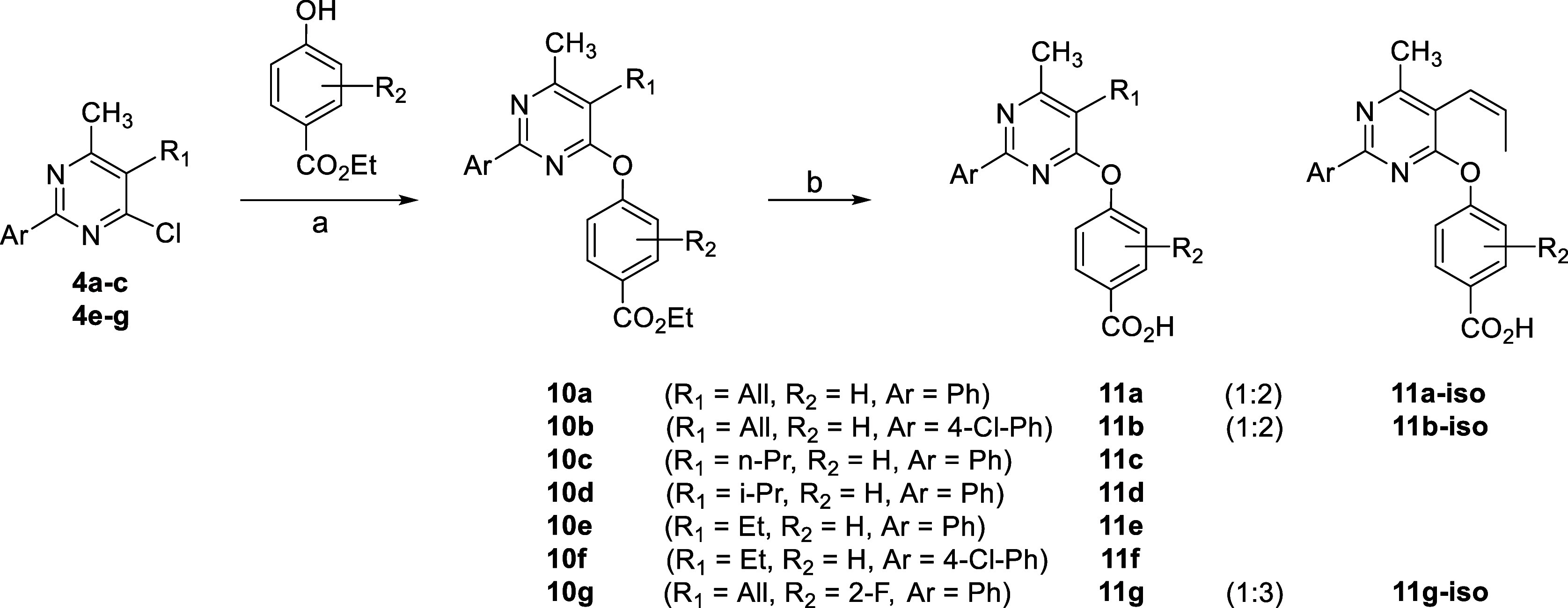
Entry to Phenoxypyrimidine Analogues **11** Reagents and conditions: (a) DMF, Cs_2_CO_3_, rt, 16 h, 29–77%; (b) EtOH–1N LiOH (4:1), rt, 16 h, 17–73%.

The phenoxypyrimidine analogues **11a**–**g** were prepared accordingly in order to investigate whether substitution of the aminopyrimidine nitrogen with an oxygen atom results in an active and brain-penetrant chemotype (Scheme 3). Indeed, treatment of chlorides **4a**–**c** and **4e**–**g** with a 4-hydroxybenzoate substrate, in the presence of Cs_2_CO_3_ and DMF, produced diaryl ethers **10a**–**g**. However, in the case of chlorides **4a** and **4b**, chlorine displacement occurred with concomitant isomerization of the C5 allyl group, resulting in phenoxypyrimidines **10a**, **10b**, and **10g** as the minor component of an inseparable mixture (∼1:1.5 to 1:4) with the corresponding C5 *cis*-propenyl isomer. Subsequent ester hydrolysis with LiOH afforded acids **11a**–**g**, where acids **11a**, **11b**, and **11g** were isolated as an inseparable mixture (1:2 to 1:3) with the propenyl isomers **11a-iso**, **11b-iso**, and **11g-iso**, respectively. This type of isomerization observed in phenoxypyrimidines, in contrast to the corresponding aminopyrimidines, is presumably attributed to an inductive effect exerted by the more electron-withdrawing oxygen atom which weakens the neighboring C–H bond in the allyl moiety and facilitates the formation of conjugated propenyl pyrimidines.

**Scheme 4:**
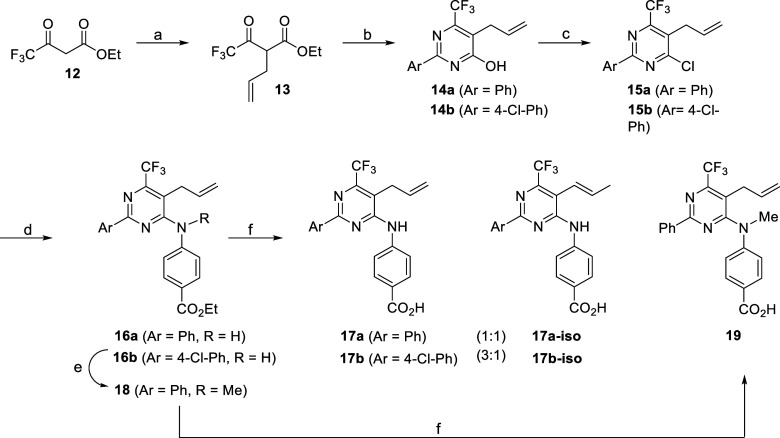
Access to Aminopyrimidines Containing a CF_3_ Group at C6 Reagents and conditions: (a) (i) NaH, THF, 0 °C, 1 h; (ii) acetone, allyl bromide, KI (cat), rt, 15 min, then at 60 °C, 48 h, 54%; (b) Ar(CH = NH)NH_2_·HCl, NaOEt, EtOH, reflux, 16 h, 62–66%; (c) POCl_3_, 80 °C, 6 h, 76–87%; (d) EtOH, concd. HCl (cat), reflux, 48–72 h, 31%; (e) DMF, Cs_2_CO_3_, MeI, rt, 18 h, 88%; (f) EtOH–1N LiOH (4:1), rt, 16 h, 42–85%.

In order to assess the effect of the substituent at C6 carbon of the pyrimidine ring in receptor binding, we investigated the synthesis of various C6-substituted compounds. Attempts to introduce other substituents at C6, larger than the methyl group (i.e., isopropyl, *t*-butyl, phenyl), were not fruitful since cyclization of the corresponding β-ketoesters with benzamidine proved problematic. However, ethyl 4,4,4-trifluoro-3-oxobutanoate (**12**) seemed an attractive substrate to explore the synthesis of analogues containing a CF_3_ group at C6 in order to assess the effect of the size of the CF_3_ group as well as its electron-withdrawing properties on receptor binding. Although the size of the CF_3_ group has been difficult to assess and this group has often been considered to be isosteric with an isopropyl substituent, its van der Waals volume is similar to that of the ethyl group.^68^ In addition, the presence of the three fluorine atoms is expected to increase the overall lipophilicity of the resulting structures and thus may lead to molecules with enhanced brain penetration.^69^ Compounds **17a**–**b** were prepared in a similar fashion starting from ketoester **13**,^70^ resulting from the alkylation of **12**(71) (Scheme 4). Cyclization of ketoester **13** with a benzamidine gave pyrimidinols **14a**–**b**, which were smoothly converted to chlorides **15a**–**b**. Then, treatment of chlorides **15a**–**b** with ethyl 4-aminobenzoate produced aminopyrimidines **16a**–**b** which underwent ester hydrolysis, accompanied by a competitive isomerization of the allyl group, to afford acids **17a** and **17b** as an inseparable mixture with the corresponding *trans*-propenyl pyrimidines **17a-iso** (**17a**/**17a-iso** = 1:1) and **17b-iso** (**17b**/**17b-iso** = 3:1), respectively.^72^ An inductive effect exerted by the electron-withdrawing CF_3_ group might as well account for the observed isomerization of the allyl moiety in aminopyrimidines **17a**–**b**. On the contrary, hydrolysis of the ester group in aminopyrimidine **18**, which was in turn derived by methylation of **16a**, resulted in aminopyrimidine **19** devoid of the propenyl byproduct (Scheme 4). However, the presence of the *N*-methyl group in compound **19** may presumably activate the pyrimidine ring and mitigate to some extent the electron-withdrawing effect of the CF_3_ group, thus prohibiting the isomerization of the C5 allyl group.

### Biological Evaluation of the Synthesized Analogues in a Nurr1-RXRα Heterodimer Transactivation Assay

The synthesized compounds were tested in cell-based assays using a luciferase reporter that measures Nurr1-RXRα transcriptional activity. Human dopaminergic SH-SY5Y cells were cotransfected with the DR5 (4×)-luciferase reporter plasmid and Nurr1 and RXRα CMV promoter-driven cDNAs. Nurr1-RXRα heterodimers recognize the DR5 element and activate luciferase transcription upon ligand (compound) binding. The cells are also cotransfected with a beta-gal plasmid that is used as a transfection efficiency control. Selected compounds at concentrations of 0.5, 2.5, and 12.5 μM were incubated with the transfected cells for 24 h. Subsequently, luciferase expression was determined in cellular extracts revealing the activity and the specificity of the compounds for the Nurr1-RXRα heterodimer. Compound **7a** (XCT) was used as a reference and found to activate the Nurr1-RXRα heterodimer with an EC_50_ = 0.3 μΜ (Table 1).

**Table 1:**
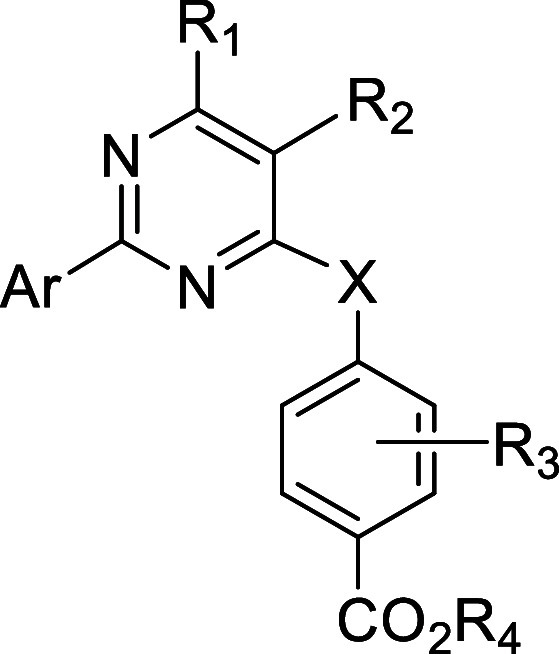

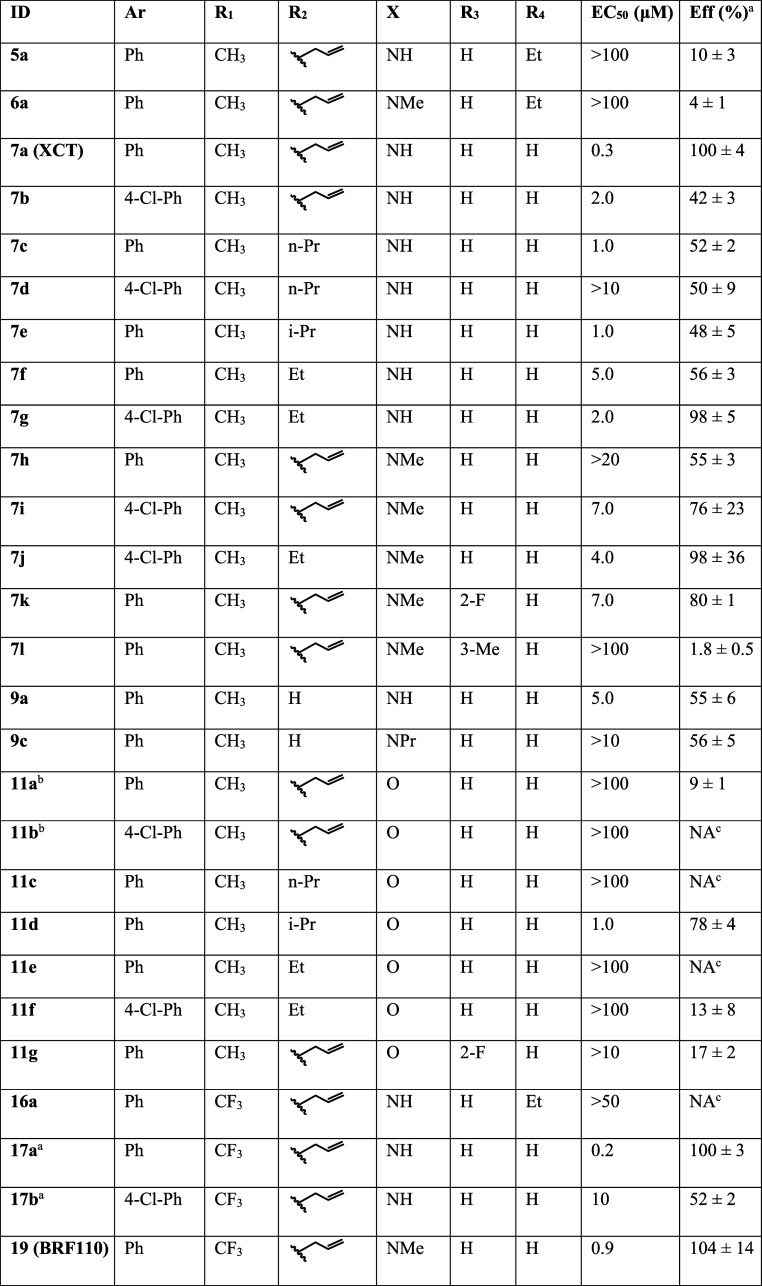
Biological Evaluation of the Synthesized Analogues in a Nurr1-RXRα Transactivation Assay^a^^,^^b^^,^^c^

a Compound efficacy at 12.5 μM compared to that of XCT (100%).

b Tested as a mixture with the isomeric propenyl compound.

c NA = not available.

Analysis of the SAR data (Table 1) showed that esters **5a** and **6a** were found to be inactive, confirming the involvement of the polar carboxyl group in a key interaction in the RXRα binding pocket. Introduction of a chlorine group at the angular C2 phenyl ring decreased the affinity for the RXRα binding pocket and resulted in a 7-fold less active compound (**7b**), compared to parent XCT compound **7a**. Substitution of the allyl with a propyl group led to a 3-fold reduction in activity (**7c**), which dropped even further by incorporation of a chlorine group in the para position at the C2 phenyl ring (**7d**). Substitution of the allyl with the isopropyl group also resulted in a 3-fold less active compound (**7e**), while substitution of the allyl with the one-carbon-shorter ethyl group gave a 16-fold less active compound (**7f**). Also, introduction of a chlorine group at the C2 phenyl ring in **7f** afforded a 7-fold less active compound (**7g**) than parent compound **7a**. *N*-Methylation of the secondary nitrogen of aminopyrimidines did not improve receptor binding and resulted in less active compounds than parent compound **7a**, as was observed for compounds **7h**, **7i**, **7j**, and **7k**. However, *N*-methylated analogue **7l** abolished activity, indicating that the presence of a methyl substituent at the benzoate group, ortho to the aniline nitrogen, is not tolerated by the receptor binding pocket.

Deletion of the alkyl substituent at the pyrimidine C5 carbon resulted in a less active analogue (**9a**), while transposition of the alkyl substituent from the C5 carbon to the secondary nitrogen center resulted in a greater loss of activity (**9c**). Phenoxypyrimidine analogues (O-series) were completely inactive compared to aminopyrimidines (N-series), as was observed for compounds **11a**–**c** and **11e**–**g**. Surprisingly, only the C5 isopropyl analogue **11d** exhibited some activity, resulting in a 3-fold less active compound than parent compound **7a**. Compound **17a** with a trifluoromethyl group at C6, isolated as an inseparable mixture (∼1:1) with its isomeric propenyl byproduct, was found to be as active as the parent XCT, suggesting that substituents larger than methyl group may be tolerated at C6. However, chlorinated analogue **17b**, also isolated as an inseparable mixture (∼3:1) with its propenyl byproduct, was 33-fold less active than XCT, indicating that substitution at the angular C2 phenyl ring is not a viable option. The assumption that the less polar and potentially brain-penetrant ester **16a**, with an electron-withdrawing trifluoromethyl group at C6, would be prone to enzymatic hydrolysis and act as a prodrug, releasing acid **17a**, did not materialize since it was found to be inactive in our transactivation assay. Although *N*-methylated aminopyrimidines were mainly inactive, surprisingly aminopyrimidine **19** with a trifluoromethyl group at C6 exhibited activity in the luciferase reporting assay and it was found to be 3-fold less active than the parent XCT.

### Brain Penetration Studies of Selected Compounds

Selected compounds were tested in vivo in order to assess their ability for brain penetration (Table 2). Ester **5a**, whose physicochemical properties were in the preferred range for BBB penetration, was found to effectively penetrate the brain. This result indicated that reduction of the HBDs with concomitant reduction of the PSA of the molecule, with regards to parent XCT, enhances brain penetration. The assumption that halogenation of XCT would increase its overall lipophilicity and the potential for brain penetration, aiming to offset the increased PSA due to the presence of two HBDs, did not materialize and halogenated analogues **7b** and **17a** were incapable of penetrating the brain. On the contrary, *N*-methylated analogues **7h** and **19** exhibited enhanced brain penetration with regards to XCT, indicating that deletion of the hydrogen bond donor capacity of the secondary amine group can lead to brain penetrant compounds with intact the carboxyl group, which is essential for activity. However, trifluoromethyl analogue **19** exhibited a 4-fold increase in brain penetration relative to nonhalogenated analogue **7h**.

**Table 2:** Brain Penetration Studies of Selected Aminopyrimidines^a^

ID	MW	HBD	HBA	rotatable bonds	PSA (Å^2^)	cLog *D*^b^ (cLog *P*)	ESOL (Log S)^c^	Ali (Log S)^c^	EC_50_ (μM)	brain conc (ng/g)^d^	blood conc (ng/mL)^d^	B/P ratio^e^
**5a**	373.45	1	4	8	64.1	5.5 (5.5)	–5.52	–6.53	>100	9.1	4.9 ± 2.1	1.9
**XCT**	345.39	2	4	6	75.1	1.9 (4.7)	–5.08	–6.05	0.3			0.02
**7b**	379.84	2	4	6	75.1	2.6 (5.4)	–5.68	–6.70	2.0	1.3 ± 0.4	24.5	0.05
**7h**	359.42	1	4	6	66.3	2.7 (5.5)	–5.24	–6.01	>20	80 ± 6.3	212 ± 55.1	0.38
**17a**^f^	399.36	2	7	7	75.1	2.6 (5.4)	–5.63	–6.59	0.2	7.3 ± 3.3	141.3 ± 6.4	0.05
**BRF110**	413.39	1	7	7	66.3	3.3 (6.1)	–5.79	–6.55	0.9	63.9 ± 58.3	35.8 ± 13.7	1.7

a Key properties and EC_50_ values are shown.

b Calculated values at pH = 7.4.

c Calculated aqueous solubility based on the ESOL and Ali models.^73,74^

d Brain and blood concentrations 2 h after IP dosing of the animals (1 mg/kg).

e Brain to plasma AUC distribution ratio 2 h after IP dosing of the animals (1 mg/kg).

f Tested as a mixture (∼1:1) with the isomeric propenyl compound.

### Modeling of Ligand Binding to RXRα

**Figure 3:**
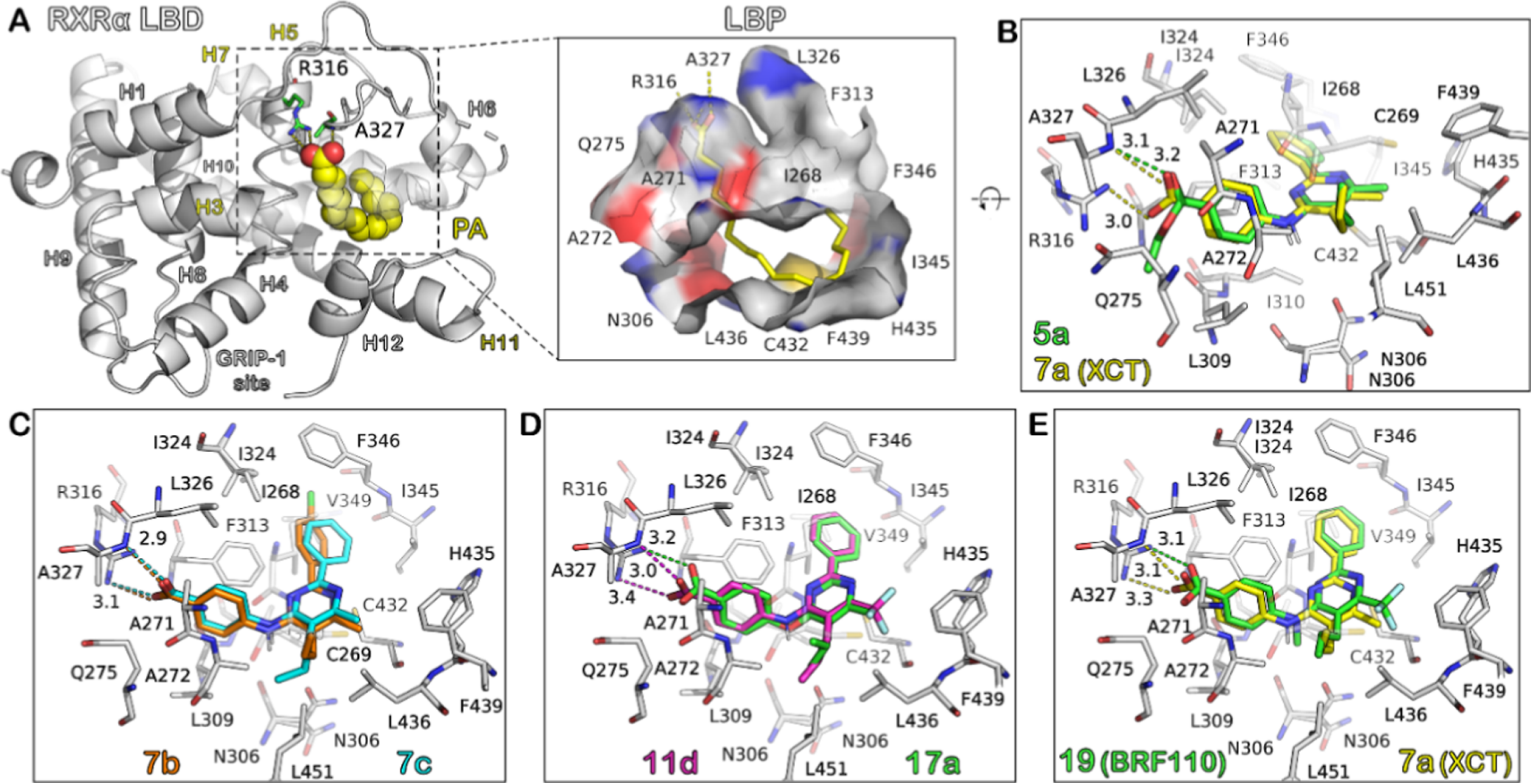
Ligand-binding domain (LBD) of the RXRα monomer in the active state. (A) X-ray structure of palmitic acid (PA)-bound RXRα LBD (PDB ID: 7a77) indicating the ligand-binding pocket (LBP), which is shown in the inset with surface representation. The two carboxylic acid-interacting residues Arg316 and Ala327 are shown as green sticks, and helices comprising residues of the LBP are highlighted. The (GRIP-1) coregulator peptide has been omitted from the binding site indicated. (B) Molecular models of XCT (**7a**) and its ethyl ester **5a** docked in the LBP of RXRα. Hydrogen bonds are indicated by dashed lines that are color-coded as the ligands and the donor–acceptor distance in Å. (C–E) Docked models of different pairs of ligands shown for comparison as in panel (B). Carbon atoms are colored as indicated in the compound ID labels, O atoms are red, N blue, S yellow, F cyan, and Cl green.

To gain insight into the binding mode of the new compounds and extract structure–activity relationships, if possible, we employed molecular docking calculations. Since there is no experimental structure of XCΤ bound to the ligand-binding domain (LBD) of RXRα, we used the recent, high-resolution X-ray crystal structure of the tertiary complex between the LBD of RXRα, the glucocorticoid receptor-interacting protein 1 (GRIP-1) coregulator peptide, and palmitic acid (PA). The structure has been resolved at 1.5 Å (PDB ID: 7a77),^75^ and it displayed the LBD of RXRα in the active, agonist-bound conformation (Figure 3A). The L-shaped ligand-binding pocket (LBP) is mainly hydrophobic, and it is composed of residues located on helices H3, H5, H7, and H11, including the β-turn residues. H3 is the element that undergoes the largest conformational change upon ligand binding to the inactive apo form of RXRα, which is mediated by the unfolding of H2 (present only in ligand-free RXRα) and the movement of H6, H11, and H12, the transactivation helix. As in all the 3 RXR isoforms, the LBD comprises mainly conserved hydrophobic residues (Figure 3A), with Phe313, Ala271, and Ala272 forming a tunnel that hosts the ligands’ carboxylic acid on one side and Leu436 and Phe439 on the other side of the cavity.^76^ The carboxylate group of the RXRα-bound PA, similar to all retinoid natural agonists, is anchored by hydrogen bonds with the side chain of the conserved Arg316 and the amide group of Ala327 (Figure 3A).

Molecular docking of the compounds that were investigated as RXRα ligands (Table 1) was performed using AutoDock Vina.^77^ Although the correlation of docking scores with experimental EC_50_ values cannot be made, we observed that activators that displayed EC_50_ values ≤1 μΜ were identified within the top 25–30% ranked poses (Supporting Information, Table S1). Considering the high structural similarity of the ligands and their consistent binding modes within the LBP of RXRα (Supporting Information, PDB file), we obtained putative SAR from the active compounds. On the other hand, most of the inactive compounds were not ranked lower than the actives in a consistent way, probably as a result of their equally potent binding mode within the LBP of RXRα. In such cases, there are probably other effects that account for the observed EC_50_ results obtained, other than binding affinity for RXRα. We cannot also rule out the possibility that the synthesized analogues bind to Nurr1. However, their predicted binding modes do not exhibit any potential for covalent binding with Cys566 or high affinity binding to other sites (Supporting Information, Figure S1). In particular, BRF110 displayed Nurr1 bound poses with scores of −6.5 up to −7.2 kcal/mol, which indicates a 30-to-100-fold lower potential (higher dissociation constant) compared with the docked score of −9.2 kcal/mol for RXRα-bound BRF110 (Supporting Information, Table S1). Therefore, we limited our analyses to the highest affinity-bound conformations of the ligands to RXRα.

The top-ranked docked pose of XCΤ (Figure 3B) displays the characteristic anchoring of its carboxylate group by Arg316 and the amide group of Ala327, whereas the benzoate ring is stacked with Phe313 in a T-shaped fashion (ring centroids distance of 4.5 Å). The secondary amine group does not participate in any polar contact, while the adjacent pyrimidine ring is positioned between Ile268 and Ile310. Its allylic and methyl substituents make extensive hydrophobic contacts with Cys269, A272, Leu451, Leu436, and Cys432, respectively, whereas its phenyl group is placed between Ile324, Val349, and the side chain rings of Phe313 and Phe346 (Supporting Information, Figure S2). A very similar bound pose was calculated for the ethyl ester of XCT, compound **5a**, but lacking the H-bonding interaction with Arg316 (O–N distances of 3.9–4.1 Å), which renders it inactive. From the remaining aminopyrimidine analogues **7b**–**7l**, most compounds were found to be active in the transactivation assay, except for **7d**, (>10 μΜ), **7h** (>20 μΜ), and **7l** (>100 μΜ). The most potent compounds, **7c** and **7e**, are predicted to bind RXRα in a very similar fashion to that of XCT, whereas the chloride substituent of **7b** has a negative effect on the estimated binding affinity and the experimental activity with respect to **7c** (Figure 3C). If the observed transactivation activity of the compounds is mainly dependent on their RXRα binding affinity, then it is very probable that the differences observed in their activities result from a sum of subtle differences in their interactions with key residues of the LBD.

With regard to the phenoxypyrimidine analogues **11a**–**11g**, their predicted poses were also similar to that of XCT irrespective of the pyrimidine substituents. However, the H-bonding interaction with Ala327 was perturbed (O–HN distance >3.9 Å) probably as an effect of the amine-to-ether substitution. This loss could account for the observed inactivity of this series, albeit **11d** was found to be active (Figure 3D). Although the closest distance between the carboxylate O atoms of **11d** and the amide N of Ala327 is predicted to be 3.6 Å, its estimated binding affinity to RXRα is higher compared to the other phenoxypyrimidine analogues. This indicates that there are other effects, except for the binding affinity, which play a role in their activity.

The CF_3_-substituted analogue of XCT, compound **17a**, is predicted to bind in a very similar pose to that displayed by its parent compound (Figure 3D). In agreement with the experimental activity of **17a**, its estimated binding affinity for RXRα is also very similar to that of XCT (Table S1). *N*-Methylation of **17a** to yield **19** (BRF110) resulted in a small reduction of activity, which is also predicted to be accompanied by small changes in the docked position of **19** within the LBD of RXRα (Figure 3E). Comparison of their bound poses with that of XCT reveals only minor shifts in their atomic positions (∼0.5 Å), with the most pronounced differences observed at the CH_3_/CF_3_ substituent of their pyrimidine rings (C-to-C distance of 1.2 Å). These subtle differences cannot account for the exact differences between the activity of **17a** and **19** with that of the parent compound, which are however small (EC_50_ (μΜ): 0.2 and 0.9, versus 0.3, respectively).

### Comparison of BRF110 and XCT Selectivity for the Nurr1-RXRα Heterodimer

**Figure 4:**
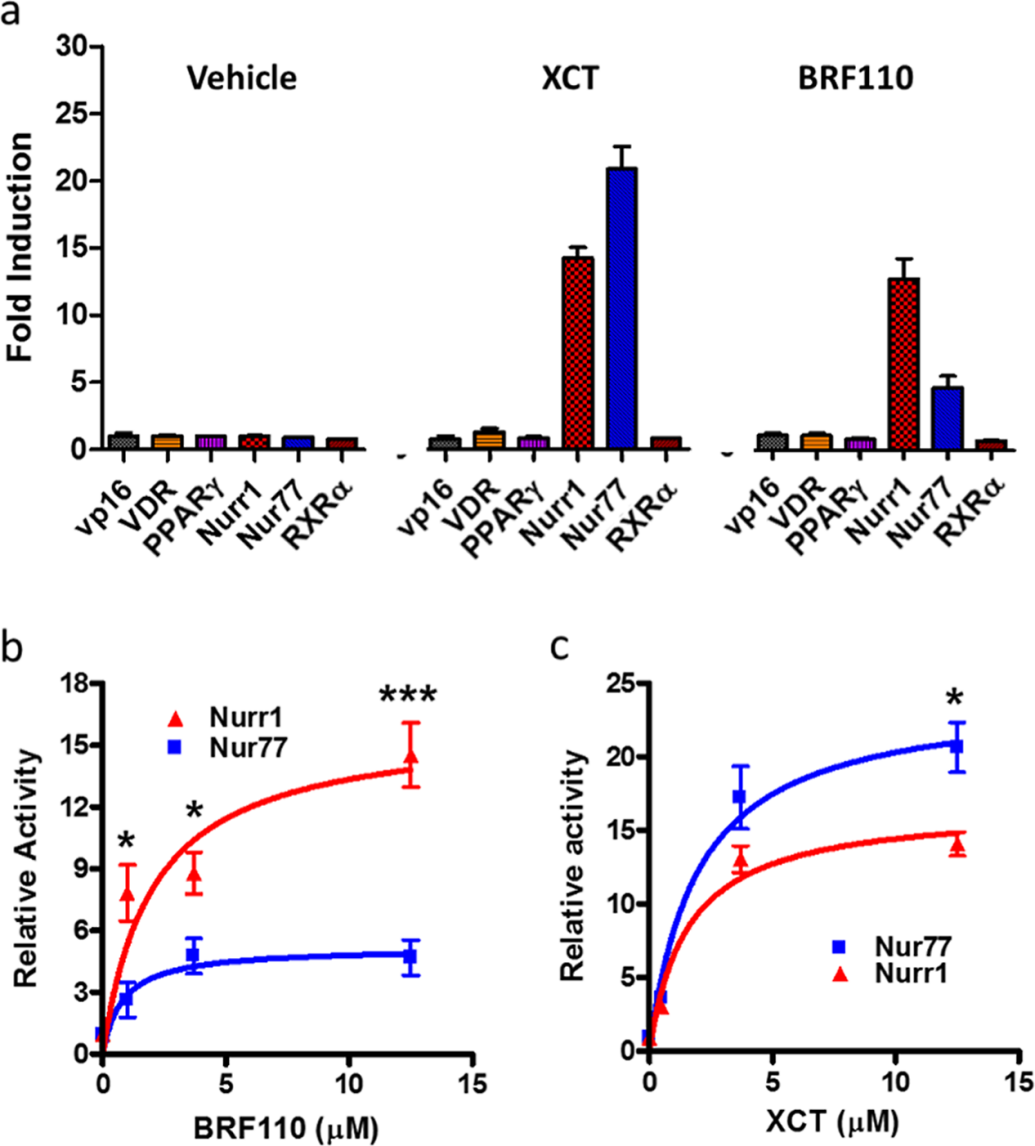
Comparison of selectivity between BRF110 and XCT. (a) Luciferase reporter assays of the chimeric proteins comprising various RXRα heterodimers and activation with the vehicle, XCT, or BRF110 (12.5 μM). (b) Nurr1GAL4-RXRαVP16 and Nur77GAL4-RXRαVP16 heterodimer activation by the indicated concentrations of BRF110. (c) Nurr1GAL4-RXRαVP16 and Nur77GAL4-RXRαVP16 heterodimer activation by the indicated concentrations of XCT. Data are presented as mean ± SEM, *n* ≥ 3, *p < 0.05, **p < 0.01.

A paramount consideration during the generation of in vivo stable and brain-penetrant rexinoids was the maintenance of the remarkable selectivity of the parent XCT compound for the Nurr1-RXRα heterodimer.^37^ BRF110 indeed does not activate the Nurr1-RXRγ heterodimers nor does it activate RXRα heterodimers with VDR, RXRγ, PPARγ, or RXRα homodimers. RXRα forms heterodimers with two members of the NR4A subfamily of nuclear receptors, Nurr1 and Nur77, and BRF110 partially activates the Nur77-RXRα heterodimers in SH-SY5Y cells.^64^ Thus, we proceeded to characterize the relative selectivity of BRF110 and XCT on Nurr1-RXRα and Nur77-RXRα heterodimers in SH-SY5Y cells. We fused the LBDs of Nurr1 or Nur77 to the GAL4 DNA-binding domain to create chimeric proteins, while the LBD of RXRα was fused to VP16. DNA expression constructs encoding these molecular chimeras, along with several others encoding other nuclear receptors (VDR, PPARγ, RXRα) capable of forming dimers with RXRα, were cotransfected in pairs with the RXRα-VP16 along with a GAL4-responsive luciferase reporter and control expression vectors in SH-SY5Y cells. The transfected cells were stimulated with BRF110 (12.5 μM) or XCT (12.5 μM) for 24 h, and transcriptional activation was measured by determination of luciferase activity (Figure 4a). Both compounds were able to activate the Nurr1-RXRα and the Nur77-RXRα heterodimers but none of the control RXRα heterodimers with VDR, RXRγ, PPARγ, or RXRα homodimers, displaying similar selectivity. Nevertheless, XCT achieved higher maximal levels at a lower concentration, indicating lower EC_50_. We proceeded to analyze in further detail the activity of XCT and BRF110 on the Nurr1GAL4-RXRαVP16 and Nur77GAL4-RXRαVP16 heterodimers using a range of different concentrations (0.5, 3.7, and 12.5 μM) for each compound. BRF110 strongly activated the Nurr1GAL4-RXRαVP16 chimeras, while maximal Nur77GAL4-RXRαVP16 heterodimer activation was lower by ∼68% (*p* = 0.001, *t*-test) (Figure 4b). On the contrary, XCT strongly activated Nur77GAL4-RXRαVP16 while its maximal activation of the Nurr1GAL4-RXRαVP16 heterodimer was decreased by ∼32% (*p* = 0.0158, *t*-test) (Figure 4c). The above experiments indicate that even small changes (less than 10-fold) in the ligand concentration can alter heterodimer selectivity and that BRF110 is highly selective for the Nurr1-RXRα heterodimer, even more so than XCT.

### Neuroprotection in Cell Lines: Comparison between BRF110 and XCT

**Figure 5:**
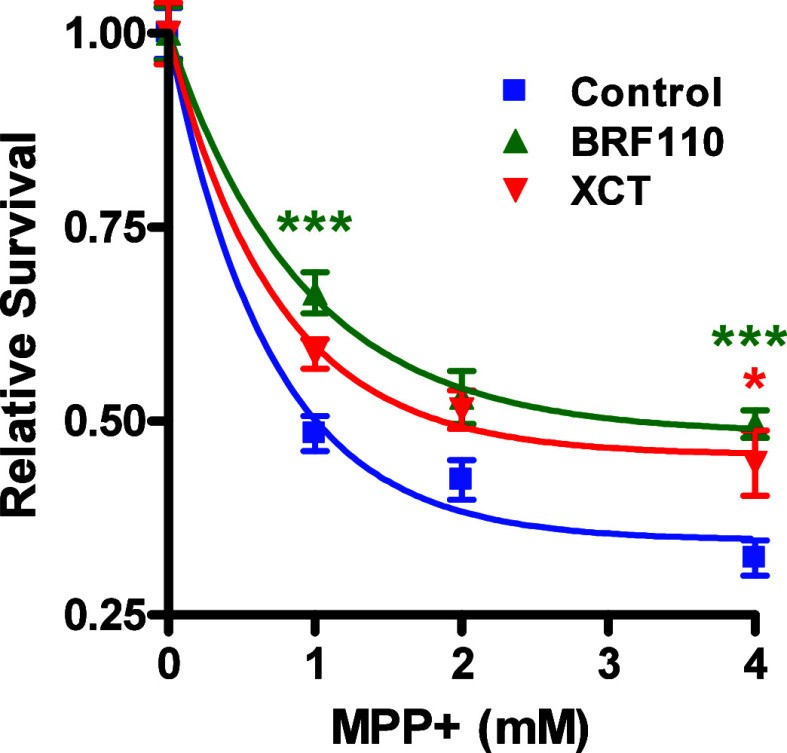
In comparison to vehicle (blue)-treated cells, BRF110 (green) and XCT (red) protect mouse Neuro-2a neuroblast cells from varying concentrations of MPP^+^ toxicity. Data are presented as mean ± SEM, *n* = 8, *p < 0.05, and ***p < 0.001.

Increased expression or activation of Nurr1 is correlated with increased survival of dopaminergic cell cultures and midbrain dopamine neurons against PD-causing toxins, while Nur77 expression is not associated with PD, and Nur77 knockdown enhances cell survival.^78^ We compared the relative neuroprotective properties of BRF110 and XCT (both used at 12.5 μM) in Neuro-2a mouse neuroblast cells. Cell death was induced by the mitochondria complex I inhibitor MPP^+^ (1-methyl-4-phenylpyridinium), the active metabolite of MPTP (1-methyl-4-phenyl-1,2,3,6-tetrahydropyridine).^79^ BRF110 treatment significantly increased the survival of the cells against varying concentrations of MPP^+^ (Figure 5). Most of the surviving MPP^+^-treated cells receiving the vehicle died, while the few surviving ones remained attached to the plate but were rounded and had lost all projections, indicating severely impaired function. On the contrary, BRF110- or XCT-treated cells appeared healthy, flattened, and well attached and their projections remained intact. XCT efficacy against MPP^+^ elicited Neuro2A mouse dopaminergic cell toxicity appeared somewhat lower (38.7%), but not statistically significant, than that of BRF110 (52.5%) (2 way ANOVA, *p* = 0.0001, *n* = 8) (interaction^BRF110^*p* = 0.0063; interaction^XCT^*p* = 0.1835). Lastly, incubation of the cells for 24 h with 50 μM of each of the compounds was equally neuroprotective and toxic effects were not observed (not shown).

### Oral Bioavailability of BRF110

The molecular characteristics of BRF110 (Table 2) are consistent with oral bioavailability;^80,81^ thus, we administered BRF110 (5 mg/kg) to mice (*n* = 5) by gavage. Intraperitoneal administration of BRF110 (1 mg/kg) to mice (*n* = 3) was used as a reference. The concentrations of BRF110 in the blood and the brain, at 1, 2, and 4 h after dosing, were determined by LC–MS/MS. Table 3 shows the blood and brain values obtained after oral dosing.

**Table 3:** BRF110 Concentration after Oral Administration (PO)

	blood average ± SEM (ng/mL)	brain average ± SEM (ng/g)
1 h	312.3 ± 66.8	434.6 ± 77.1
2 h	278.1 ± 16.6	529.5 ± 134.2
4 h	49.9 ± 2.8	114.3 ± 8.2

Our results indicate that BRF110 administered to mice by gavage is orally bioavailable. Within 1 h post-PO dosing, BRF110 was detectable in the blood. At 2 h, its concentration decreased by only ∼10%, indicating continuing absorption. BRF110 readily crossed the BBB, and at 1 h post-PO, its concentration was higher than the blood, indicating accumulation in the brain. After 2 h, the concentration of the compound was further increased in the brain, indicating continuing distribution. Total amounts of BRF110 PO compared to IP dosing revealed a relative bioavailability (*F*_rel_ = area under the curve, AUC oral/AUC IP × 100%) of ∼70%. The brain/blood distribution AUC ratio was calculated to be 1.7 (Table 2). It should be noted that in this study, vehicle composition for either mode of administration was not optimized and additional future experimentation could improve these values.

### BRF110 Regulates the Expression of BDNF by Activating Nurr1-RXRα

**Figure 6:**
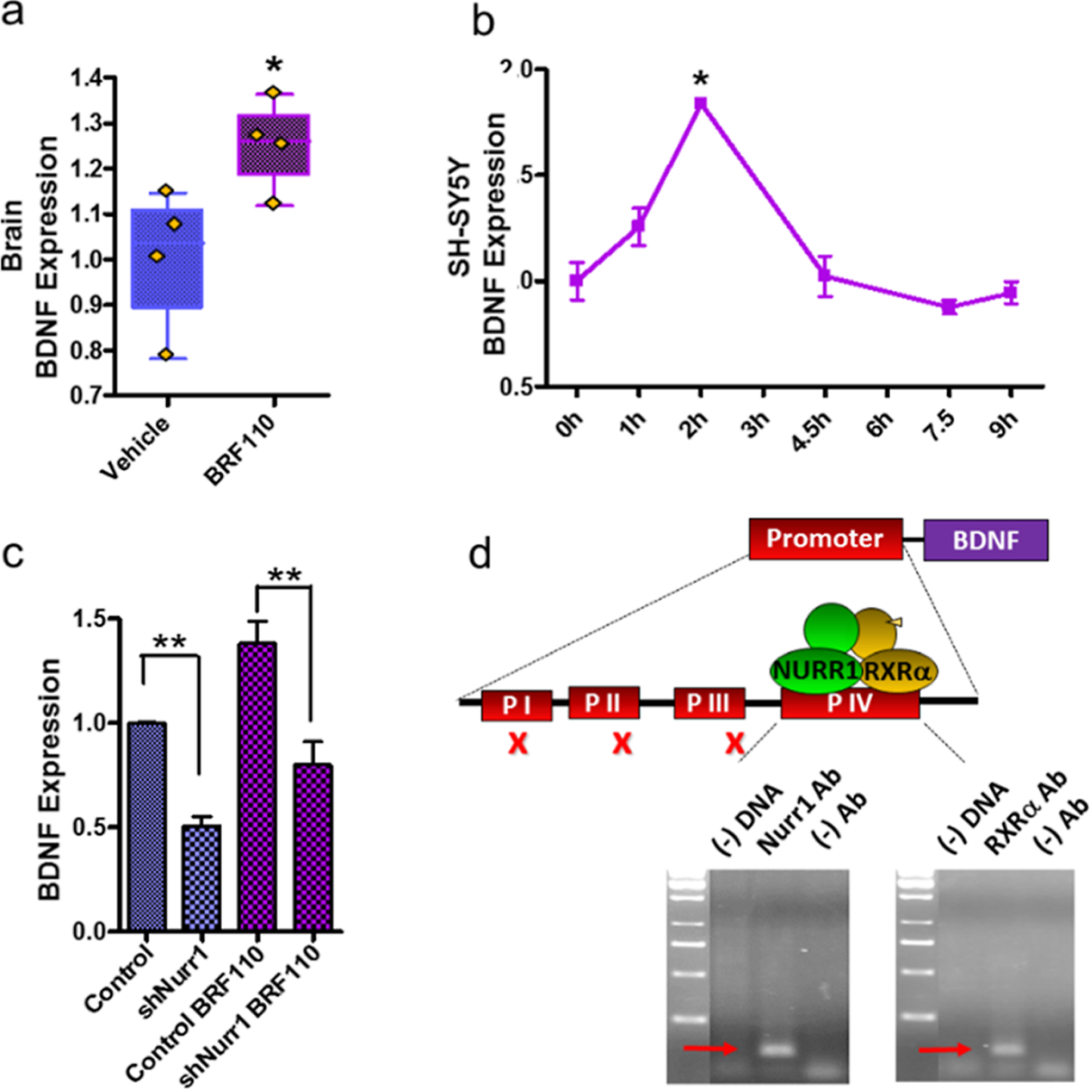
BRF110 activates Nurr1-RXRα and regulates BDNF expression. (a) Brain BDNF expression after oral administration of BRF110 (15 mg/kg) to mice. (b) qPCR of temporal BDNF expression after stimulation with BRF110 in SH-SY5Y cells. (c) SH-SY5Y cell endogenous Nurr1 knockdown decreases basal BDNF expression and activation by BRF110. (d) ChIP-PCR of direct binding of both Nurr1 and RXRα to BDNF promoter IV. The lanes for the samples treated with the respective antibodies are indicated as (−) DNA for no input DNA control and (−) Ab for no specific antibody control. Data are presented as mean ± SEM, n = 8, *p < 0.05, and ***p < 0.001.

We have previously shown that BRF110 can alleviate dopaminergic neuron damage in vitro and in vivo.^64^ We therefore endeavored to better characterize its actions and putative clinical importance. The brain-derived neurotrophic factor (BDNF) is a known downstream target of Nurr1.^82^ However, the possible dependence on an RXR partner has not been investigated nor is it known whether the expression of BDNF can indeed be regulated by BRF110 and therefore account for its neuroprotective effects. For this reason, we explored the role of Nurr1-RXRα and BRF110 in the expression of the BDNF gene in vitro and in vivo. Oral administration of BRF110 (15 mg/kg) to mice resulted in a ∼ 25% increase in BDNF mRNA levels (*t*-test, *p* = 0.0306, *n* = 4, Figure 6a) in the brainstem midline 4 h postdosing compared to vehicle controls, indicating that, in addition to Nurr1, RXR may also be involved. In SH-SY5Y dopaminergic cells, BRF110 (12.5 μM) induced a transient increase in BDNF expression, reaching a maximum of ∼1.8-fold after 2 h (*p* = 0.0242), which subsequently decreased to baseline within 5 h (Figure 6b). This experiment addresses questions related to the temporal dynamics of activation and indicates that these cells could be used to determine the BRF110 mechanism of action. Knockdown of endogenous Nurr1 in SH-SY5Y cells via retroviral-mediated shRNA reduced by a similar degree both basal BDNF expression as well as its induction by BRF110 (one-way ANOVA, *n* = 4, p < 0.0001, Figure 6c). These experiments demonstrate a direct dependence of the basal BDNF expression, as well as its upregulation by BRF110, on Nurr1 as an RXRα partner.

Analysis of the human BDNF promoters I, II, III, and IV using the JASPAR CORE database of experimentally defined regulatory DNA element profiles^83^ predicted a 13-nt (ACGTCAAGGCACC, relative score 0.7714198) in promoter IV as a potential Nurr1-RXRα binding site.^84^ CLUSTAL Omega alignments revealed that this 13-nt sequence resides in the first of two 50-bp contiguous regions of the BDNF promoter IV and it is 100% conserved between humans, primates, ungulates, and bats. The identity of this sequence decreases to 98% in rodents, nevertheless, the demonstrated evolutionary conservation implies a crucial role in the regulation of BDNF expression. Chromatin immunoprecipitation (ChIP) and PCR analysis were used to examine the Nurr1 and RXRα binding to BDNF promoter IV^85^ when treated with BRF110 (12.5 μM). Indeed, both Nurr1 and RXRα were found to bind to promoter IV sequences 2 h after BRF110 treatment but not to the BDNF promoters I, II, and III (Figure 6d). These results strengthen the hypothesis of BDNF promoter-specific regulation by BRF110 through direct binding of both Nurr1 and RXRα to promoter IV.

Together, the regulation of BDNF expression in cells, coupled with the demonstration of BDNF promoter binding, helps to establish a direct link between BRF110 and this target gene in vivo. The molecular mechanism of BRF110-mediated Nurr1-RXRα activation is debatable. Recent experiments provide data supporting a transactivation model where the binding of BRF110 or other rexinoids promotes the dissociation of the Nurr1-RXRα heterodimer and the release of transcriptionally activated Nurr1.^86^ These experiments utilized the monomeric Nurr1 DNA response element NBRE where RXRα cannot bind; thus, it is possible that the mechanism of BRF110 Nurr1-RXRα heterodimer activation is different when both proteins are bound to DNA. Indeed, our ChIP-PCR data indicates that BRF110-induced transcriptional activation of BDNF correlates with both Nurr1 and RXRα protein binding to promoter IV and supports the classic transactivation model of ligand-induced heterodimer stabilization.

### Effect of BRF110 on Hypertriglyceridemia

Bexarotene, the only FDA-approved rexinoid, is used to treat cutaneous T-cell lymphoma. However, nearly 80% of patients receiving bexarotene develop moderate to severe hypertriglyceridemia, which significantly increases the risk of pancreatitis and cardiovascular disease.^47,48^ This adverse effect is attributed to the bexarotene activation of the liver LXRα-RXRα heterodimer.^42,43^

**Figure 7:**
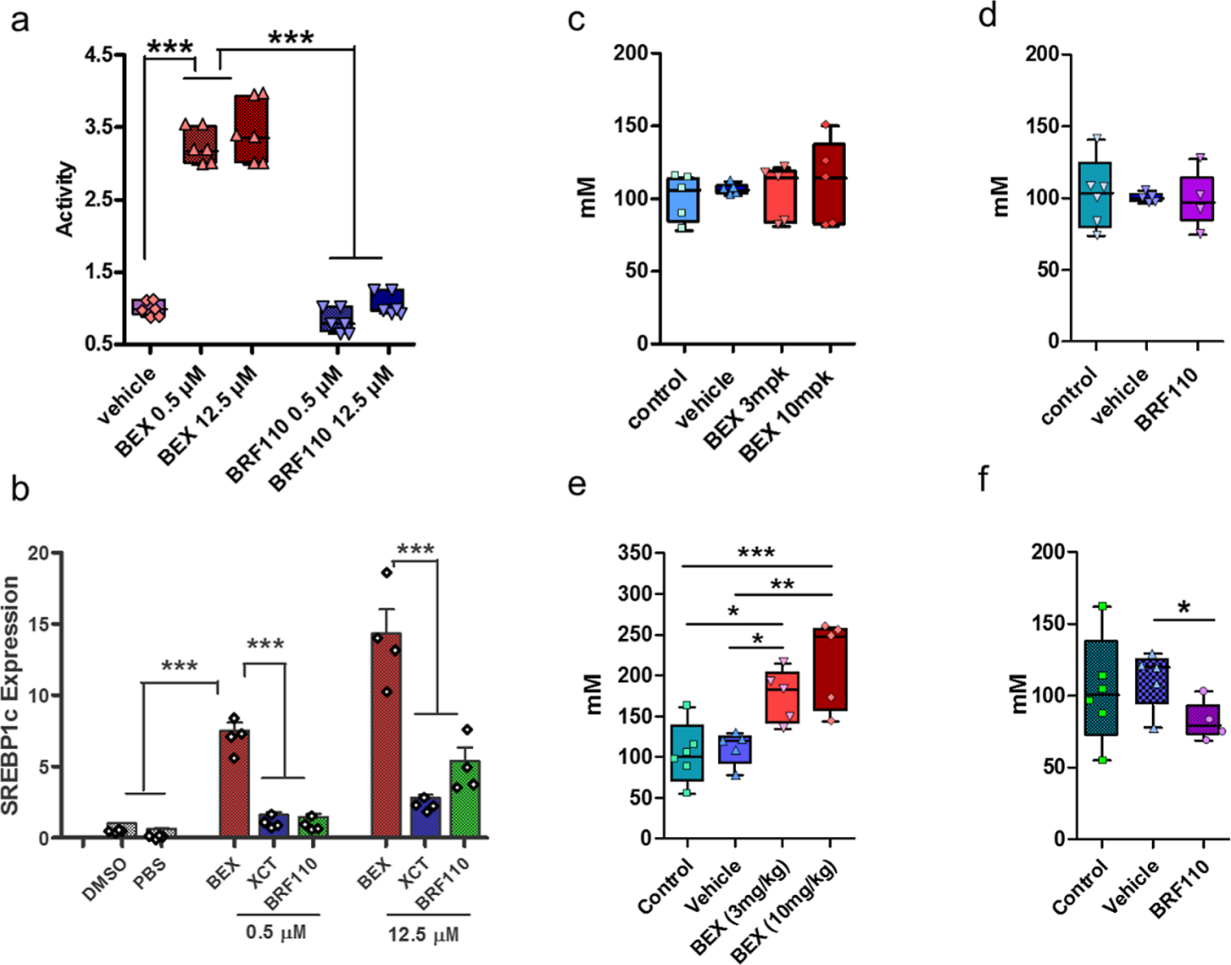
Effect of BRF110 on hypertriglyceridemia. (a) Luciferase assays: Comparison of LXRα-RXRα heterodimer transactivation of the ABCA1 reporter by bexarotene or BRF110. (b) qPCR: Induction of SREBP-1c expression by bexarotene, BRF110 or XCT in HepG2 cells. Cholesterol (c,d) and triglyceride (e,f) levels of mice treated with bexarotene (c,e) (3 and 10 mg/kg) or BRF110 (d,f) (10 mg/kg), or vehicle. The compounds and the vehicle were administered IP daily for 5 consecutive days. Blood was collected before compound administration (Control) and 3 h after the last injection on day 5. Data are presented as mean ± SEM, *n* ≥ 5, *p < 0.05, **p < 0.01, and ***p < 0.001.

XCT does not activate the LXRα-RXRα heterodimer in a transactivation assay.^37^ Thus, similarly, we examined whether cotransfection of the cDNAs encoding the two components of the heterodimer, as well as an ABCA1 promoter-driven luciferase reporter in embryonic kidney HEK 293 cells, would be activated by bexarotene or BRF110 (0.5 and 12.5 μM). This experiment showed the markedly reduced capacity of BRF110 to stimulate the LXRα-RXRα-responsive luciferase reporter which was not different than the vehicle control at both concentrations (one-way ANOVA, *n* = 6, p < 0.0001) (Figure 7a). On the contrary, bexarotene readily activated the ABCA1 reporter reaching >90% of the maximal activity at a 0.5 μM concentration (one-way ANOVA, *n* = 6, p < 0.0001) (Figure 7a). These data show that in cells expressing the two components of the LXRα-RXRα heterodimer, stimulation with BRF110 cannot activate the expression of the LXRα-RXRα responsive target reporter, making it unlikely that it can elicit hypertriglyceridemia in vivo.

Bexarotene-triggered hypertriglyceridemia in vivo is attributed, at least in part, to the induction of the Sterol Regulatory Element Binding Protein-1c (SREBP-1c) transcription in the liver and rexinoids that exhibit lowered triglycerides also exhibit lowered SREBP-1c induction.^87−89^ To test the effect of BRF110 on SREBP-1c, we treated HepG2 hepatic cells with 0.5 or 12.5 μM bexarotene, BRF110, or XCT for 24 h. SREBP-1c expression was increased in a concentration-dependent manner by bexarotene up to ∼23-fold over controls (PBS, DMSO), while BRF110 or XCT triggered significantly smaller increases, up to ∼5-fold (one-way ANOVA, p < 0.0001) (Figure 7b).

We next investigated whether the increased selectivity of BRF110 for the Nurr1-RXRα heterodimer could also account for its inability to trigger triglyceride elevation after repeated daily dosing in vivo. Mice were given bexarotene (3 or 10 mg/kg), BRF110 (10 mg/kg), or vehicle IP daily for 5 consecutive days. Blood samples were collected from all mice the day before starting treatment and 3 h after the final injection on day 5.

Cholesterol measurements showed no significant differences between the control group and mice treated with either bexarotene (Figure 7c) or BRF110 (Figure 7d). In contrast, analysis of triglyceride levels revealed that, as expected, bexarotene treatment resulted in a dose-dependent elevation in triglycerides, with increases of approximately 90% (one-way ANOVA, *p* = 0.015, 3 mg/kg) and 136% (*p* = 0.0014, 10 mg/kg) compared to the control group (one-way ANOVA, *p* = 0.0009) (Figure 7e). On the other hand, animals treated with BRF110 did not exhibit elevated triglyceride levels; instead, they showed a modest decrease of approximately 25%, which was significant compared to vehicle-treated animals by *t*-test (*p* = 0.049) but not by ANOVA (Figure 7f). No significant difference was observed between triglyceride levels in sera collected at day 0 (101.1 ± 17.36, *n* = 5) and those from vehicle-treated (111.1 ± 9.023, *n* = 5) or untreated control animals (124.1 ± 5.872, *n* = 7) on day 5. LC–MS/MS analysis showed BRF110 concentrations of 35.46 ± 0.137 ng/mL (*n* = 3) in the analyzed sera of day 5.

Triglyceride elevation has previously been documented with oral administration of bexarotene.^49^ Therefore, to extend the previous data to a different treatment mode, we administered BRF110 (14 mg/kg daily) or vehicle by gavage to mice for four consecutive days. Triglyceride levels measured 4 h after the final dose showed no statistically significant changes between day 0 and day 4 for either BRF110- or vehicle-treated animals. LC–MS/MS analysis confirmed the expected concentrations of BRF110 in the serum (49.60 ± 24.98 ng/mL, *n* = 5). These results indicate that BRF110 does not affect triglyceride levels in mice following repeated dosing either IP or by oral route, a fact that we attribute to its selective activation of the Nurr1-RXRα and not the LXR-RXRα heterodimer. Of note, the observed reduction in triglyceride levels in BRF110-treated mice compared to vehicle-treated controls warrants further investigation.

## Conclusions

The pleitropic transduction pathways affected by RXRs (α, β, and γ) offer great potential to develop novel treatments for cancer, metabolic regulation, and neurodegenerative disorders.^1−8^ However, the side effects of RXR agonists, such as thyroid axis suppression and severe triglyceride elevation, have severely tempered their clinical potential.^45−48^ In silico analyses have suggested that RXR undergoes conformational changes, resulting in distinct binding pocket variations depending on its heterodimer partner.^12^ Exploring this insight, we pursued the design of novel synthetic rexinoids with enhanced heterodimer selectivity, to circumvent the detrimental effects associated with nonspecific heterodimer activation.

The unprecedented selectivity of the XCT0135908 ligand for the Nurr1-RXRα heterodimer, in stark contrast to bexarotene, prompted us to try to resolve its shortcomings of in vivo stability and blood–brain barrier impermeability, without sacrificing its neuroprotective properties and its unique target selectivity. Through meticulous SAR experimentation, we retained the molecular scaffold of XCT0135908 and arrived at BRF110, a promising molecule that has remarkable Nurr1-RXRα selectivity,^64^ directly upregulates expression of the neuroprotective factor BDNF, and is devoid of undesirable hypertriglyceridemic side effects, therefore effectively resolving these challenges.

Our results showcase a pivotal role for heterodimer-selective rexinoid scaffolds as the foundation for developing compounds to precisely modulate therapeutic pathways and at the same time to mitigate the burden of side effects. This approach may be used to increase the clinical development of rexinoids and to significantly improve the challenging therapeutic landscape for RXR-targeting molecules.

## Experimental Section

### Chemistry

#### General Experimental Methods

All commercially available chemicals were used without further purification. All reactions were performed under an argon atmosphere with dry solvents under anhydrous conditions, unless otherwise noted. Tetrahydrofuran (THF), diethyl ether (Et_2_O), methylene chloride (CH_2_Cl_2_), acetonitrile (CH_3_CN), dimethylformamide (DMF), and dimethyl sulfoxide (DMSO) were purchased in anhydrous form and used without further purification. Air- and moisture-sensitive liquids were transferred via syringe. Organic solutions were concentrated by rotary evaporation at 40 °C. Flash-column chromatography was performed with silica gel 60 (230–400 mesh) as described by Still et al.^90^ Thin-layer chromatography (TLC) was performed on precoated silica gel 60 F254 plates, and the eluent used is reported in parentheses. TLC plates were visualized by exposure to ultraviolet light (UV) and/or submersion in an aqueous potassium permanganate solution (KMnO_4_/H_2_SO_4_) followed by heating.

HPLC purification was performed by reverse-phase HPLC on a fully automated Shimadzu preparative system comprising two Shimadzu LC-8A solvent delivery pumps coupled to a communication bus module (Shimadzu CBM-20A), which was used to control sample injection (Shimadzu SIL-10AP autosampler) and automatic collection (Shimadzu FRC-10A fraction collector). Separation was performed on a Jupiter 10u Proteo 90A column (250 × 10.0 mm, 7.56 μm particle size). The flow rate was 21 mL/min. Peaks were detected at λ = 254 nm at room temperature. The solvent system consisted of two components, A (acetonitrile) and B (0.1% TFA in water). The samples were dissolved in acetonitrile. Analytes were identified from their retention time and absorbance spectrum profile recorded on a Shimadzu SPD-M20A diode array (DAD) detector.

HPLC analysis for compound purity assessment was performed on an Agilent Eclipse XDB-C18 column with a particle size of 5 μm (15 cm × 4.6 mm I.D.), using a chromatographic system comprising an Agilent 1200 series liquid chromatograph equipped with a 20 μL sample loop injector. The peaks representing the target analyte(s) were recognized both by the retention time and their spectrum pattern recorded on a diode array detector working under Agilent ChemStation chromatography software. Elution was accomplished isocratically with a solvent system consisting of 75% CH_3_CN-25% H_2_O (0.1% TFA). The flow rate was 1 mL/min. Compounds were dissolved in CH_3_CN or a mixture of CH_3_CN–H_2_O, and HPLC chromatograms were recorded in two different wavelengths, 254 and 280 nm, in order to assess their purity. HPLC chromatogram of compound **19** (BRF110), which was tested in vivo, was recorded in three different wavelengths, 214, 254, and 280 nm. All compounds used for biological evaluation had a purity of ≥95% according to HPLC–UV analysis at wavelengths 214, 254, and 280 nm.

^1^H NMR spectra were recorded on a 250 or 400 MHz Bruker Avance FT-NMR spectrometer. ^13^C NMR spectra were recorded at 62.9 MHz. Distortionless enhancement by polarization transfer [DEPT (135)] spectra were recorded at 62.9 MHz. ^13^C NMR and [DEPT (135)] data are combined and represented as follows: chemical shift and carbon type obtained from [DEPT (135)] experiments. Chemical shifts are reported in ppm relative to the solvent signal. Multiplicity is indicated as follows: s (singlet); d (doublet); t (triplet); q (quartet); m (multiplet); br (broad); dd (doublet of doublets), ddd (doublet of doublets of doublets). Electron spray ionization (ESI) mass spectra were recorded on an Agilent 1100 Series LC/MSD instrument. High-resolution mass spectra were obtained under electron spray ionization conditions on a Thermo Scientific LC–MS/LTQ-Orbitrap mass spectrometer.

#### General Conditions for the Synthesis of Pyrimidinols **3a**–**g** (Scheme 1) and **3h** (Scheme 2)

β-Ketoester **2** or **1** (1 equiv) was added to a solution of NaOEt (21% w/w solution in EtOH, 1.1 equiv) and benzamidine (1 equiv) in EtOH. The reaction mixture was stirred overnight under reflux and then concentrated under reduced pressure. The resulting residue was treated with 1 N HCl and then extracted with CH_2_Cl_2_. The combined organic layer was washed with 1 N HCl, brine, dried over MgSO_4_, filtered, and concentrated under reduced pressure to give the crude product. Purification by recrystallization afforded the title compound.

#### 5-Allyl-6-methyl-2-phenylpyrimidin-4-ol (**3a**)

It resulted from β-ketoester **2a** and benzamidine hydrochloride hydrate. It was isolated in a 78% yield as a white solid by recrystallization in ethanol. ^1^H NMR (250 MHz, CDCl_3_): δ 13.67 (br s, 1H), 8.32 (dd, 2H, J = 7.2, 2.3 Hz), 7.50 (m, 3H), 5.95 (m, 1H), 5.17 (d, 1H, J = 18.0, 1.5 Hz), 5.08 (d, 1H, *J* = 11.5, 1.3 Hz), 3.38 (d, 2H, *J* = 6.1 Hz), 2.43 (s, 3H). ^13^C NMR (62.9 MHz, CDCl_3_): δ 165.2 (C), 162.5 (C), 153.8 (C), 134.5 (CH), 132.3 (CH), 131.6 (C), 128.8 (CH), 127.8 (CH), 120.3 (C), 115.6 (CH_2_), 29.8 (CH_2_), 21.9 (CH_3_). HRMS (ESI-LTQ) m/z for C_14_H_15_N_2_O [M + H^+^]: calcd, 227.1184; found, 227.1166.

#### 5-Allyl-2-(4-chlorophenyl)-6-methylpyrimidin-4-ol (**3b**)

It resulted from β-ketoester **2a** and 4-chlorobenzamidine hydroiodide. It was isolated in a 62% yield as a white solid by recrystallization in ethanol. ^1^H NMR (250 MHz, CDCl_3_): δ 13.28 (br s, 1H), 8.23 (d, 2H, *J* = 8.4 Hz), 7.47 (d, 2H, *J* = 8.4 Hz), 5.92 (m, 1H), 5.12 (m, 2H), 3.37 (d, 2H, *J* = 5.8 Hz), 2.42 (s, 3H). ^13^C NMR (62.9 MHz, CDCl_3_): δ 164.9 (C), 162.7 (C), 152.5 (C), 138.0 (C), 134.2 (CH), 134.1 (C), 129.2 (CH), 129.1 (CH), 120.6 (C), 115.7 (CH_2_), 29.7 (CH_2_), 21.9 (CH_3_). HRMS (ESI-LTQ) *m*/*z* for C_14_H_14_ClN_2_O [M + H^+^]: calcd, 261.0795; found, 261.0773.

#### 6-Methyl-2-phenyl-5-propylpyrimidin-4-ol (**3c**)

It resulted from β-ketoester **2b** and benzamidine hydrochloride hydrate. It was isolated in a 45% yield as a white solid by recrystallization in ethanol. ^1^H NMR (250 MHz, CDCl_3_): δ 13.46 (br s, 1H), 8.29 (dd, 2H, *J* = 7.6, 2.1 Hz), 7.50 (m, 3H), 2.59 (m, 2H), 2.44 (s, 3H), 1.62 (m, 2H), 1.04 (t, 3H, *J* = 7.3 Hz). ^13^C NMR (62.9 MHz, CDCl_3_): δ 165.4 (C), 161.5 (C), 153.3 (C), 132.4 (C), 131.5 (CH), 128.8 (CH), 127.7 (CH), 123.2 (C), 28.0 (CH_2_), 21.9 (CH_3_), 21.7 (CH_2_), 14.5 (CH_3_). HRMS (ESI-LTQ) *m*/*z* for C_14_H_17_N_2_O [M + H^+^]: calcd, 229.1341; found, 229.1320.

#### 2-(4-Chlorophenyl)-6-methyl-5-propylpyrimidin-4-ol (**3d**)

It resulted from β-ketoester **2b** and 4-chlorobenzamidine hydroiodide. It was isolated in a 59% yield as a white solid by recrystallization in ethanol. ^1^H NMR (250 MHz, CDCl_3_): δ 13.41 (br s, 1H), 8.25 (d, 2H, *J* = 8.7 Hz), 7.46 (d, 2H, *J* = 8.7 Hz), 2.58 (t, 2H, *J* = 7.8 Hz), 2.43 (s, 3H), 1.59 (m, 2H), 1.04 (t, 3H, *J* = 7.3 Hz). ^13^C NMR (62.9 MHz, CDCl_3_): δ 165.3 (C), 161.6 (C), 152.2 (C), 137.9 (C), 130.8 (C), 129.15 (CH), 129.13 (CH), 123.5 (C), 27.9 (CH_2_), 21.9 (CH_3_), 21.7 (CH_2_), 14.5 (CH_3_). HRMS (ESI-LTQ) *m*/*z* for C_14_H_16_ClN_2_O [M + H^+^]: calcd, 263.0951; found, 263.0936.

#### 5-Isopropyl-6-methyl-2-phenylpyrimidin-4-ol (**3e**)

It resulted from β-ketoester **2c** and benzamidine hydrochloride hydrate. It was isolated in a 29% yield as a white solid by recrystallization in ethanol. ^1^H NMR (250 MHz, CDCl_3_): δ 13.34 (br s, 1H), 8.30 (d, 2H, *J* = 5.7 Hz), 7.50 (m, 3H), 3.13 (m, 1H), 2.47 (s, 3H), 1.41 (d, 6H, *J* = 6.8 Hz). ^13^C NMR (62.9 MHz, CDCl_3_): δ 164.7 (C), 160.7 (C), 153.4 (C), 132.4 (C), 131.5 (CH), 128.8 (CH), 127.7 (CH), 127.2 (C), 28.2 (C), 22.5 (CH_3_), 19.7 (CH_3_). HRMS (ESI-LTQ) *m*/*z* for C_14_H_17_N_2_O [M + H^+^]: calcd, 229.1341; found, 229.1331.

#### 5-Ethyl-6-methyl-2-phenylpyrimidin-4-ol (**3f**)

It resulted from β-ketoester **2d** and benzamidine hydrochloride hydrate. It was isolated in an 85% yield as a white solid by recrystallization in ethanol. ^1^H NMR (250 MHz, CDCl_3_): δ 12.86 (br s, 1H), 8.23 (dd, 2H, *J* = 7.0, 1.8 Hz), 7.51 (m, 3H, *J* = 5.5, 1.8 Hz), 2.63 (q, 2H, *J* = 7.4 Hz), 2.44 (s, 3H), 1.18 (t, 3H, *J* = 7.5 Hz). ^13^C NMR (62.9 MHz, CDCl_3_): δ 164.8 (C), 161.0 (C), 153.2 (C), 132.3 (C), 131.7 (CH), 129.0 (CH), 127.6 (CH), 124.7 (C), 21.6 (CH_2_), 19.2 (CH_3_), 12.7 (CH_3_). HRMS (ESI-LTQ) *m*/*z* for C_13_H_15_N_2_O [M + H^+^]: calcd, 215.1184; found, 215.1176.

#### 2-(4-Chlorophenyl)-5-ethyl-6-methylpyrimidin-4-ol (**3g**)

It resulted from β-ketoester **2d** and 4-chlorobenzamidine hydroiodide. It was isolated in a 33% yield as a white solid by recrystallization in ethanol. ^1^H NMR (250 MHz, DMSO-*d*_6_): δ 12.51 (br s, 1H), 8.10 (d, 2H, *J* = 8.6 Hz), 7.57 (d, 2H, *J* = 8.6 Hz), 2.44 (q, 2H, *J* = 7.4 Hz), 2.31 (s, 3H), 1.03 (t, 3H, *J* = 7.4 Hz). HRMS (ESI-LTQ) *m*/*z* for C_13_H_14_ClN_2_O [M + H^+^]: calcd, 249.0795; found, 249.0780.

#### 6-Methyl-2-phenylpyrimidin-4-ol (**3h**)

It resulted from β-ketoester **1** and benzamidine hydrochloride hydrate. It was isolated in a 30% yield as a white solid by recrystallization in ethanol. ^1^H NMR (250 MHz, CDCl_3_): δ 13.19 (br s, 1H), 8.20 (dd, 2H, *J* = 7.2, 2.4 Hz), 7.53 (m, 3H), 6.30 (s, 1H), 2.40 (s, 3H). ^13^C NMR (62.9 MHz, CDCl_3_): δ 166.5 (C), 165.5 (C), 156.7 (C), 132.3 (C), 132.0 (CH), 129.1 (CH), 128.0 (CH), 111.0 (CH), 24.4 (CH_3_). HRMS (ESI-LTQ) *m*/*z* for C_11_H_11_N_2_O [M + H^+^]: calcd, 187.0871; found, 187.0862.

#### General Conditions for the Synthesis of Chloropyrimidines **4a**–**g** (Scheme 1) and **4h** (Scheme 2)

Pyrimidinol **3** (1 equiv) was dissolved in neat POCl_3_, and the resulting solution was stirred for 6 h under reflux. The solvent was evaporated under reduced pressure, and the resulting residue was dissolved in EtOAc and then washed with saturated Na_2_CO_3_ and brine. The organic layer was dried over MgSO_4_, filtered, and concentrated under reduced pressure to give the crude product. Purification by flash chromatography on silica gel with Hex/EtOAc (10:1) afforded the title compound.

#### 5-Allyl-4-chloro-6-methyl-2-phenylpyrimidine (**4a**)

It was prepared by the chlorination of **3a** and was isolated in a 59% yield as a beige solid. ^1^H NMR (250 MHz, CDCl_3_): δ 8.44 (dd, 2H, *J* = 6.7, 3.0 Hz), 7.48 (m, 3H), 5.89 (m, 1H), 5.13 (dd, 1H, *J* = 10.2, 1.4 Hz), 5.02 (dd, 1H, *J* = 17.1, 1.5 Hz), 3.52 (d, 2H, *J* = 5.8 Hz), 2.57 (s, 3H). ^13^C NMR (62.9 MHz, CDCl_3_): δ 167.9 (C), 162.3 (C), 161.4 (C), 136.4 (C), 132.5 (CH), 130.9 (CH), 128.5 (CH), 128.3 (CH), 126.8 (C), 116.8 (CH_2_), 33.0 (CH_2_), 22.6 (CH_3_). HRMS (ESI-LTQ) *m*/*z* for C_14_H_14_ClN_2_ [M + H^+^]: calcd, 245.0846; found, 245.0840.

#### 5-Allyl-4-chloro-2-(4-chlorophenyl)-6-methylpyrimidine (**4b**)

It was prepared by the chlorination of **3b** and was isolated in a 66% yield as a beige solid. ^1^H NMR (250 MHz, CDCl_3_): δ 8.35 (d, 2H, *J* = 8.7 Hz), 7.41 (d, 2H, *J* = 8.7 Hz), 5.88 (m, 1H), 5.13 (dd, 1H, *J* = 10.2, 1.4 Hz), 5.01 (dd, 1H, *J* = 17.1, 1.5 Hz), 3.52 (dd, 2H, *J* = 5.8, 1.6 Hz), 2.57 (s, 3H). ^13^C NMR (62.9 MHz, CDCl_3_): δ 168.0 (C), 161.6 (C), 161.2 (C), 137.3 (C), 134.8 (C), 132.3 (CH), 129.7 (CH), 128.8 (CH), 127.2 (C), 117.0 (CH_2_), 33.1 (CH_2_), 22.6 (CH_3_). HRMS (ESI-LTQ) *m*/*z* for C_14_H_13_Cl_2_N_2_ [M + H^+^]: calcd, 279.0456; found, 279.0446.

#### 4-Chloro-6-methyl-2-phenyl-5-propylpyrimidine (**4c**)

It was prepared by the chlorination of **3c** and was isolated in a 75% yield as a beige solid. ^1^H NMR (250 MHz, CDCl_3_): δ 8.41(m, 2H), 7.46 (m, 3H), 2.73 (t, 2H, *J* = 7.8 Hz), 2.62 (s, 3H), 1.62 (m, 2H), 1.04 (t, 3H, *J* = 7.3 Hz). ^13^C NMR (62.9 MHz, CDCl_3_): δ 167.2 (C), 161.7 (C), 161.5 (C), 136.2 (C), 131.0 (CH), 129.8 (C), 128.6 (CH), 128.3 (CH), 31.0 (CH_2_), 22.5 (CH_3_), 21.7 (CH_2_), 14.3 (CH_3_). HRMS (ESI-LTQ) *m*/*z* for C_14_H_16_ClN_2_ [M + H^+^]: calcd, 247.1002; found, 247.0985.

#### 4-Chloro-2-(4-chlorophenyl)-6-methyl-5-propylpyrimidine (**4d**)

It was prepared by the chlorination of **3d** and was isolated in a 72% yield as a beige solid. ^1^H NMR (250 MHz, CDCl_3_): δ 8.36 (d, 2H, *J* = 8.6 Hz), 7.42 (d, 2H, *J* = 8.6 Hz), 2.74 (t, 2H, *J* = 8.0 Hz), 2.64 (s, 3H), 1.62 (m, 2H), 1.05 (t, 3H, *J* = 7.3 Hz). ^13^C NMR (62.9 MHz, CDCl_3_): δ 167.1 (C), 161.9 (C), 160.5 (C), 137.5 (C), 134.4 (C), 130.3 (C), 129.8 (CH), 128.9 (CH), 31.1 (CH_2_), 22.4 (CH_2_), 21.7 (CH_3_), 14.3 (CH_3_). HRMS (ESI-LTQ) *m*/*z* for C_14_H_15_Cl_2_N_2_ [M + H^+^]: calcd, 281.0612; found, 281.0603.

#### 4-Chloro-5-isopropyl-6-methyl-2-phenylpyrimidine (**4e**)

It was prepared by the chlorination of **3e** and was isolated in an 81% yield as a beige solid. ^1^H NMR (250 MHz, CDCl_3_): δ 8.41 (m, 2H), 7.45 (m, 3H), 3.57 (m, 1H), 2.66 (s, 3H), 1.41 (d, 6H, *J* = 7.2 Hz). ^13^C NMR (62.9 MHz, CDCl_3_): δ 167.2 (C), 161.5 (C), 160.5 (C), 136.4 (C), 133.9 (C), 130.8 (CH), 128.5 (CH), 128.2 (CH), 28.7 (CH), 24.1 (CH_3_), 19.8 (CH_3_). HRMS (ESI-LTQ) *m*/*z* for C_14_H_16_ClN_2_ [M + H^+^]: calcd, 247.1002; found, 247.0975.

#### 4-Chloro-5-ethyl-6-methyl-2-phenylpyrimidine (**4f**)

It was prepared by the chlorination of **3f** and was isolated in a 62% yield as a beige solid. ^1^H NMR (250 MHz, CDCl_3_): δ 8.41 (m, 2H), 7.50 (m, 3H), 2.83 (q, 2H, *J* = 7.5 Hz), 2.71 (s, 3H), 1.23 (t, 3H, *J* = 7.5 Hz). ^13^C NMR (62.9 MHz, CDCl_3_): δ 166.1 (C), 162.4 (C), 160.9 (C), 134.6 (C), 131.7 (CH), 128.7 (CH), 128.5 (CH), 22.4 (CH_2_), 21.4 (CH_3_), 12.3 (CH_3_). HRMS (ESI-LTQ) *m*/*z* for C_13_H_14_ClN_2_ [M + H^+^]: calcd, 233.0846; found, 233.0839.

#### 4-Chloro-2-(4-chlorophenyl)-5-ethyl-6-methylpyrimidine (**4g**)

It was prepared by the chlorination of **3g** and was isolated in a 70% yield as a beige solid. ^1^H NMR (250 MHz, CDCl_3_): δ 8.33 (d, 2H, *J* = 8.6 Hz), 7.40 (d, 2H, *J* = 8.7 Hz), 2.77 (q, 2H, *J* = 7.5 Hz), 2.58 (s, 3H), 1.20 (t, 3H, *J* = 7.5 Hz). ^13^C NMR (62.9 MHz, CDCl_3_): δ 167.1 (C), 161.0 (C), 160.8 (C), 137.0 (C), 135.1 (C), 131.2 (C), 129.6 (CH), 128.7 (CH), 22.5 (CH_2_), 22.4 (CH_3_), 12.5 (CH_3_). HRMS (ESI-LTQ) *m*/*z* for C_13_H_13_Cl_2_N_2_ [M + H^+^]: calcd, 267.0456; found, 267.0439.

#### 4-Chloro-6-methyl-2-phenylpyrimidine (**4h**)

It was prepared by the chlorination of **3h** and was isolated in a 59% yield as a beige solid. ^1^H NMR (250 MHz, CDCl_3_): δ 8.44 (m, 2H), 7.48 (m, 3H), 7.08 (s, 1H), 2.57 (s, 3H). ^13^C NMR (62.9 MHz, CDCl_3_): δ 169.1 (C), 165.1 (C), 161.5 (C), 136.5 (C), 131.4 (CH), 128.7 (CH), 128.6 (CH), 118.5 (CH), 24.2 (CH_3_). HRMS (ESI-LTQ) *m*/*z* for C_11_H_10_ClN_2_ [M + H^+^]: calcd, 205.0533; found, 205.0517.

#### General Conditions for the Synthesis of Aminopyrimidine Esters **5a**–**i** (Scheme 1) and **8a** (Scheme 2)

A solution of chloropyrimidine **4** (1 equiv), ethyl or methyl 4-aminobenzoate (1.8 equiv), and a catalytic amount of concentrated HCl (2–3 drops) in EtOH was stirred overnight under reflux. When TLC indicated the consumption of chloropyrimidine, the solvent was evaporated under reduced pressure and the resulting residue was treated with water, followed by extraction with EtOAc. The combined organic layer was washed with water, dried over MgSO_4_, filtered, and concentrated under reduced pressure to give the crude product. Purification by flash chromatography on silica gel afforded the title compound.

#### Ethyl 4-((5-Allyl-6-methyl-2-phenylpyrimidin-4-yl)amino)benzoate (**5a**)

It was prepared from **4a** and ethyl 4-aminobenzoate. It was isolated in a 45% yield as a white solid by flash chromatography on silica gel with Hex/EtOAc (10:1 to 6:1). ^1^H NMR (250 MHz, CDCl_3_): δ 8.43 (m, 2H), 8.08 (d, 2H, *J* = 8.8 Hz), 7.76 (d, 2H, *J* = 8.8 Hz), 7.49 (m, 3H), 6.91 (s, 1H), 6.01 (m, 1H), 5.32 (d, 1H, *J* = 10.3 Hz), 5.26 (d, 1H, *J* = 17.0 Hz), 4.39 (q, 2H, *J* = 7.1 Hz), 3.49 (d, 2H, *J* = 5.5 Hz), 2.60 (s, 3H), 1.42 (t, 3H, *J* = 7.1 Hz). HRMS (ESI-LTQ) *m*/*z* for C_23_H_24_N_3_O_2_ [M + H^+^]: calcd, 374.1869; found, 374.1852.

#### Ethyl 4-((5-Allyl-2-(4-chlorophenyl)-6-methylpyrimidin-4-yl)amino)benzoate (**5b**)

It was prepared from **4b** and ethyl 4-aminobenzoate. It was isolated in a 49% yield as a white solid by flash chromatography on silica gel with Hex/EtOAc (10:1 to 6:1). ^1^H NMR (250 MHz, CDCl_3_): δ 8.35 (d, 2H, *J* = 8.7 Hz), 8.06 (d, 2H, *J* = 8.7 Hz), 7.70 (d, 2H, *J* = 8.7 Hz), 7.43 (d, 2H, *J* = 8.6 Hz), 6.87 (s, 1H), 5.99 (m, 1H), 5.30 (d, 1H, *J* = 10.3 Hz), 5.23 (d, 1H, *J* = 17.3 Hz), 4.38 (q, 2H, *J* = 7.1 Hz), 3.45 (d, 2H, *J* = 5.4 Hz), 2.54 (s, 3H), 1.41 (t, 3H, *J* = 7.1 Hz). ^13^C NMR (62.9 MHz, CDCl_3_): δ 166.5 (C), 164.5 (C), 160.6 (C), 158.5 (C), 143.7 (C), 136.6 (C), 136.5 (C), 133.5 (CH), 130.8 (CH), 129.5 (CH), 128.8 (CH), 124.7 (C), 119.3 (CH), 117.7 (CH_2_), 111.6 (C), 60.9 (CH_2_), 30.6 (CH_2_), 22.4 (CH_3_), 14.5 (CH_3_). HRMS (ESI-LTQ) *m*/*z* for C_23_H_23_ClN_3_O_2_ [M + H^+^]: calcd, 408.1479; found, 408.1454.

#### Ethyl 4-((6-Methyl-2-phenyl-5-propylpyrimidin-4-yl)amino)benzoate (**5c**)

It was prepared from **4c** and ethyl 4-aminobenzoate. It was isolated in a 56% yield as a white solid by flash chromatography on silica gel with Hex/EtOAc (10:1 to 6:1). ^1^H NMR (250 MHz, CDCl_3_): δ 8.41 (m, 2H), 8.08 (d, 2H, *J* = 8.8 Hz), 7.82 (d, 2H, *J* = 8.8 Hz), 7.47 (m, 3H), 6.73 (s, 1H), 4.39 (q, 2H, *J* = 7.1 Hz), 2.63 (t, 2H, *J* = 7.4 Hz), 2.55 (s, 3H), 1.66 (m, 2H), 1.42 (t, 3H, *J* = 7.1 Hz), 1.09 (t, 3H, *J* = 7.3 Hz). ^13^C NMR (62.9 MHz, CDCl_3_): δ 166.5 (C), 164.0 (C), 160.8 (C), 157.8 (C), 143.9 (C), 138.2 (C), 130.8 (CH), 130.3 (CH), 128.5 (CH), 128.1 (CH), 124.5 (C), 119.4 (CH), 114.7 (C), 60.9 (CH_2_), 28.2 (CH_2_), 22.4 (CH_3_), 21.5 (CH_2_), 14.5 (CH_3_), 14.4 (CH_3_). HRMS (ESI-LTQ) *m*/*z* for C_23_H_26_N_3_O_2_ [M + H^+^]: calcd, 376.2025; found, 376.1997.

#### Ethyl 4-((2-(4-Chlorophenyl)-6-methyl-5-propylpyrimidin-4-yl)amino)benzoate (**5d**)

It was prepared from **4d** and ethyl 4-aminobenzoate. It was isolated in a 56% yield as a white solid by flash chromatography on silica gel with Hex/EtOAc (10:1 to 6:1). ^1^H NMR (250 MHz, CDCl_3_): δ 8.34 (d, 2H, *J* = 8.7 Hz), 8.08 (d, 2H, *J* = 8.8 Hz), 7.77 (d, 2H, *J* = 8.8 Hz), 7.43 (d, 2H, *J* = 8.7 Hz), 6.72 (s, 1H), 4.39 (q, 2H, *J* = 7.1 Hz), 2.63 (t, 2H, *J* = 7.5 Hz), 2.55 (s, 3H), 1.67 (m, 2H), 1.42 (t, 3H, *J* = 7.1 Hz), 1.10 (t, 3H, *J* = 7.3 Hz). ^13^C NMR (62.9 MHz, CDCl_3_): δ 166.5 (C), 164.1 (C), 159.9 (C), 157.9 (C), 143.7 (C), 136.7 (C), 136.4 (C), 130.8 (CH), 129.4 (CH), 128.7 (CH), 124.7 (C), 119.5 (CH), 114.9 (C), 60.9 (CH_2_), 28.2 (CH_2_), 22.4 (CH_3_), 21.5 (CH_2_), 14.6 (CH_3_), 14.4 (CH_3_). HRMS (ESI-LTQ) *m*/*z* for C_23_H_25_ClN_3_O_2_ [M + H^+^]: calcd, 410.1635; found, 410.1611.

#### Ethyl 4-((5-Isopropyl-6-methyl-2-phenylpyrimidin-4-yl)amino)benzoate (**5e**)

It was prepared from **4e** and ethyl 4-aminobenzoate. It was isolated in a 51% yield as a white solid by flash chromatography on silica gel with Hex/EtOAc (10:1 to 6:1). ^1^H NMR (250 MHz, CDCl_3_): δ 8.39 (dd, 2H, *J* = 7.3, 2.0 Hz), 8.09 (d, 2H, *J* = 8.7 Hz), 7.80 (d, 2H, *J* = 8.8 Hz), 7.46 (m, 3H), 6.82 (s, 1H), 4.39 (q, 2H, *J* = 7.1 Hz), 3.46 (m, 1H), 2.60 (s, 3H), 1.47 (d, 6H, *J* = 7.3 Hz), 1.42 (t, 3H, *J* = 7.3 Hz). ^13^C NMR (62.9 MHz, CDCl_3_): δ 166.5 (C), 163.6 (C), 160.6 (C), 157.7 (C), 143.8 (C), 138.0 (C), 130.9 (CH), 130.3 (CH), 128.6 (CH), 128.0 (CH), 124.5 (C), 119.4 (CH), 60.9 (CH_2_), 26.3 (CH), 23.5 (CH_3_), 20.6 (CH_3_), 14.6 (CH_3_). HRMS (ESI-LTQ) *m*/*z* for C_23_H_26_N_3_O_2_ [M + H^+^]: calcd, 376.2025; found, 376.1990.

#### Ethyl 4-((5-Ethyl-6-methyl-2-phenylpyrimidin-4-yl)amino)benzoate (**5f**)

It was prepared from **4f** and ethyl 4-aminobenzoate. It was isolated in a 43% yield as a white solid by flash chromatography on silica gel with Hex/EtOAc (10:1 to 6:1). ^1^H NMR (250 MHz, CDCl_3_): δ 9.96 (s, 1H), 8.39 (d, 2H, *J* = 7.6 Hz), 7.95 (d, 2H, *J* = 8.4 Hz), 7.81 (d, 2H, *J* = 8.5 Hz), 7.53–7.37 (m, 3H), 4.33 (q, 2H, *J* = 7.1 Hz), 2.99 (d, 2H, *J* = 6.8 Hz), 2.83 (s, 3H), 1.36 (t, 3H, *J* = 7.1 Hz), 1.22 (t, 3H, *J* = 6.3 Hz). ^13^C NMR (62.9 MHz, CDCl_3_): δ 166.1 (C), 159.5 (C), 156.8 (C), 153.9 (C), 141.1 (C), 133.5 (C), 130.0 (CH), 129.9 (CH), 129.6 (CH), 129.0 (CH), 127.7 (C), 124.2 (CH), 124.0 (C), 61.2 (CH_2_), 18.8 (CH_3_), 16.8 (CH_2_), 14.5 (CH_3_), 12.3 (CH_3_). HRMS (ESI-LTQ) *m*/*z* for C_22_H_24_N_3_O_2_ [M + H^+^]: calcd, 362.1869; found, 362.1843.

#### Ethyl 4-((2-(4-Chlorophenyl)-5-ethyl-6-methylpyrimidin-4-yl)amino)benzoate (**5g**)

It was prepared from **4g** and ethyl 4-aminobenzoate. It was isolated in a 57% yield as a white solid by flash chromatography on silica gel with Hex/EtOAc (10:1 to 6:1). ^1^H NMR (250 MHz, CDCl_3_): δ 8.34 (d, 2H, *J* = 8.7 Hz), 8.09 (d, 2H, *J* = 8.8 Hz), 7.78 (d, 2H, *J* = 8.8 Hz), 7.43 (d, 2H, *J* = 8.6 Hz), 6.74 (s, 1H), 4.39 (q, 2H, *J* = 7.1 Hz), 2.69 (q, 2H, *J* = 7.6 Hz), 2.57 (s, 3H), 1.42 (t, 3H, *J* = 7.1 Hz), 1.29 (t, 3H, *J* = 7.6 Hz). ^13^C NMR (62.9 MHz, CDCl_3_): δ 166.4 (C), 157.7 (C), 143.6 (C), 136.5 (C), 130.9 (CH), 129.5 (CH), 128.7 (CH), 119.6 (CH), 116.2 (C), 61.0 (CH_2_), 29.8 (CH_3_), 19.4 (CH_2_), 14.6 (CH_3_), 12.4 (CH_3_). HRMS (ESI-LTQ) *m*/*z* for C_22_H_23_ClN_3_O_2_ [M + H^+^]: calcd, 396.1479; found, 396.1461.

#### Ethyl/Methyl 4-((5-Allyl-6-methyl-2-phenylpyrimidin-4-yl)amino)-2-fluorobenzoate (**5h**)

It was prepared from **4a** and methyl 4-amino-2-fluorobenzoate. It was isolated as a mixture (60/40) of the ethyl and methyl ester, respectively, in a 54% yield as a beige solid by flash chromatography on silica gel with Hex/EtOAc (10:1 to 6:1). ^1^H NMR (250 MHz, CDCl_3_): δ 8.41 (m, 2H), 7.95 (t, 1H, *J* = 8.6 Hz), 7.86 (dd, 1H, *J* = 13.6, 1.9 Hz), 7.48 (m, 3H), 7.26 (m, 1H), 6.93 (s, 1H), 5.98 (m, 1H), 5.30 (d, 1H, *J* = 10.0 Hz), 5.22 (d, 1H, *J* = 17.5 Hz), 4.39 (q, 1.2H, *J* = 7.1 Hz, for the ethyl ester), 3.93 (s, 1.1H, for the methyl ester), 3.46 (d, 2H, *J* = 5.5 Hz), 2.56 (s, 3H), 1.41 (t, 2H, *J* = 7.1 Hz, for the ethyl ester). HRMS (ESI-LTQ) *m*/*z* for C_23_H_23_FN_3_O_2_ [M + H^+^]: calcd, 392.1774; found, 392.1758 (for the ethyl ester).

#### Ethyl/Methyl 4-((5-Allyl-6-methyl-2-phenylpyrimidin-4-yl)amino)-3-methylbenzoate (**5i**)

It was prepared from **4a** and methyl 4-amino-3-methylbenzoate. It was isolated as a mixture (75/25) of the ethyl and methyl ester, respectively, in a 36% yield as a beige solid by flash chromatography on silica gel with Hex/EtOAc (10:1 to 6:1). ^1^H NMR (250 MHz, CDCl_3_): δ 8.42 (m, 3H), 8.00 (d, 1H, *J* = 8.4 Hz), 7.90 (s, 1H), 7.44 (m, 3H), 6.63 (s, 1H), 5.99 (m, 1H), 5.28 (d, 1H, *J* = 10.3 Hz), 5.18 (d, 1H, *J* = 17.5 Hz), 4.38 (q, 1.6H, *J* = 6.9 Hz, for the ethyl ester), 3.92 (s, 0.75H, for the methyl ester), 3.47 (s, 2H), 2.55 (s, 3H), 2.29 (s, 3H), 1.42 (t, 2.3H, *J* = 7.0 Hz, for the ethyl ester). HRMS (ESI-LTQ) *m*/*z* for C_24_H_26_N_3_O_2_ [M + H^+^]: calcd, 388.2025; found, 388.2006 (for the ethyl ester).

#### Ethyl 4-((6-Methyl-2-phenylpyrimidin-4-yl)amino)benzoate (**8a**)

It was prepared from **4h** and ethyl 4-aminobenzoate. It was isolated in a 20% yield as a beige solid by flash chromatography on silica gel with Hex/EtOAc (10:1 to 6:1). ^1^H NMR (250 MHz, CDCl_3_): δ 8.41 (dd, 2H, *J* = 6.7, 3.0 Hz), 8.06 (d, 2H, *J* = 8.7 Hz), 7.60 (d, 2H, *J* = 8.7 Hz), 7.47 (m, 3H), 7.31 (s, 1H), 6.48 (s, 1H), 4.38 (q, 2H, *J* = 7.1 Hz), 2.45 (s, 3H), 1.40 (t, 3H, *J* = 7.1 Hz). ^13^C NMR (62.9 MHz, CDCl_3_): δ 166.9 (C), 166.5 (C), 164.3 (C), 160.4 (C), 143.6 (C), 138.1 (C), 131.0 (CH), 130.6 (CH), 128.5 (CH), 128.3 (CH), 124.7 (C), 119.3 (CH), 102.9 (CH), 60.9 (CH_2_), 24.4 (CH_3_), 14.5 (CH_3_). HRMS (ESI-LTQ) *m*/*z* for C_20_H_20_N_3_O_2_ [M + H^+^]: calcd, 334.1556; found, 334.1542.

#### General Conditions for the Synthesis of *N*-Alkylated Aminopyrimidines **6a**–**e** (Scheme 1) and **8b**–**c** (Scheme 2)

A solution of aminopyrimidine **5** or **8a** (1 equiv) in DMF was treated with Cs_2_CO_3_ (1.2 equiv) and an alkyl halide (1.5 equiv). The reaction mixture was stirred at room temperature overnight, and then it was diluted with water, followed by extraction with EtOAc. The combined organic layer was washed with water, dried over MgSO_4_, filtered, and concentrated under reduced pressure to give the crude product. Purification by flash chromatography on silica gel afforded the title compound.

#### Ethyl 4-((5-Allyl-6-methyl-2-phenylpyrimidin-4-yl)(methyl)amino)benzoate (**6a**)

It resulted from the alkylation of **5a** with iodomethane and was isolated in a 56% yield as a white solid by flash chromatography on silica gel with Hex/EtOAc (10:1 to 6:1). ^1^H NMR (250 MHz, CDCl_3_): δ 8.44 (m, 2H), 7.94 (d, 2H, *J* = 8.8 Hz), 7.46 (m, 3H), 6.87 (d, 2H, *J* = 8.8 Hz), 5.61 (m, 1H), 4.96 (dd, 1H, *J* = 10.2, 1.5 Hz), 4.79 (dd, 1H, *J* = 17.1, 1.5 Hz), 4.35 (q, 2H, *J* = 7.1 Hz), 3.54 (s, 3H), 3.10 (d, 2H, *J* = 5.8 Hz), 2.57 (s, 3H), 1.38 (t, 3H, *J* = 7.1 Hz). ^13^C NMR (62.9 MHz, CDCl_3_): δ 168.1 (C), 166.5 (C), 163.7 (C), 162.4 (C), 151.7 (C), 137.6 (C), 134.2 (CH), 131.2 (CH), 130.5 (CH), 128.6 (CH), 128.1 (CH), 123.3 (C), 121.6 (C), 118.0 (CH), 116.2 (CH_2_), 60.8 (CH_2_), 39.9 (CH_3_), 31.7 (CH_2_), 22.8 (CH_3_), 14.5 (CH_3_). HRMS (ESI-LTQ) *m*/*z* for C_24_H_26_N_3_O_2_ [M + H^+^]: calcd, 388.2025; found, 388.2001.

#### Ethyl 4-((5-Allyl-2-(4-chlorophenyl)-6-methylpyrimidin-4-yl)(methyl)amino)benzoate (**6b**)

It resulted from the alkylation of **5b** with iodomethane and was isolated in a 66% yield as a beige solid by flash chromatography on silica gel with Hex/EtOAc (10:1 to 6:1). ^1^H NMR (250 MHz, CDCl_3_): δ 8.39 (d, 2H, *J* = 8.6 Hz), 7.94 (d, 2H, *J* = 8.8 Hz), 7.42 (d, 2H, *J* = 8.6 Hz), 6.87 (d, 2H, *J* = 8.8 Hz), 5.59 (m, 1H), 4.96 (dd, 1H, *J* = 10.1, 1.3 Hz), 4.78 (dd, 1H, *J* = 17.1, 1.4 Hz), 4.35 (q, 2H, *J* = 7.1 Hz), 3.52 (s, 3H), 3.08 (d, 2H, *J* = 5.8 Hz), 2.55 (s, 3H), 1.38 (t, 3H, *J* = 7.1 Hz).

#### Ethyl 4-((2-(4-Chlorophenyl)-5-ethyl-6-methylpyrimidin-4-yl)(methyl)amino)benzoate (**6c**)

It resulted from the alkylation of **5g** with iodomethane and was isolated in a 76% yield as a white solid by flash chromatography on silica gel with EtOAc/Hex (10:1 to 6:1). ^1^H NMR (250 MHz, CDCl_3_): δ 8.42 (d, 2H, *J* = 7.5 Hz), 7.97 (d, 2H, *J* = 8.6 Hz), 7.44 (d, 2H, *J* = 8.6 Hz), 6.92 (d, 2H, *J* = 6.9 Hz), 4.36 (q, 2H, *J* = 7.1 Hz), 3.57 (s, 3H), 2.63 (s, 3H), 2.29 (m, 2H), 1.38 (t, 3H, *J* = 7.1 Hz), 0.90 (t, 3H, *J* = 7.2 Hz).

#### Ethyl/Methyl 4-((5-Allyl-6-methyl-2-phenylpyrimidin-4-yl)(methyl)amino)-2-fluorobenzoate (**6d**)

It resulted from the alkylation of **5h** with iodomethane and was isolated as a mixture (60/40) of the ethyl and methyl ester, respectively, in an 81% yield as a beige solid by flash chromatography on silica gel with EtOAc/Hex (10:1 to 6:1). ^1^H NMR (250 MHz, CDCl_3_): δ 8.43 (m, 2H), 7.82 (t, 1H, *J* = 8.5 Hz), 7.46 (m, 3H), 6.53 (d, 1H, *J* = 8.8 Hz), 6.48 (d, 1H, *J* = 13.5 Hz), 5.67 (m, 1H), 5.01 (d, 1H, *J* = 9.9 Hz), 4.83 (d, 1H, *J* = 17.1 Hz), 4.35 (q, 1.2H, *J* = 7.1 Hz, for the ethyl ester), 3.89 (s, 1.2H, for the methyl ester), 3.47 (s, 3H), 3.20 (d, 2H, *J* = 5.6 Hz), 2.61 (s, 3H), 1.37 (t, 2H, *J* = 7.1 Hz, for the ethyl ester). HRMS (ESI-LTQ) *m*/*z* for C_24_H_25_FN_3_O_2_ [M + H^+^]: calcd, 406.1931; found, 406.1910 (for the ethyl ester).

#### Ethyl/Methyl 4-((5-Allyl-6-methyl-2-phenylpyrimidin-4-yl)(methyl)amino)-3-methylbenzoate (**6e**)

It resulted from the alkylation of **5i** with iodomethane and was isolated as a mixture (75/25) of the ethyl and methyl ester, respectively, in an 83% yield as a beige solid by flash chromatography on silica gel with EtOAc/Hex (10:1 to 6:1). ^1^H NMR (250 MHz, CDCl_3_): δ 8.49 (m, 2H), 7.97 (s, 1H), 7.80 (d, 1H, *J* = 8.2 Hz), 7.47 (m, 3H), 7.00 (d, 1H, *J* = 8.2 Hz), 5.28 (m, 1H), 4.82 (d, 1H, *J* = 10.2 Hz), 4.68 (d, 1H, *J* = 17.1 Hz), 4.38 (q, 1.5H, *J* = 7.1 Hz, for the ethyl ester), 3.92 (s, 0.75H, for the methyl ester), 3.40 (s, 3H), 2.80 (d, 2H, *J* = 5.2 Hz), 2.44 (s, 3H), 2.32 (s, 3H), 1.41 (t, 2.3H, *J* = 7.1 Hz, for the ethyl ester). HRMS (ESI-LTQ) *m*/*z* for C_25_H_28_N_3_O_2_ [M + H^+^]: calcd, 402.2182; found, 402.2165 (for the ethyl ester).

#### Ethyl 4-(Allyl(6-methyl-2-phenylpyrimidin-4-yl)amino)benzoate (**8b**)

It resulted from the alkylation of **8a** with allyl bromide and was isolated in an 83% yield as a white solid by flash chromatography on silica gel with EtOAc/Hex (10:1 to 6:1). ^1^H NMR (250 MHz, CDCl_3_): δ 8.41 (dd, 2H, *J* = 6.6, 2.9 Hz), 8.12 (d, 2H, *J* = 8.5 Hz), 7.45 (m, 3H), 7.38 (d, 2H, *J* = 8.5 Hz), 6.20 (s, 1H), 6.05 (m, 1H), 5.19 (d, 1H, *J* = 18.0 Hz), 5.17 (d, 1H, *J* = 9.3 Hz), 4.74 (d, 2H, *J* = 5.6 Hz), 4.41 (q, 2H, *J* = 7.1 Hz), 2.36 (s, 3H), 1.42 (t, 3H, *J* = 7.1 Hz). ^13^C NMR (62.9 MHz, CDCl_3_): δ 166.1 (C), 165.8 (C), 163.7 (C), 162.1 (C), 148.0 (C), 138.4 (C), 133.7 (CH), 131.3 (CH), 130.4 (CH), 128.5 (C), 128.4 (CH), 128.3 (CH), 126.7 (CH), 117.5 (CH_2_), 102.2 (CH), 61.3 (CH_2_), 52.7 (CH_2_), 24.4 (CH_3_), 14.5 (CH_3_). HRMS (ESI-LTQ) *m*/*z* for C_23_H_24_N_3_O_2_ [M + H^+^]: calcd, 374.1869; found, 374.1856.

#### Ethyl 4-((6-Methyl-2-phenylpyrimidin-4-yl)(propyl)amino)benzoate (**8c**)

It resulted from the alkylation of **8a** with *n*-propyl bromide and was isolated in a 63% yield as a white solid by flash chromatography on silica gel with EtOAc/Hex (10:1 to 6:1). ^1^H NMR (250 MHz, CDCl_3_): δ 8.41 (dd, 2H, *J* = 6.5, 2.8 Hz), 8.14 (d, 2H, *J* = 8.4 Hz), 7.45 (m, 3H), 7.35 (d, 2H, *J* = 8.4 Hz), 6.09 (s, 1H), 4.41 (q, 2H, *J* = 7.1 Hz), 4.08 (t, 2H, *J* = 7.5 Hz), 2.33 (s, 3H), 1.75 (m, 2H), 1.42 (t, 3H, *J* = 7.1 Hz), 0.98 (t, 3H, *J* = 7.4 Hz). ^13^C NMR (62.9 MHz, CDCl_3_): δ 166.1 (C), 165.4 (C), 163.7 (C), 162.5 (C), 148.2 (C), 138.6 (C), 131.4 (CH), 130.3 (CH), 128.6 (C), 128.4 (CH), 128.3 (CH), 127.2 (CH), 101.9 (CH), 61.3 (CH_2_), 51.8 (CH_2_), 24.4 (CH_3_), 21.4 (CH_2_), 14.5 (CH_3_), 11.6 (CH_3_). HRMS (ESI-LTQ) *m*/*z* for C_23_H_26_N_3_O_2_ [M + H^+^]: calcd, 376.2025; found, 376.2013.

#### General Conditions for the Synthesis of Aminopyrimidine Acid Analogues **7a**–**l** (Scheme 1) and **9a**–**c** (Scheme 2)

They were prepared from the aqueous hydrolysis of the corresponding aminopyrimidine esters **5a**–**i**, **6a**–**e**, and **8a**–**c**. A solution of the ester (1 equiv) in EtOH was treated with 1 N LiOH (2.2 equiv), and the resulting mixture in EtOH-1 N LiOH (4:1) was stirred at room temperature overnight. The solvent was then evaporated under reduced pressure, and the resulting residue was treated with 1 N NaOH to pH ∼ 9–10 and then extracted with Et_2_O. The remaining aqueous layer was acidified with 1 N HCl to pH ∼ 2–3 and then extracted with EtOAc. The combined organic layer was washed with 0.1 N HCl, dried over MgSO_4_, filtered, and concentrated under reduced pressure to afford the title compound in high purity. The compound could be further purified, when necessary, by reverse-phase preparative HPLC with a CH_3_CN–H_2_O (0.1% TFA) solution (75:25 unless indicated otherwise) as the eluent (λ = 254 nm, flow rate = 21 mL/min).

#### 4-((5-Allyl-6-methyl-2-phenylpyrimidin-4-yl)amino)benzoic Acid (**7a**)

It resulted from the hydrolysis of **5a** and was isolated in a 90% yield as a white solid. ^1^H NMR (250 MHz, DMSO-*d*_6_): δ 8.68 (s, 1H), 8.29 (m, 2H), 7.93 (d, 2H, *J* = 9.1 Hz), 7.88 (d, 2H, *J* = 9.1 Hz), 7.46 (m, 3H), 5.92 (m, 1H), 5.02 (m, 2H), 3.56 (d, 2H, *J* = 5.0 Hz), 2.44 (s, 3H). HRMS (ESI-LTQ) *m*/*z* for C_21_H_20_N_3_O_2_ [M + H^+^]: calcd, 346.1550; found, 346.1545.

#### 4-((5-Allyl-2-(4-chlorophenyl)-6-methylpyrimidin-4-yl)amino)benzoic Acid (**7b**)

It resulted from the hydrolysis of **5b** and was isolated in a 69% yield as a beige solid. ^1^H NMR (250 MHz, CD_3_OD): δ 8.29 (d, 2H, *J* = 8.7 Hz), 8.01 (d, 2H, *J* = 8.9 Hz), 7.83 (d, 2H, *J* = 8.9 Hz), 7.45 (d, 2H, *J* = 8.7 Hz), 6.01 (m, 1H), 5.14 (dd, 1H, *J* = 10.3, 1.5 Hz), 5.05 (dd, 1H, *J* = 17.3, 1.8 Hz), 3.55 (d, 2H, *J* = 5.4 Hz), 2.49 (s, 3H). ^13^C NMR (62.9 MHz, CD_3_OD): δ 165.8 (C), 161.4 (C), 160.0 (C), 145.8 (C), 138.2 (C), 137.3 (C), 134.9 (C), 131.5 (CH), 130.5 (CH), 129.5 (CH), 121.5 (CH), 116.2 (CH_2_), 114.0 (C), 30.2 (CH_2_), 21.9 (CH_3_). HRMS (ESI-LTQ) *m*/*z* for C_21_H_19_ClN_3_O_2_ [M + H^+^]: calcd, 380.1166; found, 380.1141.

#### 4-((6-Methyl-2-phenyl-5-propylpyrimidin-4-yl)amino)benzoic Acid (**7c**)

It resulted from the hydrolysis of **5c** and was isolated in a 62% yield as a beige solid. ^1^H NMR (250 MHz, CD_3_OD): δ 8.20 (m, 2H), 8.03 (d, 2H, *J* = 8.8 Hz), 7.83 (d, 2H, *J* = 8.8 Hz), 7.48 (m, 3H), 2.76 (t, 2H, *J* = 7.9 Hz), 2.55 (s, 3H), 1.63 (m, 2H), 1.08 (t, 3H, *J* = 7.3 Hz). HRMS (ESI-LTQ) *m*/*z* for C_21_H_22_N_3_O_2_ [M + H^+^]: calcd, 348.1707; found, 348.1700.

#### 4-((2-(4-Chlorophenyl)-6-methyl-5-propylpyrimidin-4-yl)amino)benzoic Acid (**7d**)

It resulted from the hydrolysis of **5d** and was isolated in a 61% yield as a white solid. ^1^H NMR (250 MHz, CD_3_OD): δ 8.12 (d, 2H, *J* = 8.6 Hz), 8.08 (d, 2H, *J* = 8.6 Hz), 7.73 (d, 2H, *J* = 8.6 Hz), 7.60 (d, 2H, *J* = 8.6 Hz), 2.83 (t, 2H, *J* = 7.8 Hz), 2.66 (s, 3H), 1.70 (m, 2H), 1.13 (t, 3H, *J* = 7.3 Hz). ^13^C NMR (62.9 MHz, CD_3_OD): δ 167.9 (C), 160.5 (C), 156.6 (C), 155.2 (C), 141.7 (C), 139.1 (C), 130.4 (C), 130.2 (CH), 129.8 (CH), 129.2 (CH), 123.9 (CH), 116.6 (C), 26.2 (CH_2_), 20.7 (CH_3_), 16.4 (CH_2_), 12.8 (CH_3_). HRMS (ESI-LTQ) *m*/*z* for C_21_H_21_ClN_3_O_2_ [M + H^+^]: calcd, 382.1317; found, 382.1313.

#### 4-((5-Isopropyl-6-methyl-2-phenylpyrimidin-4-yl)amino)benzoic Acid (**7e**)

It resulted from the hydrolysis of **5e** and was isolated in an 81% yield as a beige solid. ^1^H NMR (250 MHz, CD_3_OD): δ 8.26 (m, 2H), 8.02 (d, 2H, *J* = 8.7 Hz), 7.81 (d, 2H, *J* = 8.8 Hz), 7.44 (m, 3H), 3.50 (m, 1H), 2.59 (s, 3H), 1.46 (d, 6H, *J* = 7.3 Hz). ^13^C NMR (62.9 MHz, CD_3_OD): δ 170.0 (C), 164.6 (C), 161.6 (C), 159.6 (C), 145.9 (C), 139.1 (C), 131.5 (CH), 131.3 (CH), 129.4 (CH), 129.0 (CH), 125.9 (C), 121.8 (CH), 121.7 (C), 26.8 (CH), 23.4 (CH_3_), 20.5 (CH_3_). HRMS (ESI-LTQ) *m*/*z* for C_21_H_22_N_3_O_2_ [M + H^+^]: calcd, 348.1707; found, 348.1697.

#### 4-((5-Ethyl-6-methyl-2-phenylpyrimidin-4-yl)amino)benzoic Acid (**7f**)

It resulted from the hydrolysis of **5f** and was isolated in a 73% yield as a beige solid. ^1^H NMR (250 MHz, CD_3_OD): δ 8.27 (m, 2H), 8.03 (d, 2H, *J* = 8.8 Hz), 7.90 (d, 2H, *J* = 8.8 Hz), 7.46 (m, 3H), 2.82 (q, 2H, *J* = 7.5 Hz), 2.55 (s, 3H), 1.24 (t, 3H, *J* = 7.5 Hz). ^13^C NMR (62.9 MHz, CD_3_OD): δ 164.1 (C), 161.8 (C), 161.7 (C), 159.7 (C), 145.9 (C), 139.2 (C), 131.4 (CH), 131.3 (CH), 129.4 (CH), 129.0 (CH), 126.0 (C), 121.8 (CH), 118.4 (C), 21.3 (CH_3_), 19.5 (CH_2_), 12.8 (CH_3_). HRMS (ESI-LTQ) *m*/*z* for C_20_H_20_N_3_O_2_ [M + H^+^]: calcd, 334.1550 found, 334.1544.

#### 4-((2-(4-Chlorophenyl)-5-ethyl-6-methylpyrimidin-4-yl)amino)benzoic Acid (**7g**)

It resulted from the hydrolysis of **5g** and was isolated in a 65% yield as a white solid. ^1^H NMR (250 MHz, CD_3_OD): δ 8.28 (d, 2H, *J* = 8.7 Hz), 8.03 (d, 2H, *J* = 8.8 Hz), 7.86 (d, 2H, *J* = 8.8 Hz), 7.46 (d, 2H, *J* = 8.7 Hz), 2.82 (q, 2H, *J* = 7.5 Hz), 2.54 (s, 3H), 1.23 (t, 3H, *J* = 7.3 Hz). HRMS (ESI-LTQ) *m*/*z* for C_20_H_19_ClN_3_O_2_ [M + H^+^]: calcd, 368.1160; found, 368.1151.

#### 4-((5-Allyl-6-methyl-2-phenylpyrimidin-4-yl)(methyl)amino)benzoic Acid (**7h**)

It resulted from the hydrolysis of **6a** and was isolated in a 63% yield as a white solid. ^1^H NMR (250 MHz, CD_3_OD): δ 8.39 (m, 2H), 7.97 (d, 2H, *J* = 8.8 Hz), 7.48 (m, 3H), 6.97 (d, 2H, *J* = 8.7 Hz), 5.63 (m, 1H), 4.89 (m, 2H), 3.54 (s, 3H), 3.13 (d, 2H, *J* = 5.8 Hz), 2.54 (s, 3H). HRMS (ESI-LTQ) *m*/*z* for C_22_H_22_N_3_O_2_ [M + H^+^]: calcd, 360.1707; found, 360.1695.

#### 4-((5-Allyl-2-(4-chlorophenyl)-6-methylpyrimidin-4-yl)(methyl)amino)benzoic Acid (**7i**)

It resulted from the hydrolysis of **6b** and was isolated in a 69% yield as a white solid. ^1^H NMR (250 MHz, CD_3_OD): δ 8.30 (d, 2H, *J* = 8.5 Hz), 8.07 (d, 2H, *J* = 8.7 Hz), 7.62 (d, 2H, *J* = 8.7 Hz), 7.30 (d, 2H, *J* = 8.7 Hz), 5.51 (m, 1H), 5.01 (d, 1H, *J* = 10.3 Hz), 4.98 (d, 1H, *J* = 17.3 Hz), 3.69 (s, 3H), 2.98 (d, 2H, *J* = 5.5 Hz), 2.56 (s, 3H).

#### 4-((2-(4-Chlorophenyl)-5-ethyl-6-methylpyrimidin-4-yl)(methyl)amino)benzoic Acid (**7j**)

It resulted from the hydrolysis of **6c** and was isolated in a 57% yield as a white solid by HPLC purification. ^1^H NMR (250 MHz, CD_3_OD): δ 8.30 (d, 2H, *J* = 8.7 Hz), 8.07 (d, 2H, *J* = 8.7 Hz), 7.60 (d, 2H, *J* = 8.6 Hz), 7.29 (d, 2H, *J* = 8.3 Hz), 3.68 (s, 3H), 2.60 (s, 3H), 2.19 (q, 2H, *J* = 7.4 Hz), 0.87 (t, 3H, *J* = 7.5 Hz).

#### 4-((5-Allyl-6-methyl-2-phenylpyrimidin-4-yl)(methyl)amino)-2-fluorobenzoic Acid (**7k**)

It resulted from the hydrolysis of **6d** and was isolated in a 72% yield as a beige solid by HPLC purification. ^1^H NMR (250 MHz, CDCl_3_): δ 8.42 (m, 2H), 7.88 (t, 1H, *J* = 8.6 Hz), 7.47 (m, 3H), 6.58–6.43 (m, 2H), 5.71 (m, 1H), 5.04 (d, 1H, *J* = 9.0 Hz), 4.86 (d, 1H, *J* = 17.1 Hz), 3.47 (s, 3H), 3.24 (d, 2H, *J* = 5.7 Hz), 2.64 (s, 3H). HRMS (ESI-LTQ) *m*/*z* for C_22_H_21_FN_3_O_2_ [M + H^+^]: calcd, 378.1612; found, 378.1600.

#### 4-((5-Allyl-6-methyl-2-phenylpyrimidin-4-yl)(methyl)amino)-3-methylbenzoic Acid (**7l**)

It resulted from the hydrolysis of **6e** and was isolated in a 74% yield as a white solid by HPLC purification. ^1^H NMR (250 MHz, CDCl_3_): δ 9.78 (s, 1H), 8.38 (d, 2H, *J* = 7.3 Hz), 8.07 (s, 1H), 7.97 (d, 1H, *J* = 9.2 Hz), 7.60 (m, 3H), 7.22 (d, 1H, *J* = 8.0 Hz), 5.26 (m, 1H), 4.96 (d, 1H, *J* = 10.3 Hz), 4.70 (d, 1H, *J* = 17.3 Hz), 3.62 (s, 3H), 2.72 (s, 2H), 2.63 (s, 3H), 2.30 (s, 3H). ^13^C NMR (62.9 MHz, CDCl_3_): δ 169.1 (C), 162.8 (C), 158.8 (C), 157.1 (C), 147.7 (C), 135.3 (C), 133.9 (CH), 133.6 (CH), 131.6 (CH), 130.6 (C), 130.5 (C), 129.8 (CH), 129.3 (CH), 129.2 (CH), 127.6 (CH), 117.2 (CH_2_), 113.6 (C), 42.5 (CH_3_), 30.1 (CH_2_), 18.2 (CH_3_), 17.4 (CH_3_). HRMS (ESI-LTQ) *m*/*z* for C_23_H_24_N_3_O_2_ [M + H^+^]: calcd, 374.1863; found, 374.1862.

#### 4-((6-Methyl-2-phenylpyrimidin-4-yl)amino)benzoic Acid (**9a**)

It resulted from the hydrolysis of **8a** and was isolated in a 47% yield as a white solid by HPLC purification. ^1^H NMR (250 MHz, CD_3_OD): δ 8.26 (dd, 2H, *J* = 7.9, 1.7 Hz), 8.09 (d, 2H, *J* = 8.8 Hz), 7.87 (d, 2H, *J* = 8.8 Hz), 7.63 (m, 3H), 6.78 (s, 1H), 2.57 (s, 3H).

#### 4-(Allyl(6-methyl-2-phenylpyrimidin-4-yl)amino)benzoic Acid (**9b**)

It resulted from the hydrolysis of **8b** and was isolated in a 69% yield as a white solid by HPLC purification. ^1^H NMR (250 MHz, CD_3_OD): δ 8.29 (m, 2H), 8.13 (d, 2H, *J* = 8.6 Hz), 7.46 (m, 5H), 6.30 (s, 1H), 6.07 (m, 1H), 5.18 (m, 2H), 4.76 (d, 2H, *J* = 5.5 Hz), 2.34 (s, 3H). HRMS (ESI-LTQ) *m*/*z* for C_21_H_20_N_3_O_2_ [M + H^+^]: calcd, 346.1550; found, 346.1550.

#### 4-((6-Methyl-2-phenylpyrimidin-4-yl)(propyl)amino)benzoic Acid (**9c**)

It resulted from the hydrolysis of **8c** and was isolated in a 78% yield as a white solid by HPLC purification. ^1^H NMR (250 MHz, CD_3_OD): δ 8.30 (m, 2H), 8.16 (d, 2H, *J* = 8.5 Hz), 7.46 (m, 5H), 6.19 (s, 1H), 4.12 (t, 2H, *J* = 7.5 Hz), 2.31 (s, 3H), 1.77 (m, 2H), 1.00 (t, 3H, *J* = 7.4 Hz). HRMS (ESI-LTQ) *m*/*z* for C_21_H_22_N_3_O_2_ [M + H^+^]: calcd, 348.1707; found, 348.1704.

#### General Conditions for the Synthesis of phenoxypyrimidine esters **10a**–**g** (Scheme 3)

A solution of chloropyrimidine **4** (1 equiv) and ethyl 4-hydroxybenzoate (2 equiv) in DMF was treated with Cs_2_CO_3_ (2 equiv). The reaction mixture was stirred at room temperature overnight, and then it was diluted with water, followed by extraction with EtOAc. The combined organic layer was washed with water, dried over MgSO_4_, filtered, and concentrated under reduced pressure to give the crude product. Purification by flash chromatography on silica gel afforded the title compound.

#### Ethyl 4-((5-Allyl-6-methyl-2-phenylpyrimidin-4-yl)oxy)benzoate (**10a**)

It resulted from **4a** and ethyl 4-hydroxybenzoate. It was isolated as an inseparable mixture (∼1:4) of the title compound and its propenyl isomer **10a-iso**, respectively, in a 29% yield as a white solid by flash chromatography on silica gel with EtOAc/Hex (10:1 to 6:1). ^1^H NMR (250 MHz, CDCl_3_): δ 8.16 (m, 4H), 7.37 (m, 3H), 7.28 (m, 2H), 6.50 (s, 1.4H, for **10a-iso**), 5.98 (m, 0.2H, for **10a**), 5.09 (m, 0.4H, for **10a**), 4.42 (q, 2H, *J* = 7.1 Hz), 3.54 (d, 0.4H, *J* = 5.9 Hz, for **10a**), 2.66 (s, 2.4H, for **10a-iso**), 2.60 (s, 0.6H, for **10a**), 2.00 (d, 2.3H, *J* = 4.7 Hz, for **10a-iso**), 1.43 (t, 3H, *J* = 7.1 Hz). HRMS (ESI-LTQ) *m*/*z* for C_23_H_23_N_2_O_3_ [M + H^+^]: calcd, 375.1709; found, 375.1700.

#### Ethyl 4-((5-Allyl-2-(4-chlorophenyl)-6-methylpyrimidin-4-yl)oxy)benzoate (**10b**)

It resulted from **4b** and ethyl 4-hydroxybenzoate. It was isolated as an inseparable mixture (∼1:4) of the title compound and its propenyl isomer **10b-iso**, respectively, in a 77% yield as a white solid by flash chromatography on silica gel with EtOAc/Hex (10:1 to 6:1). ^1^H NMR (250 MHz, CDCl_3_): δ 8.12 (m, 4H), 7.29 (m, 4H), 6.49 (s, 1.4H, for **10b-iso**), 5.98 (m, 0.15H, for **10b**), 5.11 (m, 0.30H, for **10b**), 4.42 (q, 2H, *J* = 7.1 Hz), 3.54 (d, 0.3H, *J* = 5.9 Hz, for **10b**), 2.65 (s, 2.2H, for **10b-iso**), 2.58 (s, 0.4H, for **10b**), 1.99 (d, 2.2H, *J* = 4.7 Hz, for **10b-iso**), 1.43 (t, 3H, *J* = 7.1 Hz). HRMS (ESI-LTQ) *m*/*z* for C_23_H_22_ClN_2_O_3_ [M + H^+^]: calcd, 409.1319; found, 409.1314.

#### Ethyl 4-((6-Methyl-2-phenyl-5-propylpyrimidin-4-yl)oxy)benzoate (**10c**)

It resulted from **4c** and ethyl 4-hydroxybenzoate. It was isolated in a 56% yield as a white solid by flash chromatography on silica gel with EtOAc/Hex (10:1 to 6:1). ^1^H NMR (250 MHz, CDCl_3_): δ 8.19 (m, 2H), 8.15 (d, 2H, *J* = 8.5 Hz), 7.36 (m, 3H), 7.28 (d, 2H, *J* = 8.7 Hz), 4.42 (q, 2H, *J* = 7.1 Hz), 2.76 (t, 2H, *J* = 7.5 Hz), 2.61 (s, 3H), 1.68 (m, 2H), 1.43 (t, 3H, *J* = 7.1 Hz), 1.05 (t, 3H, *J* = 7.4 Hz). ^13^C NMR (62.9 MHz, CDCl_3_): δ 167.2 (C), 167.0 (C), 166.3 (C), 160.8 (C), 157.2 (C), 137.3 (C), 131.2 (CH), 130.5 (CH), 128.5 (CH), 127.9 (CH), 127.1 (C), 121.6 (CH), 118.4 (C), 61.2 (CH_2_), 27.4 (CH_2_), 22.3 (CH_2_), 22.0 (CH_3_), 14.5 (CH_3_), 14.3 (CH_3_). HRMS (ESI-LTQ) *m*/*z* for C_23_H_25_N_2_O_3_ [M + H^+^]: calcd, 377.1865; found, 377.1877.

#### Ethyl 4-((5-Isopropyl-6-methyl-2-phenylpyrimidin-4-yl)oxy)benzoate (**10d**)

It resulted from **4e** and ethyl 4-hydroxybenzoate. It was isolated in a 47% yield as a beige solid by flash chromatography on silica gel with EtOAc/Hex (10:1 to 6:1). ^1^H NMR (250 MHz, CDCl_3_): δ 8.15 (m, 4H), 7.36 (m, 3H), 7.26 (d, 2H, *J* = 8.0 Hz), 4.42 (q, 2H, *J* = 7.1 Hz), 3.36 (m, 1H), 2.65 (s, 3H), 1.44 (d, 6H, *J* = 7.0 Hz), 1.43 (t, 3H, *J* = 7.1 Hz). ^13^C NMR (62.9 MHz, CDCl_3_): δ 167.0 (C), 166.4 (C), 166.3 (C), 160.6 (C), 157.2 (C), 137.2 (C), 131.2 (CH), 130.4 (CH), 128.5 (CH), 127.9 (CH), 127.0 (C), 123.4 (C), 121.6 (CH), 61.2 (CH_2_), 27.2 (CH), 23.0 (CH_3_), 20.5 (CH_3_), 14.5 (CH_3_). HRMS (ESI-LTQ) *m*/*z* for C_23_H_25_N_2_O_3_ [M + H^+^]: calcd, 377.1865; found, 377.1841.

#### Ethyl 4-((5-Ethyl-6-methyl-2-phenylpyrimidin-4-yl)oxy)benzoate (**10e**)

It resulted from **4f** and ethyl 4-hydroxybenzoate. It was isolated in a 62% yield as a white solid by flash chromatography on silica gel with EtOAc/Hex (10:1 to 6:1). ^1^H NMR (250 MHz, CDCl_3_): δ 8.17 (m, 2H), 8.12 (d, 2H, *J* = 8.7 Hz), 7.37 (m, 3H), 7.28 (d, 2H, *J* = 8.7 Hz), 4.41 (q, 2H, *J* = 7.1 Hz), 2.81 (q, 2H, *J* = 7.5 Hz), 2.61 (s, 3H), 1.43 (t, 3H, *J* = 7.1 Hz), 1.28 (t, 3H, *J* = 7.5 Hz). ^13^C NMR (62.9 MHz, CDCl_3_): δ 166.8 (C), 166.4 (C), 160.8 (C), 157.3 (C), 137.4 (C), 131.2 (CH), 130.4 (CH), 128.5 (CH), 127.9 (CH), 127.1 (C), 121.6 (CH), 119.9 (C), 61.2 (CH_2_), 21.8 (CH_3_), 18.9 (CH_2_), 14.5 (CH_3_), 13.4 (CH_3_). HRMS (ESI-LTQ) *m*/*z* for C_22_H_23_N_2_O_3_ [M + H^+^]: calcd, 363.1709; found, 363.1697.

#### Ethyl 4-((2-(4-Chlorophenyl)-5-ethyl-6-methylpyrimidin-4-yl)oxy)benzoate (**10f**)

It resulted from **4g** and ethyl 4-hydroxybenzoate. It was isolated in a 50% yield as a white solid by flash chromatography on silica gel with EtOAc/Hex (10:1 to 6:1). ^1^H NMR (250 MHz, CDCl_3_): δ 8.12 (m, 4H), 7.28 (m, 4H), 4.42 (q, 2H, *J* = 7.1 Hz), 2.81 (q, 2H, *J* = 7.5 Hz), 2.61 (s, 3H), 1.43 (t, 3H, *J* = 7.1 Hz), 1.26 (t, 3H, *J* = 7.5 Hz). ^13^C NMR (62.9 MHz, CDCl_3_): δ 166.8 (C), 166.3 (C), 159.8 (C), 157.1 (C), 136.6 (C), 135.8 (C), 131.2 (CH), 129.4 (CH), 128.7 (CH), 127.3 (C), 121.6 (CH), 120.2 (C), 61.2 (CH_2_), 21.7 (CH_3_), 18.9 (CH_2_), 14.5 (CH_3_), 13.3 (CH_3_). HRMS (ESI-LTQ) *m*/*z* for C_22_H_22_ClN_2_O_3_ [M + H^+^]: calcd, 397.1319; found, 397.1296.

#### Ethyl 4-((5-Allyl-6-methyl-2-phenylpyrimidin-4-yl)oxy)-2-fluorobenzoate (**10g**)

It resulted from **4a** and ethyl 2-fluoro-4-hydroxybenzoate. It was isolated as an inseparable mixture (∼1:1.5) of the title compound and its propenyl isomer **10g-iso**, respectively, in a 45% yield as a beige solid by flash chromatography on silica gel with EtOAc/Hex (10:1 to 6:1). ^1^H NMR (250 MHz, CDCl_3_): δ 8.20 (m, 2H), 8.04 (t, 1H, *J* = 8.6 Hz), 7.39 (m, 3H), 7.08 (m, 2H), 6.46 (d, 1.2H, *J* = 2.3 Hz, for **10g-iso**), 5.95 (m, 0.35H, for **10g**), 5.11 (d, 0.45H, *J* = 9.8 Hz, for **10g**), 5.16 (d, 0.45H, *J* = 17.0 Hz, for **10g**), 4.43 (q, 2H, *J* = 7.1 Hz), 3.53 (d, 0.7H, *J* = 5.9 Hz, for **10g**), 2.67 (s, 1.8H, for **10g-iso**), 2.60 (s, 1.2H, for **10g**), 1.99 (d, 1.9H, *J* = 2.1 Hz, for **10g-iso**), 1.43 (t, 3H, *J* = 7.1 Hz).

#### General Conditions for the Synthesis of Phenoxypyrimidine Acid Analogues **11a**–**g** (Scheme 3)

They were prepared from the aqueous hydrolysis of the corresponding esters **10a**–**g** according to the conditions described for the synthesis of aminopyrimidine acid analogues **7a**–**l** and **9a**–**c**.

#### 4-((5-Allyl-6-methyl-2-phenylpyrimidin-4-yl)oxy)benzoic Acid (**11a**)

It resulted from the hydrolysis of **10a** and was isolated as an inseparable mixture (∼1:3) of the title compound and its propenyl isomer **11a-iso**, respectively, in a 73% yield as a white solid. ^1^H NMR (250 MHz, CD_3_OD): δ 8.15–8.00 (m, 4H), 7.33 (m, 3H), 7.19 (m, 2H), 6.54 (s, 1.4H, for **11a-iso**), 6.02 (m, 0.2H, for **11a**), 5.14 (d, 0.25H, *J* = 10.3 Hz, for **11a**), 5.06 (d, 0.25H, *J* = 17.3 Hz, for **11a**), 3.57 (d, 0.6H, *J* = 6.2 Hz, for **11a**), 2.62 (s, 2.2 H, for **11a-iso**), 2.56 (s, 0.8H, for **11a**), 1.98 (d, 2.1H, *J* = 4.5 Hz, for **11a-iso**). HRMS (ESI-LTQ) *m*/*z* for C_21_H_19_N_2_O_3_ [M + H^+^]: calcd, 347.1390; found, 347.1388.

#### 4-((5-Allyl-2-(4-chlorophenyl)-6-methylpyrimidin-4-yl)oxy)benzoic Acid (**11b**)

It resulted from the hydrolysis of **10b** and was isolated as an inseparable mixture (∼1:3) of the title compound and its propenyl isomer **11b-iso**, respectively, in a 66% yield as a white solid. ^1^H NMR (250 MHz, CDCl_3_): δ 8.20 (d, 2H, *J* = 8.4 Hz), 8.11 (d, 2H, *J* = 8.1 Hz), 7.32 (m, 4H), 6.50 (s, 1.3H, for **11b-iso**), 5.96 (m, 0.3H, for **11b**), 5.12 (d, 0.35H, *J* = 10.3 Hz, for **11b**), 5.07 (d, 0.35H, *J* = 17.3 Hz, for **11b**), 3.55 (d, 0.6H, *J* = 3.9 Hz, for **11b**), 2.66 (s, 2.2H, for **11b-iso**), 2.59 (s, 0.8H, for **11b**), 2.00 (d, 2.3H, *J* = 4.5 Hz, for **11b-iso**). HRMS (ESI-LTQ) *m*/*z* for C_21_H_18_ClN_2_O_3_ [M + H^+^]: calcd, 381.1000; found, 381.0997.

#### 4-((6-Methyl-2-phenyl-5-propylpyrimidin-4-yl)oxy)benzoic Acid (**11c**)

It resulted from the hydrolysis of **10c** and was isolated in a 67% yield as a white solid. ^1^H NMR (250 MHz, CD_3_OD): δ 8.12 (m, 4H), 7.35 (m, 5H), 2.82 (t, 2H, *J* = 7.8 Hz), 2.61 (s, 3H), 1.73 (m, 2H), 1.08 (t, 3H, *J* = 7.4 Hz). ^13^C NMR (62.9 MHz, CD_3_OD): δ 169.3 (C), 168.5 (C), 162.1 (C), 158.6 (C), 138.4 (C), 132.4 (CH), 131.5 (CH), 129.3 (CH), 128.9 (CH), 128.7 (C), 122.8 (CH), 119.7 (C), 28.1 (CH_2_), 23.2 (CH_2_), 21.7 (CH_3_), 14.5 (CH_3_). HRMS (ESI-LTQ) *m*/*z* for C_21_H_21_N_2_O_3_ [M + H^+^]: calcd, 349.1547; found, 349.1534.

#### 4-((5-Isopropyl-6-methyl-2-phenylpyrimidin-4-yl)oxy)benzoic Acid (**11d**)

It resulted from the hydrolysis of **10d** and was isolated in a 46% yield as a beige solid. ^1^H NMR (250 MHz, CD_3_OD): δ 8.09 (m, 4H), 7.34 (m, 3H), 7.25 (dd, 2H, *J* = 8.6, 1.7 Hz), 3.43 (m, 1H), 2.64 (s, 3H), 1.46 (d, 6H, *J* = 7.0 Hz). ^13^C NMR (62.9 MHz, CD_3_OD): δ 171.3 (C), 168.5 (C), 167.6 (C), 161.9 (C), 157.7 (C), 138.3 (C), 132.2 (CH), 131.4 (CH), 131.3 (C), 129.3 (CH), 128.8 (CH), 124.5 (C), 122.4 (CH), 28.2 (CH), 22.6 (CH_3_), 20.6 (CH_3_). HRMS (ESI-LTQ) *m*/*z* for C_21_H_21_N_2_O_3_ [M + H^+^]: calcd, 349.1547; found, 349.1534.

#### 4-((5-Ethyl-6-methyl-2-phenylpyrimidin-4-yl)oxy)benzoic Acid (**11e**)

It resulted from the hydrolysis of **10e** and was isolated in a 53% yield as a white solid. ^1^H NMR (250 MHz, CD_3_OD): δ 8.12 (m, 4H), 7.34 (m, 5H), 2.86 (q, 2H, *J* = 7.5 Hz), 2.62 (s, 3H), 1.29 (t, 3H, *J* = 7.5 Hz). HRMS (ESI-LTQ) *m*/*z* for C_20_H_19_N_2_O_3_ [M + H^+^]: calcd, 335.1390; found, 335.1391.

#### 4-((2-(4-Chlorophenyl)-5-ethyl-6-methylpyrimidin-4-yl)oxy)benzoic Acid (**11f**)

It resulted from the hydrolysis of **10f** and was isolated in a 48% yield as a white solid. ^1^H NMR (250 MHz, CD_3_OD): δ 8.14 (d, 2H, *J* = 8.8 Hz), 8.10 (d, 2H, *J* = 8.8 Hz), 7.37 (d, 2H, *J* = 8.8 Hz), 7.31 (d, 2H, *J* = 8.8 Hz), 2.86 (q, 2H, *J* = 7.5 Hz), 2.62 (s, 3H), 1.30 (t, 3H, *J* = 7.5 Hz). HRMS (ESI-LTQ) *m*/*z* for C_20_H_18_ClN_2_O_3_ [M + H^+^]: calcd, 369.1000; found, 369.1014.

#### 4-((5-Allyl-6-methyl-2-phenylpyrimidin-4-yl)oxy)-2-fluorobenzoic Acid (**11g**)

It resulted from the hydrolysis of **10g** and was isolated as a mixture (∼1:2) of the title compound and its propenyl isomer **11g-iso**, respectively. Further purification by reverse-phase HPLC with a CH_3_CN–H_2_O (0.1% TFA) solution (90:10) as the eluent, enabled the isolation of acid **11g** as a beige solid (17%). ^1^H NMR (250 MHz, CD_3_OD): δ 8.16 (dd, 2H, *J* = 7.5, 2.1 Hz), 8.06 (t, 1H, *J* = 8.5 Hz), 7.40 (m, 3H), 7.16 (m, 2H), 6.03 (m, 1H), 5.13 (d, 1H, *J* = 10.3 Hz), 5.05 (d, 1H, *J* = 17.3 Hz), 3.58 (d, 2H, *J* = 5.8 Hz), 2.60 (s, 3H). HRMS (ESI-LTQ) *m*/*z* for C_21_H_18_FN_2_O_3_ [M + H^+^]: calcd, 365.1296; found, 365.1286.

#### Experimental Details for the Synthesis of CF_3_-Substituted Pyrimidines **14**–**19** (Scheme 4)

A detailed description for the preparation of compounds that led to target compound **19** (BRF110) is provided below.

#### 5-Allyl-2-phenyl-6-(trifluoromethyl)pyrimidin-4-ol (**14a**)

Ketoester **13** (3.3 g, 14.7 mmol) was added to a solution of NaOEt (5.5 mL of 21% w/w solution in EtOH, 16.7 mmol) and benzamidine hydrochloride hydrate (2.3 g, 14.7 mmol) in EtOH (15 mL). The reaction mixture was stirred overnight under reflux and concentrated under reduced pressure. The resulting residue was treated with 1 N HCl (40 mL) and then extracted with CH_2_Cl_2_ (2 × 30 mL). The combined organic layer was washed with 1 N HCl, brine, dried over MgSO_4_, filtered, and concentrated under reduced pressure to give the crude product. Purification by recrystallization in ethanol afforded the title compound (2.7 g, 66%) as white needle crystals. ^1^H NMR (250 MHz, CDCl_3_): δ 8.27 (d, 2H, *J* = 8.0 Hz), 7.58 (m, 3H), 5.92 (m, 1H), 5.21 (dd, 1H, *J* = 17.0, 1.3 Hz), 5.10 (dd, 1H, *J* = 10.0, 1.5 Hz), 3.51 (m, 2H). ^13^C NMR (62.9 MHz, CDCl_3_): δ 165.3 (C), 155.1 (C), 149.7 (C, q, *J*_C–F_ = 34.3 Hz), 133.5 (CH), 132.8 (CH), 131.2 (C), 129.3 (CH), 127.8 (CH), 124.6 (C), 121.7 (C, d, *J*_C–F_ = 276.9 Hz), 117.3 (CH_2_), 29.3 (CH_2,_ d, *J*_C–F_ = 2.0 Hz). HRMS (ESI-LTQ) *m*/*z* for C_14_H_12_F_3_N_2_O [M + H^+^]: calcd, 281.0896; found, 281.0893.

#### 5-Allyl-2-(4-chlorophenyl)-6-(trifluoromethyl)pyrimidin-4-ol (**14b**)

It resulted from β-ketoester **13** and 4-chlorobenzamidine hydroiodide according to the conditions described for the preparation of **14a**. It was isolated in a 62% yield as a white solid by recrystallization in ethanol. ^1^H NMR (250 MHz, CDCl_3_): δ 8.27 (d, 2H, *J* = 8.8 Hz), 7.52 (d, 2H, *J* = 8.8 Hz), 5.92 (m, 1H), 5.21 (dd, 1H, *J* = 17.0, 1.3 Hz), 5.10 (dd, 1H, *J* = 10.0, 1.5 Hz), 3.51 (dd, 2H, *J* = 6.3, 1.4 Hz). ^13^C NMR (62.9 MHz, CDCl_3_): δ 165.6 (C), 154.0 (C), 149.9 (C, q, *J*_C–F_ = 34.0 Hz), 133.3 (CH), 129.5 (CH), 129.4 (C), 129.2 (CH), 124.7 (C), 121.5 (C, d, *J*_C–F_ = 275.5 Hz), 117.4 (CH_2_), 29.3 (CH_2_). HRMS (ESI-LTQ) *m*/*z* for C_14_H_11_ClF_3_N_2_O [M + H^+^]: calcd, 315.0512; found, 315.0501.

#### 5-Allyl-4-chloro-2-phenyl-6-(trifluoromethyl)pyrimidine (**15a**)

Pyrimidinol **14a** (2.7 g, 9.6 mmol) was dissolved in neat POCl_3_ (10 mL), and the resulting solution was stirred for 6 h under reflux. The solvent was evaporated under reduced pressure, and the resulting residue was dissolved in EtOAc (60 mL) and then washed with saturated Na_2_CO_3_ (2 × 25 mL) and brine (25 mL). The organic layer was dried over MgSO_4_, filtered, and concentrated under reduced pressure to give the crude product. Purification by flash chromatography on silica gel with Hex/EtOAc (10:1) afforded the title compound (2.5 g, 87%) as a yellow oil. ^1^H NMR (250 MHz, CDCl_3_): δ 8.47 (dd, 2H, *J* = 7.3, 1.8 Hz), 7.50 (m, 3H), 5.92 (m, 1H), 5.22 (d, 1H, *J* = 11.5 Hz), 5.15 (d, 1H, *J* = 18.3 Hz), 3.69 (d, 2H, *J* = 6.0 Hz). ^13^C NMR (62.9 MHz, CDCl_3_): δ 164.9 (C), 162.9 (C), 154.8 (C, q, *J*_C–F_ = 34.5 Hz), 135.0 (C), 132.2 (CH), 132.1 (CH), 128.8 (CH), 128.7 (CH), 127.3 (C), 121.1 (C, d, *J*_C–F_ = 277.4 Hz), 118.1 (CH_2_), 32.1 (CH_2_, d, *J*_C–F_ = 2.1 Hz). HRMS (ESI-LTQ) *m*/*z* for C_14_H_11_ClF_3_N_2_ [M + H^+^]: calcd, 299.0557; found, 299.0560.

#### 5-Allyl-4-chloro-2-(4-chlorophenyl)-6-(trifluoromethyl)pyrimidine (**15b**)

It resulted from the chlorination of **14b** according to the conditions described for the preparation of **15a**. It was isolated in a 76% yield as a beige solid. ^1^H NMR (250 MHz, CDCl_3_): δ 8.37 (d, 2H, *J* = 8.7 Hz), 7.44 (d, 2H, *J* = 8.7 Hz), 5.89 (m, 1H), 5.19 (dd, 1H, *J* = 10.3, 1.3 Hz), 5.12 (dd, 1H, *J* = 17.8, 1.8 Hz), 3.68 (dd, 2H, *J* = 6.1, 1.0 Hz). ^13^C NMR (62.9 MHz, CDCl_3_): δ 165.0 (C), 161.9 (C), 154.8 (C, q, *J*_C–F_ = 34.4 Hz), 138.5 (C), 133.4 (CH), 130.0 (CH), 129.1 (CH), 127.6 (C), 121.0 (C, d, *J*_C–F_ = 277.2 Hz), 118.3 (CH_2_), 32.2 (CH_2_, d, *J*_C–F_ = 2.1 Hz). HRMS (ESI-LTQ) *m*/*z* for C_14_H_10_Cl_2_F_3_N_2_ [M + H^+^]: calcd, 333.0173; found, 334.1548.

#### Ethyl 4-((5-Allyl-2-phenyl-6-(trifluoromethyl)pyrimidin-4-yl)amino)benzoate (**16a**)

A solution of chloropyrimidine **15a** (2.5 g, 8.37 mmol), ethyl 4-aminobenzoate (2.5 g, 15.1 mmol) recrystallized from EtOH, and a catalytic amount of concentrated HCl (2–3 drops) in EtOH (15 mL) was stirred under reflux. The reaction progress was monitored by TLC, and catalytic amounts of concentrated HCl were gradually added to the reaction mixture in order to drive the reaction to completion. When TLC indicated the consumption of most of the chloropyrimidine, the solvent was evaporated under reduced pressure. The resulting residue was treated with water (60 mL), followed by extraction with EtOAc (3 × 40 mL). The combined organic layer was washed with water (3 × 25 mL), dried over MgSO_4_, filtered, and concentrated under reduced pressure to give the crude product. Purification by flash chromatography on silica gel with Hex/EtOAc (10:1 to 6:1) afforded the title compound (1.1 g, 31%) as a yellowish solid. ^1^H NMR (250 MHz, CDCl_3_): δ 8.44 (m, 2H), 8.11 (d, 2H, *J* = 8.8 Hz), 7.74 (d, 2H, *J* = 8.8 Hz), 7.50 (m, 3H), 7.23 (s, 1H), 6.0 (m, 1H), 5.42 (dd, 1H, *J* = 10.0, 0.8 Hz), 5.37 (d, 1H, *J* = 16.8 Hz), 4.40 (q, 2H, *J* = 7.1 Hz), 3.60 (d, 2H, *J* = 5.5 Hz), 1.43 (t, 3H, *J* = 7.1 Hz). ^13^C NMR (62.9 MHz, CDCl_3_): δ 166.3 (C), 162.3 (C), 160.3 (C), 152.8 (C, q, *J*_C–F_ = 32.9 Hz), 142.8 (C), 136.8 (C), 133.6 (CH), 131.2 (CH), 130.8 (CH), 128.7 (CH), 128.4 (CH), 125.6 (C), 121.8 (C, d, *J*_C–F_ = 276.9 Hz), 119.8 (CH), 118.9 (CH_2_), 111.9 (C), 61.0 (CH_2_), 30.1 (CH_2_, d, *J*_C–F_ = 2.1 Hz), 14.5 (CH_3_). HRMS (ESI-LTQ) *m*/*z* for C_23_H_21_F_3_N_3_O_2_ [M + H^+^]: calcd, 428.1580; found, 428.1583.

#### Ethyl 4-((5-Allyl-2-(4-chlorophenyl)-6-(trifluoromethyl)pyrimidin-4-yl)amino)benzoate (**16b**)

It was prepared from **15b** and ethyl 4-aminobenzoate according to the conditions described for the preparation of **16a**. It was isolated in a 31% yield as a white solid by flash chromatography on silica gel with Hex/EtOAc (10:1 to 6:1). ^1^H NMR (250 MHz, CDCl_3_): δ 8.37 (d, 2H, *J* = 8.7 Hz), 8.11 (d, 2H, *J* = 8.8 Hz), 7.69 (d, 2H, *J* = 8.8 Hz), 7.45 (d, 2H, *J* = 8.7 Hz), 7.23 (s, 1H), 5.99 (m, 1H), 5.42 (d, 1H, *J* = 10.0 Hz), 5.37 (d, 1H, *J* = 17.3 Hz), 4.40 (q, 2H, *J* = 7.1 Hz), 3.60 (d, 2H, *J* = 5.4 Hz), 1.42 (t, 3H, *J* = 7.1 Hz). ^13^C NMR (62.9 MHz, CDCl_3_): δ 166.2 (C), 161.4 (C), 160.4 (C), 152.9 (C, q, *J*_C–F_ = 30.8 Hz), 142.6 (C), 137.5 (C), 135.4 (C), 133.5 (CH), 130.9 (CH), 129.8 (CH), 129.0 (CH), 125.9 (C), 121.7 (C, d, *J*_C–F_ = 277.0 Hz), 120.0 (CH), 119.0 (CH_2_), 112.2 (C), 61.1 (CH_2_), 30.2 (CH_2_), 14.5 (CH_3_). HRMS (ESI-LTQ) *m*/*z* for C_23_H_20_ClF_3_N_3_O_2_ [M + H^+^]: calcd, 462.1196; found, 462.1448.

#### 4-((5-Allyl-2-phenyl-6-(trifluoromethyl)pyrimidin-4-yl)amino)benzoic Acid (**17a**)

It resulted from the hydrolysis of ester **16a** according to the conditions described for the preparation of aminopyrimidine acid analogues **7a**–**l**. It was purified by reverse-phase HPLC with a CH_3_CN–H_2_O (0.1% TFA) solution (75:25) as the eluent (λ = 254 nm, flow rate = 21 mL/min) and was isolated as an inseparable mixture (∼1:1) of the title compound and its propenyl isomer **17a-iso**, respectively, in a 65% yield as a white solid. ^1^H NMR (250 MHz, CD_3_OD): δ 8.40 (m, 2H), 8.08 (d, 2H, *J* = 8.8 Hz), 8.00–7.85 (m, 2H), 7.50 (m, 3H), 6.30 (m, 1H, for **17a-iso**), 6.01 (m, 0.6H, for **17a**), 5.20 (d, 0.6H, *J* = 9.3 Hz, for **17a**), 5.12 (d, 0.6H, *J* = 17.0 Hz, for **17a**), 3.69 (s, 1.2H, for **17a**), 1.63 (d, 1.2H, *J* = 6.3 Hz, for **17a-iso**). HRMS (ESI-LTQ) *m*/*z* for C_21_H_17_F_3_N_3_O_2_ [M + H^+^]: calcd, 400.1267; found, 400.1274.

#### 4-((5-Allyl-2-(4-chlorophenyl)-6-(trifluoromethyl)pyrimidin-4-yl)amino)benzoic Acid (**17b**)

It resulted from the hydrolysis of ester **16b** according to the conditions described for the preparation of **17a**. It was purified by reverse-phase HPLC and was isolated as an inseparable mixture (∼3:1) of the title compound and its propenyl isomer **17b-iso**, respectively, in a 42% yield as a white solid. ^1^H NMR (250 MHz, CD_3_OD): δ 8.37 (d, 2H, *J* = 8.6 Hz), 8.08 (d, 2H, *J* = 8.8 Hz), 7.84 (d, 2H, *J* = 8.9 Hz), 7.50 (d, 2H, *J* = 8.7 Hz), 6.30 (m, 0.6H, for **17b-iso**), 6.02 (m, 1H, for **17b**), 5.20 (dd, 1H, *J* = 10.5, 1.5 Hz, for **17b**), 5.11 (dd, 1H, *J* = 17.3, 1.3 Hz, for **17b**), 3.67 (s, 2H, for **17b**), 1.63 (d, 0.9H, *J* = 6.3 Hz, for **17b-iso**). HRMS (ESI-LTQ) *m*/*z* for C_21_H_16_ClF_3_N_3_O_2_ [M + H+]: calcd, 434.0883; found, 434.0878.

#### Ethyl 4-((5-Allyl-2-phenyl-6-(trifluoromethyl)pyrimidin-4-yl)(methyl)amino)benzoate (**18**)

A solution of aminopyrimidine **16a** (1.1 g, 2.58 mmol) in DMF (10 mL) was treated with Cs_2_CO_3_ (1.0 g, 3.07 mmol) and CH_3_I (241 μL, 3.87 mmol). The reaction mixture was stirred at room temperature overnight, and then it was diluted with water (40 mL), followed by extraction with EtOAc (2 × 25 mL). The combined organic layer was washed with water (2 × 20 mL), dried over MgSO_4_, filtered, and concentrated under reduced pressure to give the crude product. Purification by flash chromatography on silica gel with Hex/EtOAc (10:1 to 6:1) afforded the title compound (1.0 g, 88%) as a yellow solid. ^1^H NMR (250 MHz, CDCl_3_): δ 8.49 (m, 2H), 8.02 (d, 2H *J* = 8.8 Hz), 7.50 (m, 3H), 7.01 (d, 2H, *J* = 8.8 Hz), 5.56 (m, 1H), 4.94 (dd, 1H, *J* = 10.0, 1.5 Hz), 4.75 (dd, 1H, *J* = 17.0, 1.5 Hz), 4.38 (q, 2H, *J* = 7.1 Hz), 3.65 (s, 3H), 3.05 (d, 2H, *J* = 6.0 Hz), 1.42 (t, 3H, *J* = 7.1 Hz). ^13^C NMR (62.9 MHz, CDCl_3_): δ 166.0 (C), 165.5 (C), 162.3 (C), 154.8 (C, q, *J*_C–F_ = 32.9 Hz), 150.7 (CH), 136.6 (C), 133.9 (CH), 131.4 (CH), 131.3 (CH), 128.7 (CH), 128.4 (CH), 126.1 (C), 121.8 (C, d, *J*_C–F_ = 277.1 Hz), 121.1 (CH), 120.3 (C), 116.6 (CH_2_), 61.1 (CH_2_), 41.1 (CH_3_), 30.1 (CH_2_, d, *J*_C–F_ = 2.3 Hz), 14.5 (CH_3_). HRMS (ESI-LTQ) *m*/*z* for C_24_H_23_F_3_N_3_O_2_ [M + H^+^]: calcd, 442.1737; found, 442.1740.

#### 4-((5-Allyl-2-phenyl-6-(trifluoromethyl)pyrimidin-4-yl)(methyl)amino)benzoic Acid (**19**, BRF110)

A solution of aminopyrimidine **18** (1.0 g, 2.27 mmol) in 20 mL of EtOH was treated with 1 N LiOH (5 mL, 5 mmol), and the reaction mixture was stirred at room temperature overnight. The solvent was then evaporated under reduced pressure, and the resulting residue was treated with 1 N NaOH (50 mL) to pH ∼ 9–10 and then extracted with Et_2_O (2 × 20 mL). The remaining aqueous layer was acidified with 1 N HCl (40 mL) to pH ∼ 2–3 and then extracted with EtOAc (3 × 30 mL). The combined organic layer was washed with 0.1 N HCl (20 mL), dried over MgSO_4_, filtered, and concentrated under reduced pressure to afford the title compound (800 mg, 85%) as an off-white solid in high purity. The title compound can be further recrystallized in ethanol. ^1^H NMR (250 MHz, CDCl_3_): δ 8.47 (m, 2H), 8.05 (d, 2H, *J* = 8.8 Hz), 7.47 (m, 3H), 6.98 (d, 2H, *J* = 8.8 Hz), 5.55 (m, 1H), 4.93 (dd, 1H, *J* = 10.0, 1.0 Hz), 4.72 (dd, *J* = 17.1, 0.9 Hz), 3.62 (s, 3H), 3.08 (d, 2H, *J* = 5.8 Hz). ^13^C NMR (62.9 MHz, CDCl_3_): δ 171.7 (C), 165.6 (C), 162.6 (C), 155.1 (C, q, *J*_C–F_ = 32.9 Hz), 151.5 (C), 136.5 (C), 133.9 (CH), 132.1 (CH), 131.4 (CH), 128.8 (CH), 128.4 (CH), 124.1 (C), 121.8 (C, d, *J*_C–F_ = 277.2 Hz), 121.1 (C), 120.2 (CH), 116.7 (CH_2_), 40.8 (CH_3_), 30.6 (CH_2_, d, *J*_C–F_ = 2.1 Hz). HRMS (ESI-LTQ) *m*/*z* for C_22_H_19_F_3_N_3_O_2_ [M + H^+^]: calcd, 414.1424; found, 414.1418.

### In Vitro Compound Characterization

Naive human SH-SY5Y neuroblastoma cells were cultured in a humidified incubator at 37 °C in an atmosphere with 5% CO_2_. The cells were maintained as an adherent culture with RPMI 1640, 10% fetal bovine serum. Proliferating undifferentiated SH-SY5Y cells are used in the assays. Neuro-2a cells are mouse neuroblasts with neuronal and amoeboid stem cell morphology. Cells were maintained in Eagle’s Minimum Essential Medium, with fetal bovine serum to a final concentration of 10% at a temperature of 37 °C and an atmosphere of 5% CO_2_.

### Transactivation Assay

Transfection experiments were performed in 24-well plates at a 70% confluency with Lipofectamine Plus reagent (1.5 μL lipofectamine reagent/well) according to the manufacturer’s instructions (Invitrogen). Human HEK 293 cells were transfected with 0.10 μg of pLuc Renilla/well, 0.25 μg of reporter DNA (containing ABCA1 proximal promoter)/well, and 0.10 μg of each receptor (pCDNA3-RXRα, pCDNA3–6myc-LXRα)/well. The transfected cells were cultured in high-glucose DMEM containing 10% FBS serum, 100 U of penicillin/mL, and 100 mg of streptomycin/mL for 6 h. The medium containing transfection reagents was removed, and cells were cultured for another 16 h in a fresh medium containing either 0.5 μM Bex, 12.5 μM Bex, 0.5 μM BRF110, 12.5 μM BRF110, or standard cell culture medium (vehicle). Luciferase activity was then measured by using the Dual Glo Luciferase assay system (Promega) and normalized with Renilla.

### RT-qPCR SREBP-1c Expression

HepG2 cells were cultured in 48-well plates (5–10 × 10^3^ per well) in a complete medium containing Dulbecco’s modified Eagle’s medium (DMEM, Sigma-Aldrich, #D6429) supplemented with 10% heat-inactivated fetal bovine serum (FBS, Gibco, #1050064) and penicillin (100 U/mL)/streptomycin (100 μg/mL) (Gibco, #15140122) in a humidified atmosphere at 37 °C with 5% CO_2_. At a confluency of ∼70%, cells were treated with a medium containing 0.5 μM Bex, 12.5 μM Bex, or 0.5 μM XCT, 12.5 μM XCT or 0.5 μM BRF110, 12.5 μM BRF110 for 24 h. Following treatment, total RNA was isolated according to the RNeasy Mini Kit protocol (Qiagen, Hilden, Germany) and cDNA was synthesized using the PrimeScript RT reagent Kit (Takara, RR037A) using oligo-dT and random hexamers. Quantitative PCR was performed in a 7500 Real-Time PCR System (Applied Biosystems, Carlsbad, CA, USA) using 2× SYBR Select Master Mix (Applied Biosystems, #4472918). Gene expression analysis was carried out using the 2^–ΔΔ*CT*^ relative quantification method. Triplicate reactions were performed for each sample, and the average CT was calculated for the quantification analysis. GAPDH was used as an endogenous reference control. Human SREBP-1c (F, 5′-CCATGGATTGCACTTTCGAA-3′; R, 5′-GGCCAGGGAAGTCACTGTCTT-3′). Human GAPDH (F, 5′-TGGGCTACACTGAGCACCAG-3′; R, 5′-GGGTGTCGCTGTTGAAGTCA-3′).

#### Constructs

DNA sequences encoding for the Nurr1 and the Nur77 ligand-binding domain (LBD) were fused to the gal4 DNA binding domain, while the RXRα (LBD) was fused to vp16 as described.^63^

#### Screening Assays

Synthesized compounds were evaluated for activity in cellular assays. Undifferentiated proliferating SH-SY5Y cells were transiently cotransfected with 100 ng of a DR5-tk promoter-driven luciferase reporter construct along with 50 ng of human Nurr1 and 50 ng of human RXRα CMV-driven expression plasmids using Lipofectamine 2000 (Invitrogen). A CMV β-galactosidase plasmid was included to serve as a transfection control. After 24 h, the culture medium was changed. The test compounds were added to the culture medium at final concentrations of 0.5, 2.5, and 12.5 μM, and the incubation continued overnight. XCT0135908 was used as a positive control in the same concentration as the test compounds. The relative luciferase expression was estimated by determining its activity, using the luciferase assay system (Promega) as per the manufacturer’s instructions.

#### Selectivity Assays

SH-SY5Y cells were transiently cotransfected using Lipofectamine 2000 (Invitrogen) for 4–6 h in OptiMEM-I Reduced Serum Medium (Invitrogen) with the following constructs: 20 ng of MH100-tk-luciferace, 20 ng of CMX-VP16-RXRα-LBD, and 20 ng of CMX-GAL4-Nurr1-LBD or CMX-GAL4-Nur77-LBD. Ten ng of CMX-βgal was also cotransfected as a control. Seventeen hours after transfection, BRF110 or XCT was added to the medium at the indicated concentrations and incubation continued for 12 h. Cell lysates were assayed for luciferase activity normalized to galactosidase activity using a luciferase assay system (Promega) as per the manufacturer’s instructions. Each experiment was done at least in triplicate. All values are means ± SEM for at least three independent experiments.

### In Vivo Evaluation

#### Animals

Animals were housed at the Animal Care Facilities of the Academy of Athens (BRFAA) in a pathogen-free room with controlled light–dark cycle (12 h light-12 h dark) and free access to food and water. Animal breeding and handling was performed according to the European Communities Council directive (86/609/EEC) guidelines, and all animal procedures were approved by the Institutional Animal Care and Use Committee of BRFAA.

#### Compounds

BRF110 or bexarotene were kept as powders (4 °C) and were dissolved in the vehicle before administration to mice. The vehicle consisted of a 10:30:60 mixture of ethanol: propylene glycol: saline (buffered to pH = 10 using NaOH). BRF110 (10 mg/kg) or bexarotene (3 mg/kg and 10 mg/kg) or vehicle were injected intraperitoneally (200 μL) every morning for 5 consecutive days. Two hours after the last injections, the mice were euthanized and intracardial blood was collected.

#### Triglyceride Determination

Mouse serum triglyceride concentrations were determined using the commercially available Triglyceride FS kit by DiaSys (Diagnostic Systems GmbH, Holzheim, Germany). The determination is based on a colorimetric enzymatic assay using glycerol-3-phosphate-oxidase. Samples were diluted 3× in 0.9% NaCl (same as used for the standard curve) and were applied in 96-well ELISA trays followed by the kit-contained reagent. After 10 min at room temperature, the plate was read at 492 nm at a Tecan Sunrise plate reader.

#### Oral Bioavailability

BRF110 (5 mg/kg) was administered to young male mice (*n* = 5) by gavage or intraperitoneally (1 mg/kg) (*n* = 3) as a reference. At 1 h, 2, and 4 h after dosing, the mice were euthanized and cardiac blood and the brain were collected and stored at −80 °C until LC–MS/MS analysis. Quantitative analysis of BRF110 in blood and brain samples was performed using a liquid chromatography–tandem mass spectrometry system consisting of a Waters HPLC (Alliance HT 2795), fitted with a Poroshell 120 Phenyl-Hexyl column (2.1 × 50 mm, 2.7 μm, Agilent–USA) and a Micromass Quattro Micro tandem MS system operated in the positive ion mode. The mobile phase consisted of 0.1% formic acid in water (solvent A) and acetonitrile (solvent B) and was delivered at a flow rate of 0.3 mL/min with an injection volume of 50 μL. BRF110 was extracted from the blood and the brain samples using a protein precipitation (PP) method with acetonitrile (HPLC grade, Sigma-Aldrich, Germany). Briefly after PP, the samples were vortexed and centrifuged at 10,000*g* for 15 min, and the supernatant was dried on a Centrivap Cold Trap concentrator (Labconco, USA). Subsequently, the dried extracts were reconstituted in 1 mL of H_2_O and filtered through RC 0.22 μm filters (Phenomenex, Torrance, US). BRF110 was detected using the multiple reaction monitoring (MRM) scan mode (product-ions *m*/*z* 414.44 > 304.14), and all data were processed using the MassLynx v.4.0 software.

#### ChIP-PCR Assay

ChIP and ChIP-PCR experiments were carried out essentially as previously described.^91^ Briefly, SH-SY5Y cells were cultured for various time points and then cross-linked in two steps: (1) disuccinimidyl glutarate (DSG) for 30 min and then (2) 1% methanol-free ultrapure formaldehyde for 10 min at room temperature. After fixation, chromatin was treated to generate 300–600 bp fragments. Chromatin was immunoprecipitated with anti-RXRα (sc-553 D-20, Santa Cruz Biotechnology) polyclonal rabbit antibody or anti-Nurr1 (N1404 Abcam) antibodies at 4 °C overnight, using an EZ-Magna ChIP kit (Millipore, Massachusetts, USA). The eluted DNA was purified (Qiagen, MinElute) and quantified. ChIP-seq libraries were prepared by an Illumina library preparation kit according to the manufacturer’s instructions. Chromatin fragments of 300–600 bp were incubated with nonspecific IgG and used as control. The supernatant containing the chromatin fragments was retained as input for normalization correction. Protein-enriched fragments were identified by RT-PCR.

Primers for BDNF Promoter I (F) 5′-CTCGAGAGCTCGGCTTACACAGG-3′, (R) 5′-CCTTGGCGACTACAGAAGACAAAGC-3′.

Primers for BDNF Promoter II (F) 5′-TGCTTGTCTCTCAGCAGTCTTGC-3′, (R) 5′-AGTTAACCCAGTATACCAACCCGG-3′.

Primers for BDNF Promoter III (F) 5′-AGAATCAGGCGGTGGAGGTGGTGTG-3′, (R) 5′-AACCCTCTAAGCCAGCGCCCGAAAC-3′.

Primers for BDNF Promoter IV (F) 5′-TGTGTTTGCTGGGGCTGGAAGTG-3′, (R) 5′-GTCAAAGTAACCATCAAGGCAGCTGC-3′.

### Computational Methods

Molecular docking calculations of the compounds described herein have been carried out using the high-resolution X-ray crystal structure of the tertiary complex between the LBD of RXRα, the glucocorticoid receptor-interacting protein 1 coregulator peptide, and palmitic acid (PDB ID: 7a77 resolved at 1.5 Å).^75^ For docking calculations at the LBD of Nurr1, we employed the X-ray crystal structure with bound prostaglandin A1 (PDB ID: 5y41 resolved at 2.05 Å).^34^ The target structures were prepared with polar-only hydrogens and Gasteiger charges using AutoDockTools v1.5.7, whereas ligand conformations were obtained using OMEGA v4.2 (OpenEye Scientific, Cadence).^92^ Docking calculations were carried out using AutoDock Vina v1.2.3.^77^ For the LBD of RXRα, the search space was set at 30 × 30 × 25 (Å) and the exhaustiveness level at 16, whereas for the LBD of Nurr1, the search space was 55 × 60 × 55 (Å) and the exhaustiveness level was set at 32. Docked poses were investigated, and figures were rendered using the open-source PyMOL v2.5 (Schrödinger, LLC). Physiochemical properties of the compounds (Table 2) have been obtained using the SwissADME server.^93^

### Statistics

GraphPad Prism4 software was used for statistical analysis. Significance was assessed with the one- or two-way ANOVA test followed by post-hoc Bonferroni’s multiple comparison test unless otherwise indicated. A *P*-value less than 0.05 was considered significant. Error bars represent ± SEM.

## Supporting Information Available

Molecular formula strings containing molecular structures of **5a**–**19** and the associated EC_50_ (μΜ) activity data (CSV)
Experimental conditions for the synthesis of compounds **2a**–**d** and **13**, HPLC traces for purity analysis, ^1^H and ^13^C NMR spectra, and additional results (PDF)
LBD of RXRα and the top-ranked poses of the compounds presented in Table 1 (PDB)

## Supplementary Material

jm4c03046_si_002.pdf

jm4c03046_si_003.pdb
