# Supplementary material for: BRF110, an Orally Active Nurr1-RXRα-Selective Rexinoid, Enhances BDNF Expression without Elevating Triglycerides
Source: J Med Chem. 2025 Feb 13;68(4):4763–86. doi: 10.1021/acs.jmedchem.4c03046 (PMC11874024; doi:10.1021/acs.jmedchem.4c03046)
Supplement: Supplementary file 2 — jm4c03046_si_002.pdf [file jm4c03046_si_002.pdf]

Supporting Information

BRF110, an Orally Active Nurr1-RXRα Selective Rexinoid, Enhances BDNF Expression without Elevating Triglycerides

Xenophon Asvos[†], Mohamed A. El Mubarak[‡], Theodoros Karampelas[§], Theodoros Rampias[§], Constantin Tamvakopoulos[§], Gregory B. Sivolapenko[‡], Athanasios Papakyriakou[Φ], Stavros Topouzis[‡], Demetrios K. Vassilatis[§, #, *] and Demosthenes Fokas[†, #, *]

†Department of Materials Science and Engineering, University of Ioannina, Ioannina 45110, Greece.

‡Department of Pharmacy, University of Patras, Patras 26504, Greece.

ΦInstitute of Biosciences and Applications, National Centre for Scientific Research "Demokritos", 15341 Athens, Greece

§Center for Clinical Research, Experimental Surgery, and Translational Research, Biomedical Research Foundation of the Academy of Athens, Athens 11527, Greece.

***dfokas@uoi.gr, *dvassilatis@bioacademy.gr**

Table of Contents

Supplementary Figures and Tables... S2

General methods and experimental conditions for the synthesis of compounds **2a-d** and **13**....S7

HPLC traces for purity analysis.. S11

^{1}H NMR and ^{13}C spectra of synthesized compounds.. S26

Supplementary Table 1. AutoDock Vina scores (estimated free energy of binding, ΔG^{est}) of compounds **5a–19** docked to the X-ray crystal structure of the LBD of RXRα (PDB ID: 7a77). Experimental EC_{50} values from the transactivation assay (Table 1) are shown for comparison. Highlighted are the docking scores at the top quartile (lime) and EC_{50} values ≤1 μM (green). The Pearson correlation coefficient of the docking scores and MW of the compounds is 0.493.

Compound ID	MW (g/mol)	ΔG^{est} (kcal/mol)	EC_{50} (μM)
**5a**	373.45	-8.5	>100
**6a**	387.48	-8.7	>100
**7a** (**XCT**)	345.40	-9.2	0.3
**7b**	379.84	-8.3	2.0
**7c**	347.42	-9.3	1.0
**7d**	381.86	-7.7	>10
**7e**	347.42	-10.4	1.0
**7f**	333.39	-10.2	5.0
**7g**	367.83	-8.8	2.0
**7h**	359.43	-8.7	>20
**7i**	393.87	-7.8	7.0
**7j**	381.86	-8.8	4.0
**7k**	377.42	-9.3	7.0
**7l**	373.46	-7.4	>100
**9a**	305.34	-10.1	5.0
**9c**	347.42	-9.3	>10
**11a**	346.39	-8.4	>100
**11b**	380.83	-8.0	>100
**11c**	348.40	-8.2	>100
**11d**	348.40	-9.8	1.0
**11e**	334.38	-9.3	>100
**11f**	368.82	-8.1	>10
**11g**	364.38	-8.6	>10
**16a**	427.43	-8.9	>50
**17a**	399.37	-9.3	0.2
**17b**	433.82	-7.9	10
**19** (**BRF110**)	413.40	-9.2	0.9

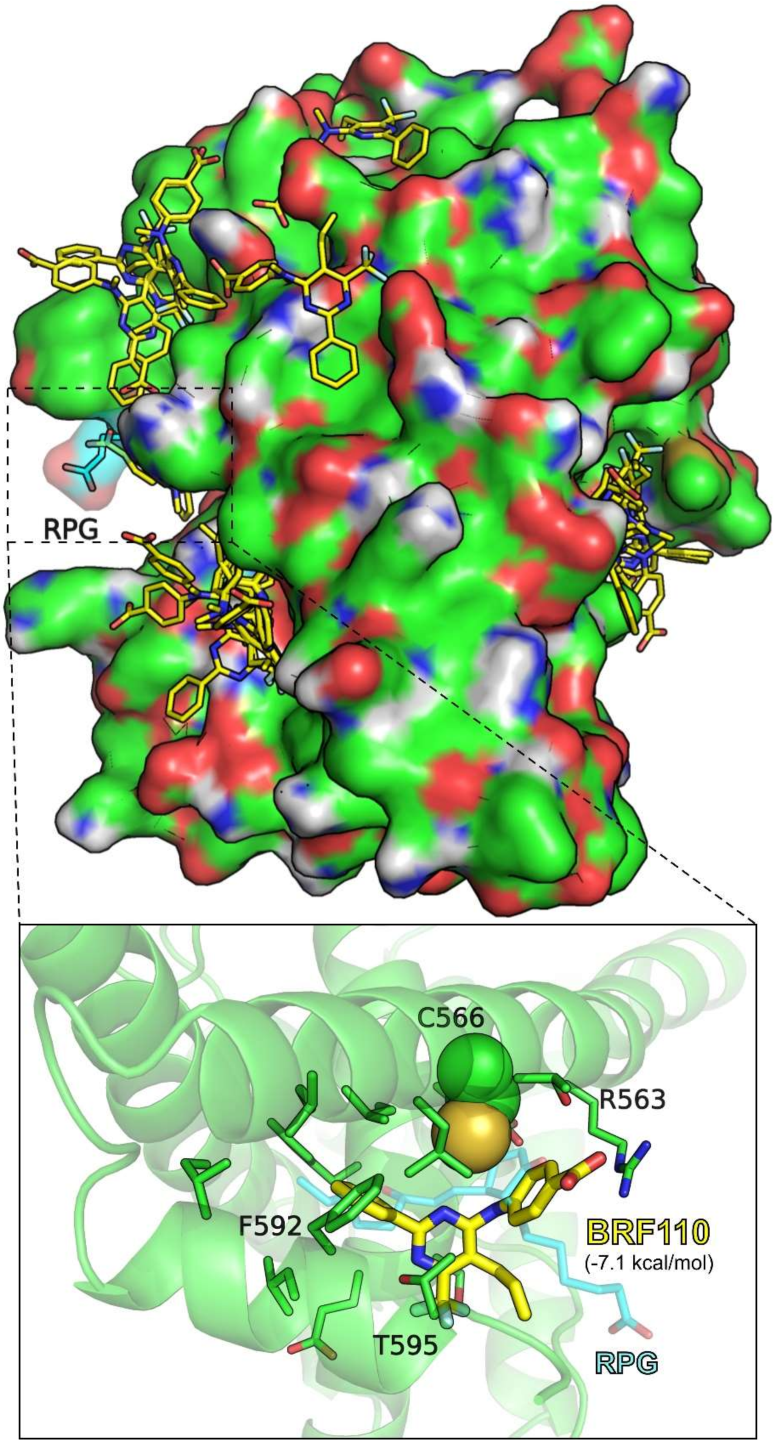

Supplementary Figure S1. The 20 top-ranked docked poses of BRF110 at the LBD of Nurr1, taken from its X-ray structure with bound prostaglandin A1 (RPG) from PDB ID: 5Y41[1]. Inset is a close-up view of the of the BRF110 bound pose at the site where RPG forms the Michael adduct

[1] DOI: https://doi.org/10.1038/s41589-020-0553-6

with Cys566 (highlighted with spheres). The 20 top-ranked poses of BRF110 displayed scores of -6.5 up to -7.2 kcal/mol, whereas the highlighted is the 4th ranked pose.

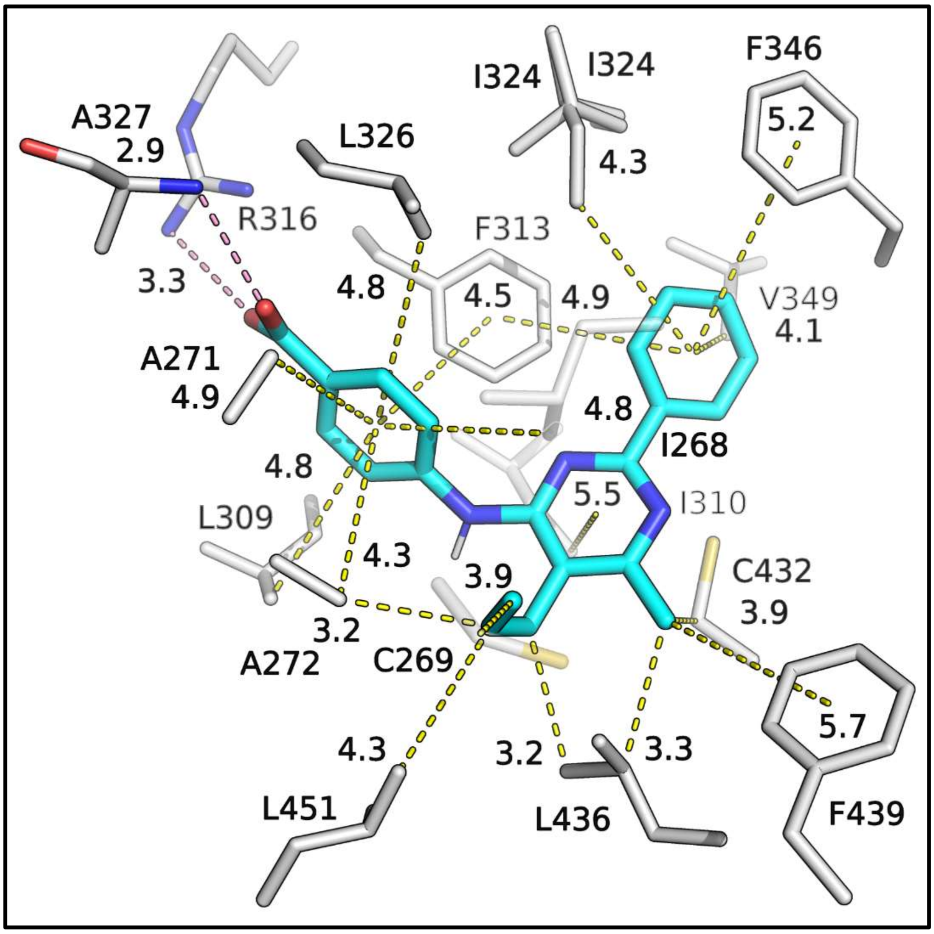

Supplementary Figure S2. Docked pose of XCT (**7a**) in the LBD of RXRα. The ligand is colored with cyan C atoms, while the receptor's side-chain C atoms are colored gray. All other colors are blue for N, red for O and yellow for S. Dashed lines indicate interacting atoms, or ring centroids, with values of the corresponding distance in Å. Polar and H-bonding interactions are highlighted with pink color, whereas Ile324 is shown with both alternative conformations resolved.

Supplementary Scheme 1. Chemical structures of the compounds reported (2 pages).

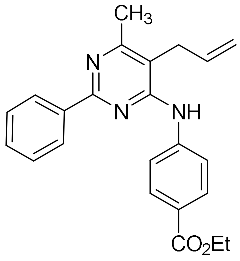
5a

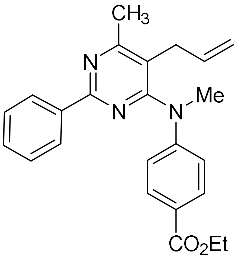
6a

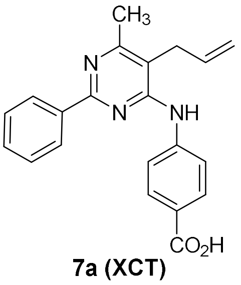
7a (XCT)

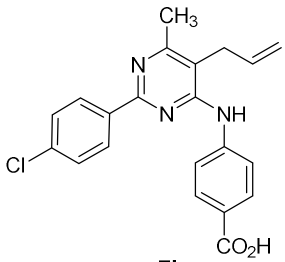
7b

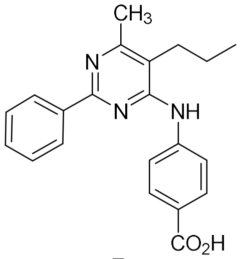
7c

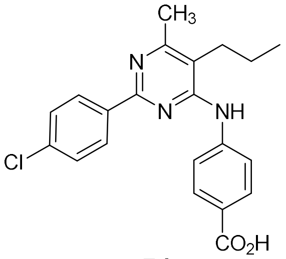
7d

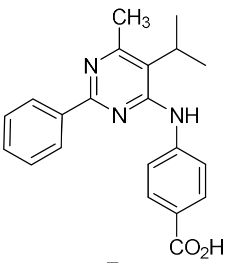
7e

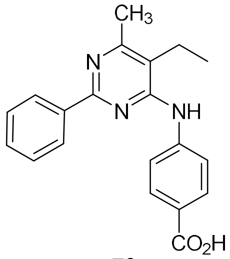
7f

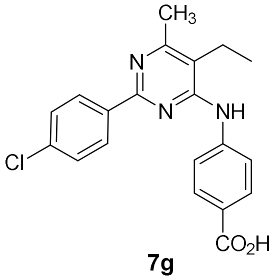
7g

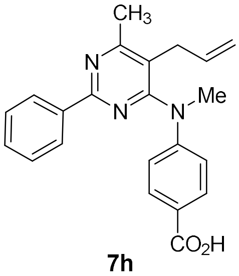
7h

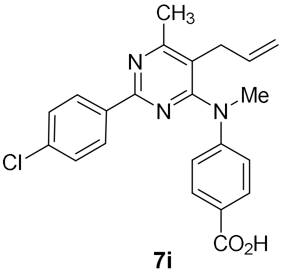
7i

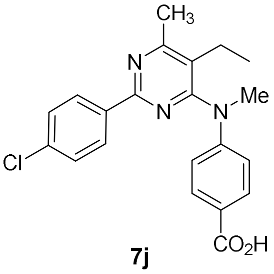
7j

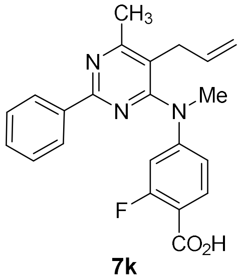
7k

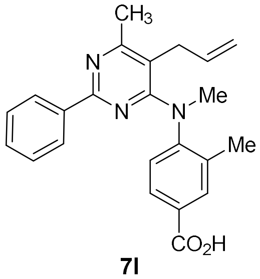
7l

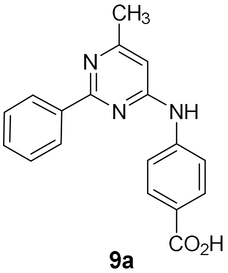
9a

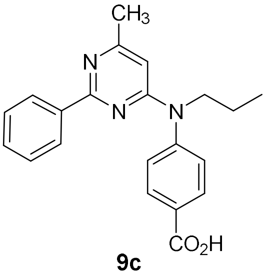
9c

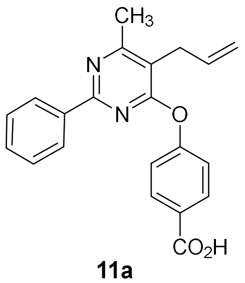
11a

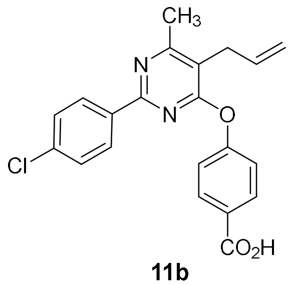
11b

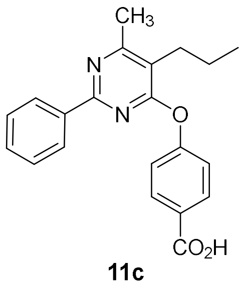
11c

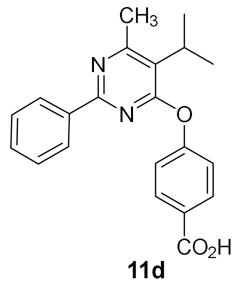
11d

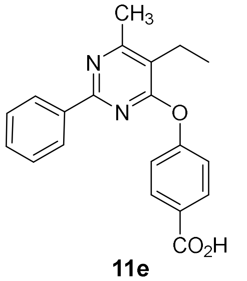
11e

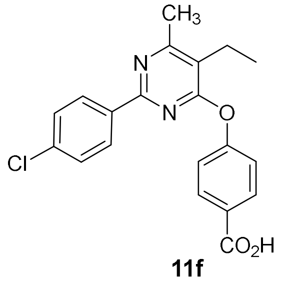
11f

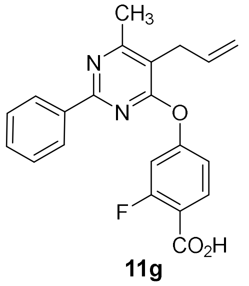
11g

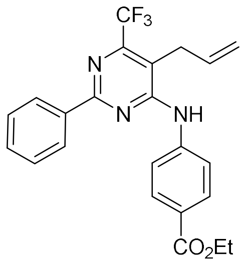
16a

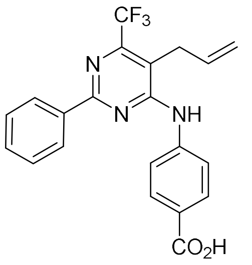
17a

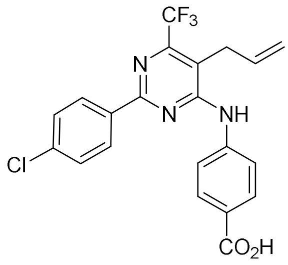
17b

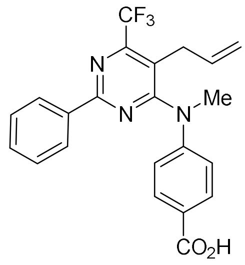
19 (BRF110)

General Methods. All commercially available chemicals were used without further purification. All reactions were performed under an argon atmosphere with dry solvents under anhydrous conditions, unless otherwise noted. Tetrahydrofuran (THF), diethyl ether (Et_2O), methylene chloride (CH_2Cl_2), acetonitrile (CH_3CN), dimethylformamide (DMF), and dimethylsulfoxide (DMSO) were purchased in anhydrous form and used without further purification. Air- and moisture-sensitive liquids were transferred via syringe. Organic solutions were concentrated by rotary evaporation at 40 °C. Flash-column chromatography was performed with silica gel 60 (230-400 mesh). Thin layer chromatography (TLC) was performed on pre-coated silica gel 60 F254 plates and the eluent used is reported in parenthesis. TLC plates were visualized by exposure to ultraviolet light (UV) and/or submersion in aqueous potassium permanganate solution ($KMnO_4/H_2SO_4$) followed by heating.

HPLC analysis for compound purity assessment was performed on an Agilent Eclipse XDB-C18 column with particle size of 5 μm (15 cm × 4.6 mm I.D.), using a chromatographic system comprised of an Agilent 1200 series liquid chromatograph equipped with a 20 μL sample loop injector. The peaks representing the target analyte(s) were recognized both by the retention time and their spectrum pattern recorded on a Diode Array detector working under Agilent ChemStation chromatography software. Elution was accomplished isocratically with a solvent system consisting of 75% CH_3CN-25% H_2O (0.1% TFA). Compounds were dissolved in CH_3CN or a mixture of CH_3CN- H_2O and HPLC chromatograms were recorded in two different wavelengths, 254 and 280 nm, in order to assess their purity. HPLC chromatogram of compound **19** (BRF110), which was tested in vivo, was recorded in three different wavelengths, 214, 254, and 280 nm. All compounds used for biological evaluation had a purity of ≥95% according to HPLC−UV analysis at wavelengths 214, 254 and 280 nm.

Experimental conditions for the synthesis of β-ketoesters 2a-d, 13.

Ethyl 2-acetylpent-4-enoate (2a)

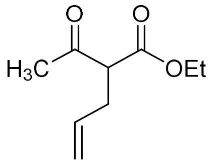

Ketoester **2a** was prepared by the alkylation of ethyl 3-oxobutanoate (**1**) with allyl bromide as described in literature (1). It was isolated in 51% yield as a colorless oil. ^{1}H NMR (250 MHz, $CDCl_3$): *δ* 5.73 (m, 1 H), 5.07 (m, 2 H), 4.18 (q, 2 H, *J* = 7.1 Hz), 3.50 (t, 1 H, *J* = 7.4 Hz), 2.59 (m, 2 H), 2.22 (s, 3 H), 1.25 (t, 3 H, *J* = 7.1 Hz). ^{13}C NMR (62.9 MHz, $CDCl_3$): *δ* 202.3 (C), 169.2 (C), 134.2 (CH), 117.3 (CH_2), 61.3 (CH_2), 59.1 (CH), 32.1 (CH_2), 29.0 (CH_3), 14.0 (CH_3).

Ethyl 2-acetylpentanoate (2b)

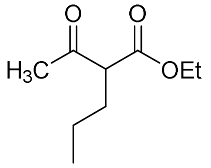

Ketoester **2b** was prepared by the alkylation of ethyl 3-oxobutanoate (**1**) with NaH and *n*-propyl bromide. Ethyl 3-oxobutanoate (1 equiv.) was added dropwise to a suspension of 60% w/w NaH in mineral oil (1 equiv.) in dry THF under argon atmosphere at 0 °C and the resulting mixture was stirred for 30 min. Then addition of neat *n*-propyl bromide (1.1 equiv.) followed, and the reaction mixture was gradually warmed up and stirred at room temperature overnight. It was then diluted with water and extracted with CH_2Cl_2. The combined organic layer was washed with water, dried over $MgSO_4$ and concentrated under reduced pressure to give the crude product. Purification by flash chromatography on silica gel with Hex:EtOAc (20:1 to 10:1) gave the title compound as a colorless oil (57%). Analytical characterization data was identical to the data reported in literature (2). ^{1}H NMR (250 MHz, $CDCl_3$): *δ* 4.18 (q, 2 H, *J* = 7.1 Hz), 3.40 (t, 1 H, *J* = 7.4 Hz), 2.21 (s, 3 H), 1.8 (m, 2 H), 1.26 (m, 5 H), 0.91 (t, 3 H, *J* = 7.2 Hz). ^{13}C NMR (62.9 MHz, $CDCl_3$): *δ* 203.4 (C), 170.0 (C), 61.4 (CH_2), 59.8 (CH), 30.3 (CH_3), 28.8 (CH_2), 20.8 (CH_2), 14.2 (CH_3), 13.9 (CH_3).

Ethyl 2-acetyl-3-methylbutanoate (2c)

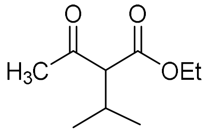

Ketoester **2c** was prepared by the alkylation of ethyl 3-oxobutanoate (**1**) with 2-bromopropane as described in literature (3). It was isolated in 35% yield as a colorless oil. ^{1}H NMR (250 MHz, $CDCl_3$): δ 4.17 (q, 2 H, J = 7.2 Hz), 3.16 (d, 1 H, J = 9.5 Hz), 2.40 (m, 1 H), 2.21 (s, 3 H), 1.26 (t, 2 H, J = 7.1 Hz), 0.96 (d, 3 H, J = 6.5 Hz), 0.92 (d, 3 H, J = 6.7 Hz).

Ethyl 2-ethyl-3-oxobutanoate (2d)

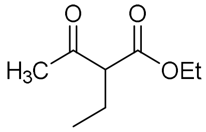

Ketoester **2d** was prepared by the alkylation of ethyl 3-oxobutanoate (**1**) with ethyl iodide as described in literature (3, 4). It was isolated in 49% yield as a colorless oil. ^{1}H NMR (250 MHz, $CDCl_3$): δ 4.18 (q, 2 H, J = 7.1 Hz), 3.31 (t, 1 H, J = 7.3 Hz), 2.20 (s, 2 H), 1.87 (m, 2 H), 1.25 (t, 3 H, J = 7.1 Hz), 0.91 (t, 3 H, J = 7.4 Hz). ^{13}C NMR (62.9 MHz, $CDCl_3$): δ 203.4 (C), 169.9 (C), 61.6 (CH_2), 61.4 (CH), 28.9 (CH_3), 21.7 (CH_2), 14.2 (CH_3), 12.0 (CH_3).

Ethyl 2-(2,2,2-trifluoroacetyl)pent-4-enoate (13)

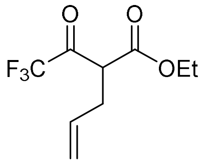

Ketoester **13** was prepared by the alkylation of ethyl 4,4,4-trifluoro-3-oxobutanoate (**12**) with allyl bromide as described in literature (5). To a suspension of 60% w/w NaH in mineral oil (1.08 g, 27.1 mmol) in 20 mL of dry THF under argon atmosphere at 0 °C, a solution of ethyl 4,4,4-trifluoro-3-oxobutanoate (**12**) (5.0 g, 27.1 mmol) in THF (10 mL) was added dropwise. The mixture was stirred at 0 °C for 1 h and the solvent was then evaporated under reduced pressure to give a white solid which was suspended in acetone (15 mL) followed by treatment with KI (449 mg, 2.7 mmol). The resulting suspension was stirred at room temperature for 15 min followed by dropwise addition of a solution of allyl bromide (3.3 g, 27.2 mmol) in acetone (10 mL). The

reaction mixture was heated at 60 °C for 48 h and the solvent was then evaporated under reduced pressure. The resulting residue was treated with 1N HCl (50 mL) and then extracted with CH_2Cl_2 (2 × 30 mL). The combined organic layer was washed with water, dried over $MgSO_4$, filtered and concentrated under reduced pressure to give the title compound (3.3 g, 54%) as an orange oil, which was used in the next step without any further purification. ^{1}H NMR (250 MHz, $CDCl_3$): δ 5.73 (m, 1 H), 5.12 (m, 2 H), 4.21 (q, 2 H, J = 7.1 Hz), 3.92 (t, 1 H, J = 7.2 Hz), 2.70 (t, 2 H, J = 7.3 Hz), 1.26 (t, 3 H, J = 7.1 Hz). ^{13}C NMR (62.9 MHz, $CDCl_3$): δ 186.6 (C, q, $J_{C\text{-}F}$ = 36.5 Hz), 166.8 (C), 132.7 (CH), 118.8 (CH_2), 115.4 (C, d, $J_{C\text{-}F}$ = 292 Hz), 62.5 (CH_2), 53.0 (CH), 31.9 (CH_2), 13.9 (CH_3).

References

(1) Baker, T. M.; Sloan, L. A.; Choudhury, L. H.; Murai, M.; Procter, D. J. A stereoselective cyclisation cascade mediated by SmI_2–H_2O: synthetic studies towards stolonidiol. *Tetrahedron: Asymmetry* **2010**, *21*, 1246–1261.

(2) Nakamura, K.; Miyai, T.; Nagar, A.; Oka, S.; Ohno, A. Stereochemical control in microbial reduction. 9. Diastereoselective reduction of 2-alkyl-3-oxobutanoate with Bakers' yeast. *Bull. Chem. Soc. Jpn.* **1989**, *62*, 1179-1187.

(3) Beddow, J. E.; Davies, S. G.; Ling, K. B.; Roberts, P. M.; Russel, A. J.; Smith, A. D. Asymmetric synthesis of β^2-amino acids: 2-substituted-3-aminopropanoic acids from *N*-acryloyl SuperQuat derivatives. *Org. Biomol. Chem.* **2007**, *5*, 2812-2825.

(4) Bishop, J. E.; Nagy, J. O.; O'Connell, J. F.; Rapoport, H. Diastereoselective Synthesis of Phycocyanobilin-Cysteine Adducts. *J. Am. Chem. Soc.* **1991**, *113*, 8024-8035.

(5) Aubert, C.; Bégué, J.-P.; Charpentier-Morize, M.; Nee, G.; Langlois. B. Methode generale d' acces aux trifluoromethylcetones. 1ère partie: Alkylation directe du trifluoroacetylacetate d'ethyle. *J. Fluorine Chem.* **1989**, *44*, 361-376.

Compound 5a

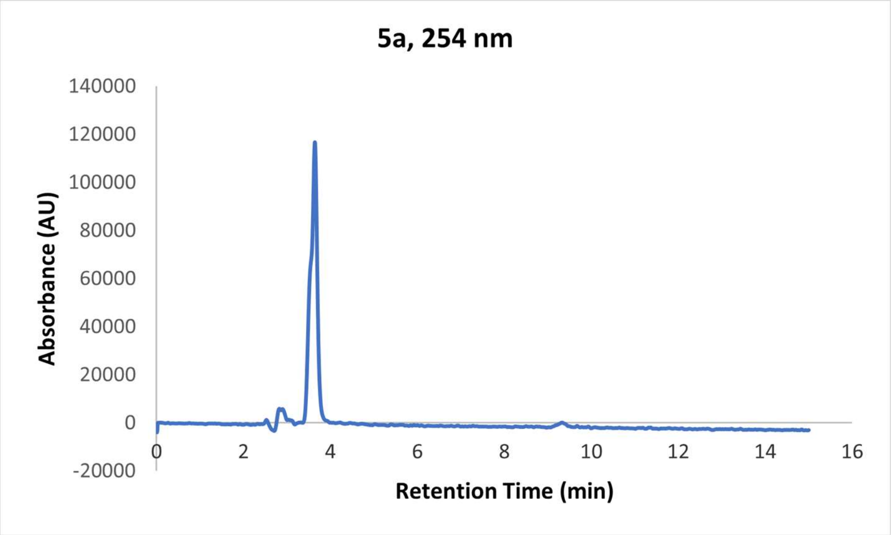

Peak#	R.Time	Area	Area %	Height
1	3.641	1257388	**97.95**	116704
2	9.321	26266	2.05	1916
		1283654	100	118620

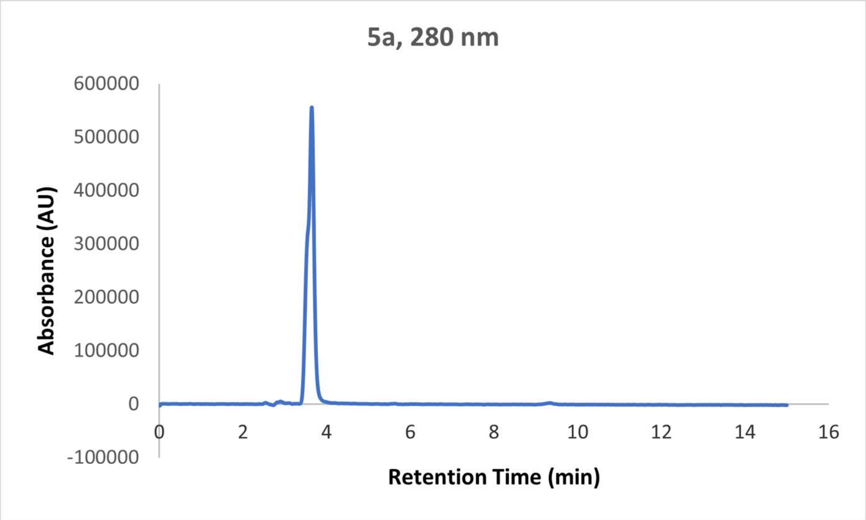

Peak#	R.Time	Area	Area %	Height
1	3.642	6062193	**99.48**	554624
2	9.334	31508	0.52	2533
		6093701	100	557157

Compound 7a

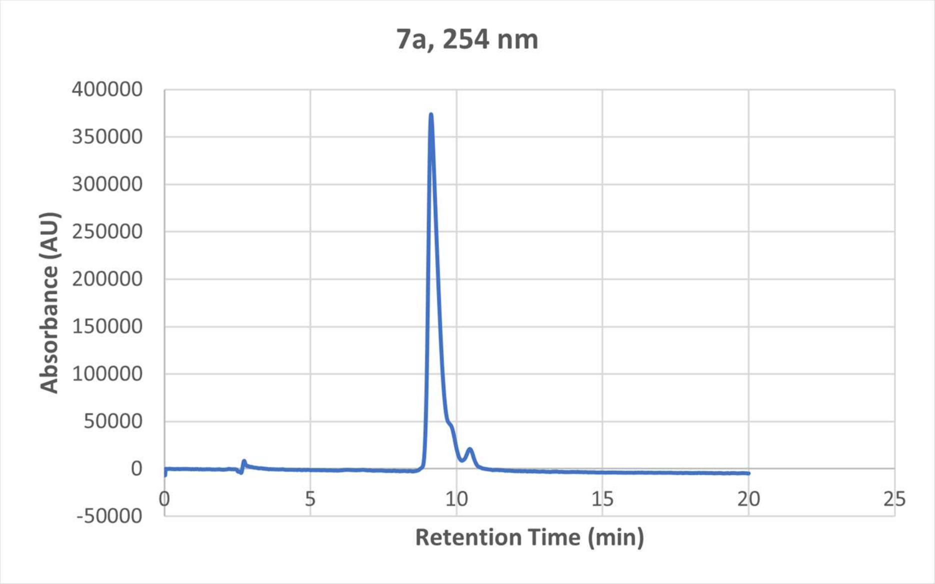

Peak#	R.Time	Area	Area %	Height
1	9.122	9465068	**97.57**	372370
2	10.447	235619	2.43	15354
		9700687	100	387724

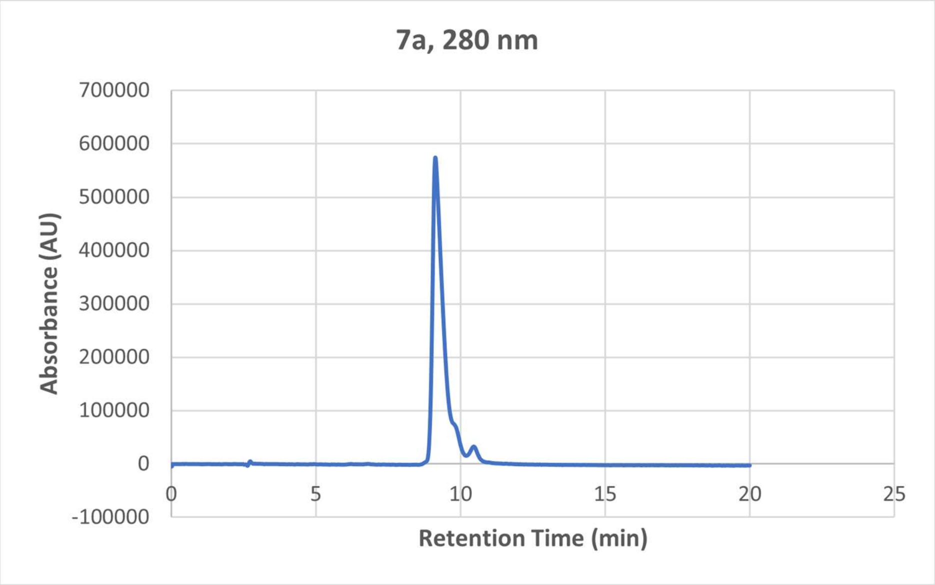

Peak#	R.Time	Area	Area %	Height
1	9.123	14572300	**97.75**	569400
2	10.451	336046	2.25	21730
		14908346	100	591130

Compound 7c

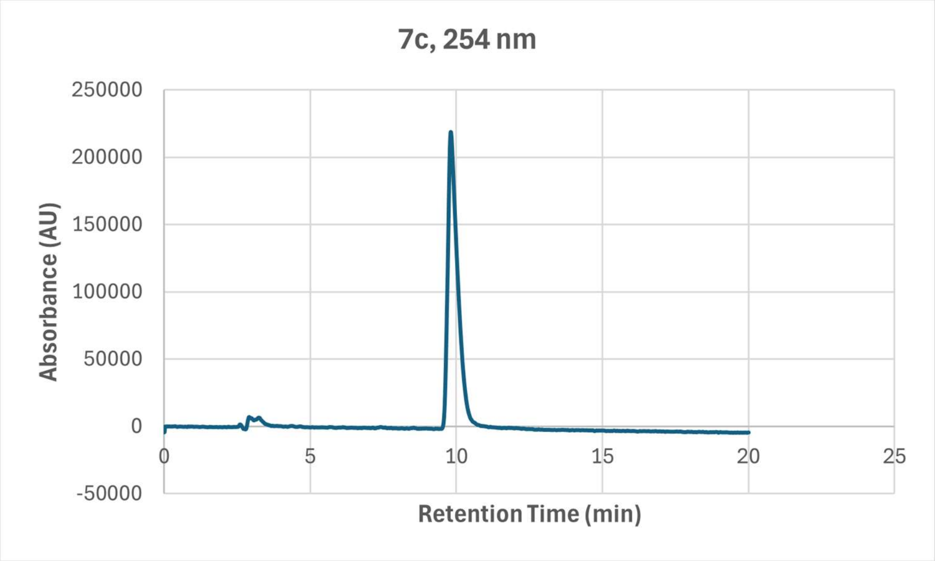

Peak#	R.Time	Area	Area %	Height
1	2.907	235109	4.33	8653
2	9.807	5189094	**95.67**	220084
		5424203	100	228737

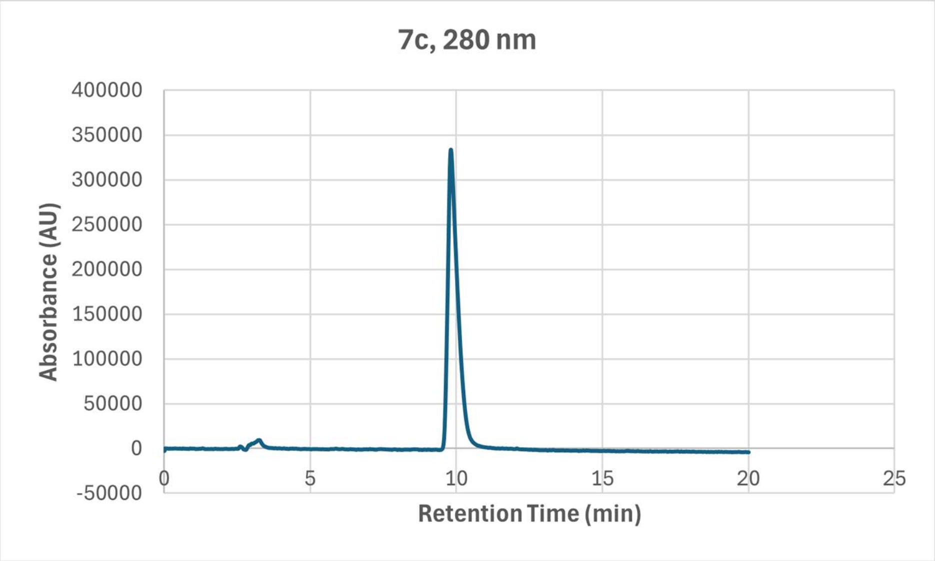

Peak#	R.Time	Area	Area %	Height
1	3.257	240701	2.94	9689
2	9.808	7959312	**97.06**	335271
		8200013	100	344960

Compound 7d

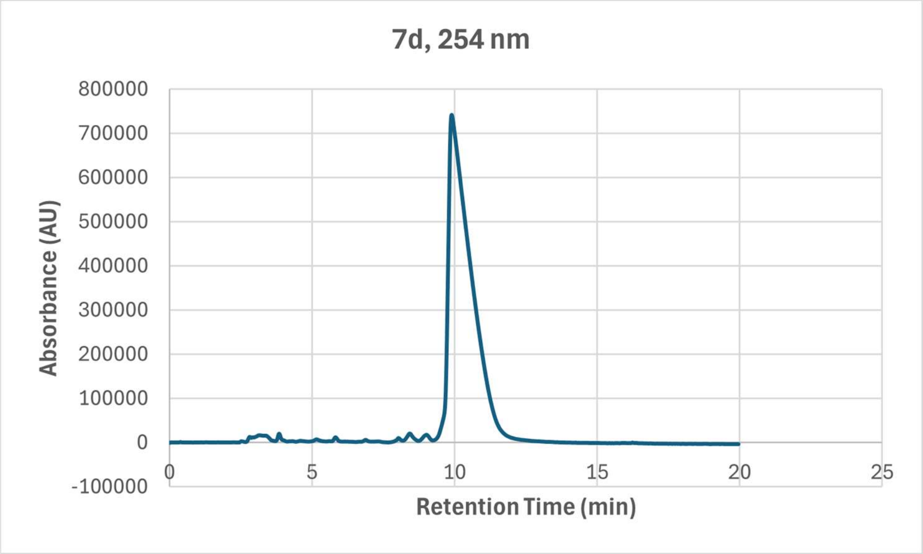

Peak#	R.Time	Area	Area %	Height
1	3.839	730049	1.75	17949
2	5.817	150140	0.36	9579
3	8.422	545915	1.31	17740
4	9.892	40276060	**96.58**	736075
		41702164	100	781343

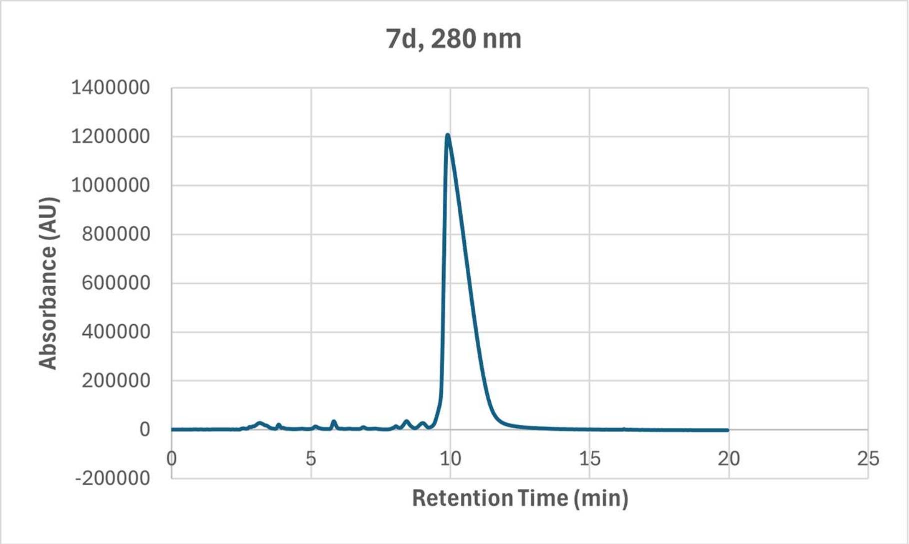

Peak#	R.Time	Area	Area %	Height
1	3.165	829736	1.15	23665
2	5.817	374500	0.52	31036
3	8.426	860976	1.18	30308
4	9.898	70381636	**97.15**	1199391
		72446848	100	1284400

Compound 7e

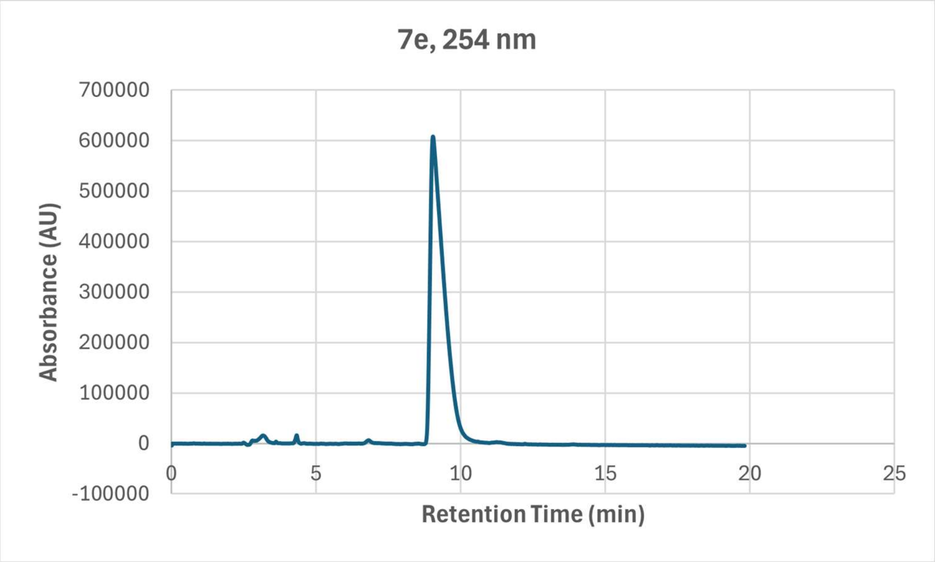

Peak#	R.Time	Area	Area %	Height
1	3.16	178644	0.87	12340
2	4.326	90782	0.44	16122
3	6.812	70354	0.34	6270
4	9.037	20235820	**98.35**	608745
		20575600	100	643477

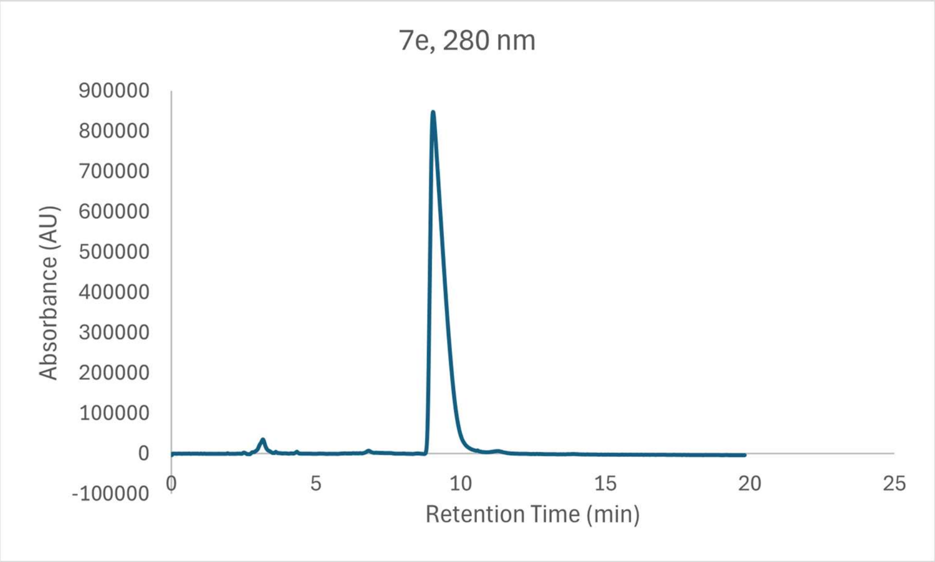

Peak#	R.Time	Area	Area %	Height
1	3.159	595028	2.02	35066
2	6.814	62046	0.21	5805
3	9.04	28761552	**97.77**	847954
		29418626	100	888825

Compound 7f

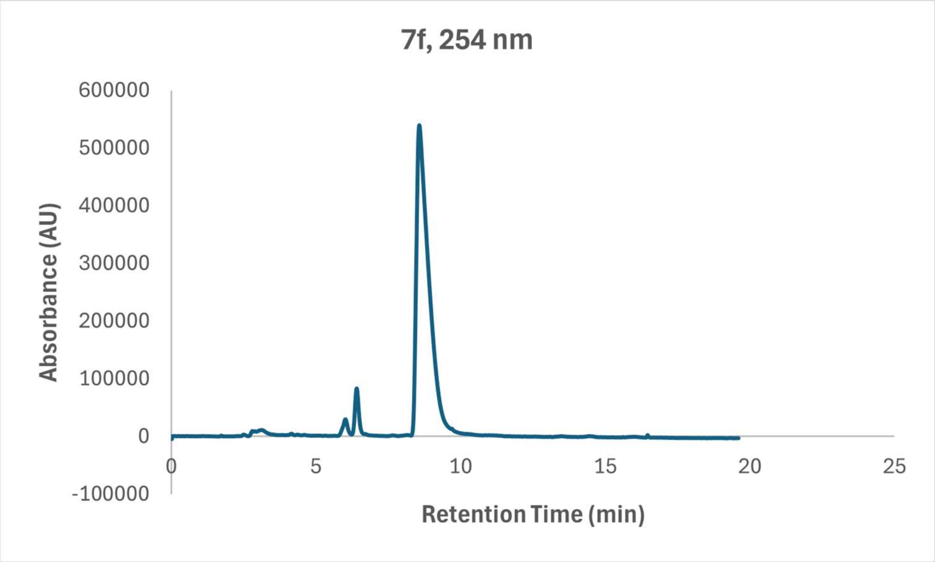

Peak#	R.Time	Area	Area %	Height
1	3.116	308462	1.77	9816
2	6.402	1042773	5.98	80245
3	8.568	16083184	**92.25**	538762
		17434419	100	628823

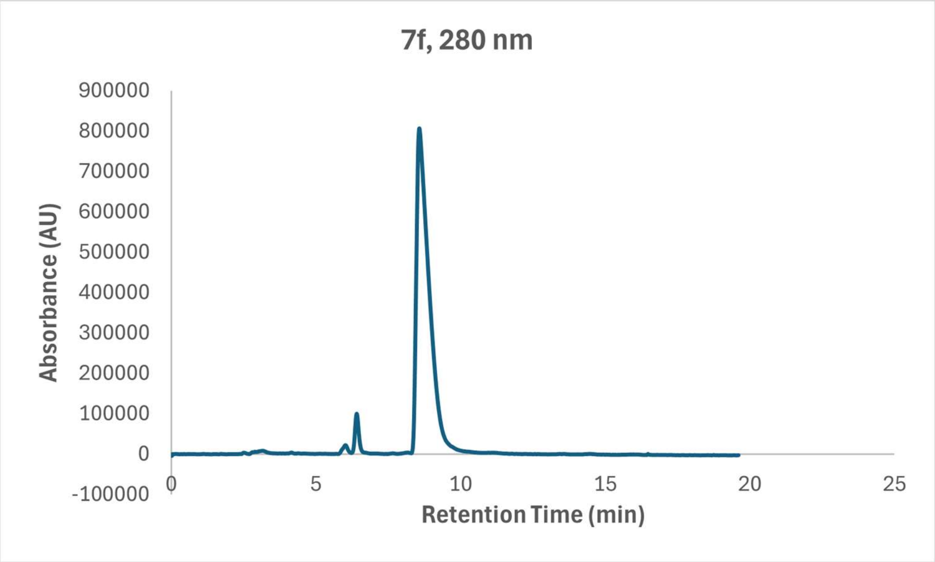

Peak#	R.Time	Area	Area %	Height
1	3.167	196593	0.76	7105
2	6.403	1208564	4.71	98863
3	8.568	24267217	**94.53**	803860
		25672374	100	909828

Compound 7g

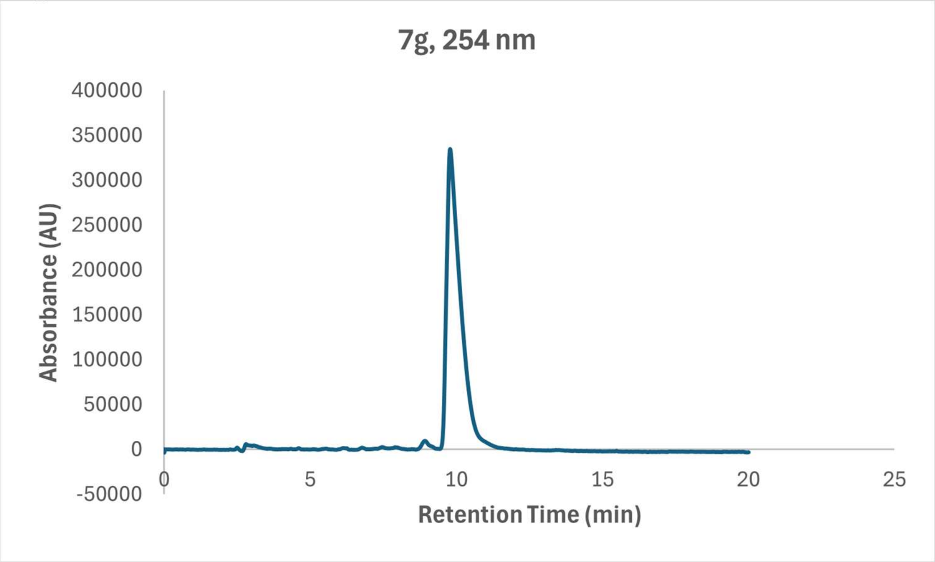

Peak#	R.Time	Area	Area %	Height
1	8.922	161608	1.45	9314
2	9.783	10997218	**98.55**	334131
		11158826	100	343445

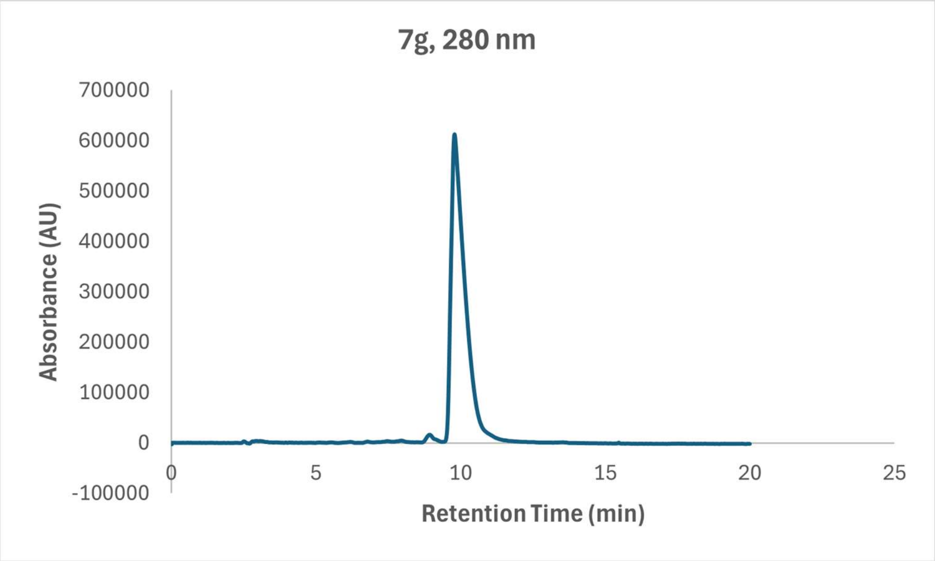

Peak#	R.Time	Area	Area %	Height
1	8.924	262595	1.27	14790
2	9.786	20338219	**98.73**	610311
		20600814	100	625101

Compound 7h

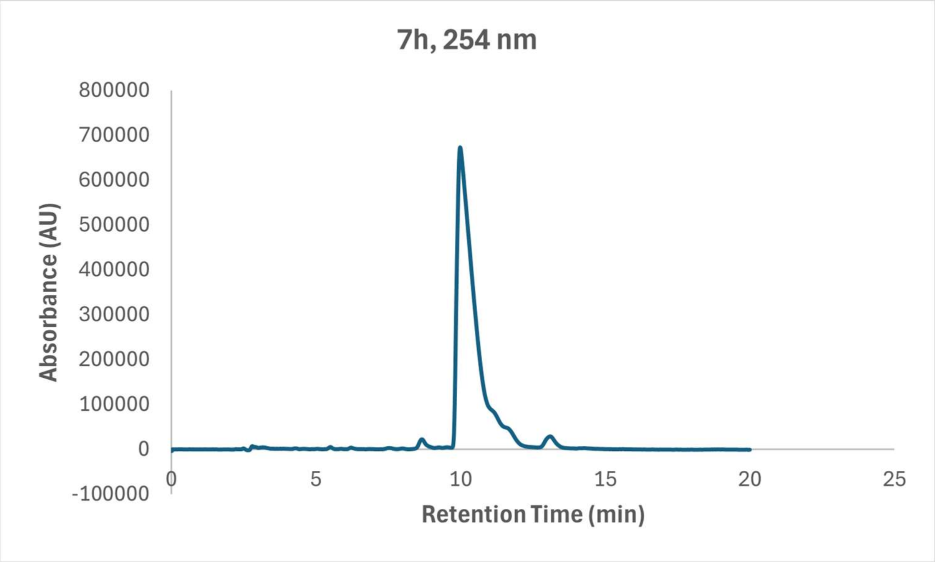

Peak#	R.Time	Area	Area %	Height
1	8.657	328046	1.11	20734
2	9.98	28546908	**96.92**	669720
3	13.094	580339	1.97	25087
		29455293	100	715541

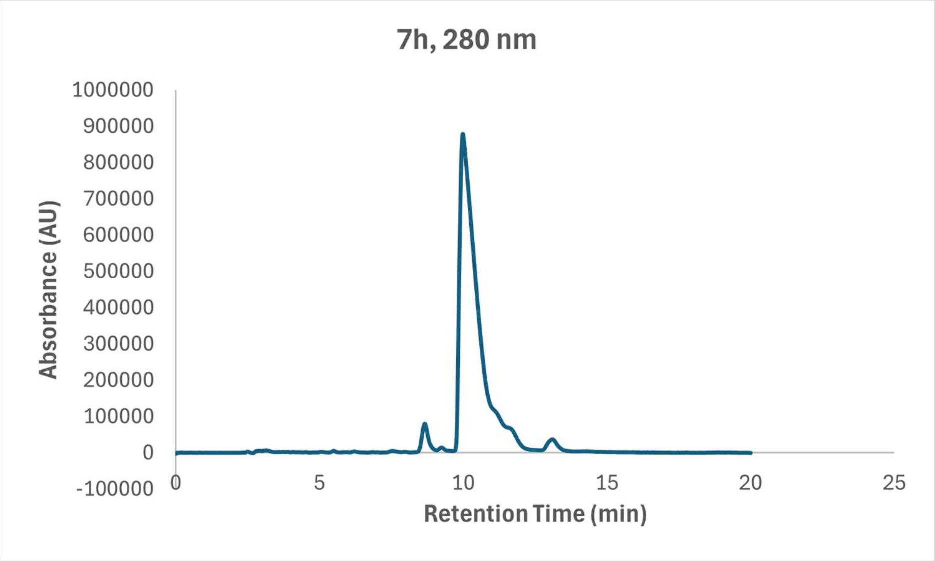

Peak#	R.Time	Area	Area %	Height
1	8.66	1129724	2.85	76482
2	9.983	37832233	**95.31**	873633
3	13.097	728873	1.84	31274
		39690830	100	981389

Compound 7l

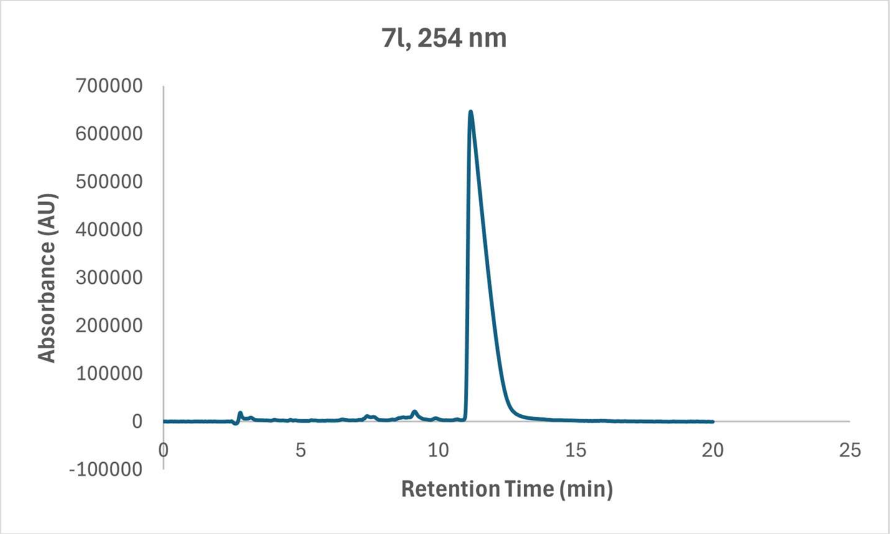

Peak#	R.Time	Area	Area %	Height
1	2.793	310032	1.01	21331
2	7.414	177887	0.58	8104
3	9.138	378537	1.23	17286
4	11.182	29867637	**97.18**	644363
		30734093	100	691084

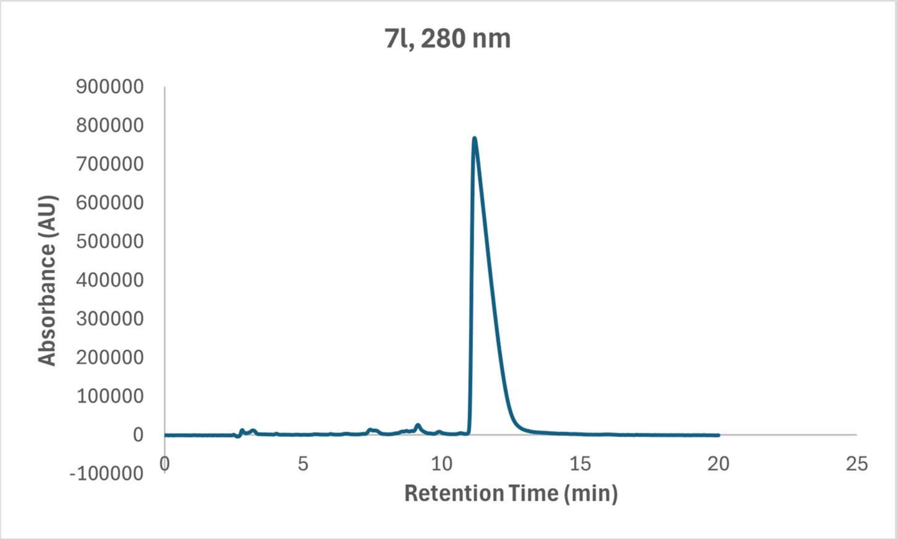

Peak#	R.Time	Area	Area %	Height
1	2.794	330723	0.90	15616
2	7.418	247235	0.68	11330
3	9.141	594187	1.62	23367
4	11.184	35466550	**96.80**	763844
		36638695	100	814157

Compound 11c

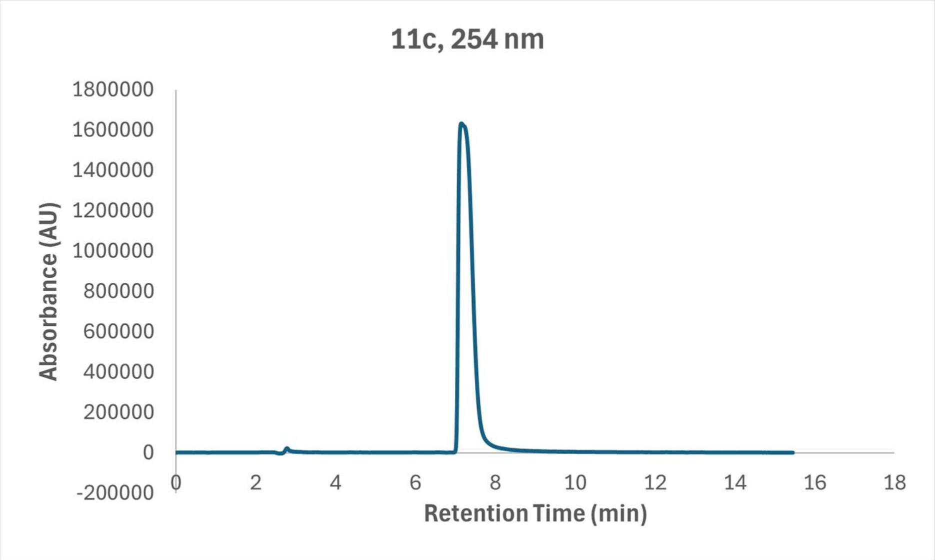

Peak#	R.Time	Area	Area %	Height
1	7.145	39029197	100	1630201

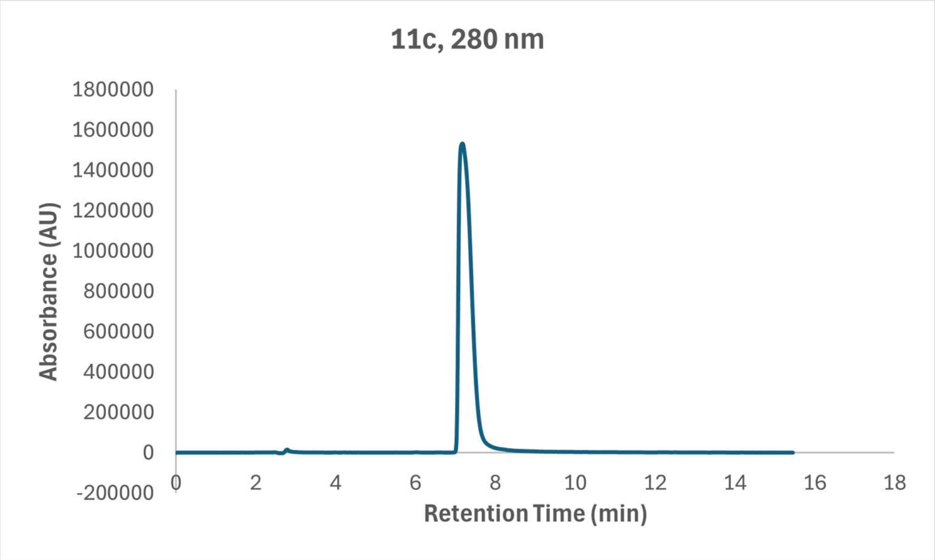

Peak#	R.Time	Area	Area %	Height
1	7.171	33689393	100	1532752

Compound 11d

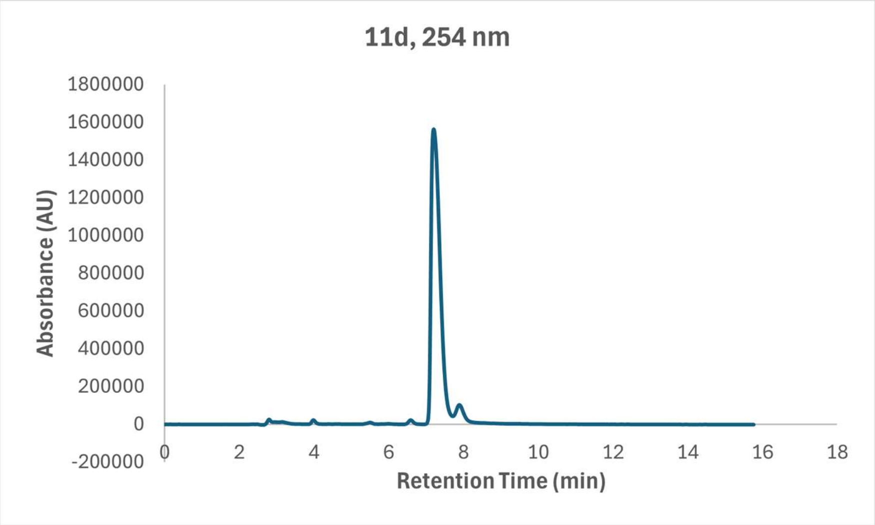

Peak#	R.Time	Area	Area %	Height
1	2.792	140821	0.53	21451
2	3.976	151781	0.58	21460
3	6.578	192045	0.73	21794
4	7.194	25142733	**95.29**	1548148
5	7.881	757100	2.87	67660
		26384480	100	1680513

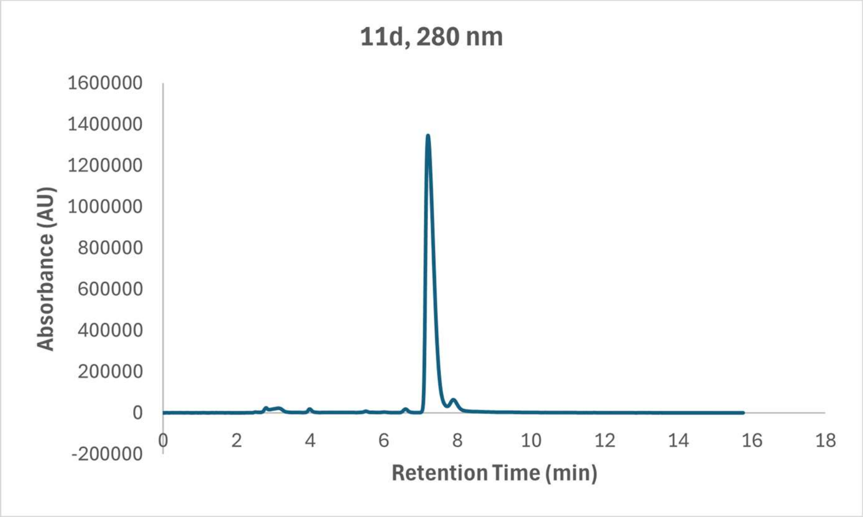

Peak#	R.Time	Area	Area %	Height
1	2.793	564049	2.63	21672
2	3.978	126547	0.59	17144
3	6.581	151647	0.71	16929
4	7.193	20156502	**94.09**	1337036
5	7.881	424054	1.98	38880
		21422799	100	1431661

Compound 11f

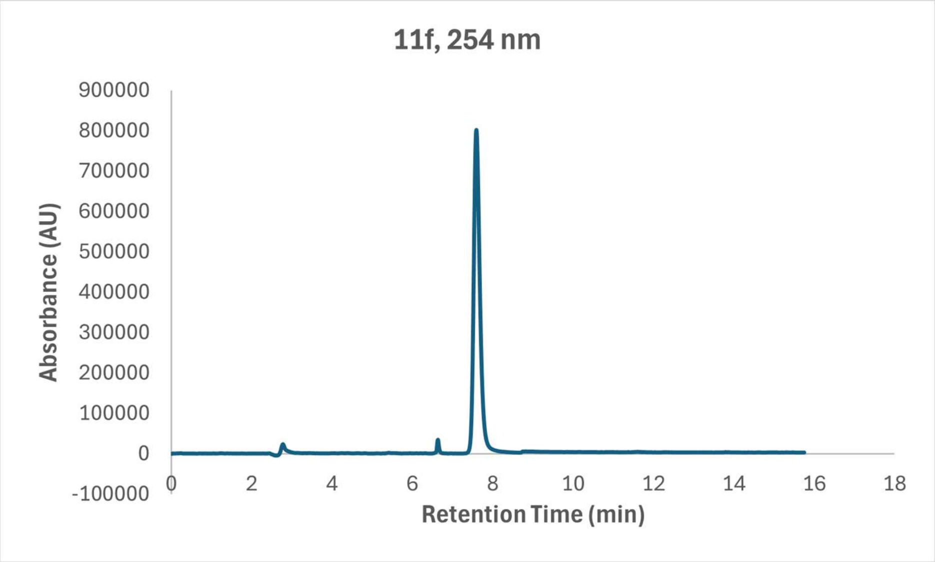

Peak#	R.Time	Area	Area %	Height
1	2.771	231949	2.57	26264
2	6.628	125390	1.39	34101
3	7.586	8673074	**96.04**	801083
		9030413	100	861448

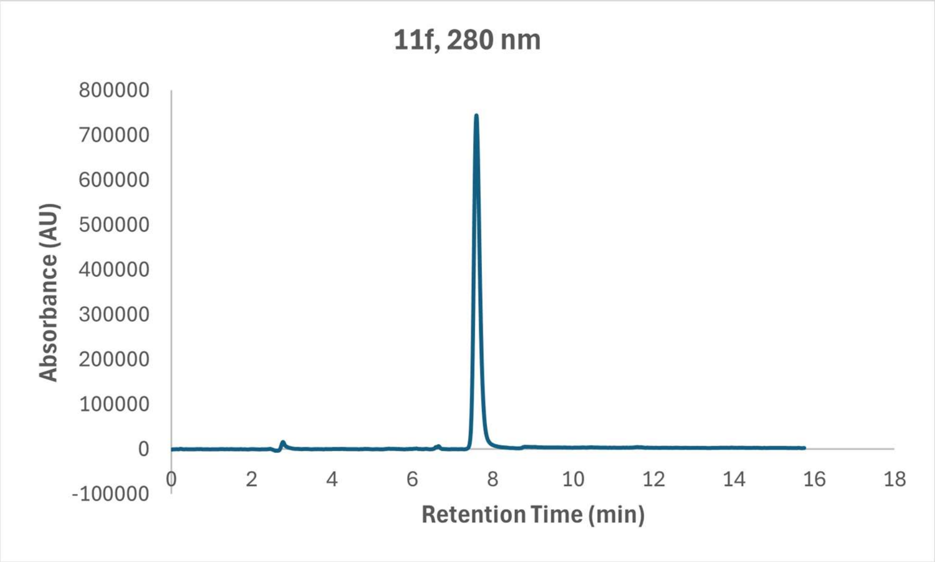

Peak#	R.Time	Area	Area %	Height
1	2.773	162889	1.97	18558
2	6.644	47225	0.57	6830
3	7.587	8066706	**97.46**	744260
		8276820	100	769648

Compound 17a

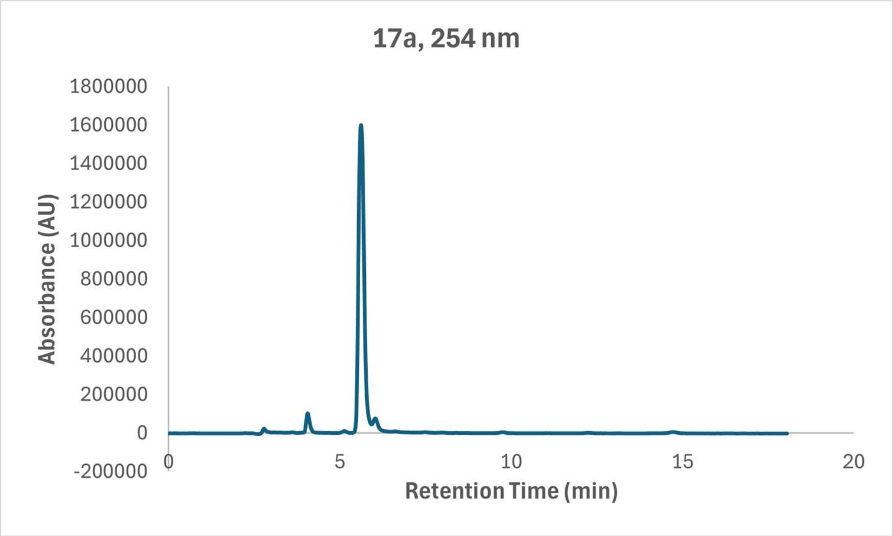

Peak#	R.Time	Area	Area %	Height
1	2.784	201711	1.09	25514
2	4.056	716871	3.87	99943
3	5.614	17397466	**93.85**	1576841
4	6.026	220707	1.19	39269
		18536755	100	1741567

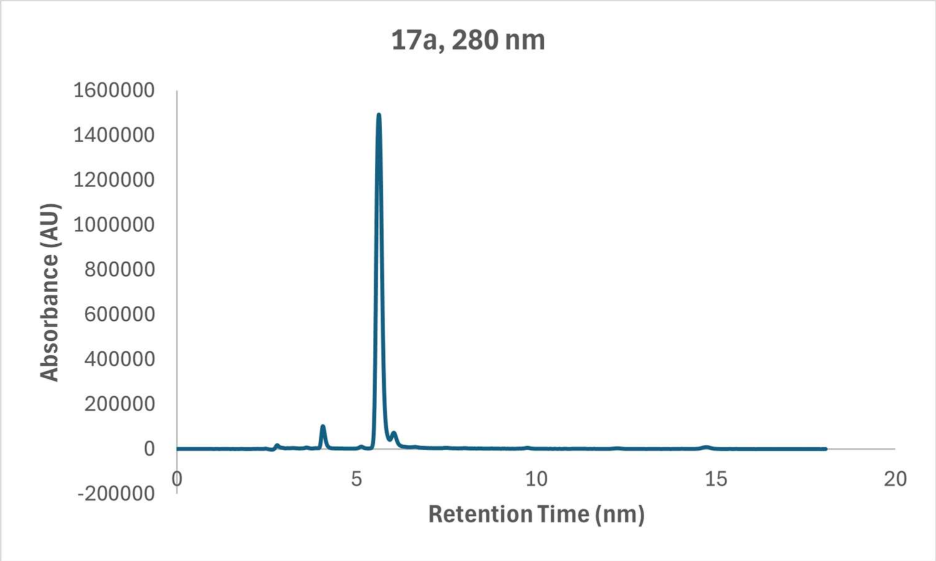

Peak#	R.Time	Area	Area %	Height
1	2.787	137411	0.80	17794
2	4.057	714777	4.17	98608
3	5.615	16059445	**93.61**	1473331
4	6.029	244258	1.42	39518
		17155891	100	1629251

Compound 19 (BRF110)

214 nm (98% purity)

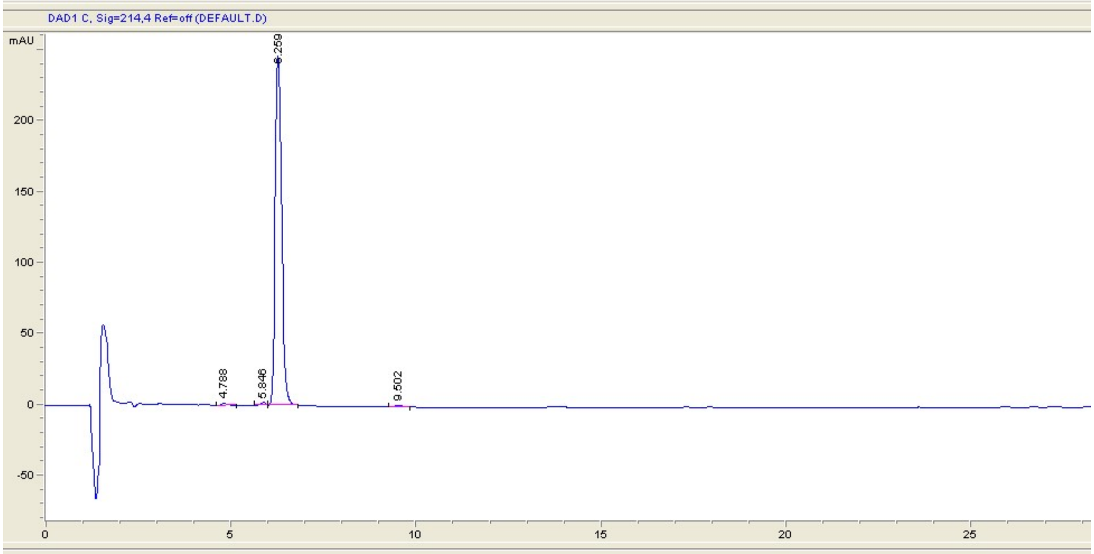

#	Time	Area	Height	Width	Area%	Symmetry
1	4.788	19.8	1.8	0.1632	0.658	0.683
2	5.846	21	2	0.1606	0.699	0.962
3	6.259	2941.8	246.7	0.1831	98.014	0.78
4	9.502	18.9	1.1	0.2522	0.629	0.643

254 nm (95.8% purity)

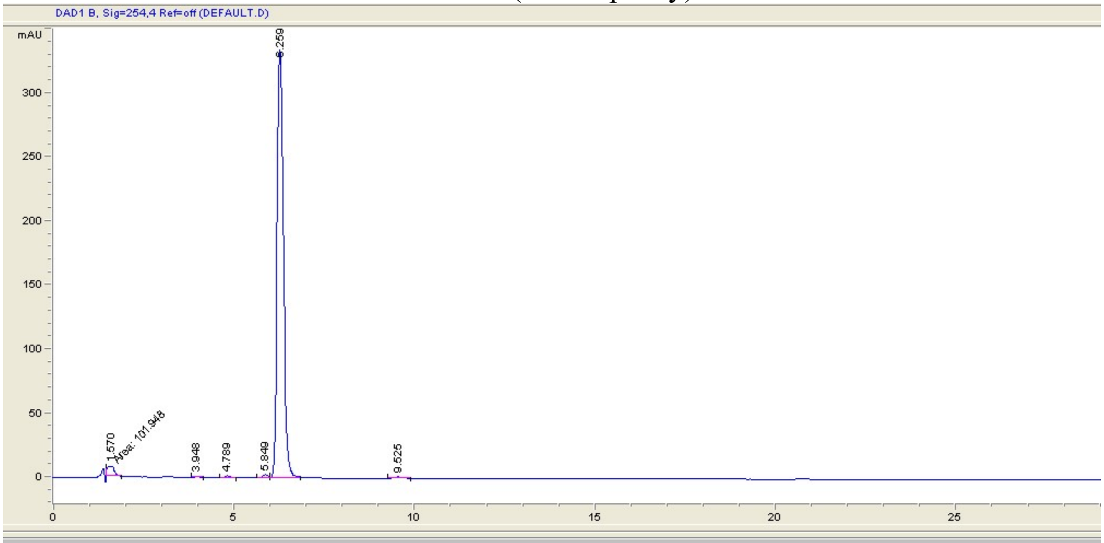

#	Time	Area	Height	Width	Area%	Symmetry
1	1.57	101.9	7.9	0.2152	2.460	0.69
2	3.948	8.4	1.1	0.1154	0.202	0.849
3	4.789	15.5	1.6	0.1452	0.373	0.768
4	5.849	25.3	2.4	0.1642	0.611	0.921
5	6.259	3972.7	334.6	0.1825	95.846	0.784
6	9.525	21	1.2	0.2598	0.508	0.688

280 nm (95.3 % purity)

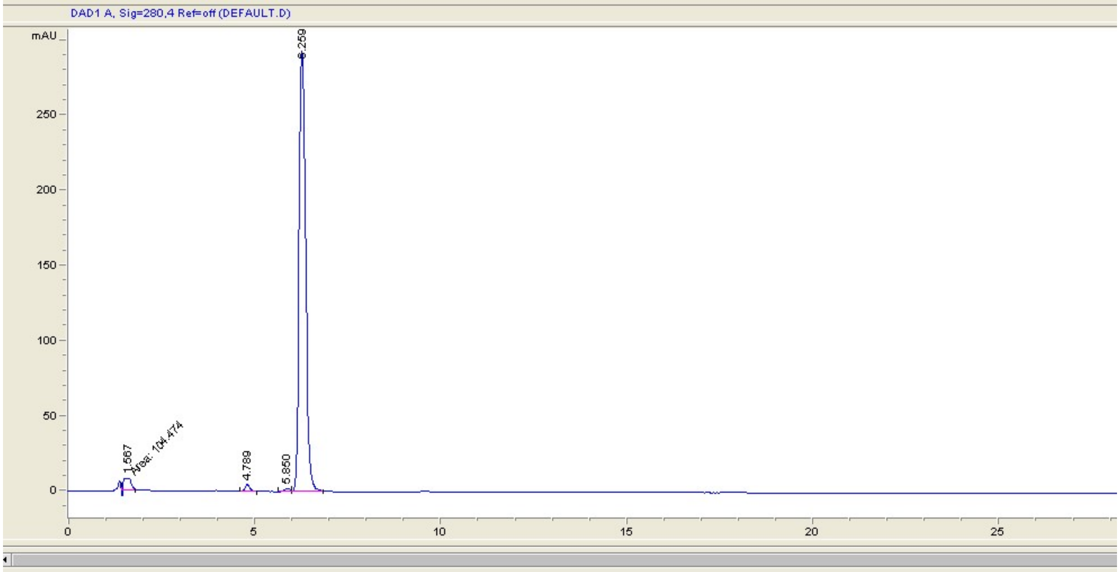

#	Time	Area	Height	Width	Area%	Symmetry
1	1.567	104.5	7.7	0.2269	2.849	0.88
2	4.789	44.8	4.8	0.1439	1.221	0.768
3	5.85	22.1	2.1	0.1664	0.602	0.947
4	6.259	3495.2	293.5	0.1829	95.328	0.783

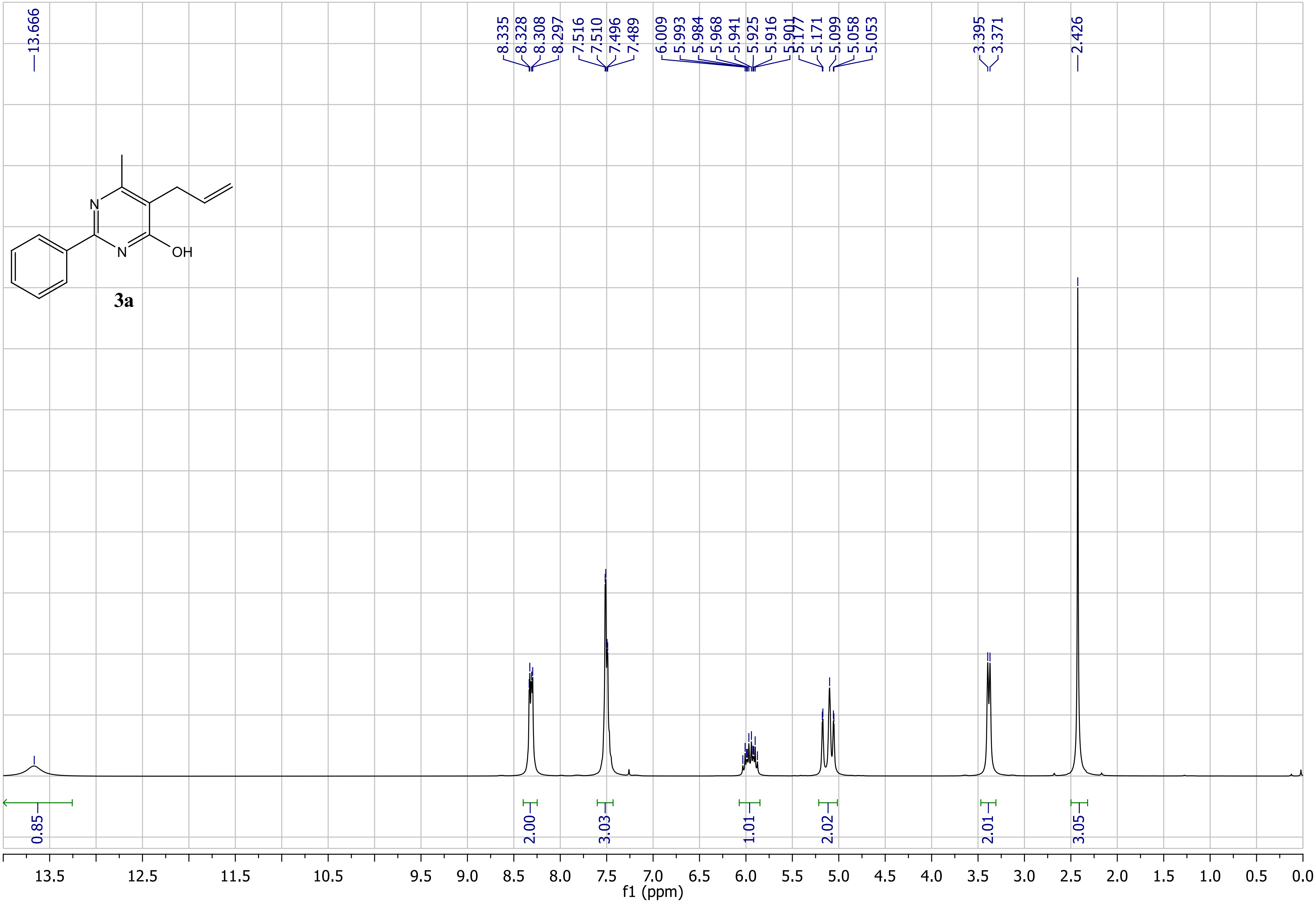

13.666
8.335
8.328
8.308
8.297
7.516
7.510
7.496
7.489
6.009
5.993
5.984
5.968
5.941
5.925
5.916
5.901
5.177
5.171
5.099
5.058
5.053
3.395
3.371
2.426
N
N
OH
3a
0.85
2.00
3.03
1.01
2.02
2.01
3.05
f1 (ppm)

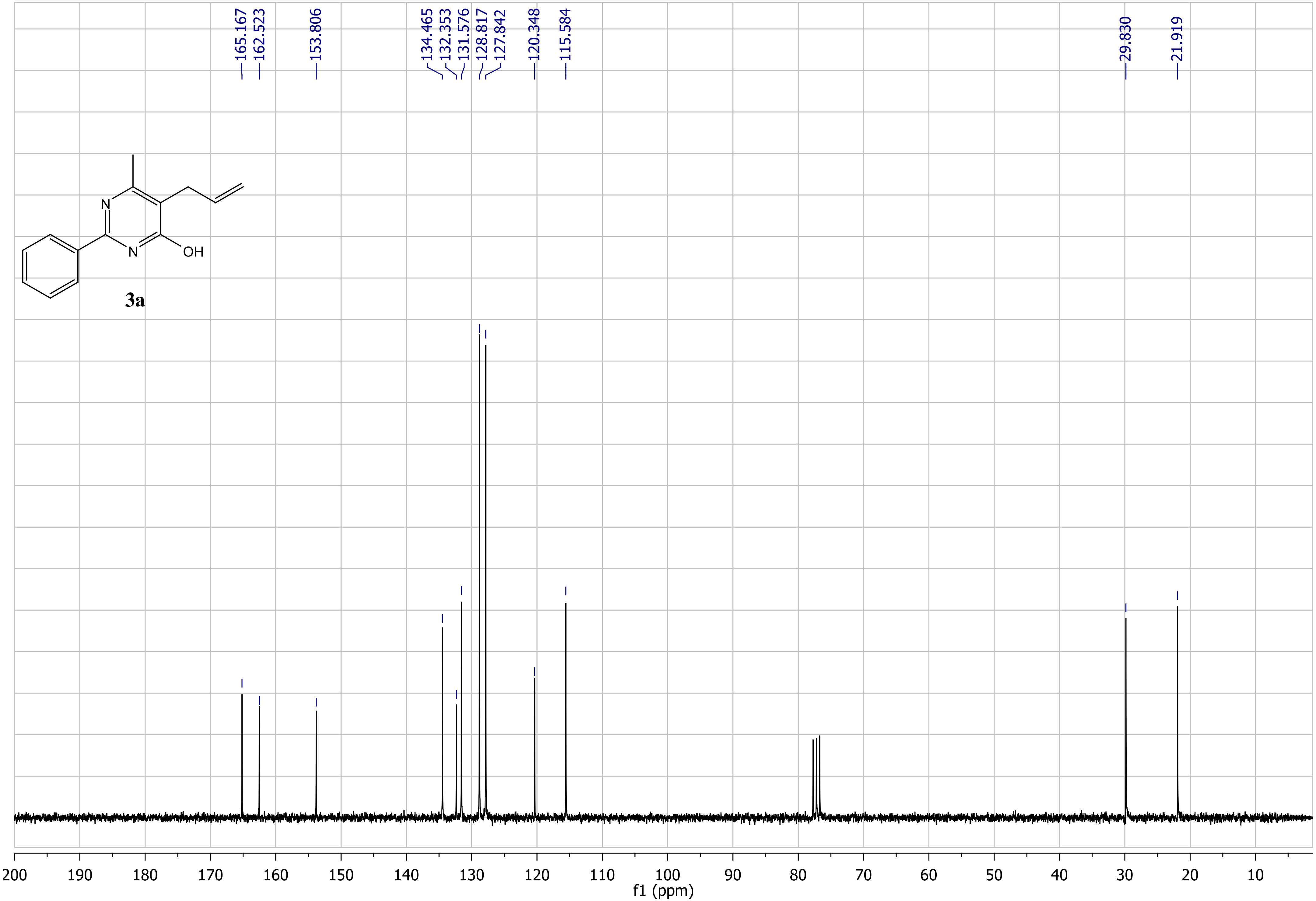
165.167
162.523
153.806
134.465
132.353
131.576
128.817
127.842
120.348
115.584
29.830
21.919
N
N
OH
3a
200
190
180
170
160
150
140
130
120
110
100
90
80
70
60
50
40
30
20
10
f1 (ppm)

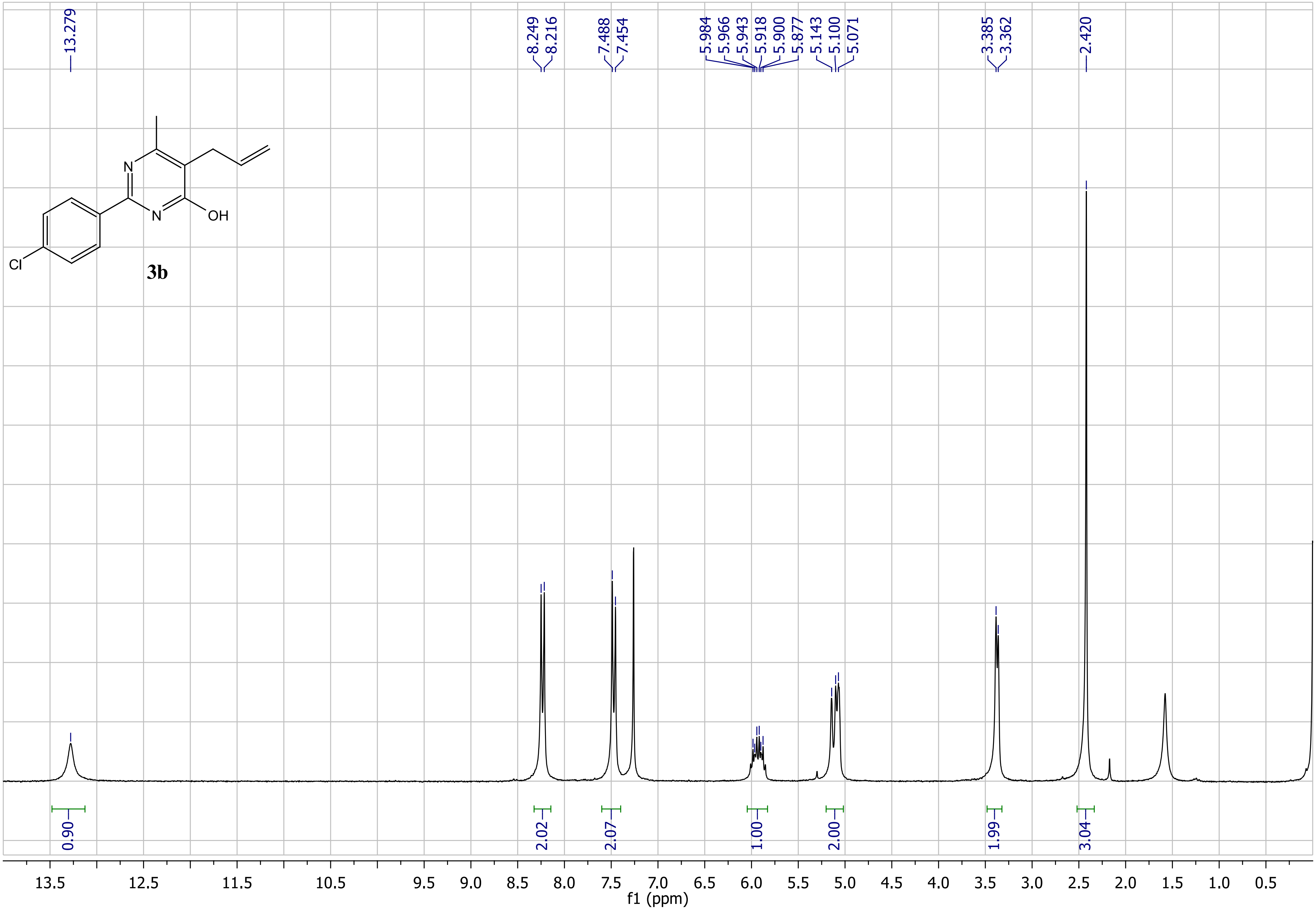
13.279
8.249
8.216
7.488
7.454
5.984
5.966
5.943
5.918
5.900
5.877
5.143
5.100
5.071
3.385
3.362
2.420
N
N
OH
Cl
3b
0.90
2.02
2.07
1.00
2.00
1.99
3.04
13.5 12.5 11.5 10.5 9.5 9.0 8.5 8.0 7.5 7.0 6.5 6.0 5.5 5.0 4.5 4.0 3.5 3.0 2.5 2.0 1.5 1.0 0.5
f1 (ppm)

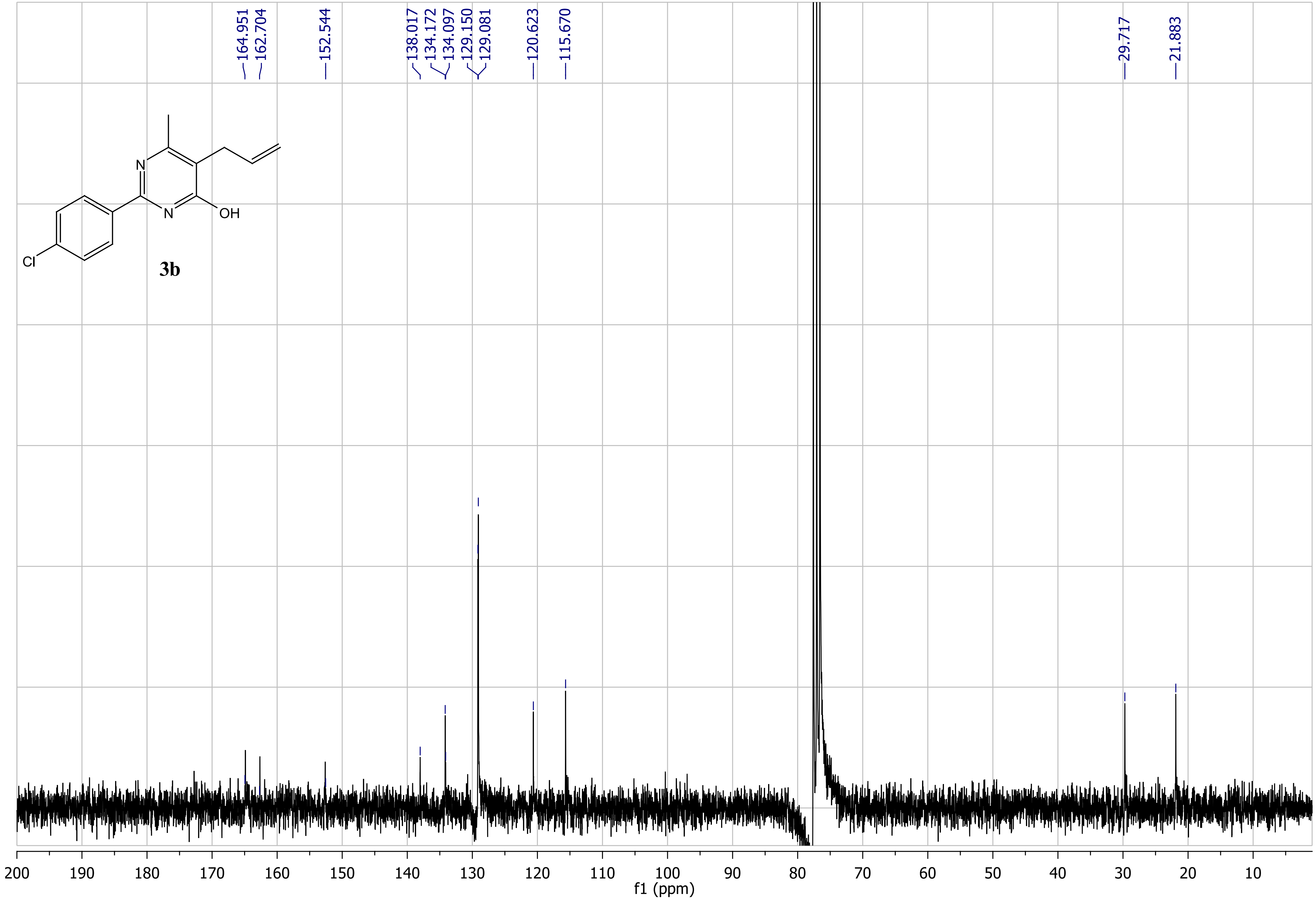
164.951
162.704
152.544
138.017
134.172
134.097
129.150
129.081
120.623
115.670
29.717
21.883
N
N
OH
Cl
3b
200 190 180 170 160 150 140 130 120 110 100 90 80 70 60 50 40 30 20 10
f1 (ppm)

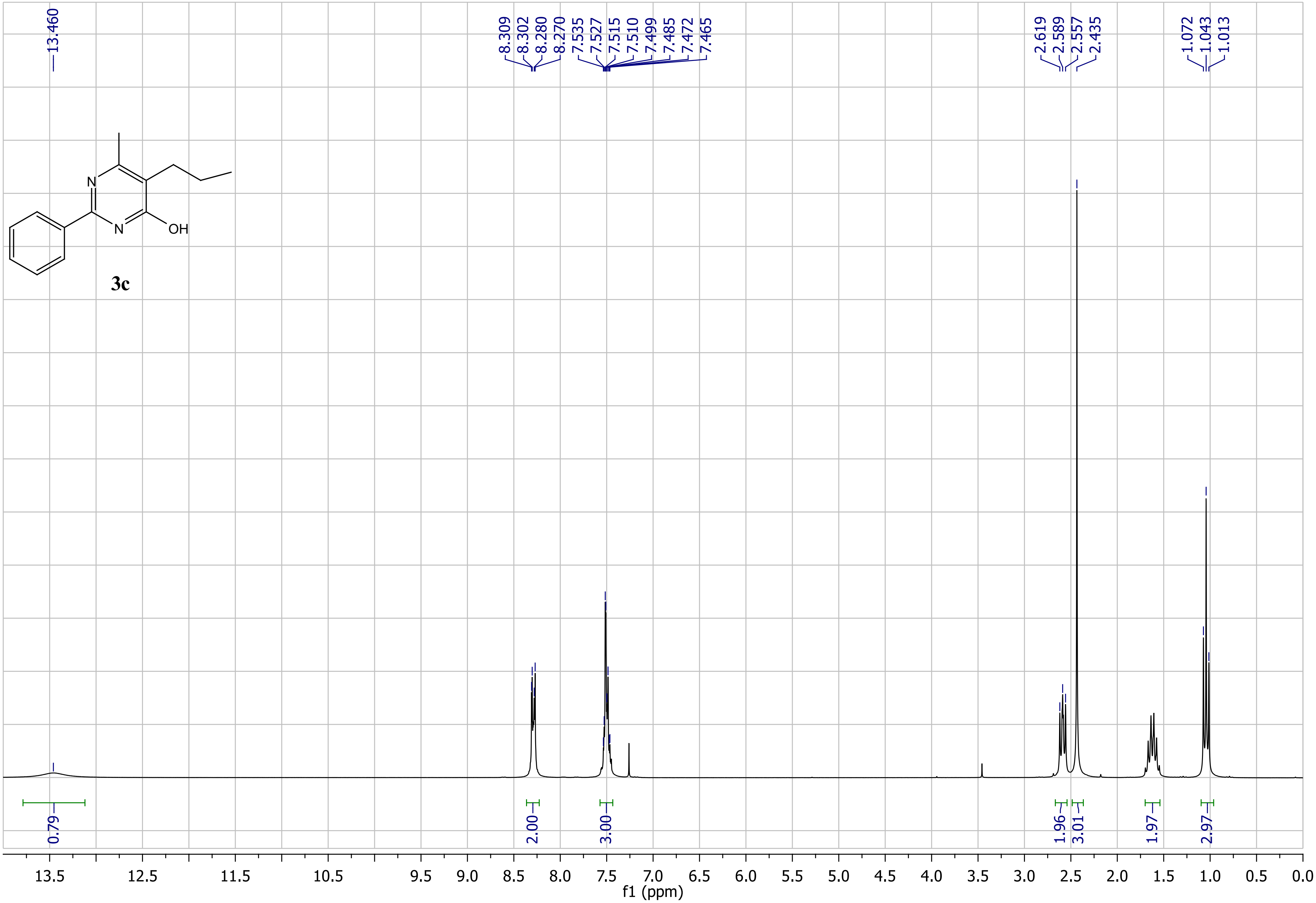
13.460
8.309
8.302
8.280
8.270
7.535
7.527
7.515
7.510
7.499
7.485
7.472
7.465
2.619
2.589
2.557
2.435
1.072
1.043
1.013
N
N
OH
3c
0.79
2.00
3.00
1.96
3.01
1.97
2.97
13.5 12.5 11.5 10.5 9.5 9.0 8.5 8.0 7.5 7.0 6.5 6.0 5.5 5.0 4.5 4.0 3.5 3.0 2.5 2.0 1.5 1.0 0.5 0.0
f1 (ppm)

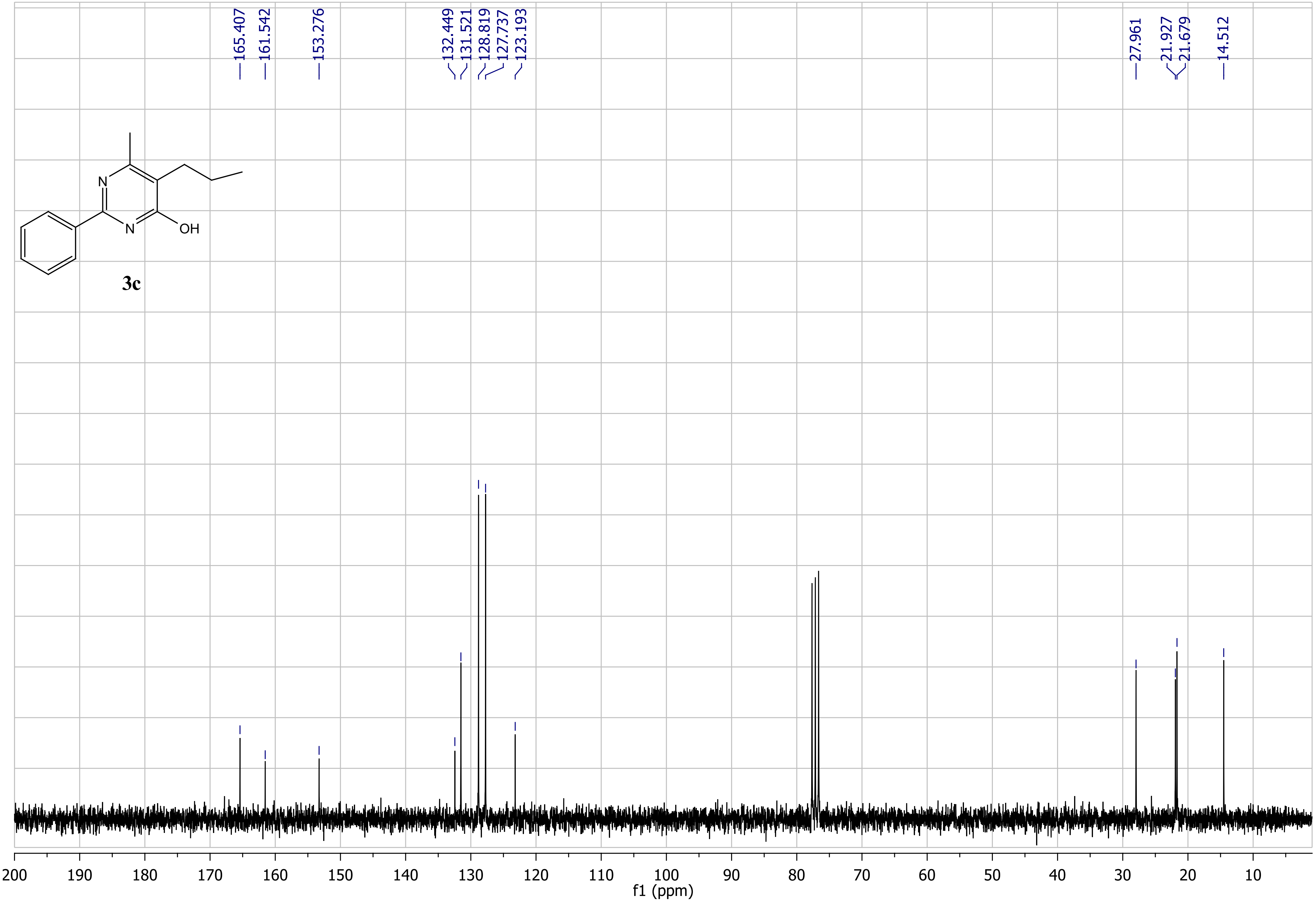

165.407
161.542
153.276
132.449
131.521
128.819
127.737
123.193
27.961
21.927
21.679
14.512
N
N
OH
200
190
180
170
160
150
140
130
120
110
100
90
80
70
60
50
40
30
20
10
f1 (ppm)

3c

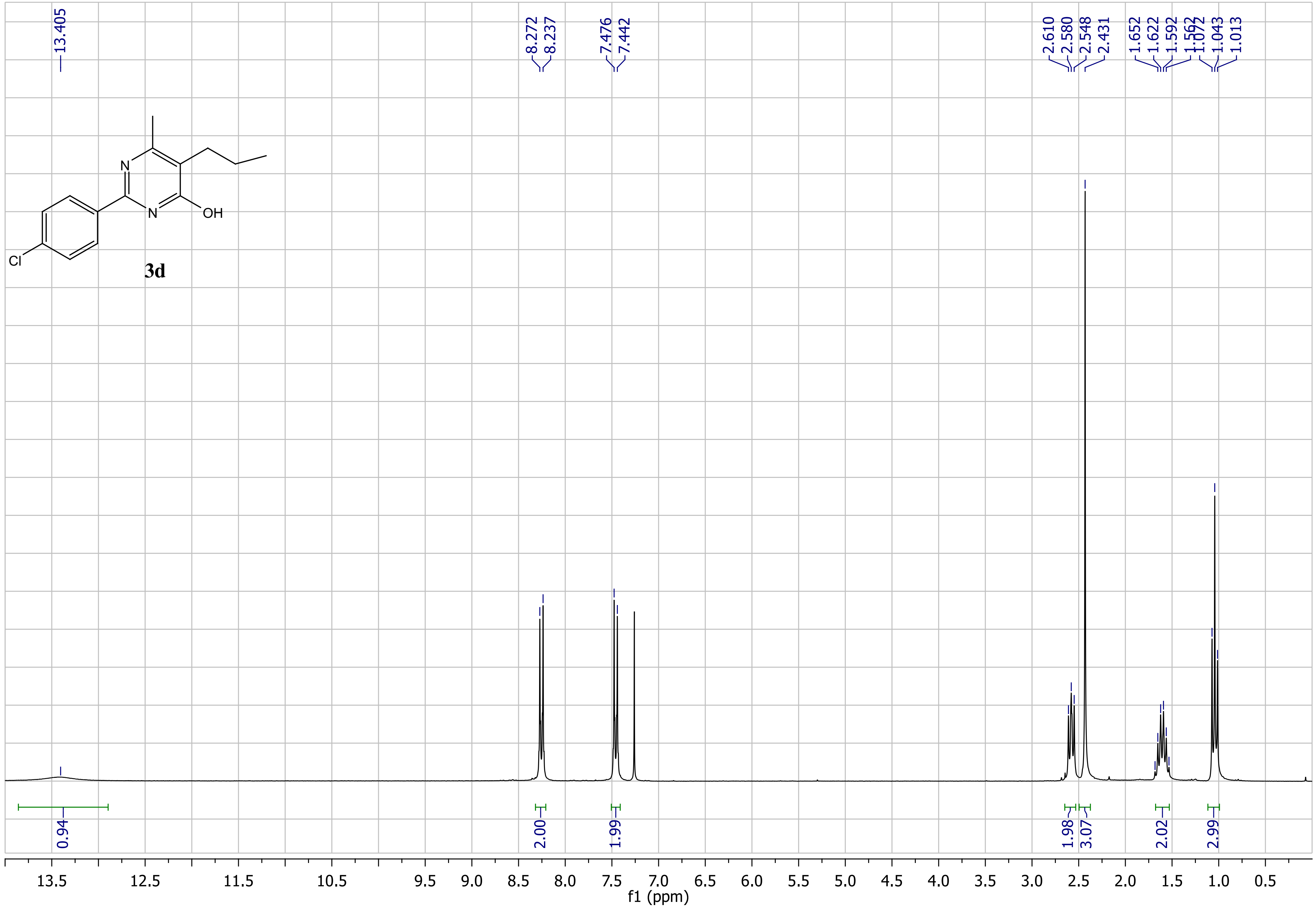
13.405
8.272
8.237
7.476
7.442
2.610
2.580
2.548
2.431
1.652
1.622
1.592
1.562
1.072
1.043
1.013
N
N
OH
Cl
3d
0.94
2.00
1.99
1.98
3.07
2.02
2.99
13.5
12.5
11.5
10.5
9.5
9.0
8.5
8.0
7.5
7.0
6.5
6.0
5.5
5.0
4.5
4.0
3.5
3.0
2.5
2.0
1.5
1.0
0.5
f1 (ppm)

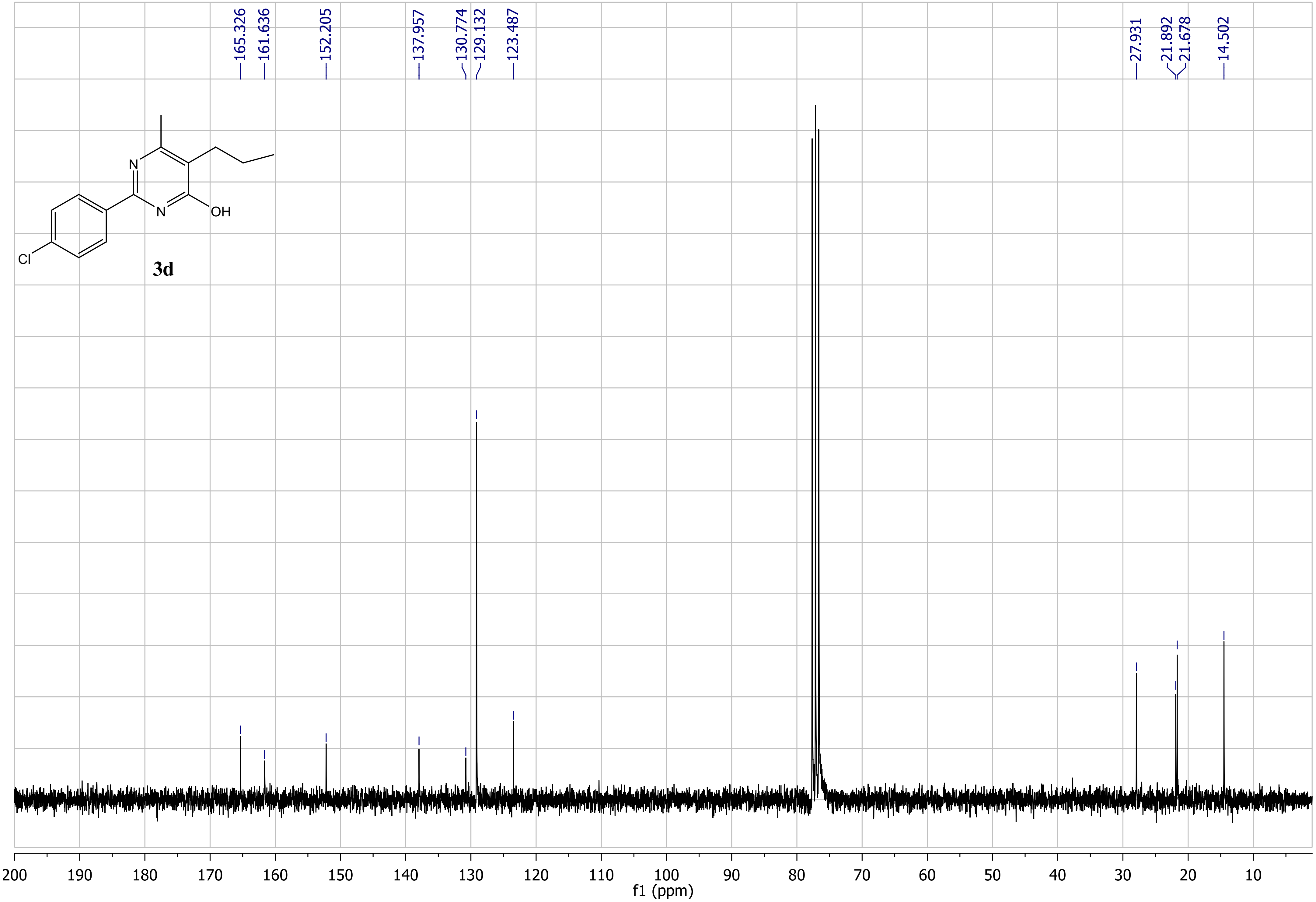
165.326
161.636
152.205
137.957
130.774
129.132
123.487
27.931
21.892
21.678
14.502
N
N
OH
Cl
3d
200 190 180 170 160 150 140 130 120 110 100 90 80 70 60 50 40 30 20 10
f1 (ppm)

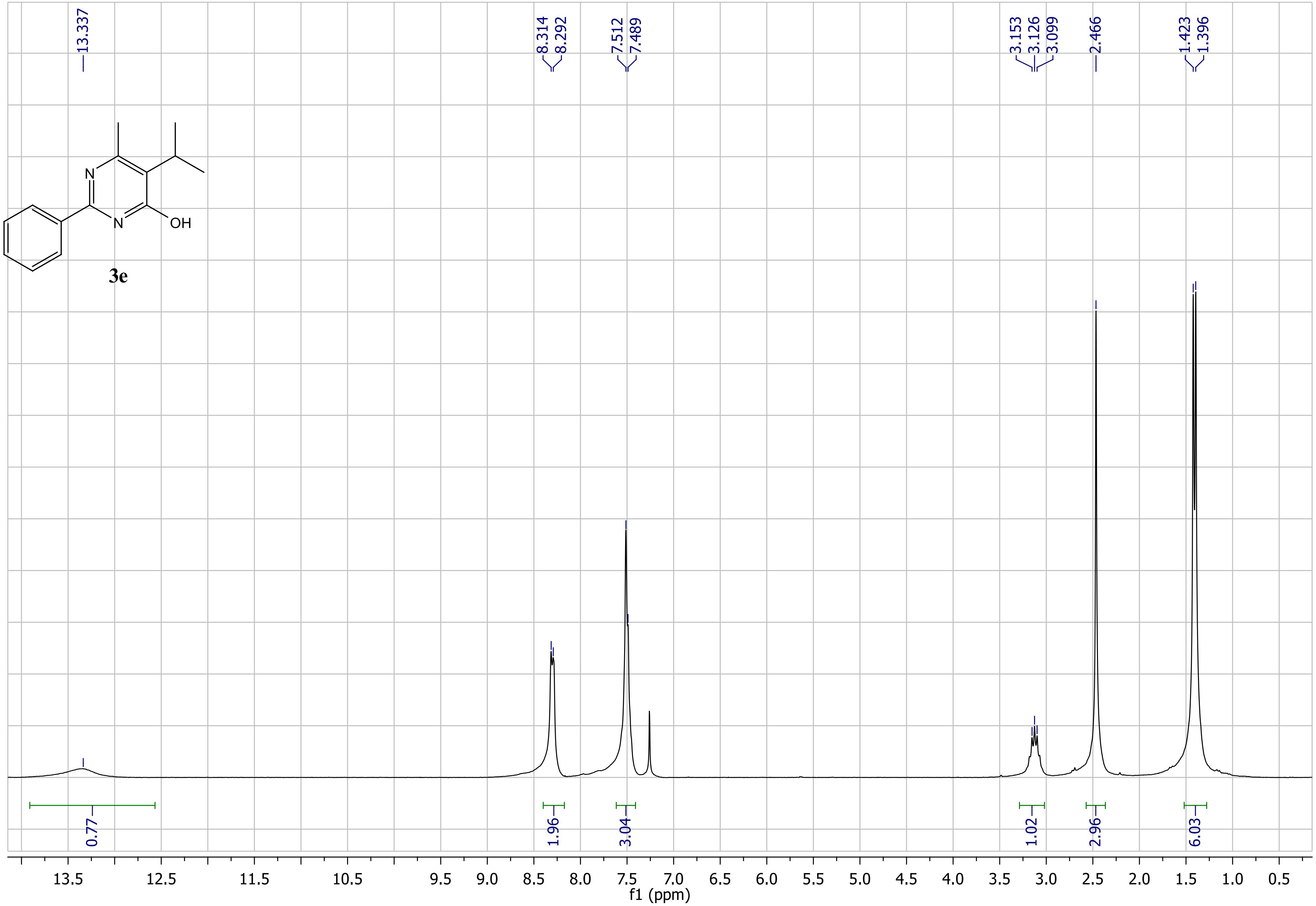
13.337
8.314
8.292
7.512
7.489
3.153
3.126
3.099
2.466
1.423
1.396
N
N
OH
3e
0.77
1.96
3.04
1.02
2.96
6.03
13.5
12.5
11.5
10.5
9.5
9.0
8.5
8.0
7.5
7.0
6.5
6.0
5.5
5.0
4.5
4.0
3.5
3.0
2.5
2.0
1.5
1.0
0.5
f1 (ppm)

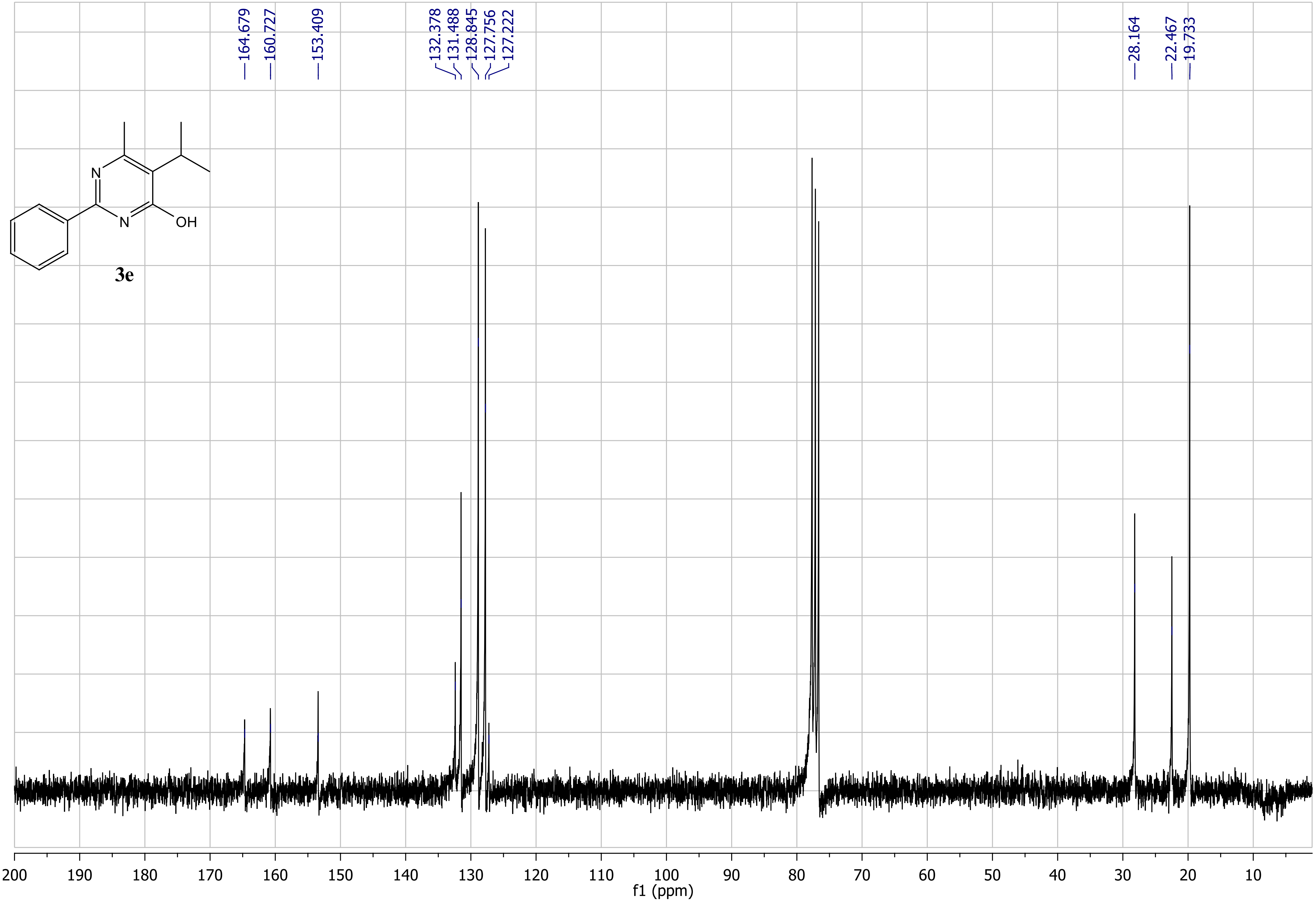
164.679
160.727
153.409
132.378
131.488
128.845
127.756
127.222
28.164
22.467
19.733
N
N
OH
3e
200 190 180 170 160 150 140 130 120 110 100 90 80 70 60 50 40 30 20 10
f1 (ppm)

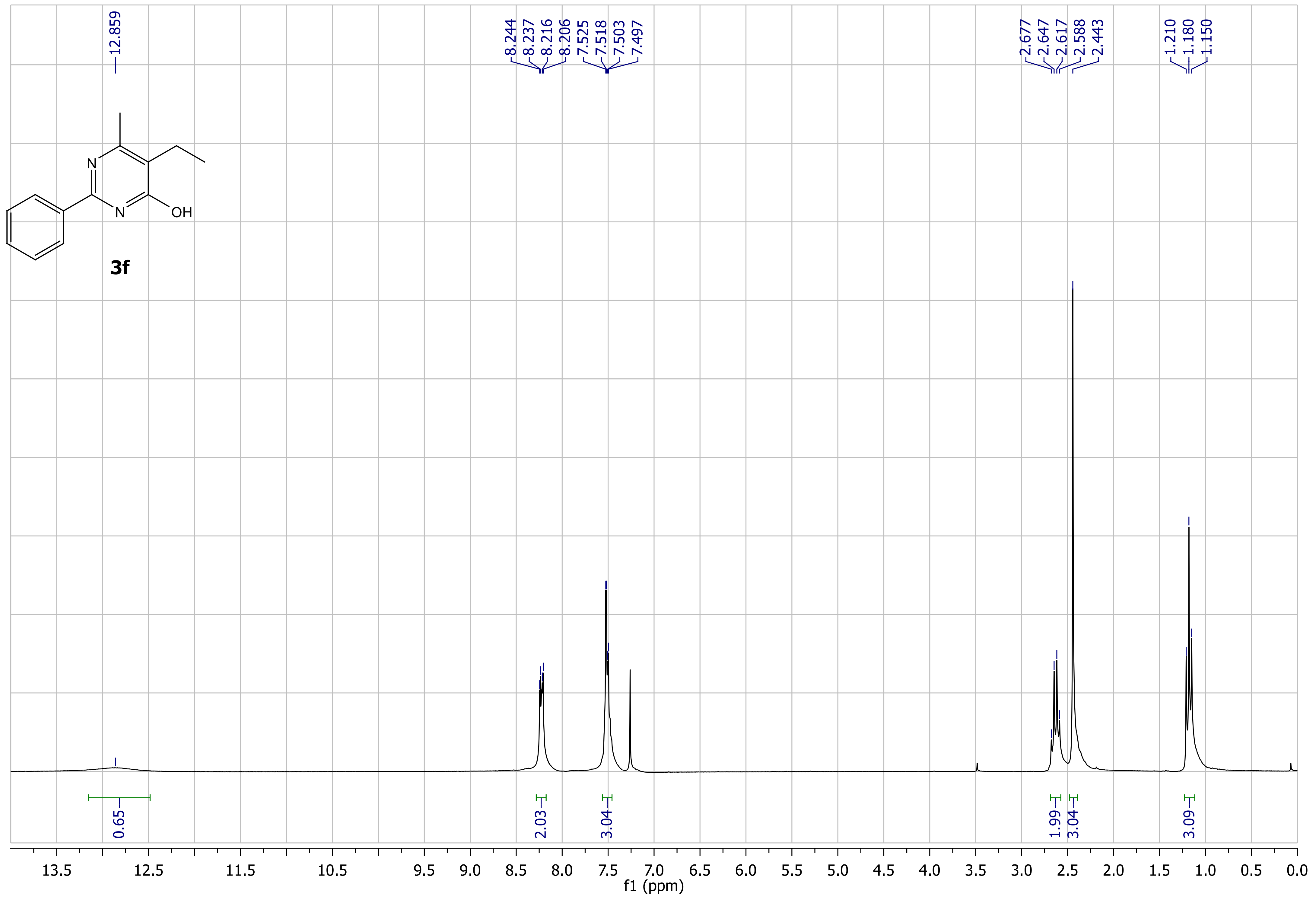

3f
12.859
8.244
8.237
8.216
8.206
7.525
7.518
7.503
7.497
2.677
2.647
2.617
2.588
2.443
1.210
1.180
1.150
0.65
2.03
3.04
1.99
3.04
3.09
13.5
12.5
11.5
10.5
9.5
9.0
8.5
8.0
7.5
7.0
6.5
6.0
5.5
5.0
4.5
4.0
3.5
3.0
2.5
2.0
1.5
1.0
0.5
0.0
f1 (ppm)

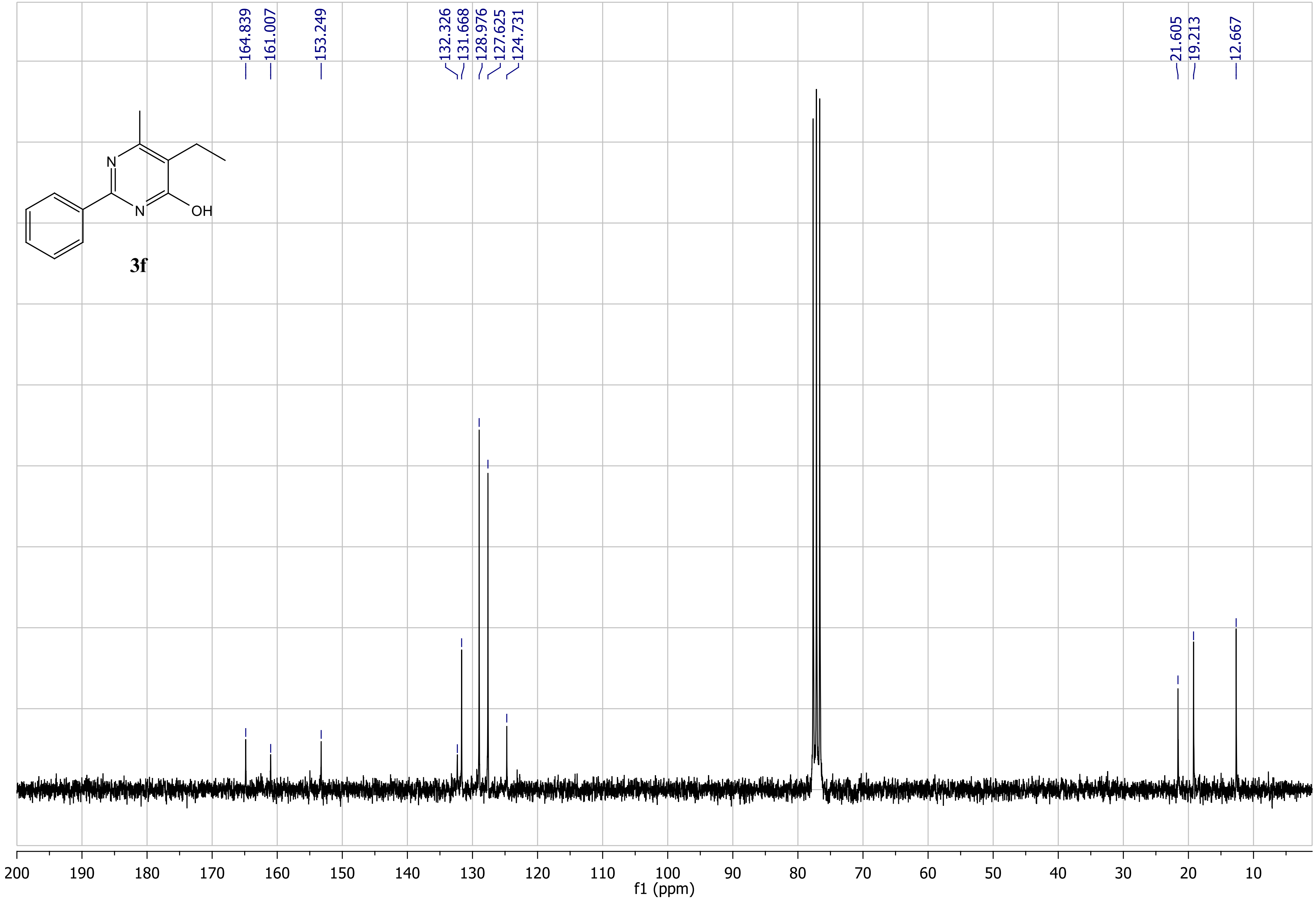
164.839
161.007
153.249
132.326
131.668
128.976
127.625
124.731
21.605
19.213
12.667
N
N
OH
3f
200 190 180 170 160 150 140 130 120 110 100 90 80 70 60 50 40 30 20 10
f1 (ppm)

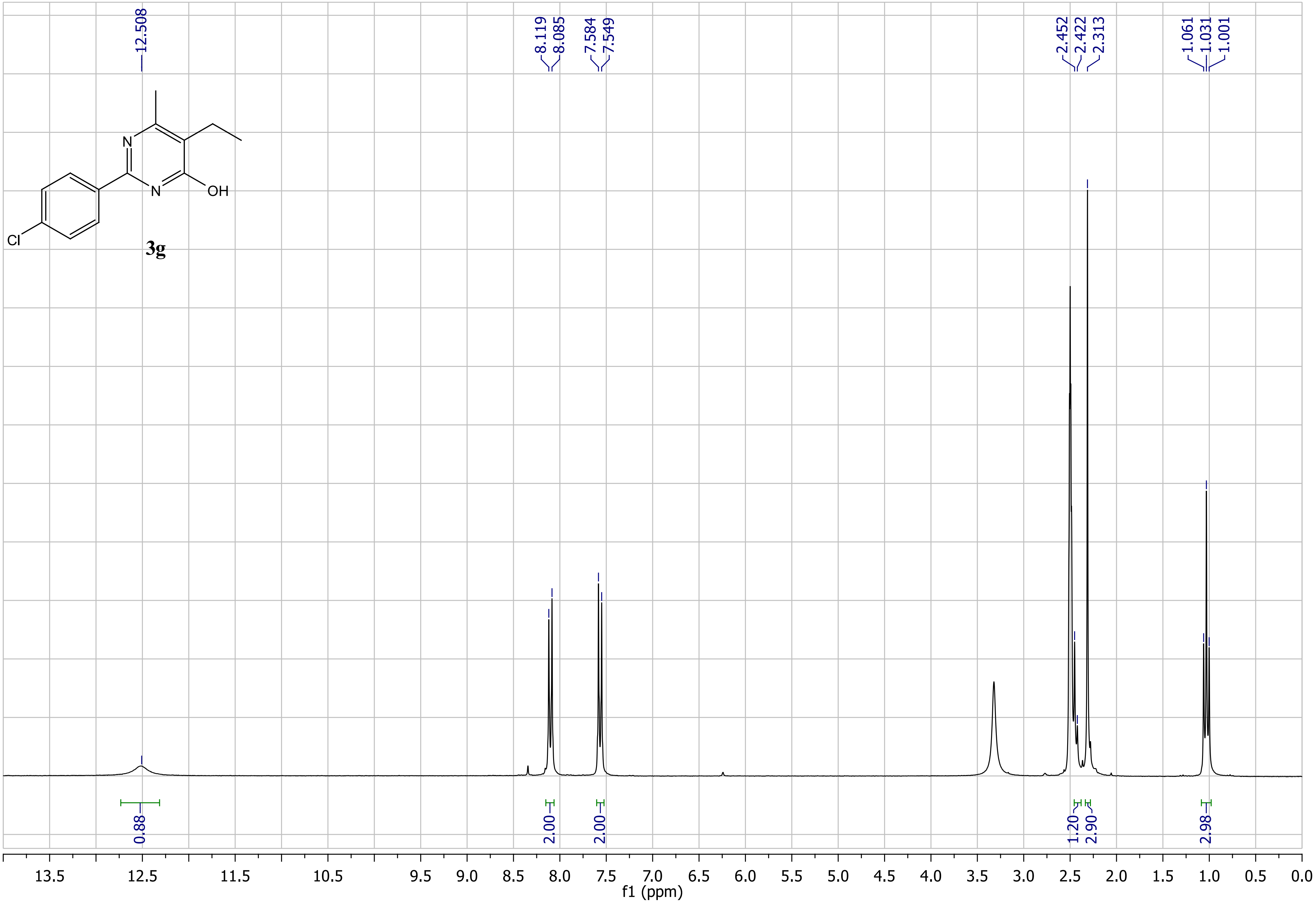
12.508
8.119
8.085
7.584
7.549
2.452
2.422
2.313
1.061
1.031
1.001
N
N
OH
Cl
3g
0.88
2.00
2.00
1.20
2.90
2.98
13.5 12.5 11.5 10.5 9.5 9.0 8.5 8.0 7.5 7.0 6.5 6.0 5.5 5.0 4.5 4.0 3.5 3.0 2.5 2.0 1.5 1.0 0.5 0.0
f1 (ppm)

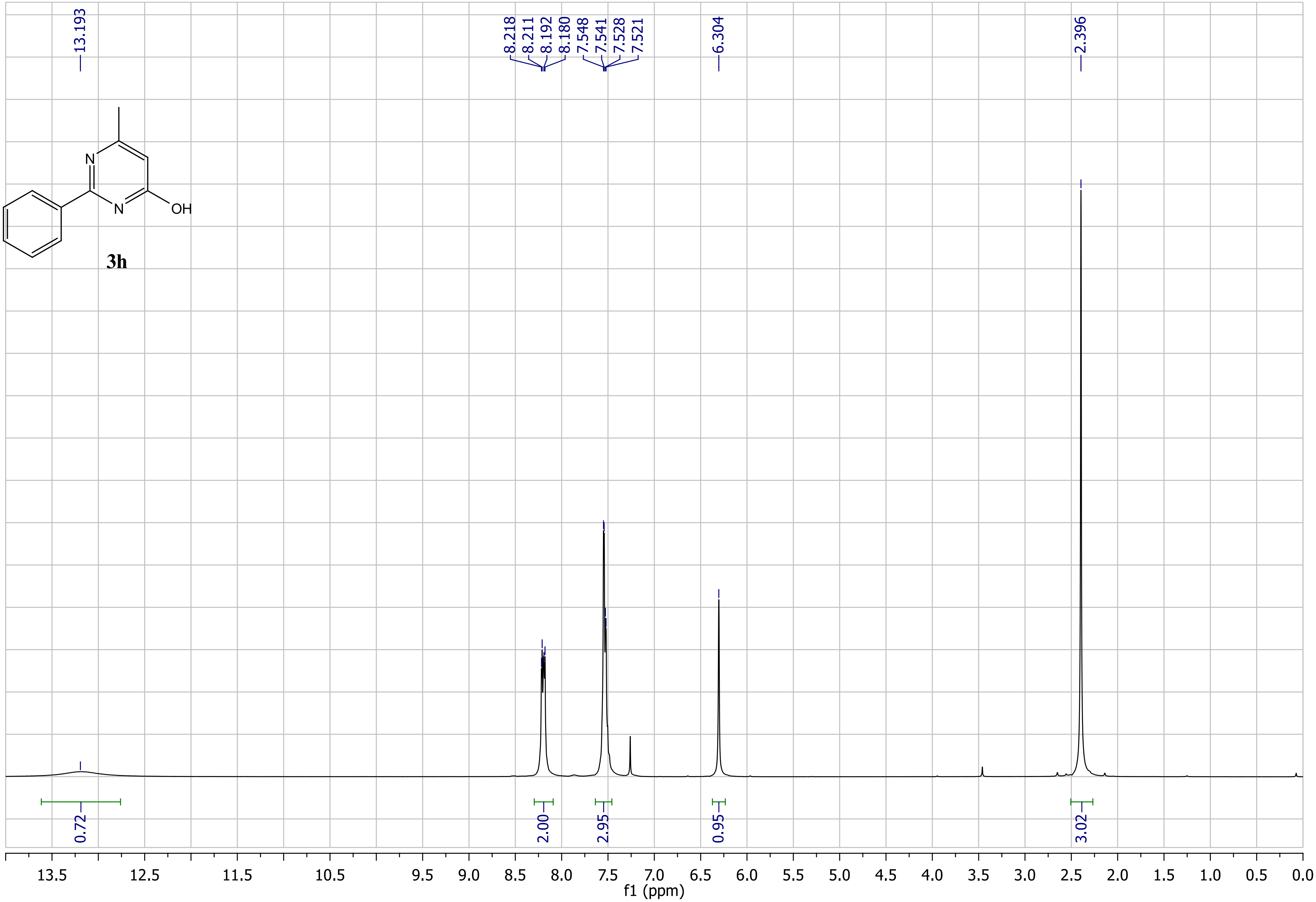
13.193
8.218
8.211
8.192
8.180
7.548
7.541
7.528
7.521
6.304
2.396
N
N
OH
3h
0.72
2.00
2.95
0.95
3.02
13.5
12.5
11.5
10.5
9.5
9.0
8.5
8.0
7.5
7.0
6.5
6.0
5.5
5.0
4.5
4.0
3.5
3.0
2.5
2.0
1.5
1.0
0.5
0.0
f1 (ppm)

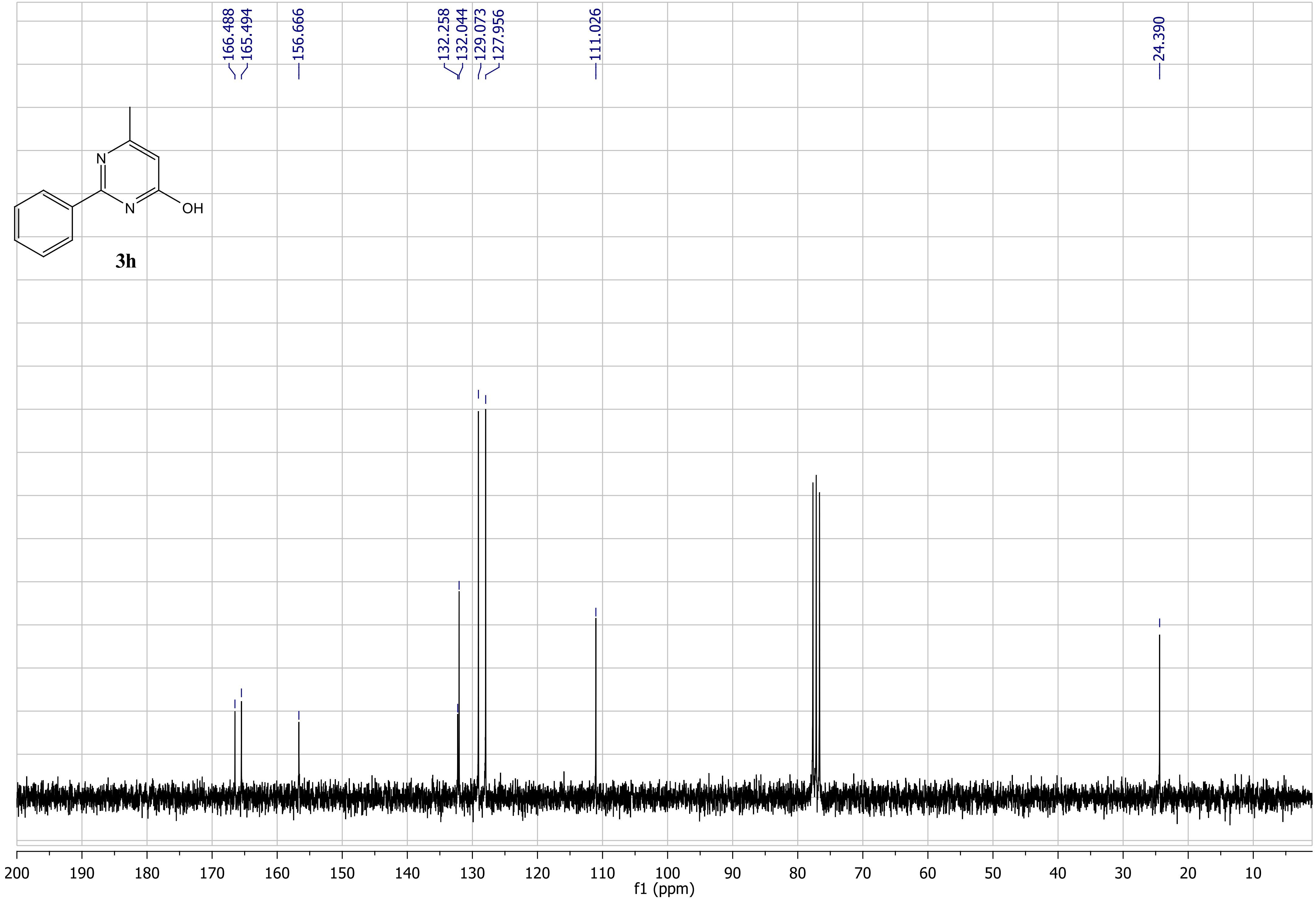
166.488
165.494
156.666
132.258
132.044
129.073
127.956
111.026
24.390
N
N
OH
3h
200 190 180 170 160 150 140 130 120 110 100 90 80 70 60 50 40 30 20 10
f1 (ppm)

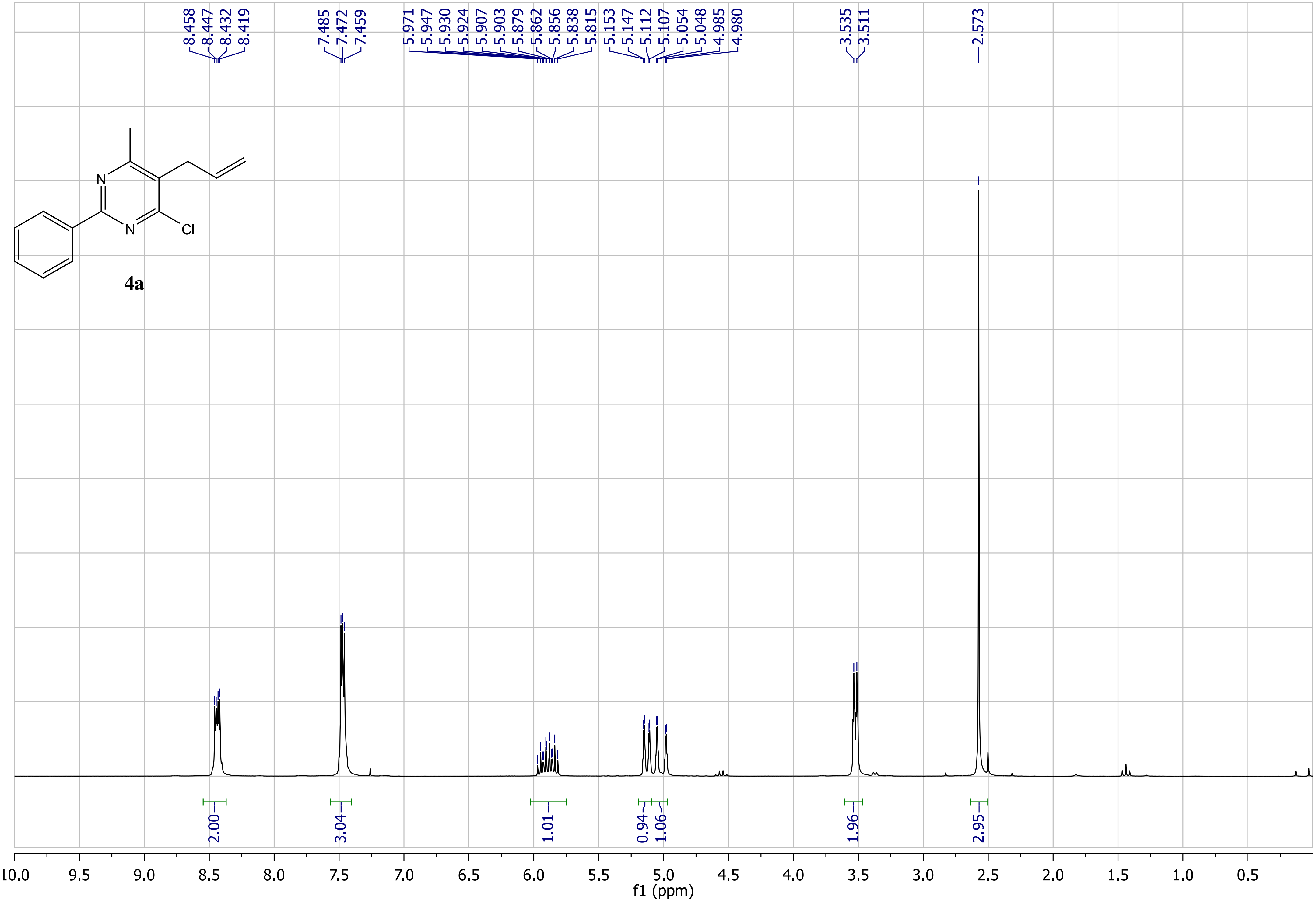
8.458
8.447
8.432
8.419
7.485
7.472
7.459
5.971
5.947
5.930
5.924
5.907
5.903
5.879
5.862
5.856
5.838
5.815
5.153
5.147
5.112
5.107
5.054
5.048
4.985
4.980
3.535
3.511
2.573
N
N
Cl
4a
2.00
3.04
1.01
0.94
1.06
1.96
2.95
10.0
9.5
9.0
8.5
8.0
7.5
7.0
6.5
6.0
5.5
5.0
4.5
4.0
3.5
3.0
2.5
2.0
1.5
1.0
0.5
f1 (ppm)

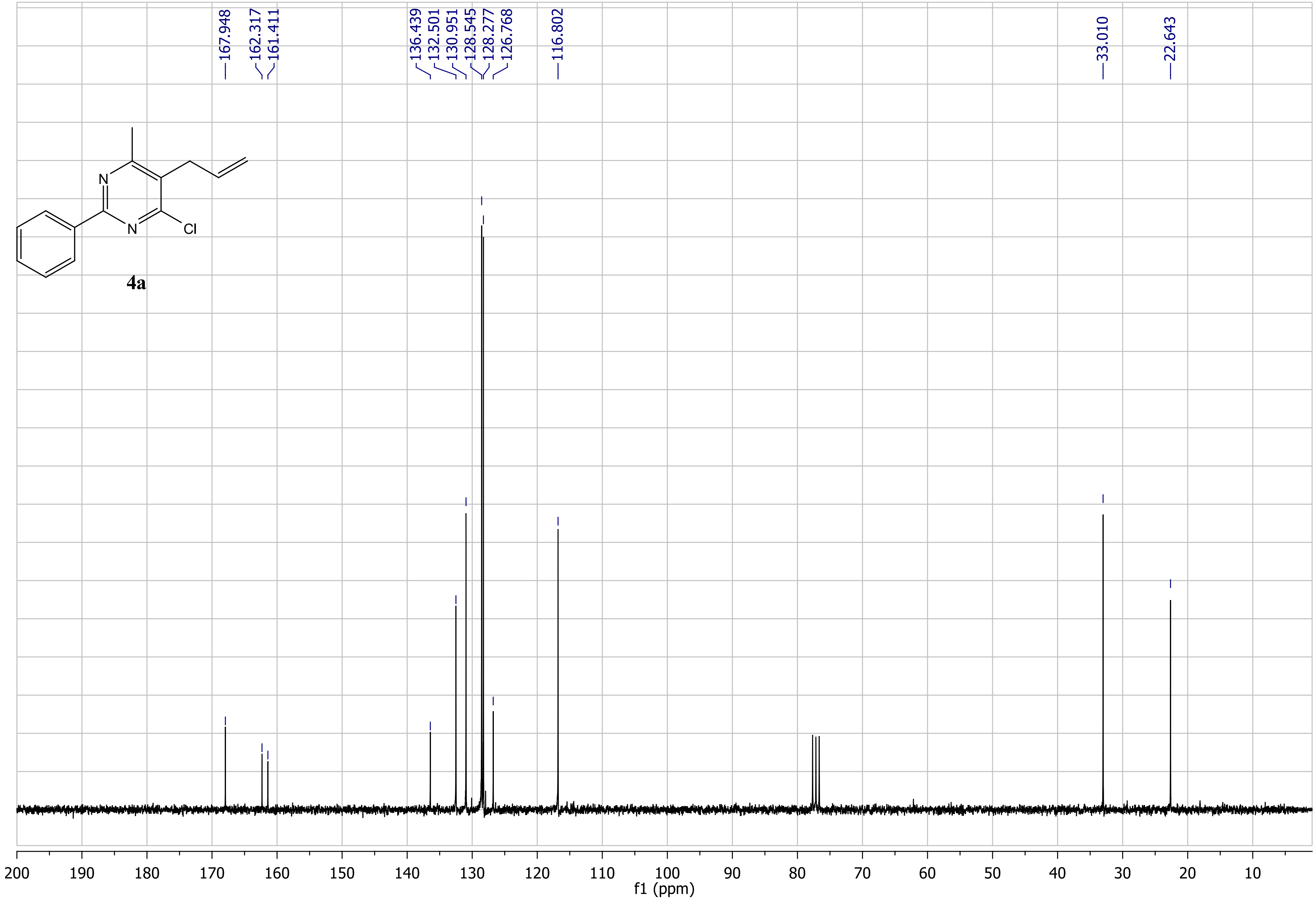
167.948
162.317
161.411
136.439
132.501
130.951
128.545
128.277
126.768
116.802
33.010
22.643
N
N
Cl
4a
200
190
180
170
160
150
140
130
120
110
100
90
80
70
60
50
40
30
20
10
f1 (ppm)

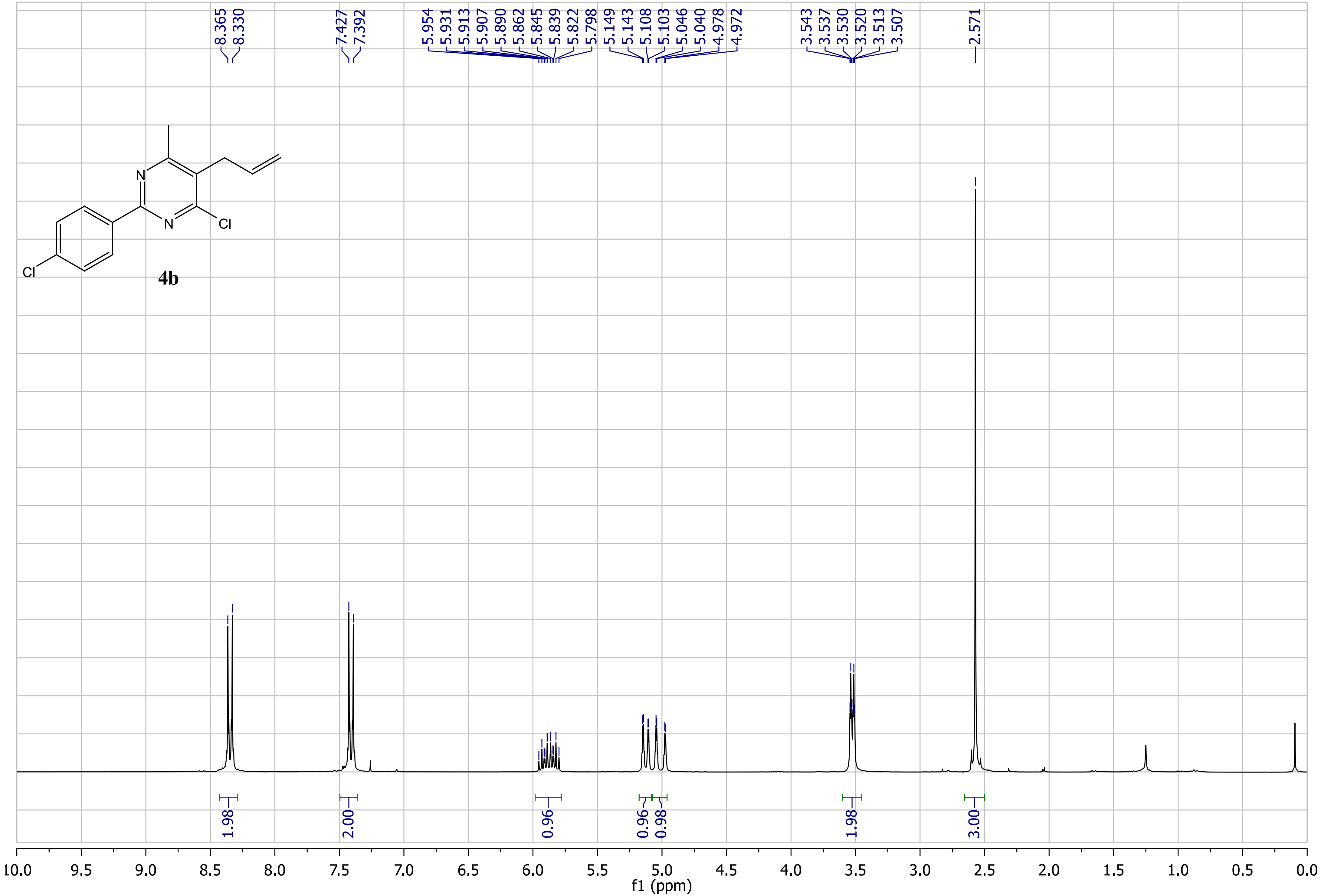
8.365
8.330
7.427
7.392
5.954
5.931
5.913
5.907
5.890
5.862
5.845
5.839
5.822
5.798
5.149
5.143
5.108
5.103
5.046
5.040
4.978
4.972
3.543
3.537
3.530
3.520
3.513
3.507
2.571
N
N
Cl
Cl
4b
1.98
2.00
0.96
0.96
0.98
1.98
3.00
10.0
9.5
9.0
8.5
8.0
7.5
7.0
6.5
6.0
5.5
5.0
4.5
4.0
3.5
3.0
2.5
2.0
1.5
1.0
0.5
0.0
f1 (ppm)

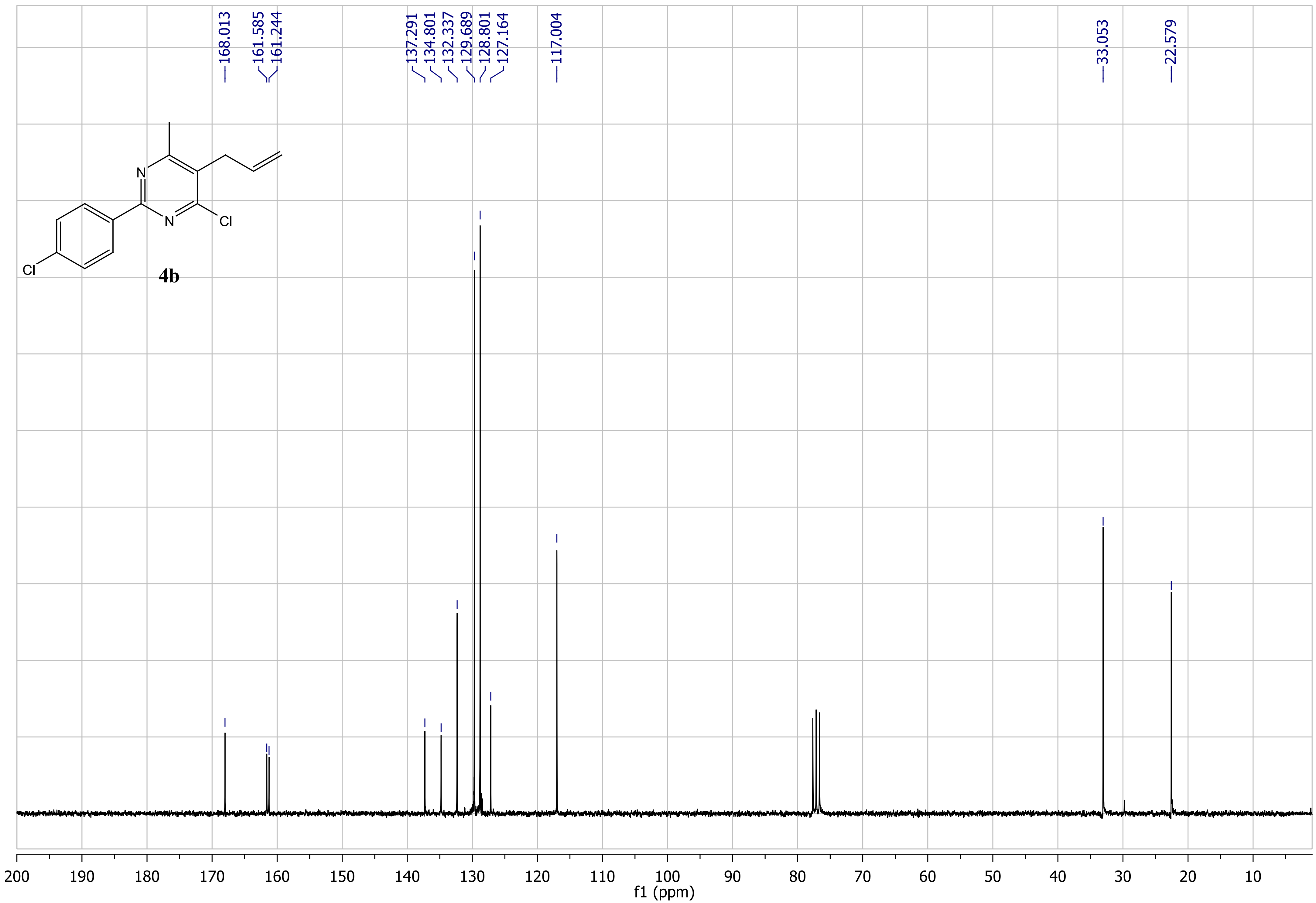
168.013
161.585
161.244
137.291
134.801
132.337
129.689
128.801
127.164
117.004
33.053
22.579
N
N
Cl
Cl
4b
200 190 180 170 160 150 140 130 120 110 100 90 80 70 60 50 40 30 20 10
f1 (ppm)

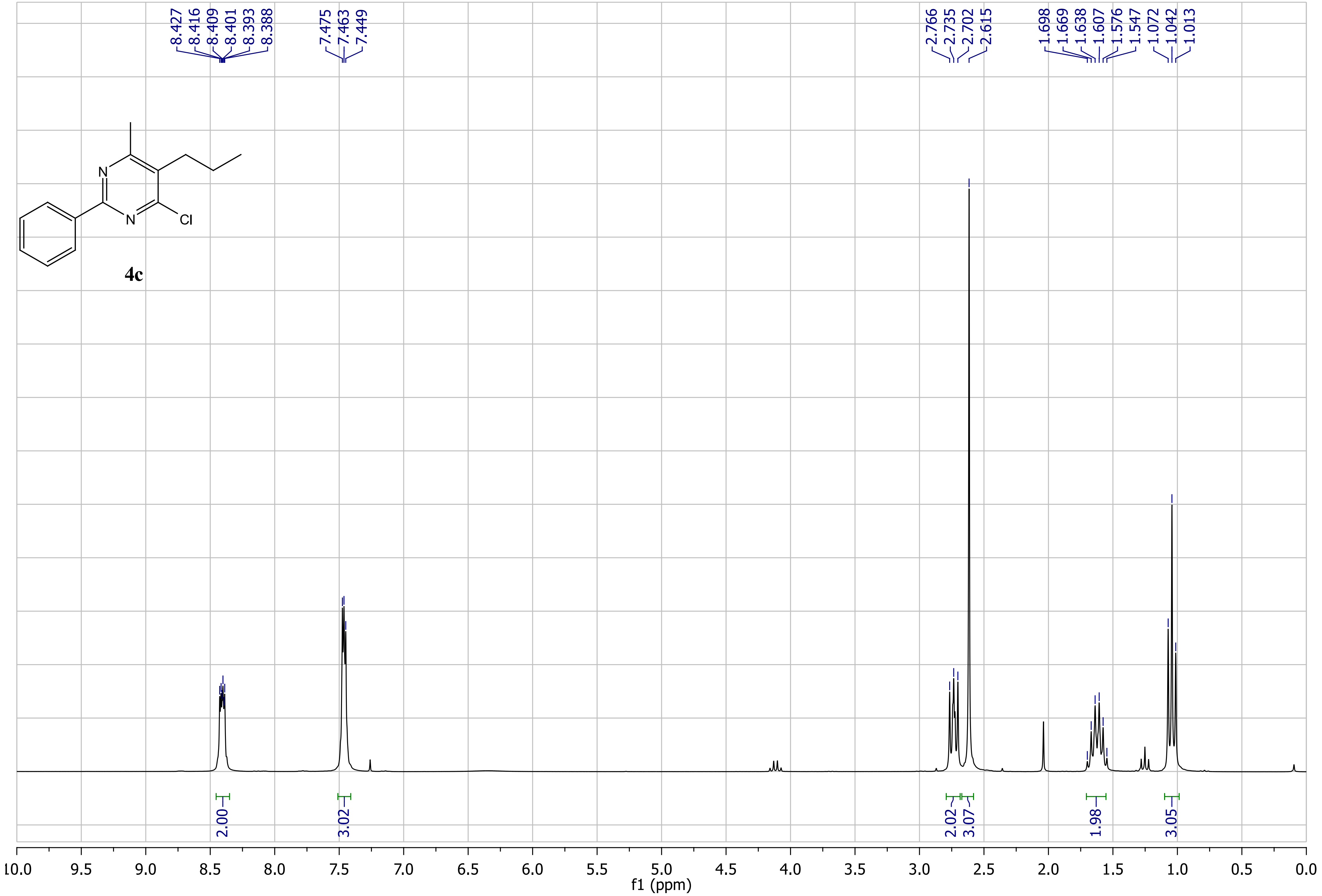
8.427
8.416
8.409
8.401
8.393
8.388
7.475
7.463
7.449
2.766
2.735
2.702
2.615
1.698
1.669
1.638
1.607
1.576
1.547
1.072
1.042
1.013
N
N
Cl
4c
2.00
3.02
2.02
3.07
1.98
3.05
10.0
9.5
9.0
8.5
8.0
7.5
7.0
6.5
6.0
5.5
5.0
4.5
4.0
3.5
3.0
2.5
2.0
1.5
1.0
0.5
0.0
f1 (ppm)

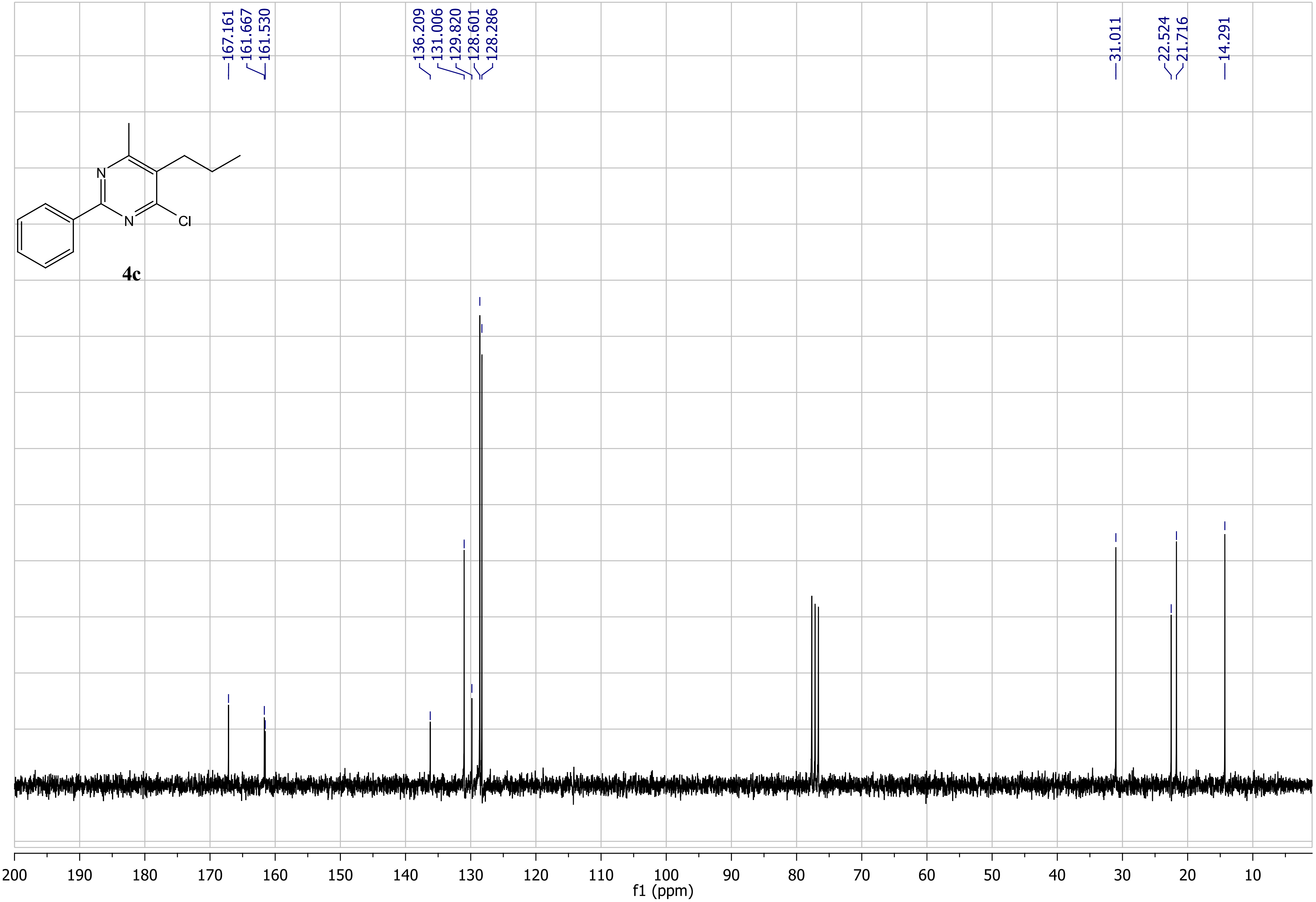

167.161
161.667
161.530
136.209
131.006
129.820
128.601
128.286
31.011
22.524
21.716
14.291
N
N
Cl
4c
200 190 180 170 160 150 140 130 120 110 100 90 80 70 60 50 40 30 20 10
f1 (ppm)

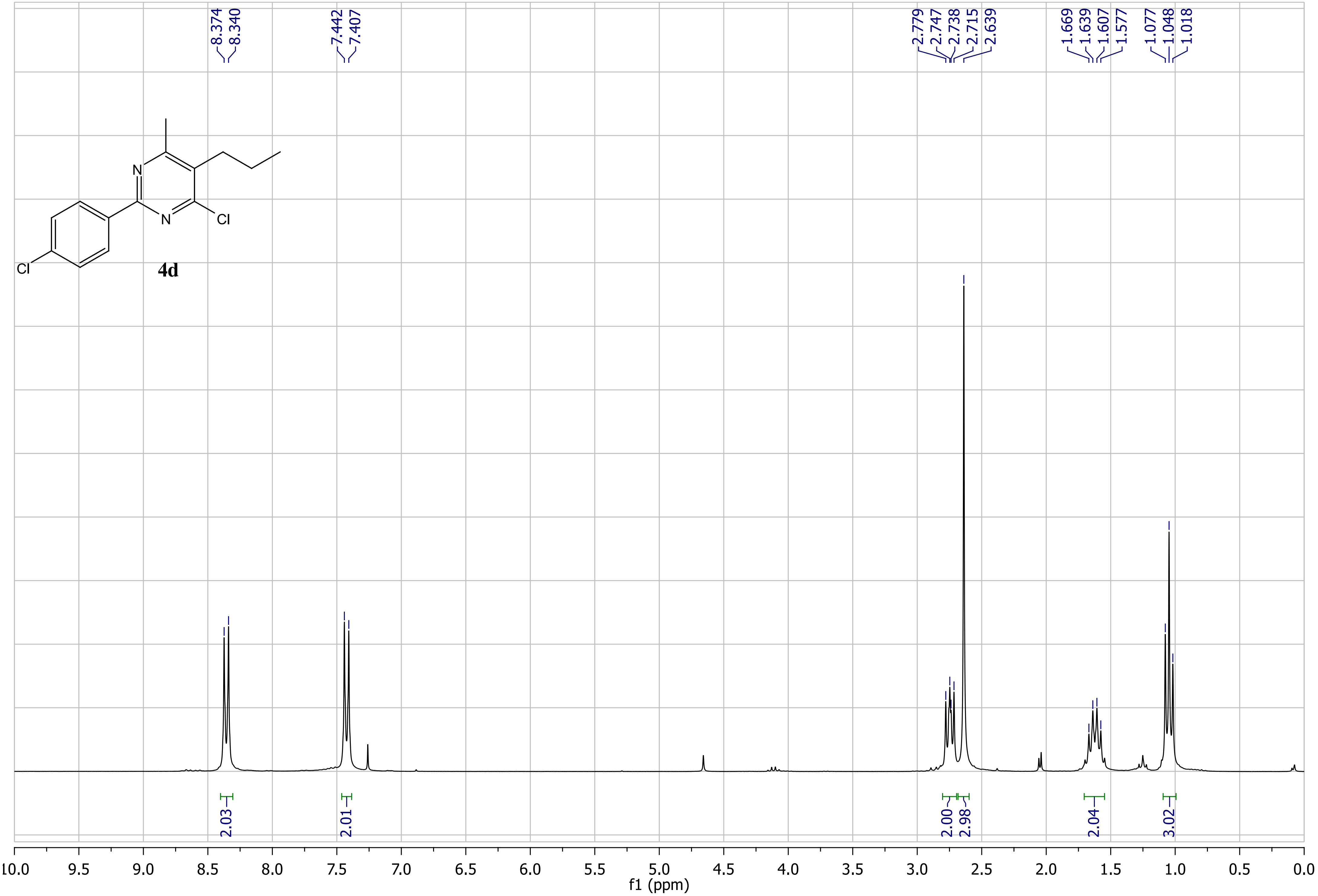
8.374
8.340
7.442
7.407
2.779
2.747
2.738
2.715
2.639
1.669
1.639
1.607
1.577
1.077
1.048
1.018
N
N
Cl
Cl
4d
2.03
2.01
2.00
2.98
2.04
3.02
10.0 9.5 9.0 8.5 8.0 7.5 7.0 6.5 6.0 5.5 5.0 4.5 4.0 3.5 3.0 2.5 2.0 1.5 1.0 0.5 0.0
f1 (ppm)

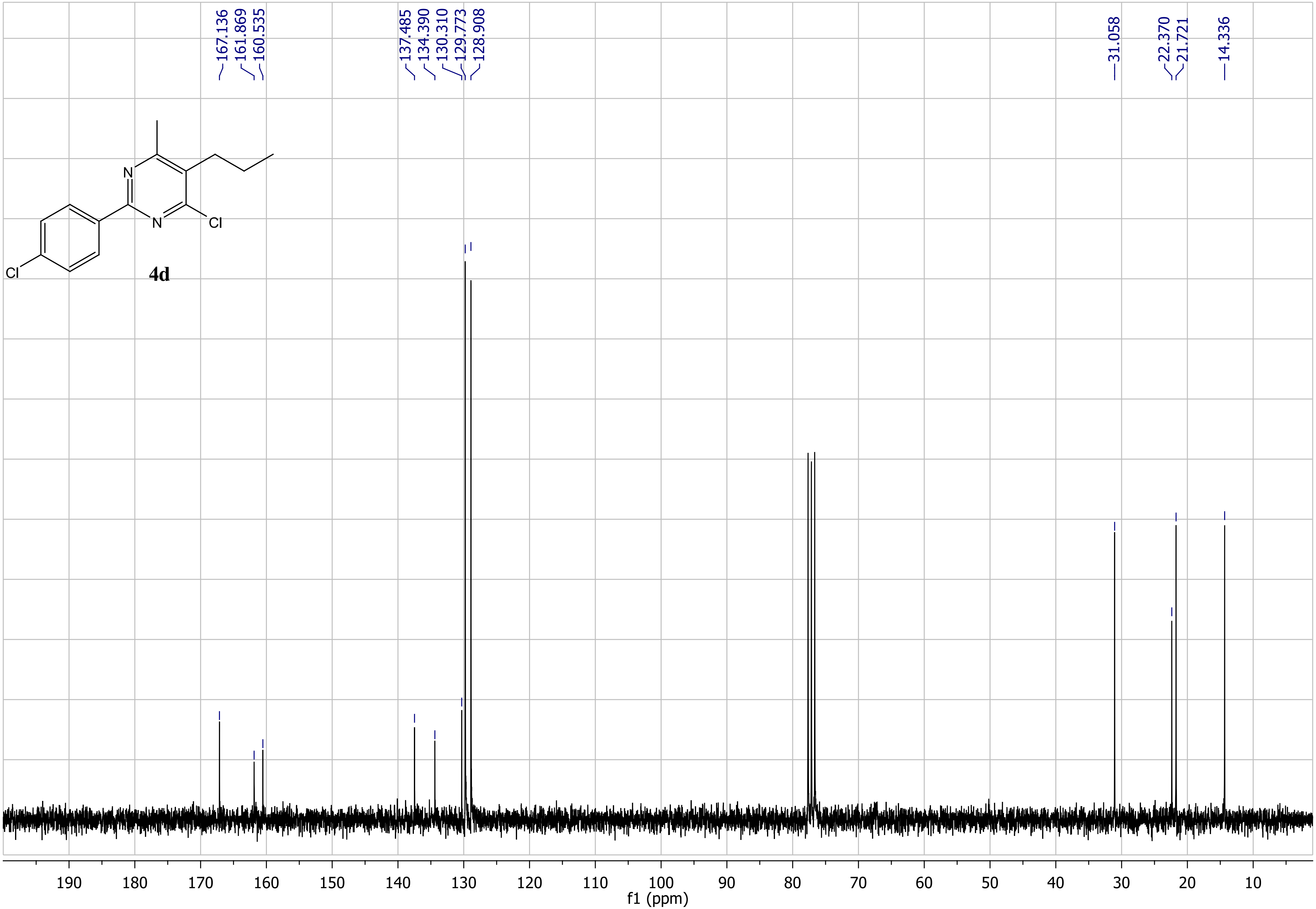
167.136
161.869
160.535
137.485
134.390
130.310
129.773
128.908
31.058
22.370
21.721
14.336
N
N
Cl
Cl
4d
190 180 170 160 150 140 130 120 110 100 90 80 70 60 50 40 30 20 10
f1 (ppm)

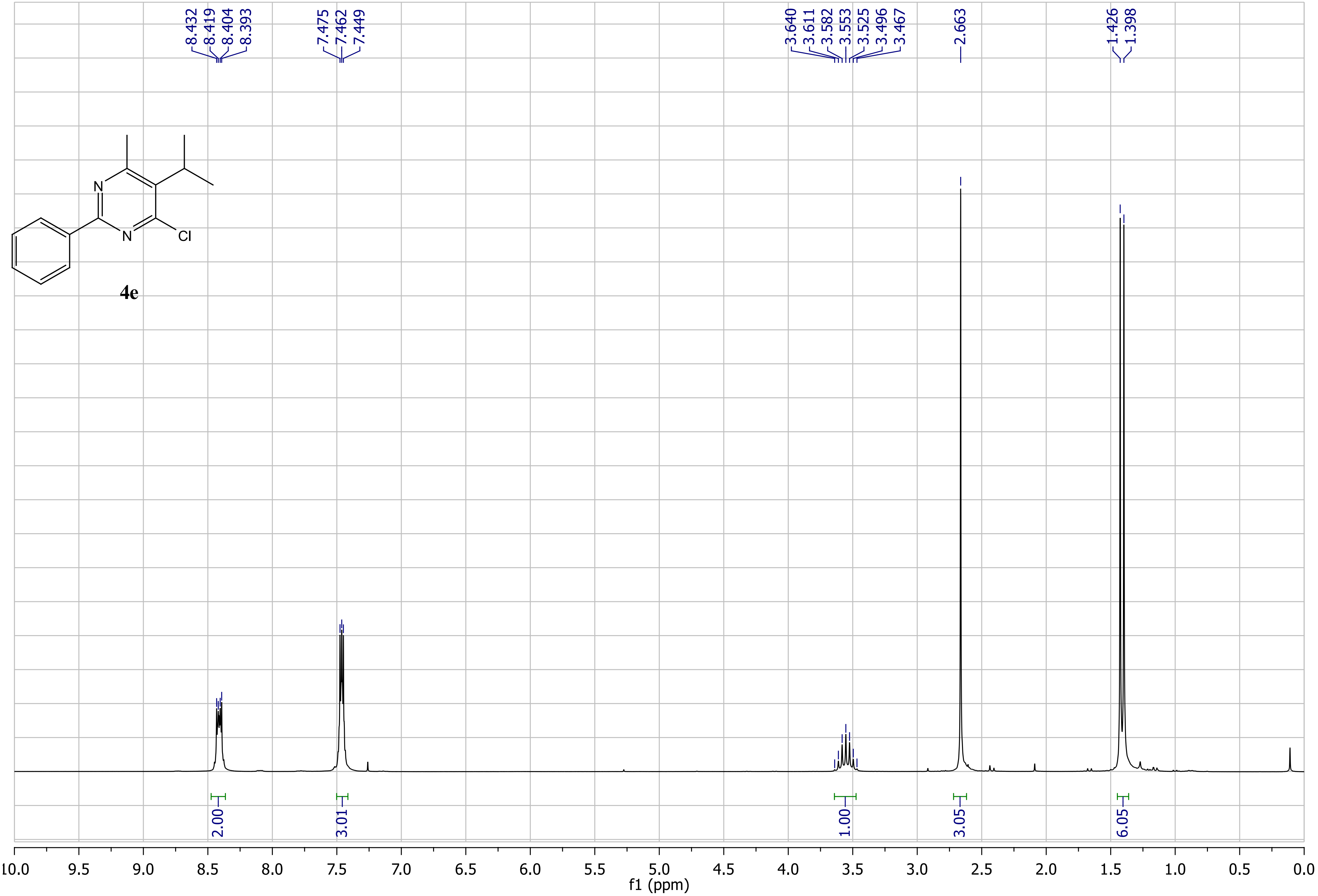
8.432
8.419
8.404
8.393
7.475
7.462
7.449
3.640
3.611
3.582
3.553
3.525
3.496
3.467
2.663
1.426
1.398
N
N
Cl
4e
2.00
3.01
1.00
3.05
6.05
10.0
9.5
9.0
8.5
8.0
7.5
7.0
6.5
6.0
5.5
5.0
4.5
4.0
3.5
3.0
2.5
2.0
1.5
1.0
0.5
0.0
f1 (ppm)

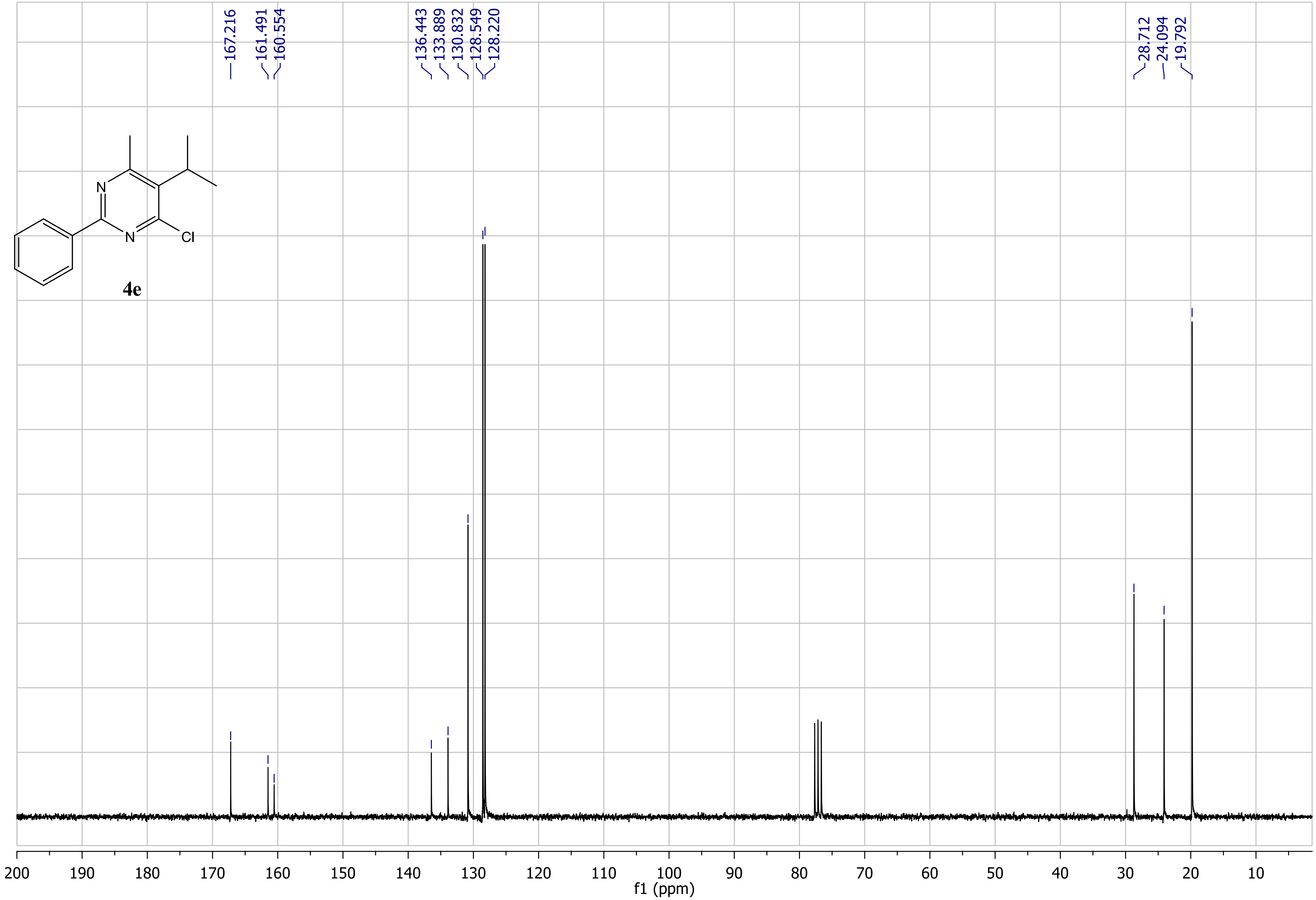
167.216
161.491
160.554
136.443
133.889
130.832
128.549
128.220
28.712
24.094
19.792
N
N
Cl
4e
200 190 180 170 160 150 140 130 120 110 100 90 80 70 60 50 40 30 20 10
f1 (ppm)

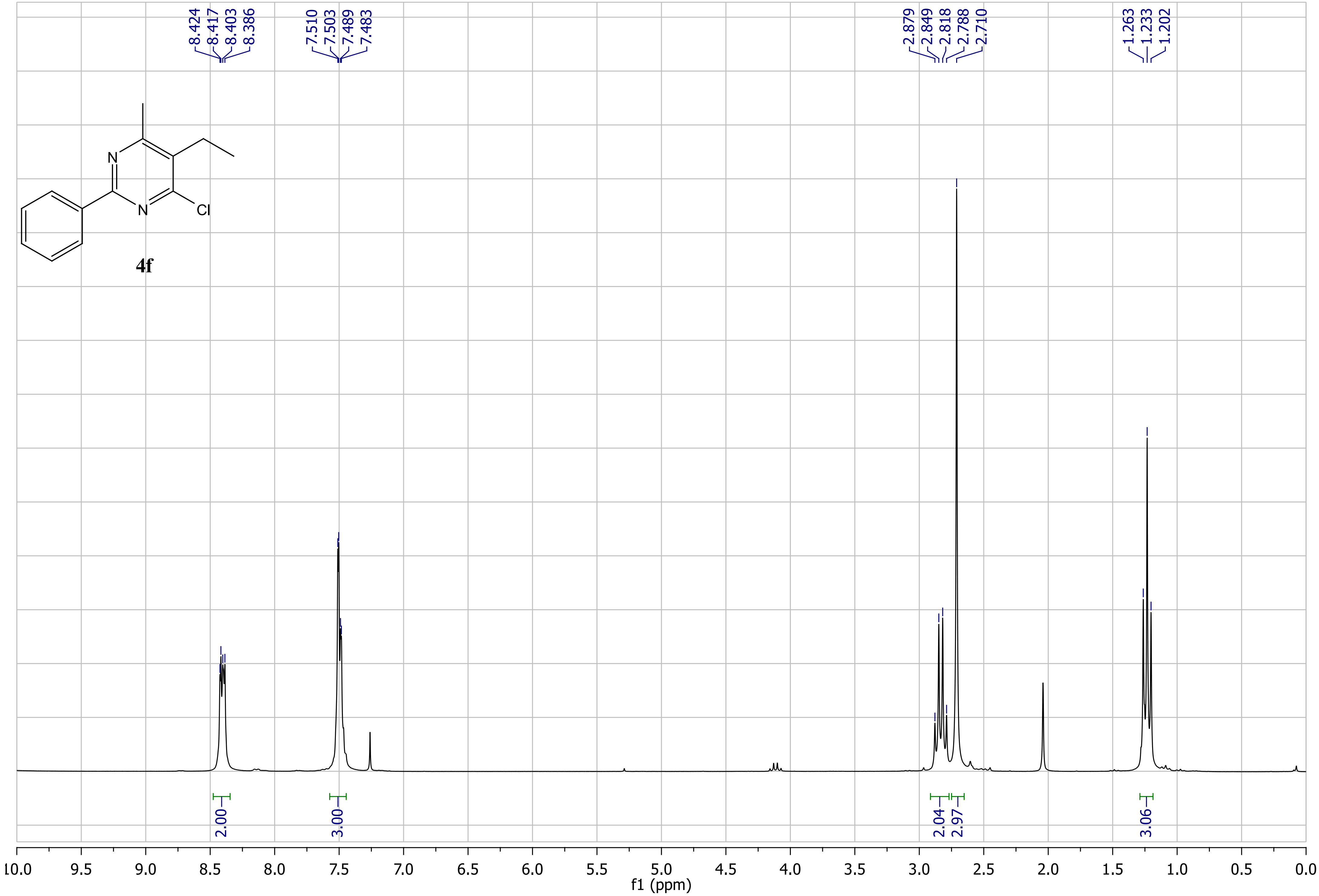
8.424
8.417
8.403
8.386
7.510
7.503
7.489
7.483
2.879
2.849
2.818
2.788
2.710
1.263
1.233
1.202
N
N
Cl
4f
2.00
3.00
2.04
2.97
3.06
10.0 9.5 9.0 8.5 8.0 7.5 7.0 6.5 6.0 5.5 5.0 4.5 4.0 3.5 3.0 2.5 2.0 1.5 1.0 0.5 0.0
f1 (ppm)

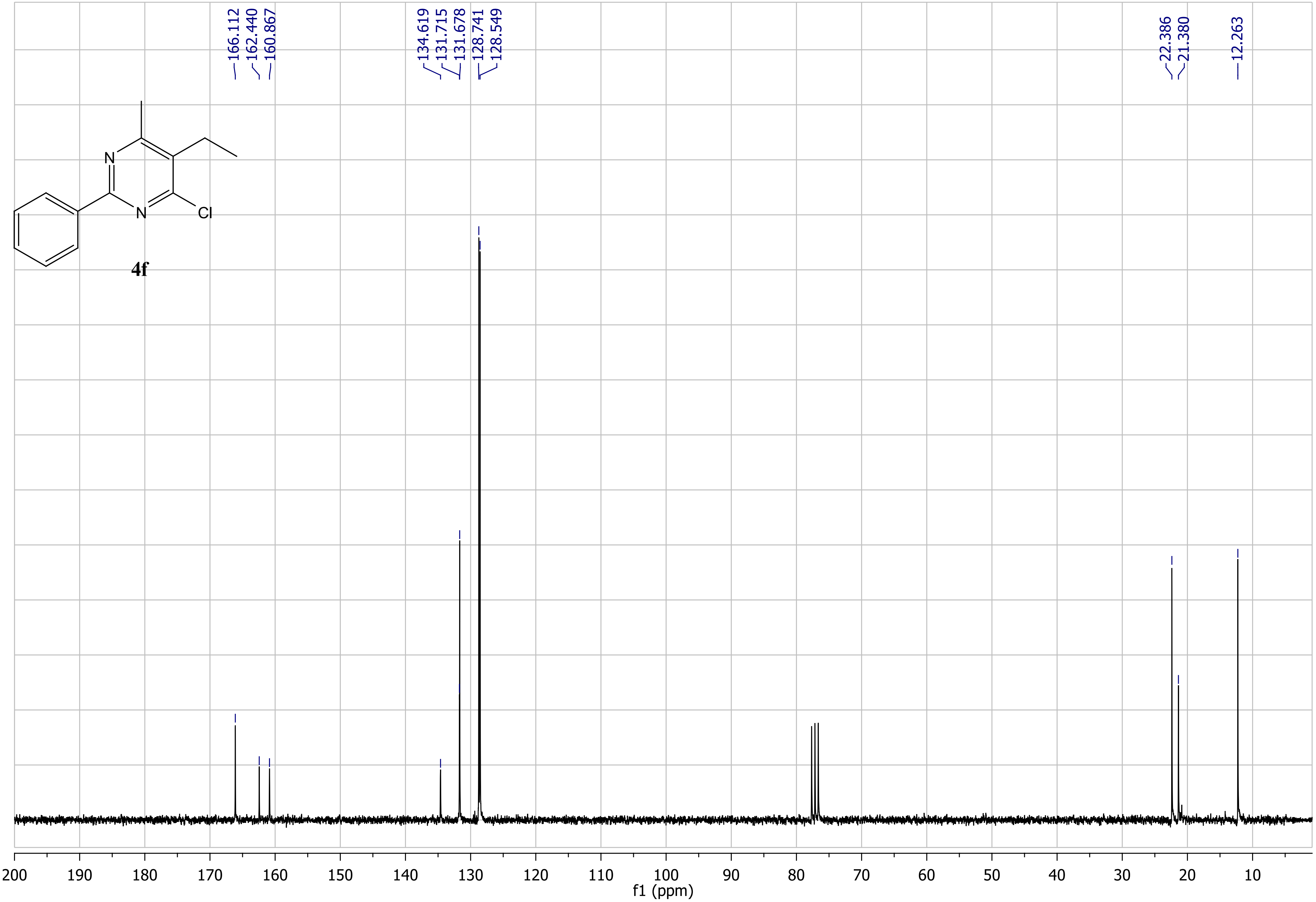
166.112
162.440
160.867
134.619
131.715
131.678
128.741
128.549
22.386
21.380
12.263
N
N
Cl
4f
200 190 180 170 160 150 140 130 120 110 100 90 80 70 60 50 40 30 20 10
f1 (ppm)

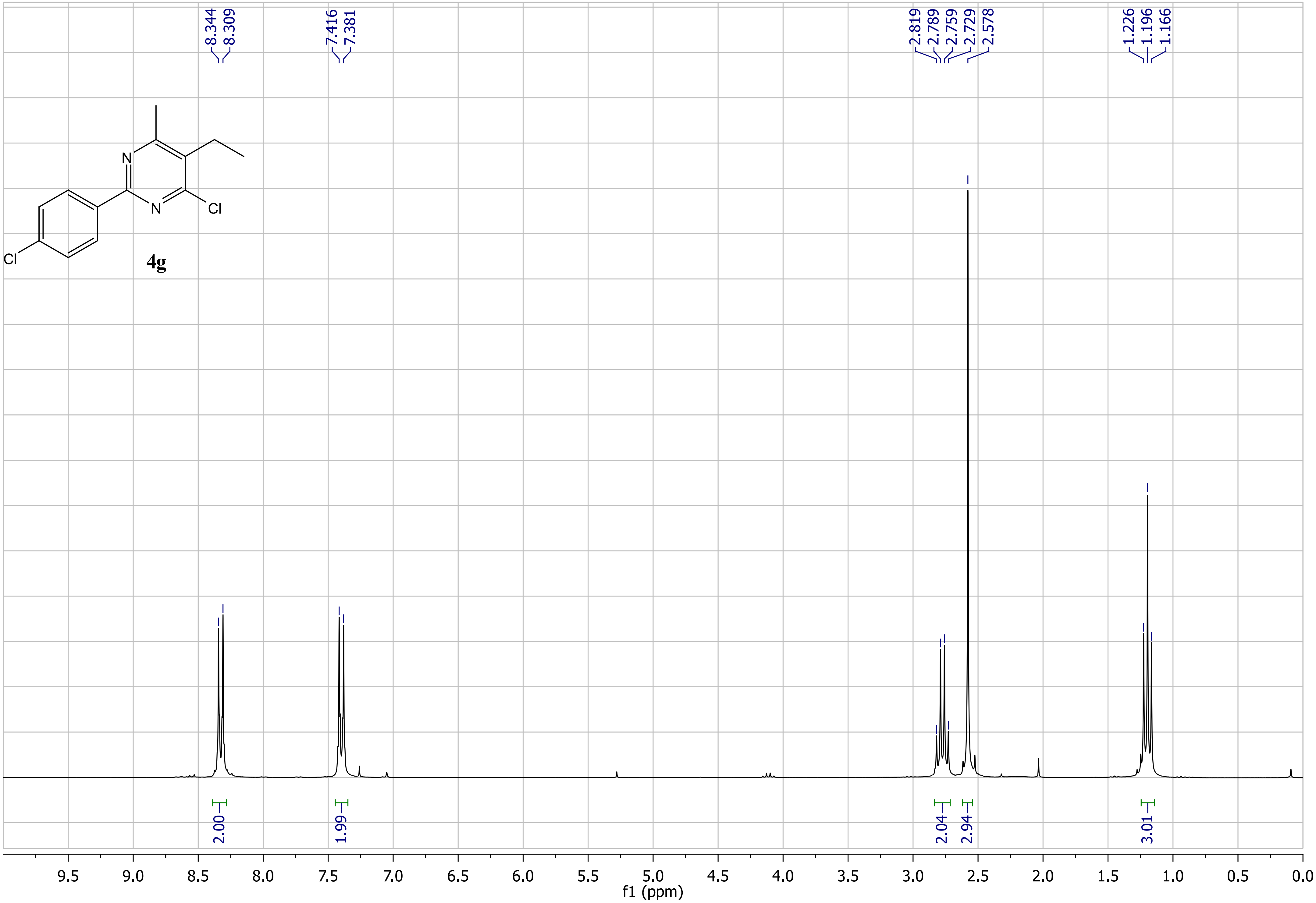

8.344
8.309
7.416
7.381
2.819
2.789
2.759
2.729
2.578
1.226
1.196
1.166
N
N
Cl
Cl
4g
2.00
1.99
2.04
2.94
3.01
9.5 9.0 8.5 8.0 7.5 7.0 6.5 6.0 5.5 5.0 4.5 4.0 3.5 3.0 2.5 2.0 1.5 1.0 0.5 0.0
f1 (ppm)

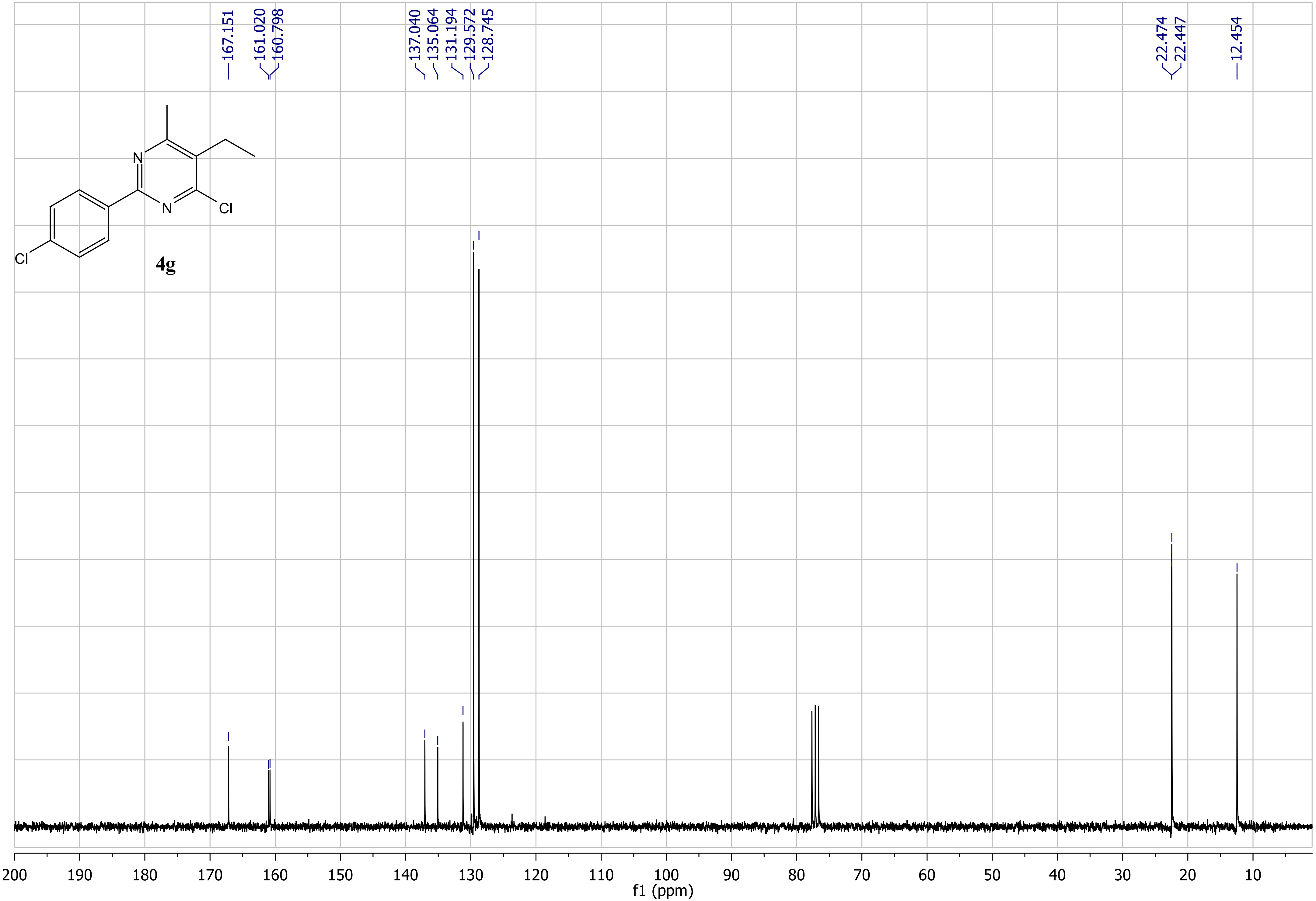
167.151
161.020
160.798
137.040
135.064
131.194
129.572
128.745
22.474
22.447
12.454
N
N
Cl
Cl
4g
200 190 180 170 160 150 140 130 120 110 100 90 80 70 60 50 40 30 20 10
f1 (ppm)

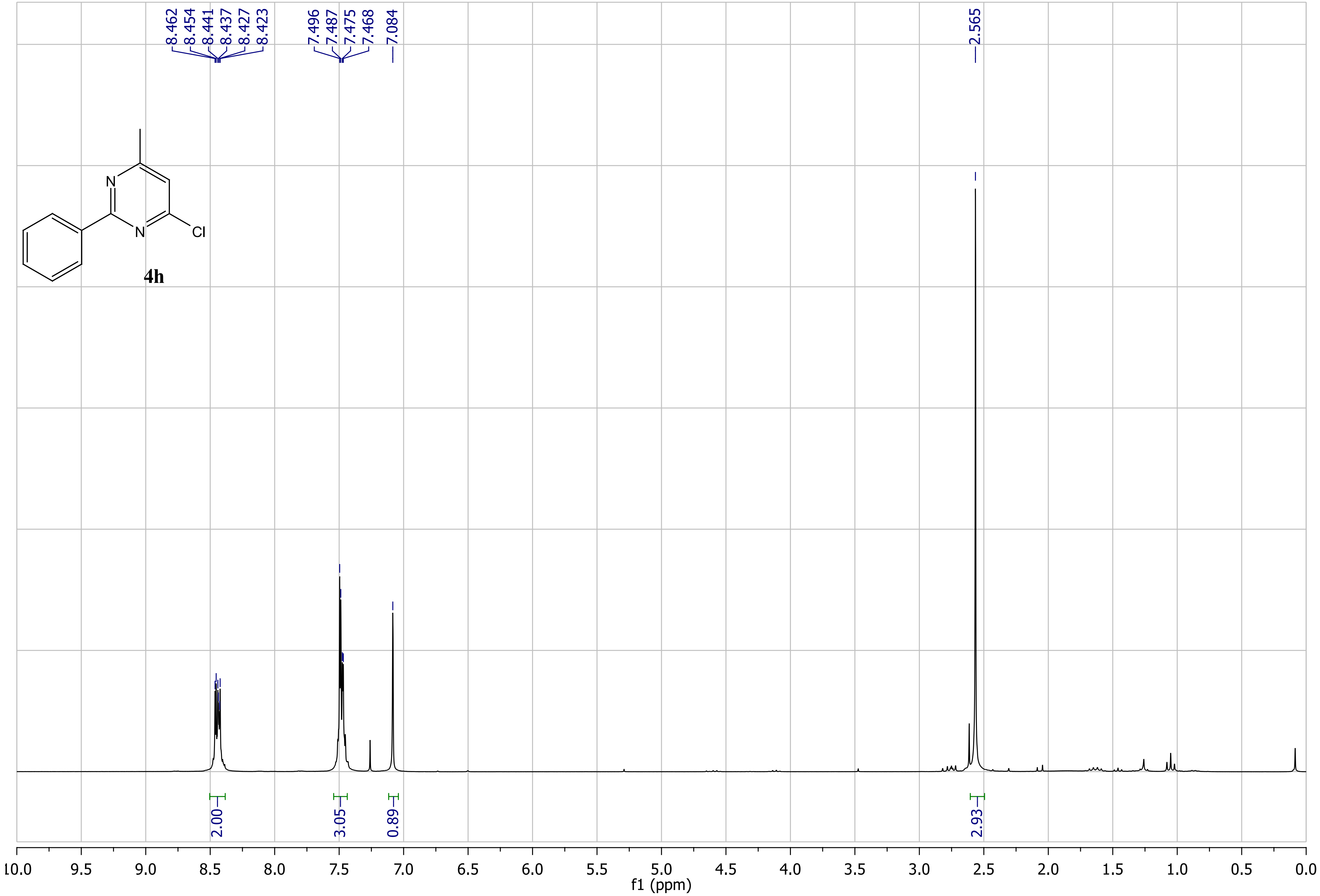
8.462
8.454
8.441
8.437
8.427
8.423
7.496
7.487
7.475
7.468
7.084
2.565
N
N
Cl
4h
2.00
3.05
0.89
2.93
10.0
9.5
9.0
8.5
8.0
7.5
7.0
6.5
6.0
5.5
5.0
4.5
4.0
3.5
3.0
2.5
2.0
1.5
1.0
0.5
0.0
f1 (ppm)

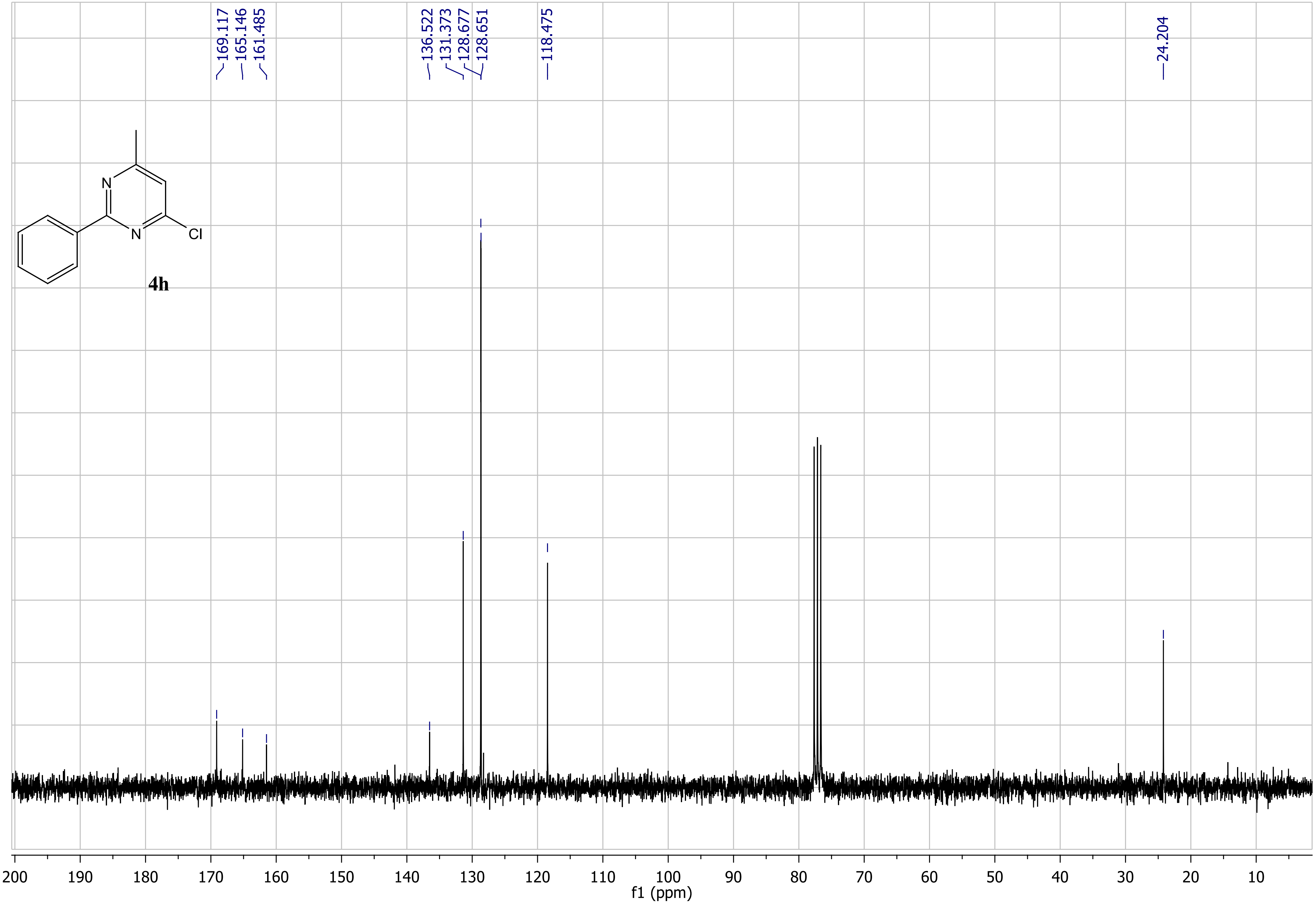

169.117
165.146
161.485
136.522
131.373
128.677
128.651
118.475
24.204
N
N
Cl
4h
200
190
180
170
160
150
140
130
120
110
100
90
80
70
60
50
40
30
20
10
f1 (ppm)

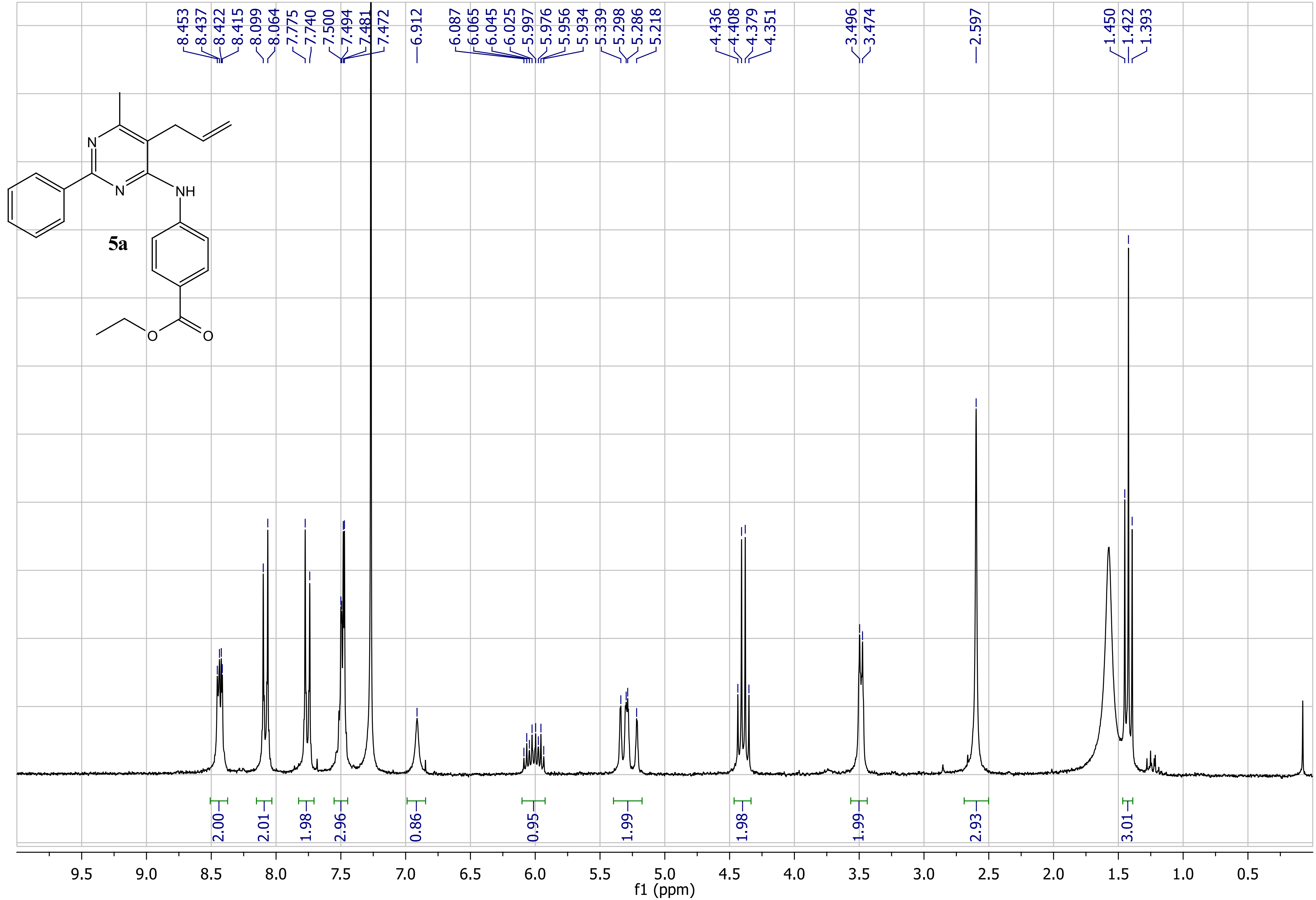
8.453
8.437
8.422
8.415
8.099
8.064
7.775
7.740
7.500
7.494
7.481
7.472
6.912
6.087
6.065
6.045
6.025
5.997
5.976
5.956
5.934
5.339
5.298
5.286
5.218
4.436
4.408
4.379
4.351
3.496
3.474
2.597
1.450
1.422
1.393
N
N
NH
5a
O
O
2.00
2.01
1.98
2.96
0.86
0.95
1.99
1.98
1.99
2.93
3.01
9.5
9.0
8.5
8.0
7.5
7.0
6.5
6.0
5.5
5.0
4.5
4.0
3.5
3.0
2.5
2.0
1.5
1.0
0.5
f1 (ppm)

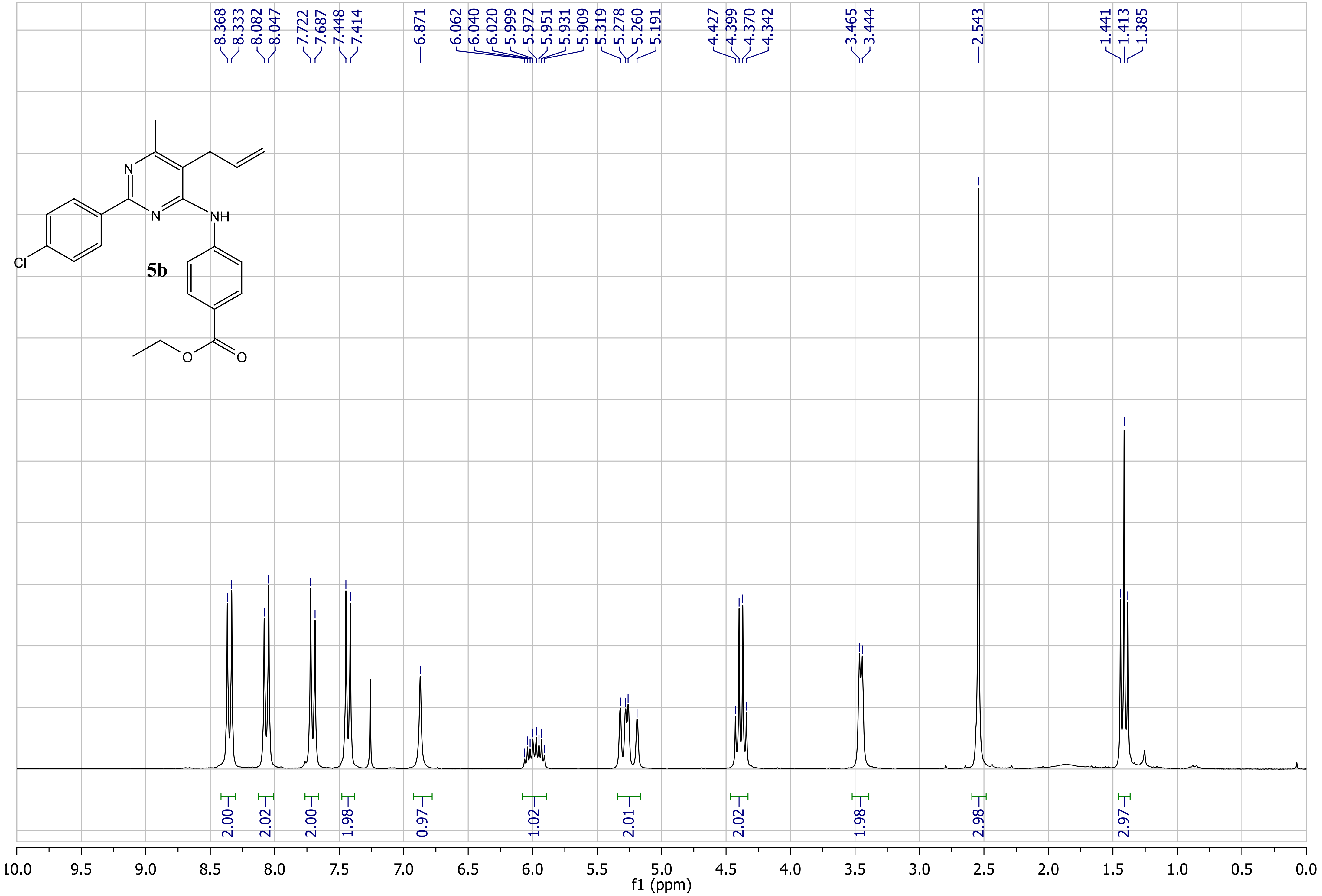

8.368
8.333
8.082
8.047
7.722
7.687
7.448
7.414
6.871
6.062
6.040
6.020
5.999
5.972
5.951
5.931
5.909
5.319
5.278
5.260
5.191
4.427
4.399
4.370
4.342
3.465
3.444
2.543
1.441
1.413
1.385
N
N
NH
Cl
5b
O
O
2.00
2.02
2.00
1.98
0.97
1.02
2.01
2.02
1.98
2.98
2.97
f1 (ppm)

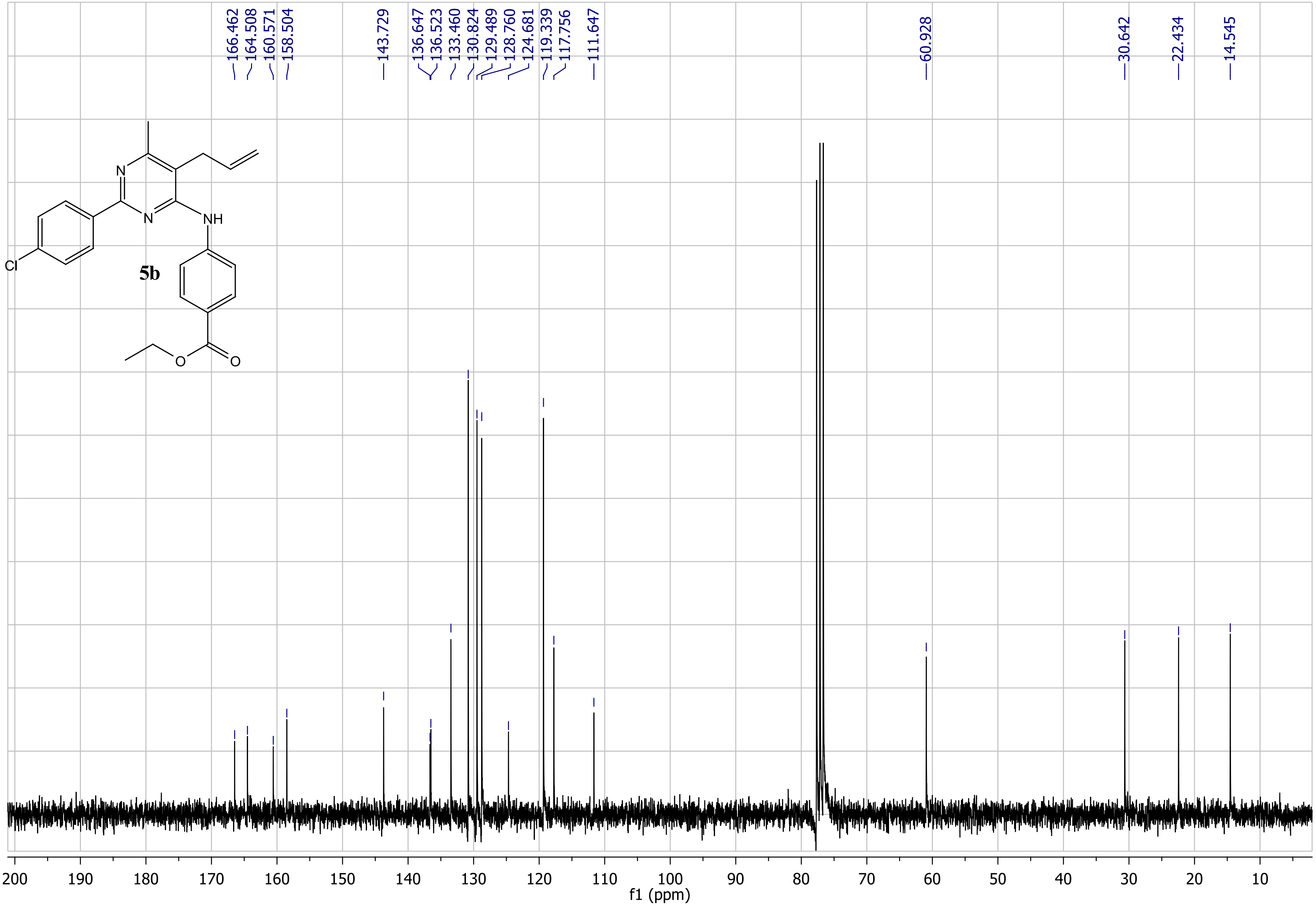
166.462
164.508
160.571
158.504
143.729
136.647
136.523
133.460
130.824
129.489
128.760
124.681
119.339
117.756
111.647
60.928
30.642
22.434
14.545
N
N
NH
Cl
O
O
5b
200 190 180 170 160 150 140 130 120 110 100 90 80 70 60 50 40 30 20 10
f1 (ppm)

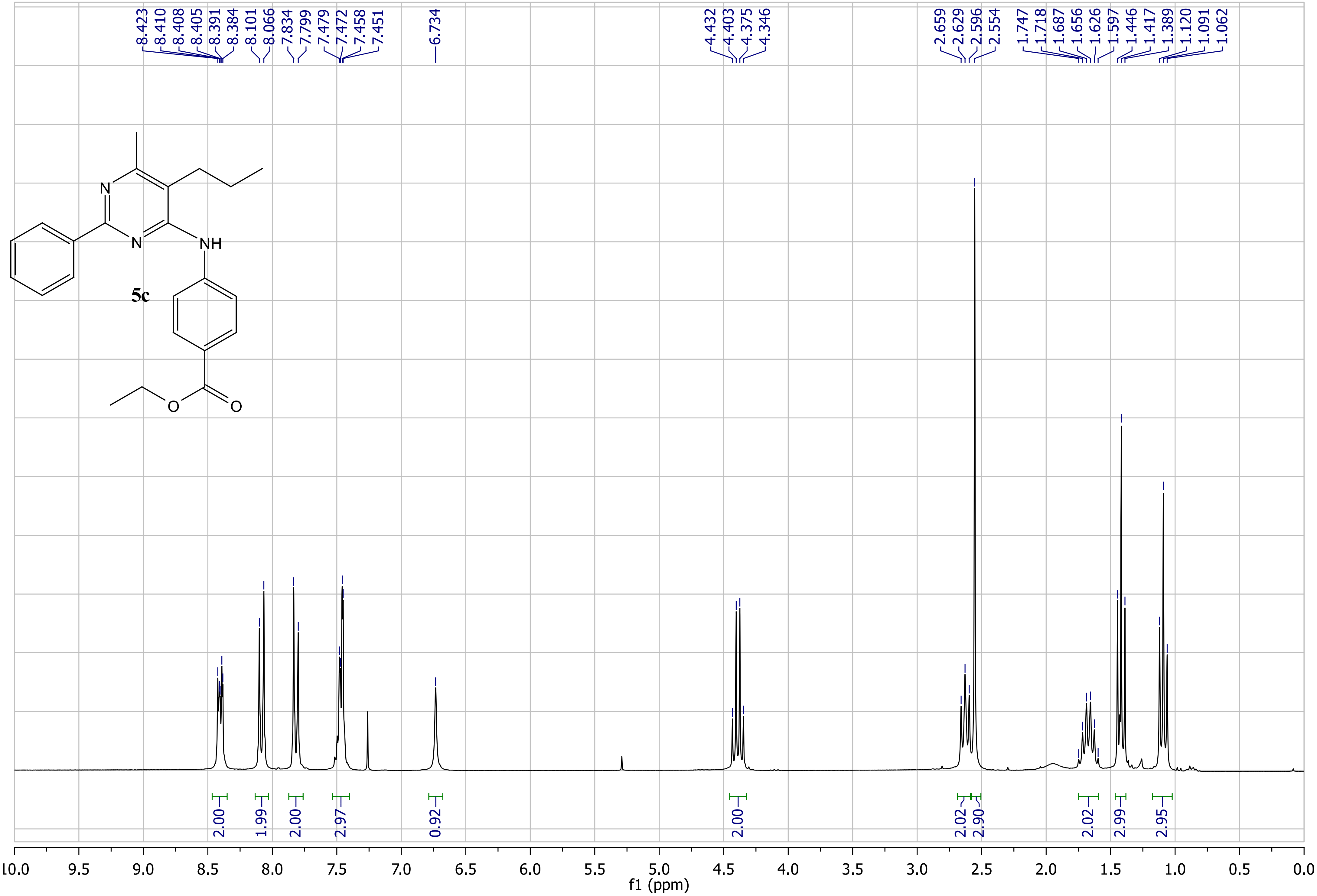
8.423
8.410
8.408
8.405
8.391
8.384
8.101
8.066
7.834
7.799
7.479
7.472
7.458
7.451
6.734
4.432
4.403
4.375
4.346
2.659
2.629
2.596
2.554
1.747
1.718
1.687
1.656
1.626
1.597
1.446
1.417
1.389
1.120
1.091
1.062
N
N
NH
O
O
5c
2.00
1.99
2.00
2.97
0.92
2.00
2.02
2.90
2.02
2.99
2.95
10.0
9.5
9.0
8.5
8.0
7.5
7.0
6.5
6.0
5.5
5.0
4.5
4.0
3.5
3.0
2.5
2.0
1.5
1.0
0.5
0.0
f1 (ppm)

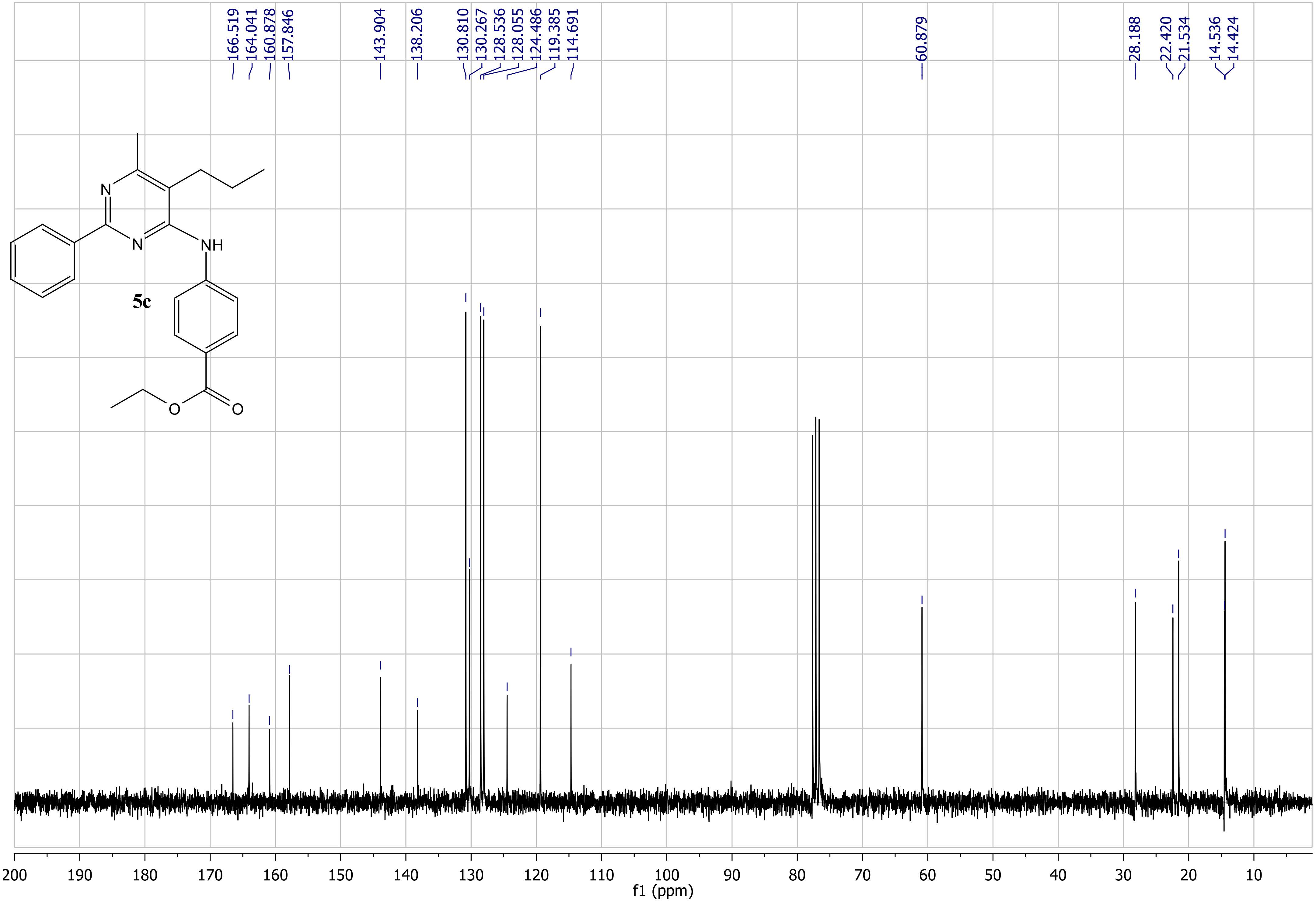
166.519
164.041
160.878
157.846
143.904
138.206
130.810
130.267
128.536
128.055
124.486
119.385
114.691
60.879
28.188
22.420
21.534
14.536
14.424
N
N
NH
O
O
5c
200
190
180
170
160
150
140
130
120
110
100
90
80
70
60
50
40
30
20
10
f1 (ppm)

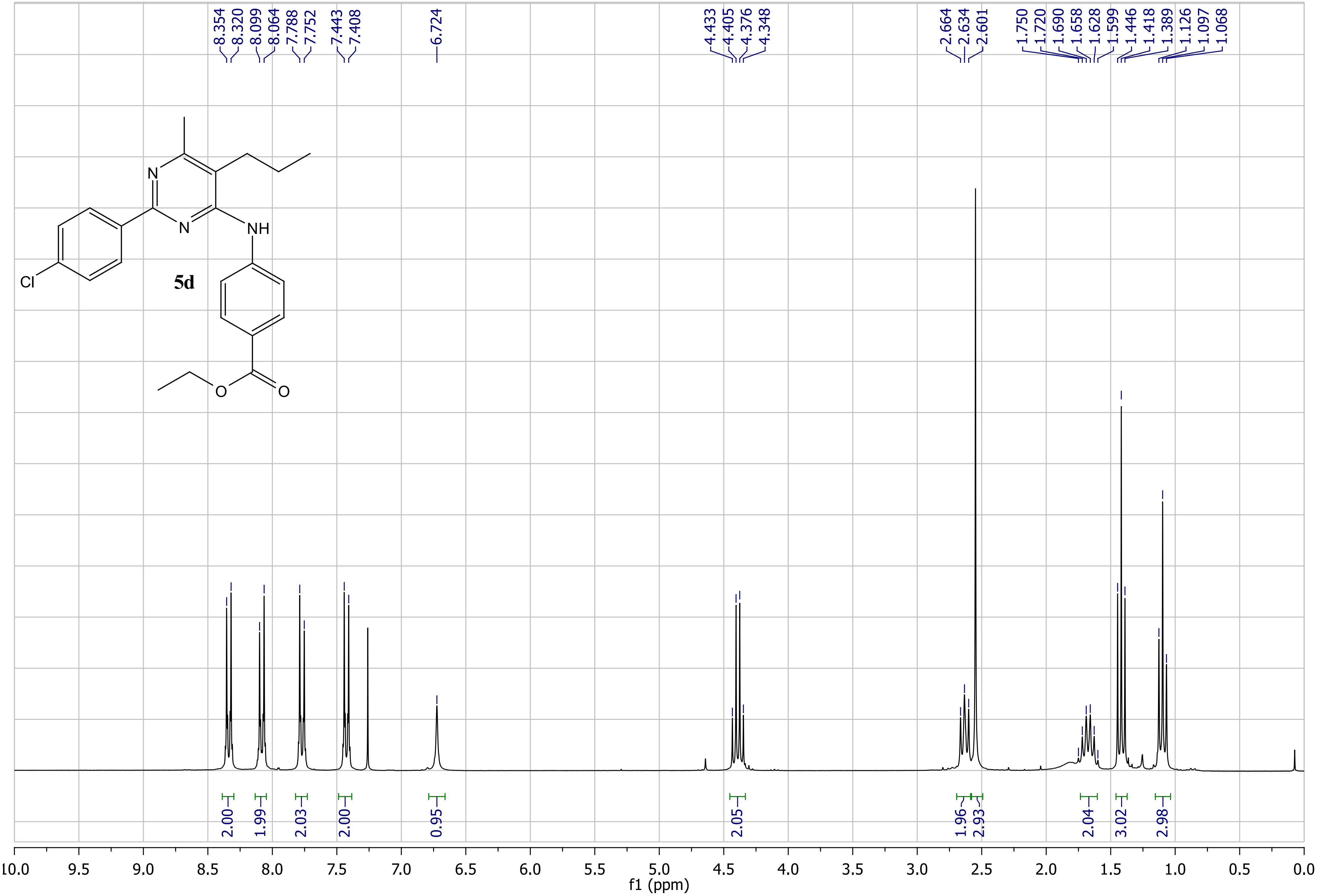
8.354
8.320
8.099
8.064
7.788
7.752
7.443
7.408
6.724
4.433
4.405
4.376
4.348
2.664
2.634
2.601
1.750
1.720
1.690
1.658
1.628
1.599
1.446
1.418
1.389
1.126
1.097
1.068
N
N
NH
Cl
5d
O
O
2.00
1.99
2.03
2.00
0.95
2.05
1.96
2.93
2.04
3.02
2.98
10.0
9.5
9.0
8.5
8.0
7.5
7.0
6.5
6.0
5.5
5.0
4.5
4.0
3.5
3.0
2.5
2.0
1.5
1.0
0.5
0.0
f1 (ppm)

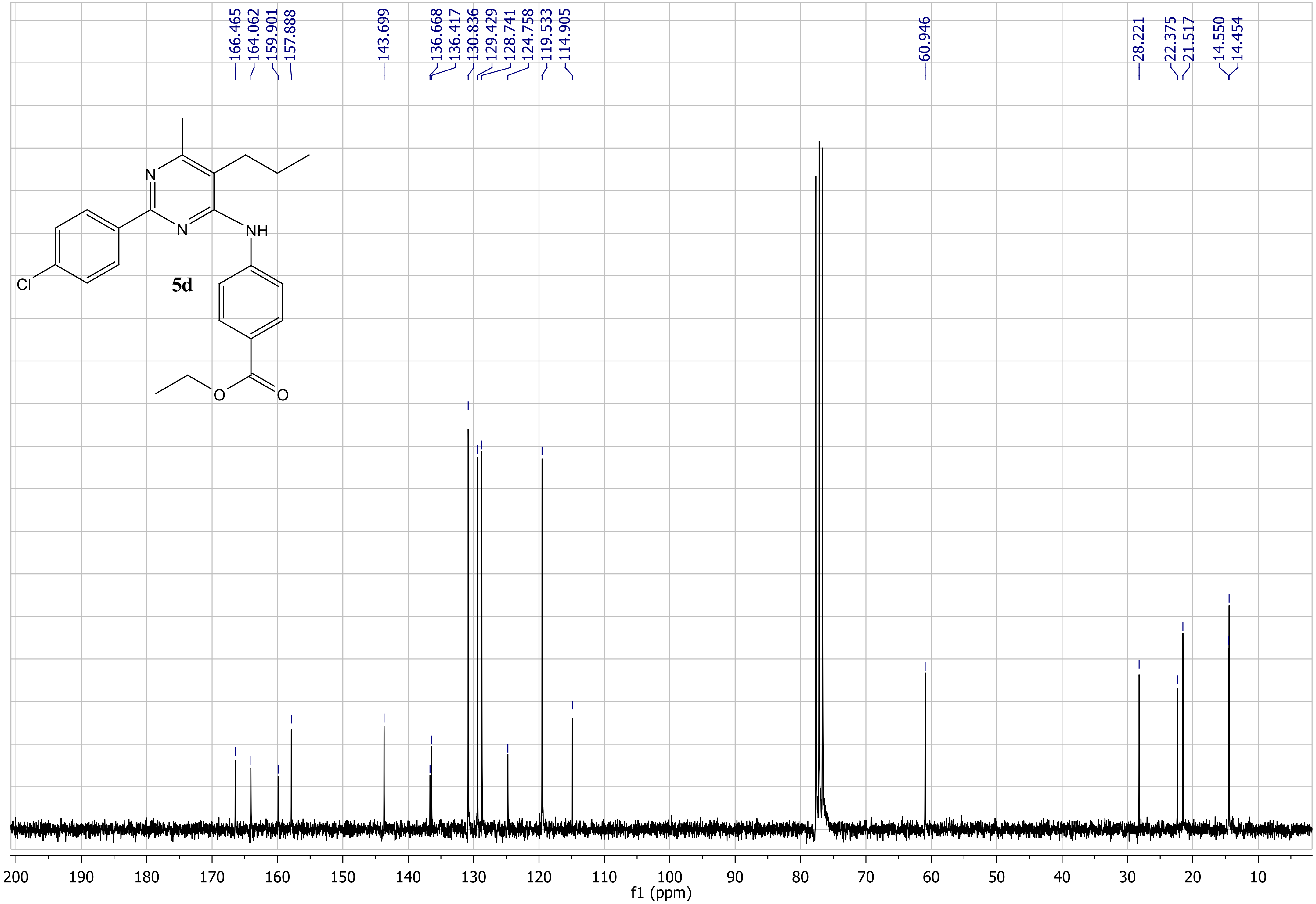
166.465
164.062
159.901
157.888
143.699
136.668
136.417
130.836
129.429
128.741
124.758
119.533
114.905
60.946
28.221
22.375
21.517
14.550
14.454
N
N
NH
Cl
5d
O
O
200 190 180 170 160 150 140 130 120 110 100 90 80 70 60 50 40 30 20 10
f1 (ppm)

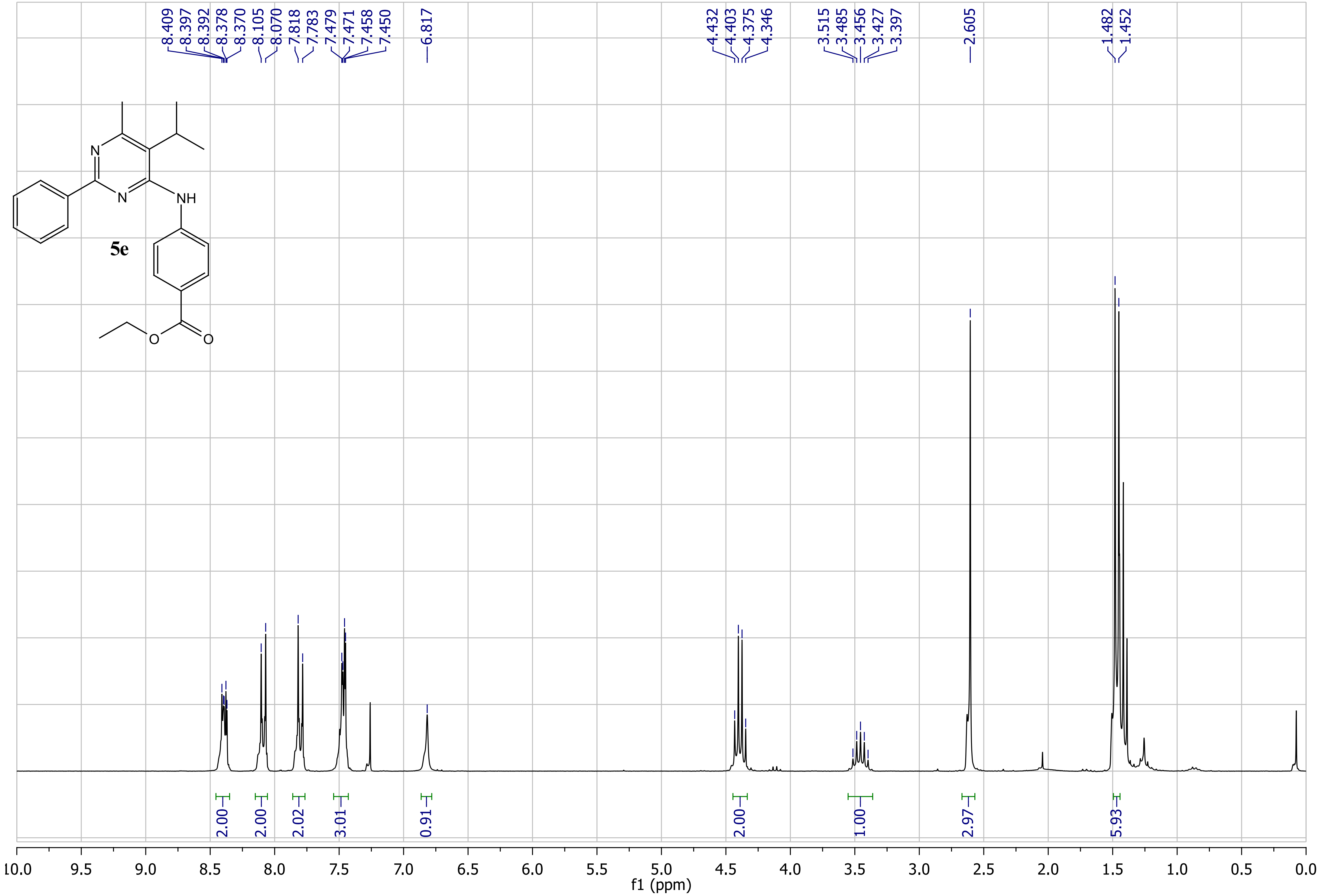
8.409
8.397
8.392
8.378
8.370
8.105
8.070
7.818
7.783
7.479
7.471
7.458
7.450
6.817
4.432
4.403
4.375
4.346
3.515
3.485
3.456
3.427
3.397
2.605
1.482
1.452
5e
2.00
2.00
2.02
3.01
0.91
2.00
1.00
2.97
5.93
f1 (ppm)

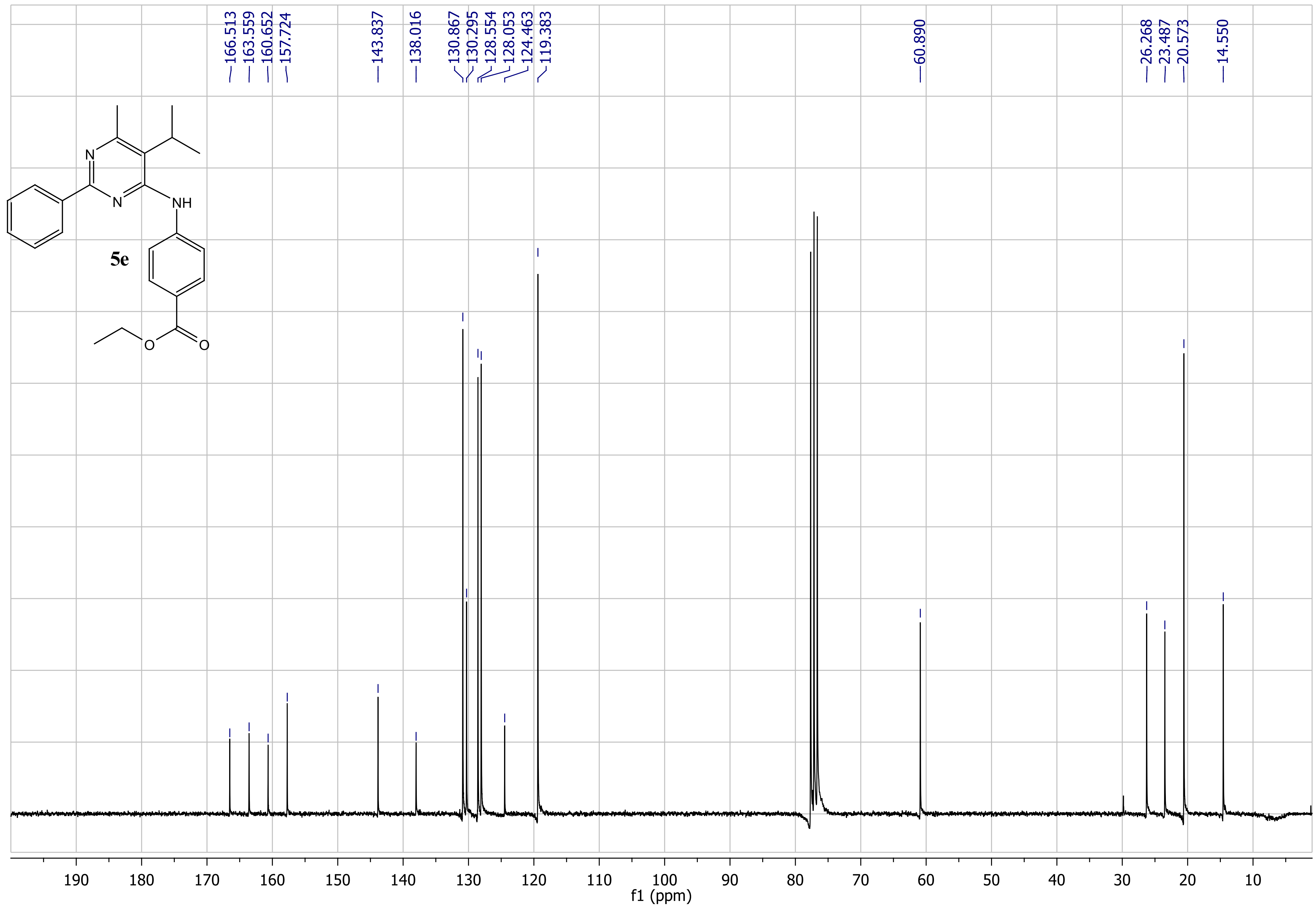

166.513
163.559
160.652
157.724
143.837
138.016
130.867
130.295
128.554
128.053
124.463
119.383
60.890
26.268
23.487
20.573
14.550
N
N
NH
O
O
5e
190
180
170
160
150
140
130
120
110
100
90
80
70
60
50
40
30
20
10
f1 (ppm)

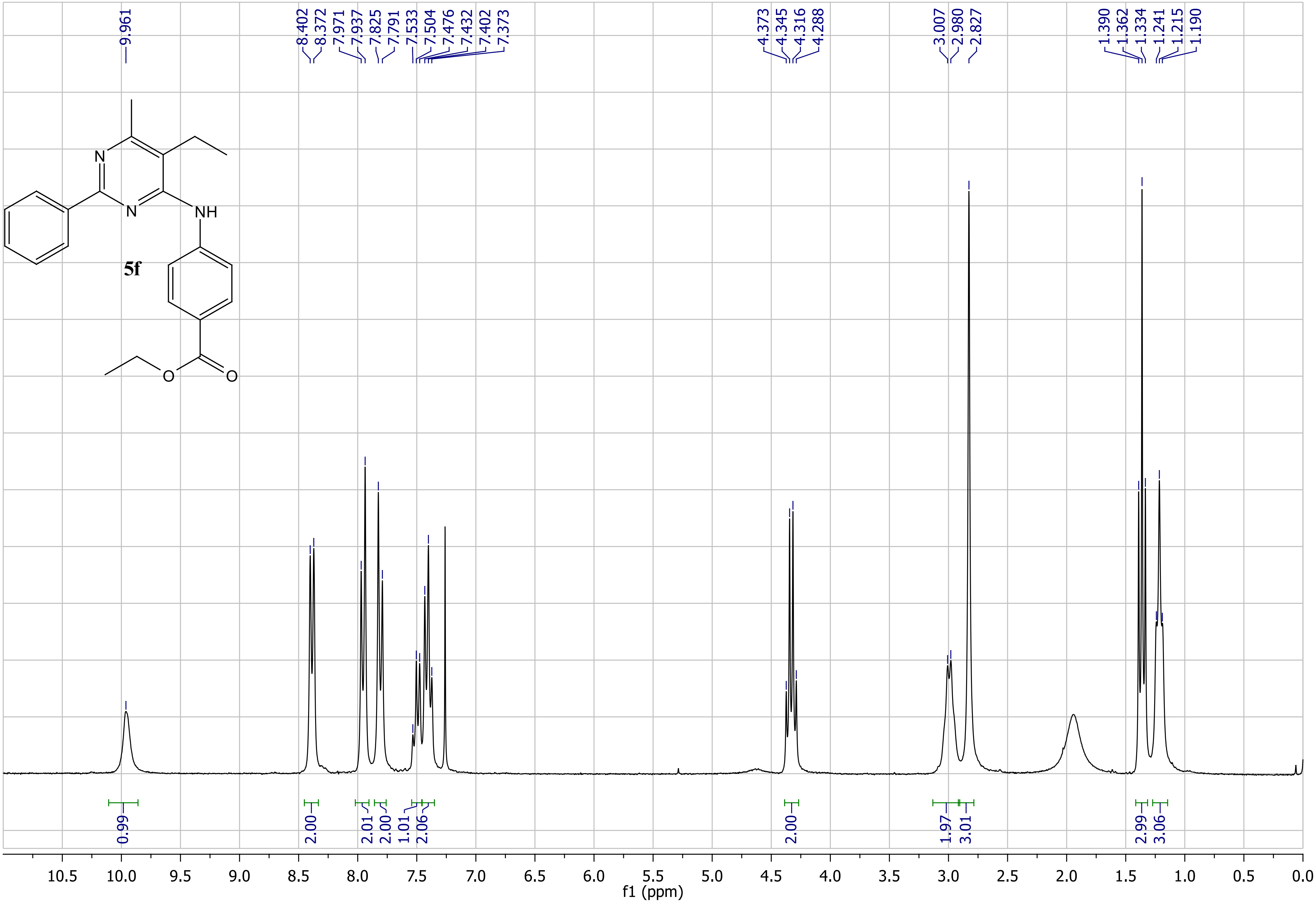
9.961
8.402
8.372
7.971
7.937
7.825
7.791
7.533
7.504
7.476
7.432
7.402
7.373
4.373
4.345
4.316
4.288
3.007
2.980
2.827
1.390
1.362
1.334
1.241
1.215
1.190
N
N
NH
5f
O
O
0.99
2.00
2.01
2.00
1.01
2.06
2.00
1.97
3.01
2.99
3.06
f1 (ppm)

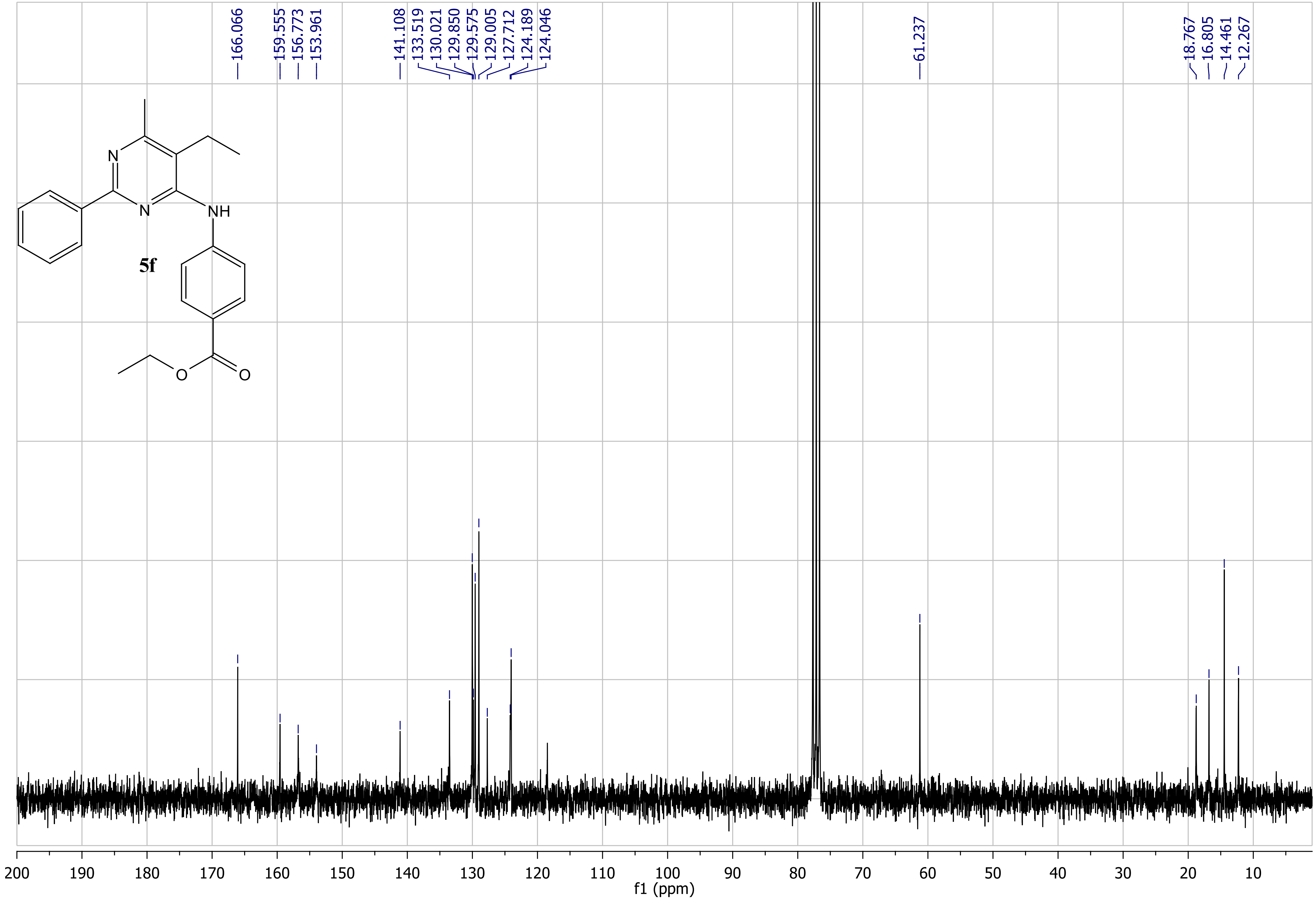
166.066
159.555
156.773
153.961
141.108
133.519
130.021
129.850
129.575
129.005
127.712
124.189
124.046
61.237
18.767
16.805
14.461
12.267
N
N
NH
5f
O
O
200 190 180 170 160 150 140 130 120 110 100 90 80 70 60 50 40 30 20 10
f1 (ppm)

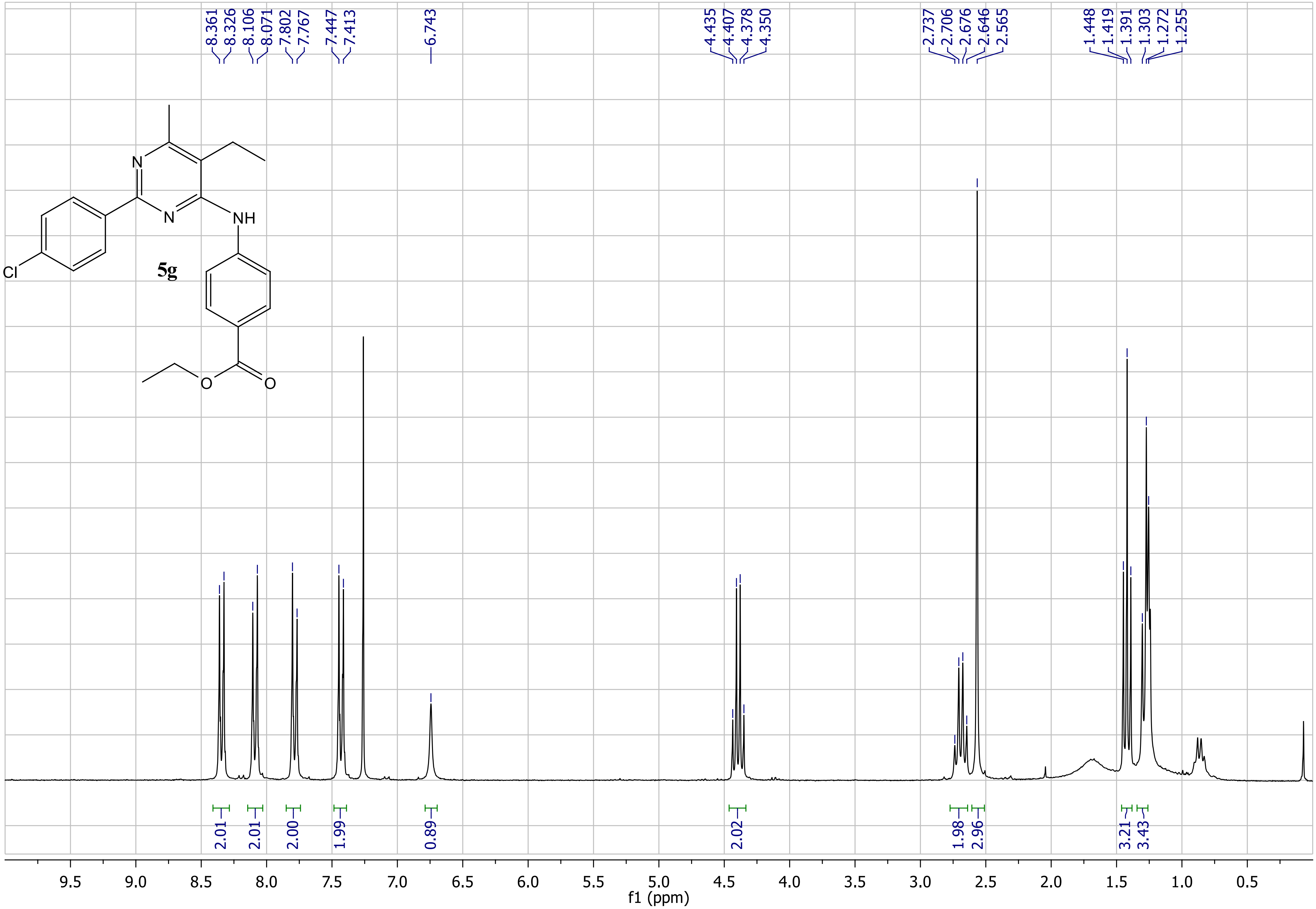
5g
8.361
8.326
8.106
8.071
7.802
7.767
7.447
7.413
6.743
4.435
4.407
4.378
4.350
2.737
2.706
2.676
2.646
2.565
1.448
1.419
1.391
1.303
1.272
1.255
2.01
2.01
2.00
1.99
0.89
2.02
1.98
2.96
3.21
3.43
9.5
9.0
8.5
8.0
7.5
7.0
6.5
6.0
5.5
5.0
4.5
4.0
3.5
3.0
2.5
2.0
1.5
1.0
0.5
f1 (ppm)

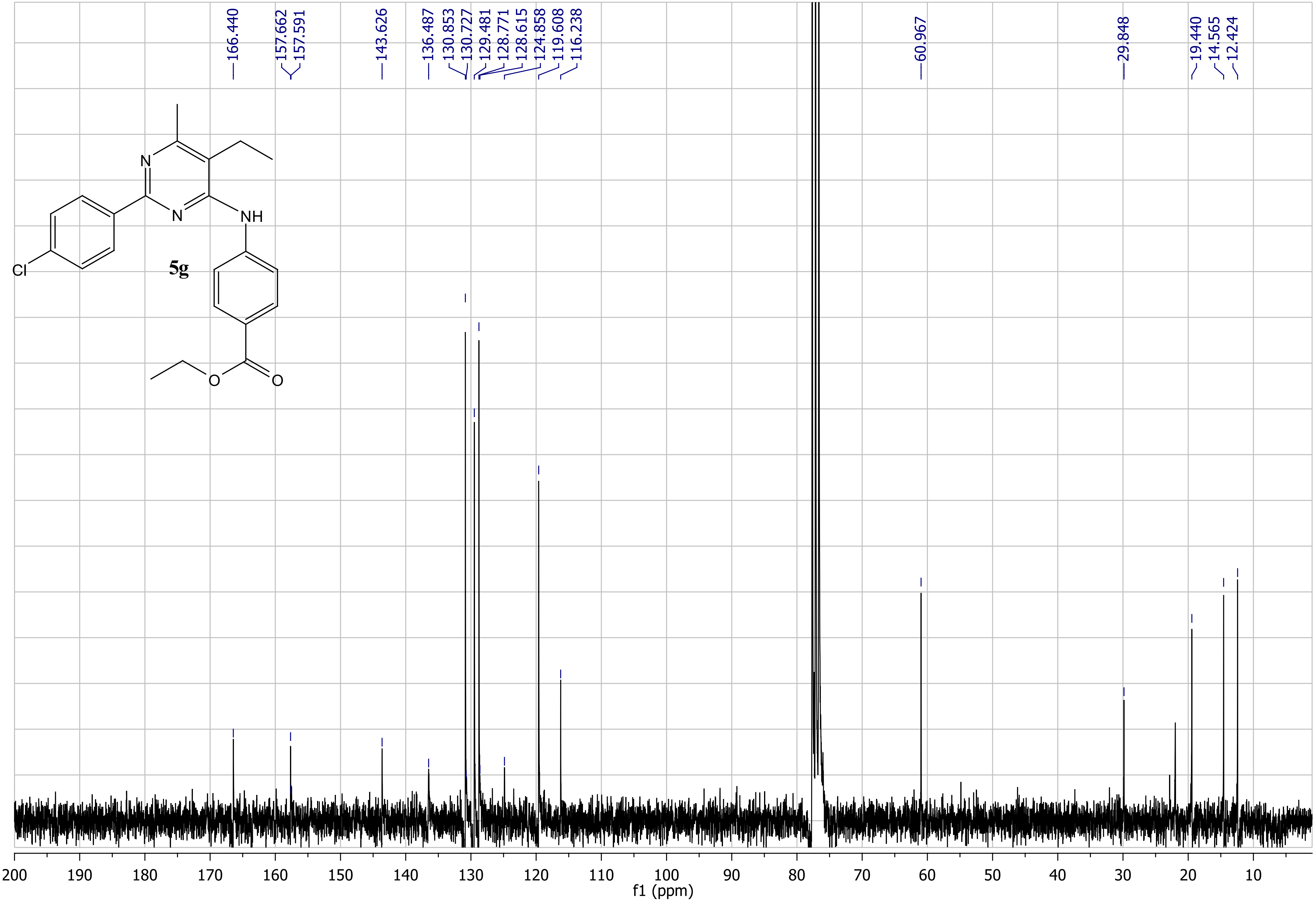

166.440
157.662
157.591
143.626
136.487
130.853
130.727
129.481
128.771
128.615
124.858
119.608
116.238
60.967
29.848
19.440
14.565
12.424
N
N
NH
Cl
5g
O
O
200
190
180
170
160
150
140
130
120
110
100
90
80
70
60
50
40
30
20
10
f1 (ppm)

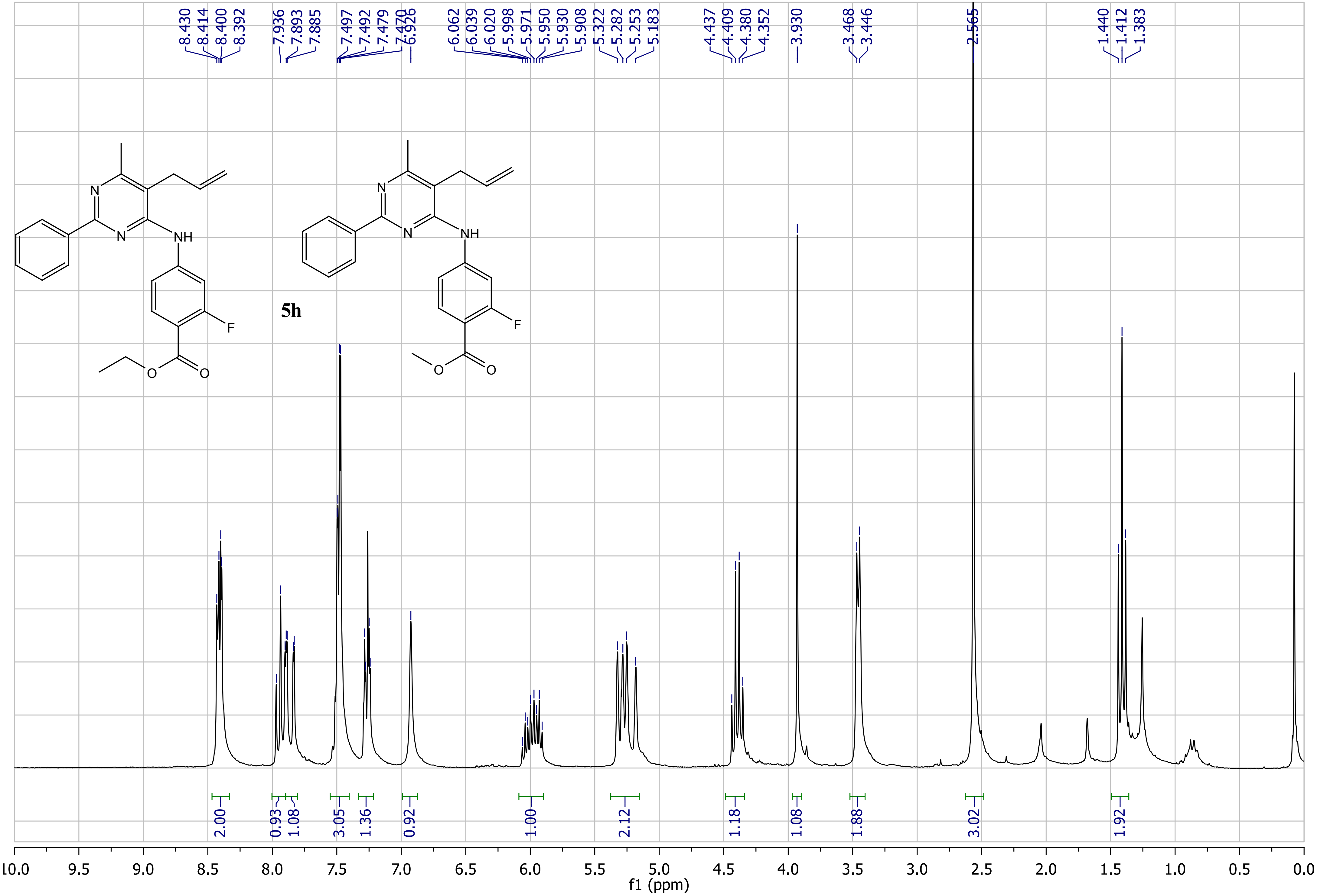

8.430
8.414
8.400
8.392
7.936
7.893
7.885
7.497
7.492
7.479
7.470
6.926
6.062
6.039
6.020
5.998
5.971
5.950
5.930
5.908
5.322
5.282
5.253
5.183
4.437
4.409
4.380
4.352
3.930
3.468
3.446
2.565
1.440
1.412
1.383
N
N
NH
F
O
O
5h
N
N
NH
F
O
O
2.00
0.93
1.08
3.05
1.36
0.92
1.00
2.12
1.18
1.08
1.88
3.02
1.92
10.0
9.5
9.0
8.5
8.0
7.5
7.0
6.5
6.0
5.5
5.0
4.5
4.0
3.5
3.0
2.5
2.0
1.5
1.0
0.5
0.0
f1 (ppm)

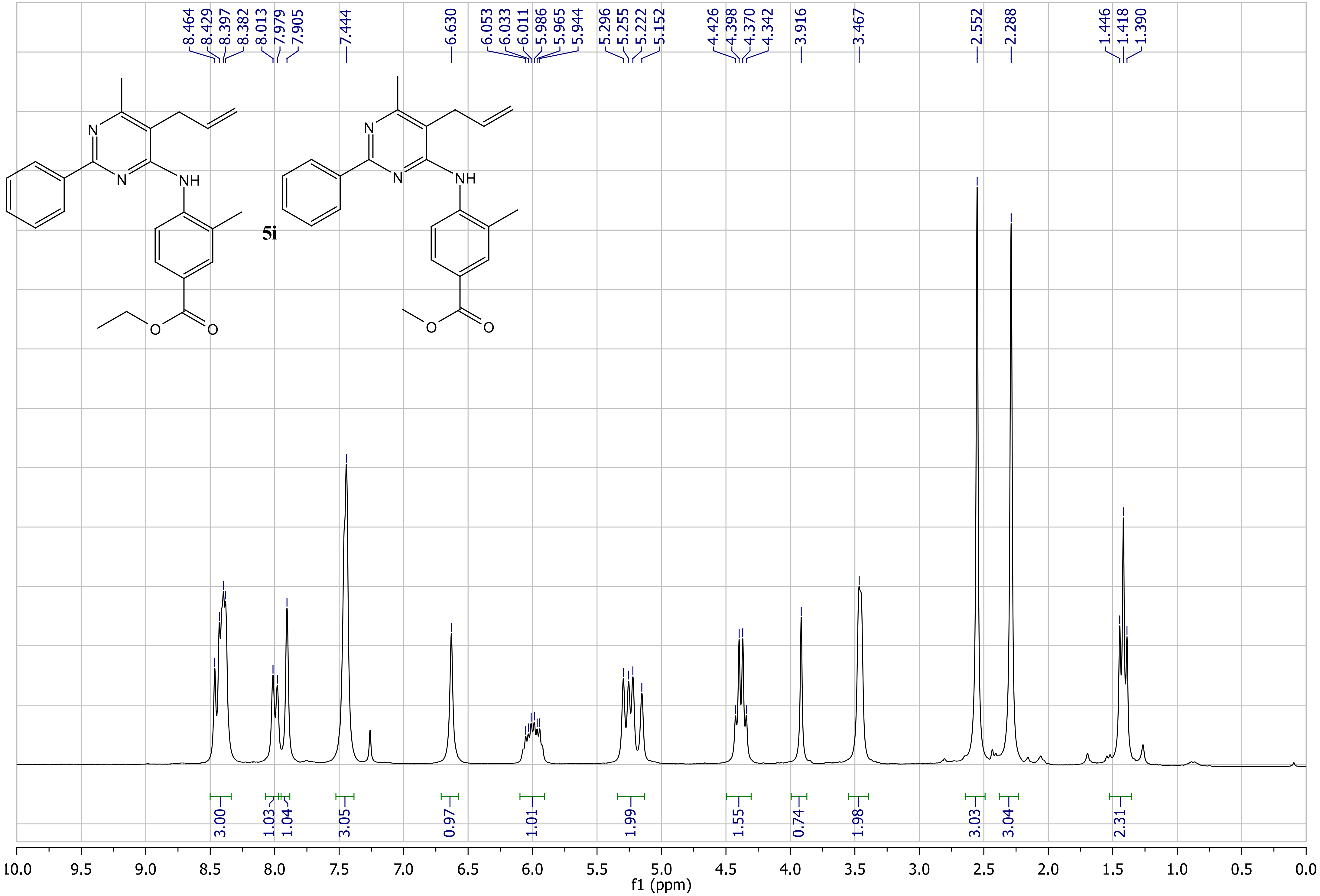
8.464
8.429
8.397
8.382
8.013
7.979
7.905
7.444
6.630
6.053
6.033
6.011
5.986
5.965
5.944
5.296
5.255
5.222
5.152
4.426
4.398
4.370
4.342
3.916
3.467
2.552
2.288
1.446
1.418
1.390
N
N
NH
O
O
5i
N
N
NH
O
O
3.00
1.03
1.04
3.05
0.97
1.01
1.99
1.55
0.74
1.98
3.03
3.04
2.31
10.0
9.5
9.0
8.5
8.0
7.5
7.0
6.5
6.0
5.5
5.0
4.5
4.0
3.5
3.0
2.5
2.0
1.5
1.0
0.5
0.0
f1 (ppm)

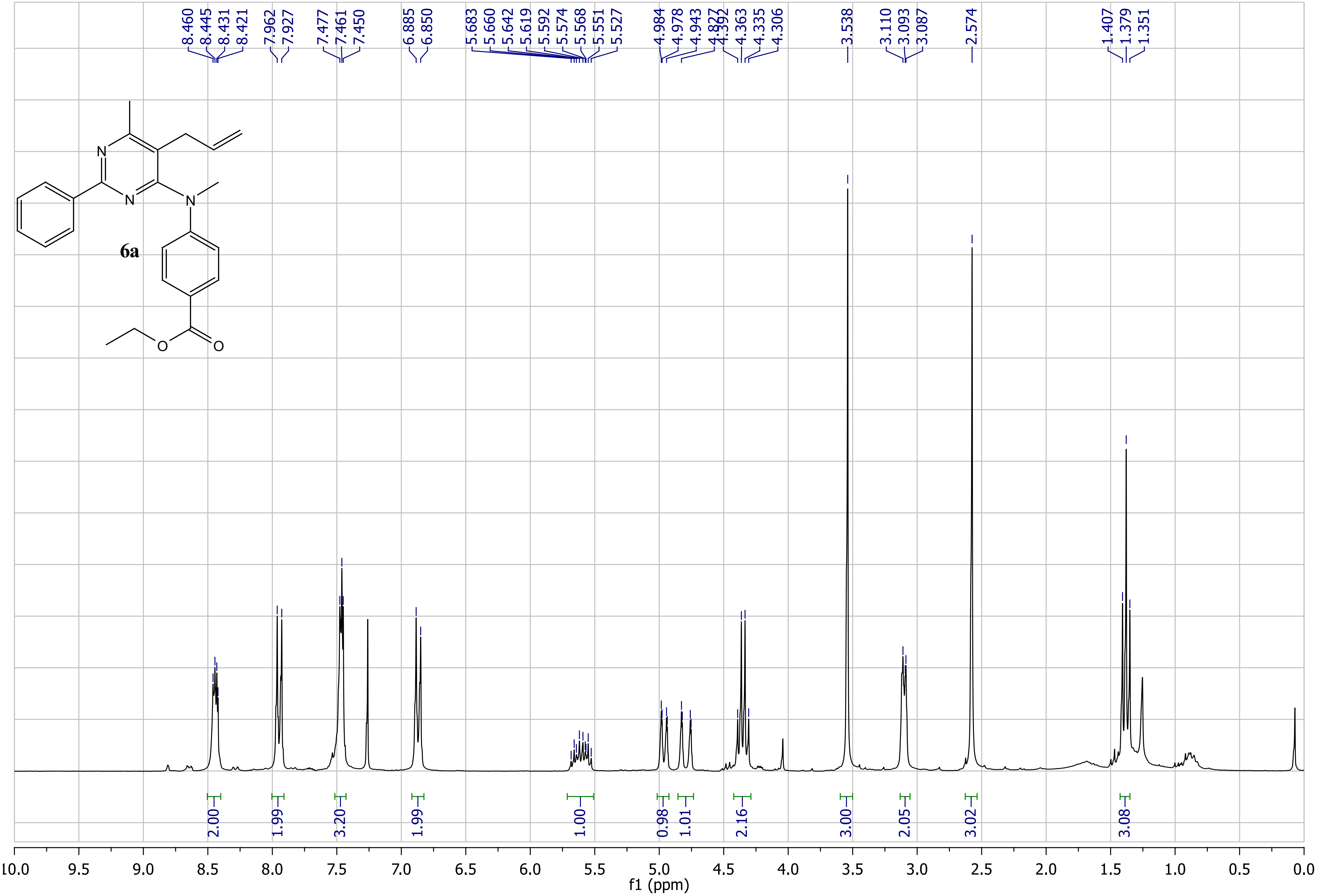

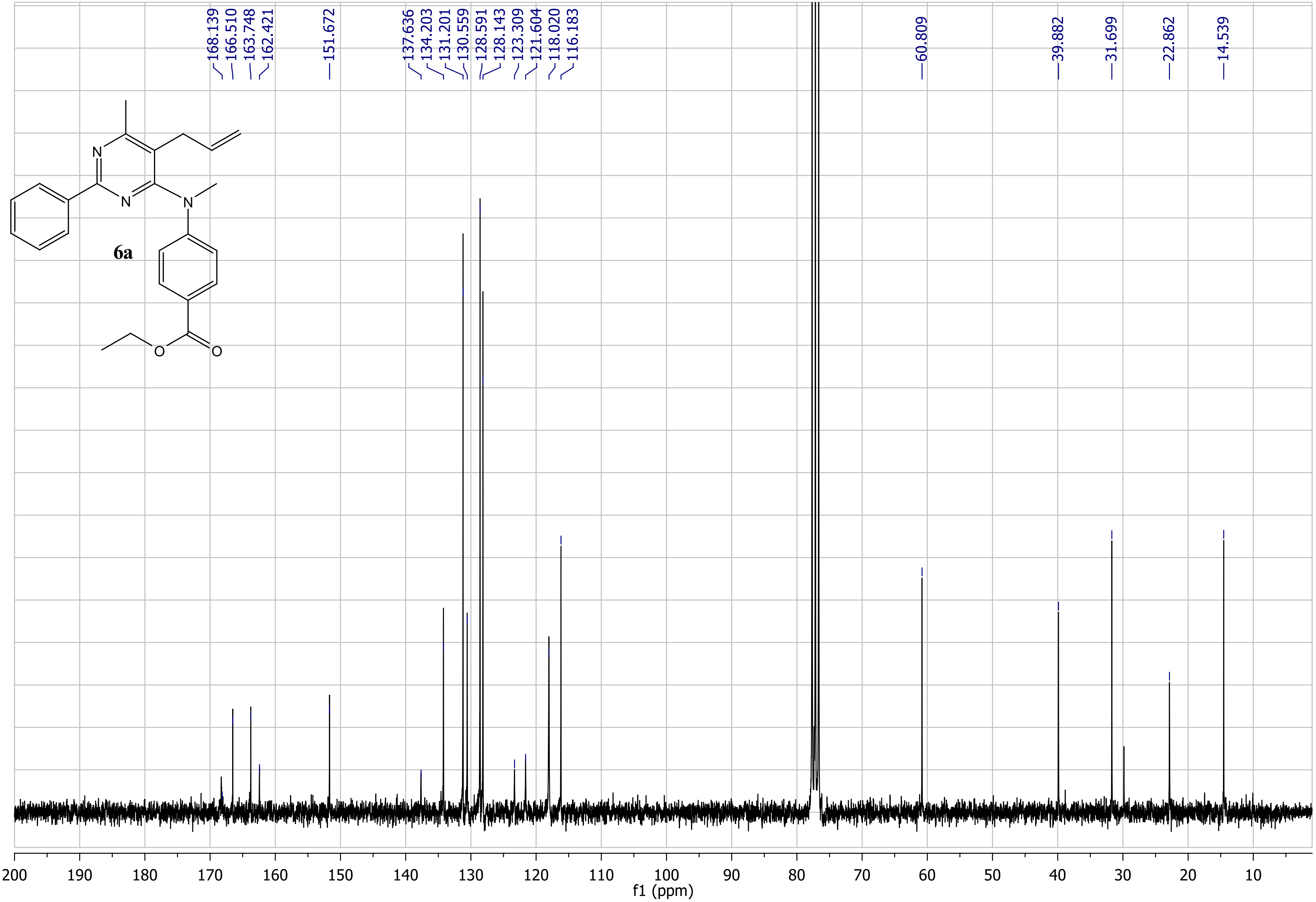

168.139
166.510
163.748
162.421
151.672
137.636
134.203
131.201
130.559
128.591
128.143
123.309
121.604
118.020
116.183
60.809
39.882
31.699
22.862
14.539
N
N
N
O
O
6a
200 190 180 170 160 150 140 130 120 110 100 90 80 70 60 50 40 30 20 10
f1 (ppm)

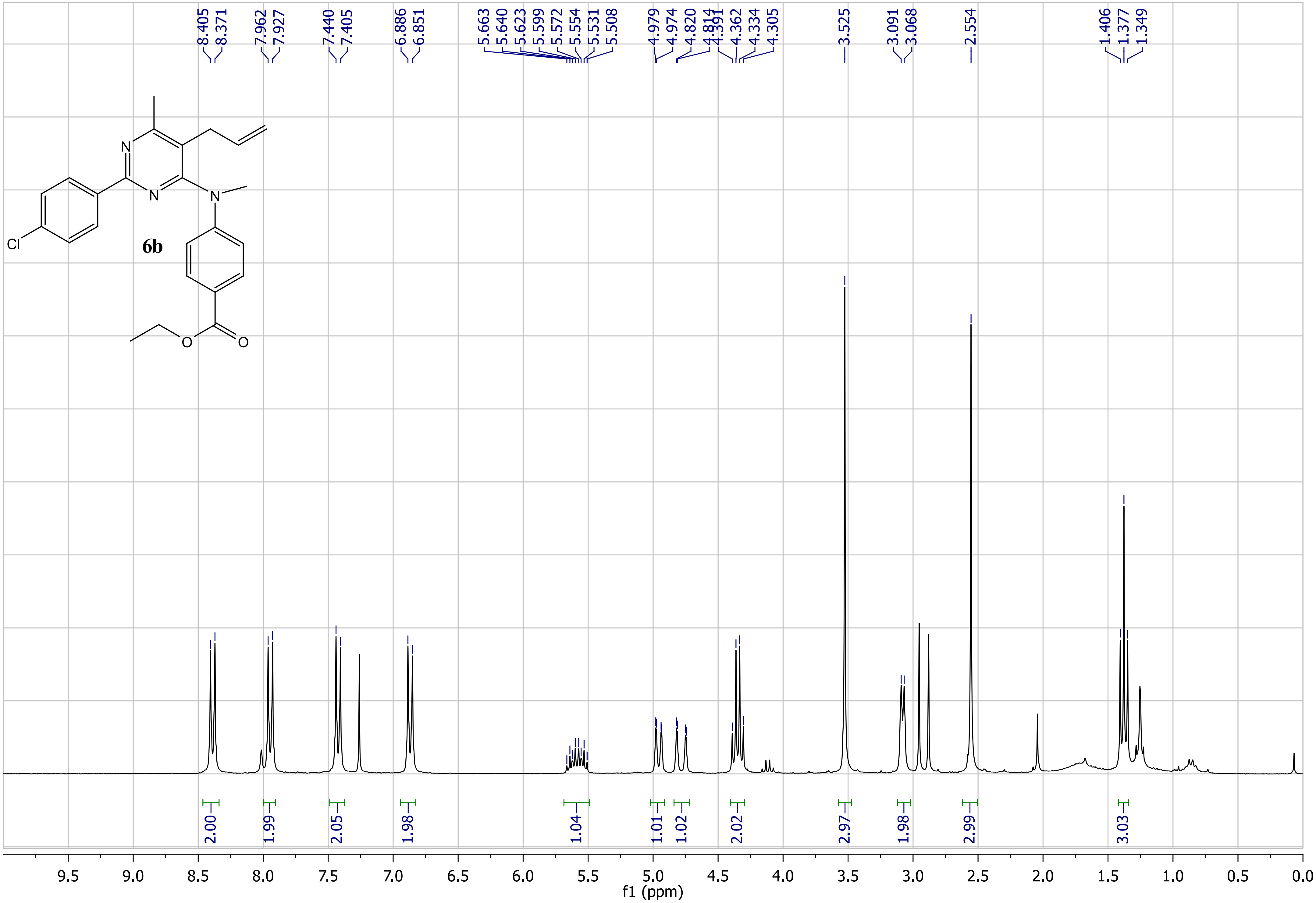
8.405
8.371
7.962
7.927
7.440
7.405
6.886
6.851
5.663
5.640
5.623
5.599
5.572
5.554
5.531
5.508
4.979
4.974
4.820
4.814
4.391
4.362
4.334
4.305
3.525
3.091
3.068
2.554
1.406
1.377
1.349
N
N
N
Cl
O
O
6b
2.00
1.99
2.05
1.98
1.04
1.01
1.02
2.02
2.97
1.98
2.99
3.03
9.5
9.0
8.5
8.0
7.5
7.0
6.5
6.0
5.5
5.0
4.5
4.0
3.5
3.0
2.5
2.0
1.5
1.0
0.5
0.0
f1 (ppm)

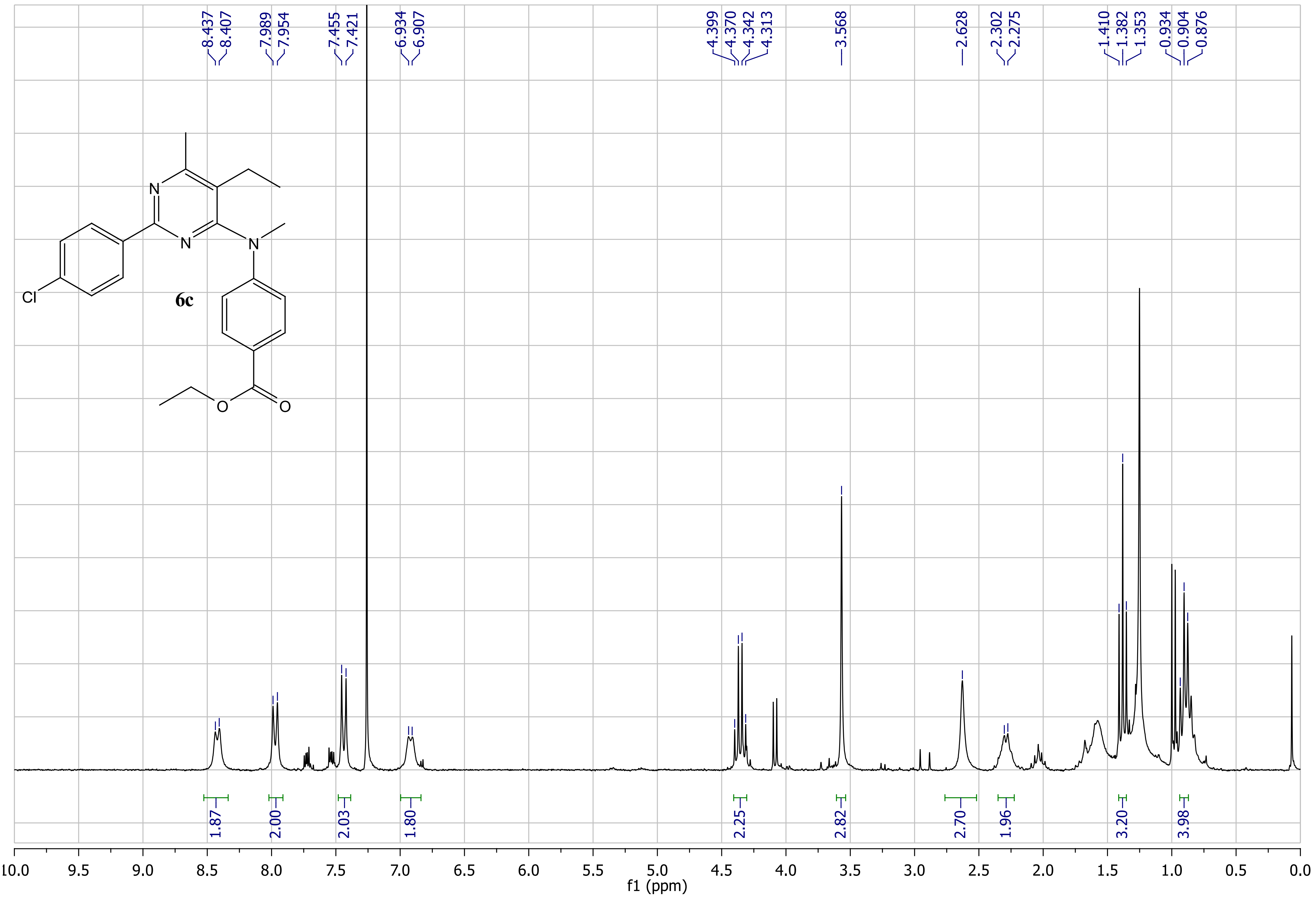
8.437
8.407
7.989
7.954
7.455
7.421
6.934
6.907
4.399
4.370
4.342
4.313
3.568
2.628
2.302
2.275
1.410
1.382
1.353
0.934
0.904
0.876
Cl
N
N
N
O
O
6c
1.87
2.00
2.03
1.80
2.25
2.82
2.70
1.96
3.20
3.98
10.0 9.5 9.0 8.5 8.0 7.5 7.0 6.5 6.0 5.5 5.0 4.5 4.0 3.5 3.0 2.5 2.0 1.5 1.0 0.5 0.0
f1 (ppm)

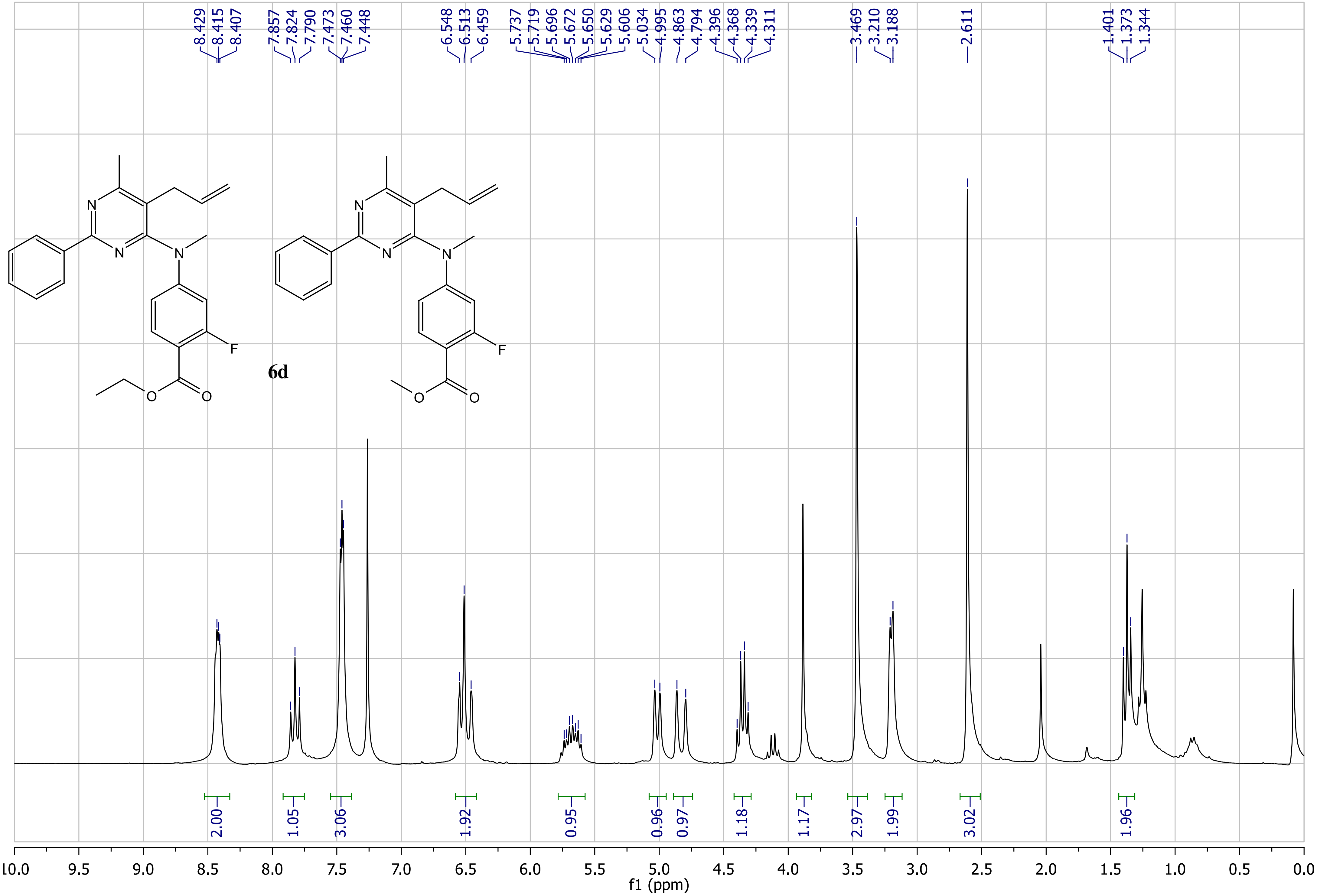
8.429
8.415
8.407
7.857
7.824
7.790
7.473
7.460
7.448
6.548
6.513
6.459
5.737
5.719
5.696
5.672
5.650
5.629
5.606
5.034
4.995
4.863
4.794
4.396
4.368
4.339
4.311
3.469
3.210
3.188
2.611
1.401
1.373
1.344
N
N
N
F
O
O
6d
2.00
1.05
3.06
1.92
0.95
0.96
0.97
1.18
1.17
2.97
1.99
3.02
1.96
f1 (ppm)

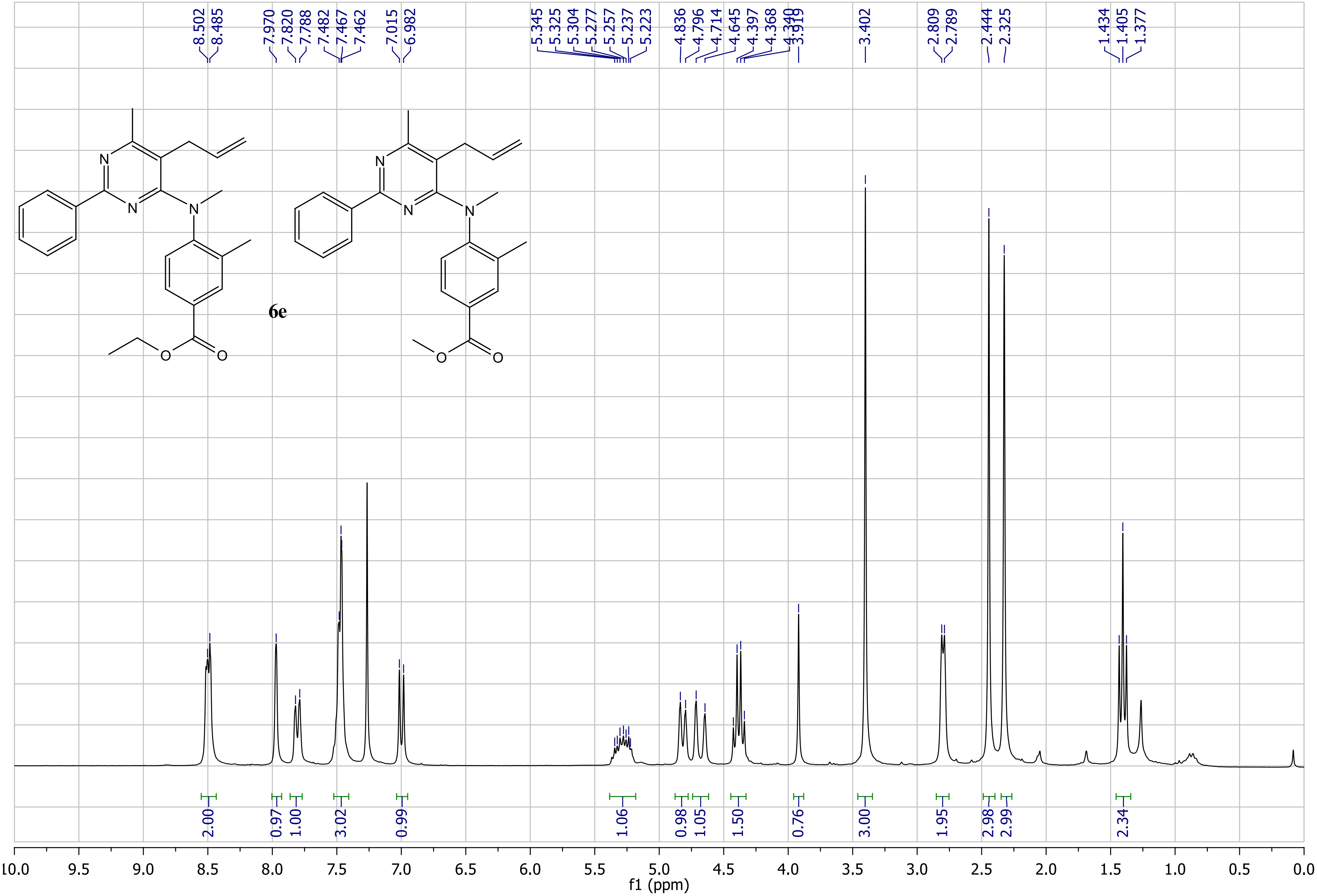

8.502
8.485
7.970
7.820
7.788
7.482
7.467
7.462
7.015
6.982
5.345
5.325
5.304
5.277
5.257
5.237
5.223
4.836
4.796
4.714
4.645
4.397
4.368
4.340
3.919
3.402
2.809
2.789
2.444
2.325
1.434
1.405
1.377
6e
2.00
0.97
1.00
3.02
0.99
1.06
0.98
1.05
1.50
0.76
3.00
1.95
2.98
2.99
2.34
f1 (ppm)

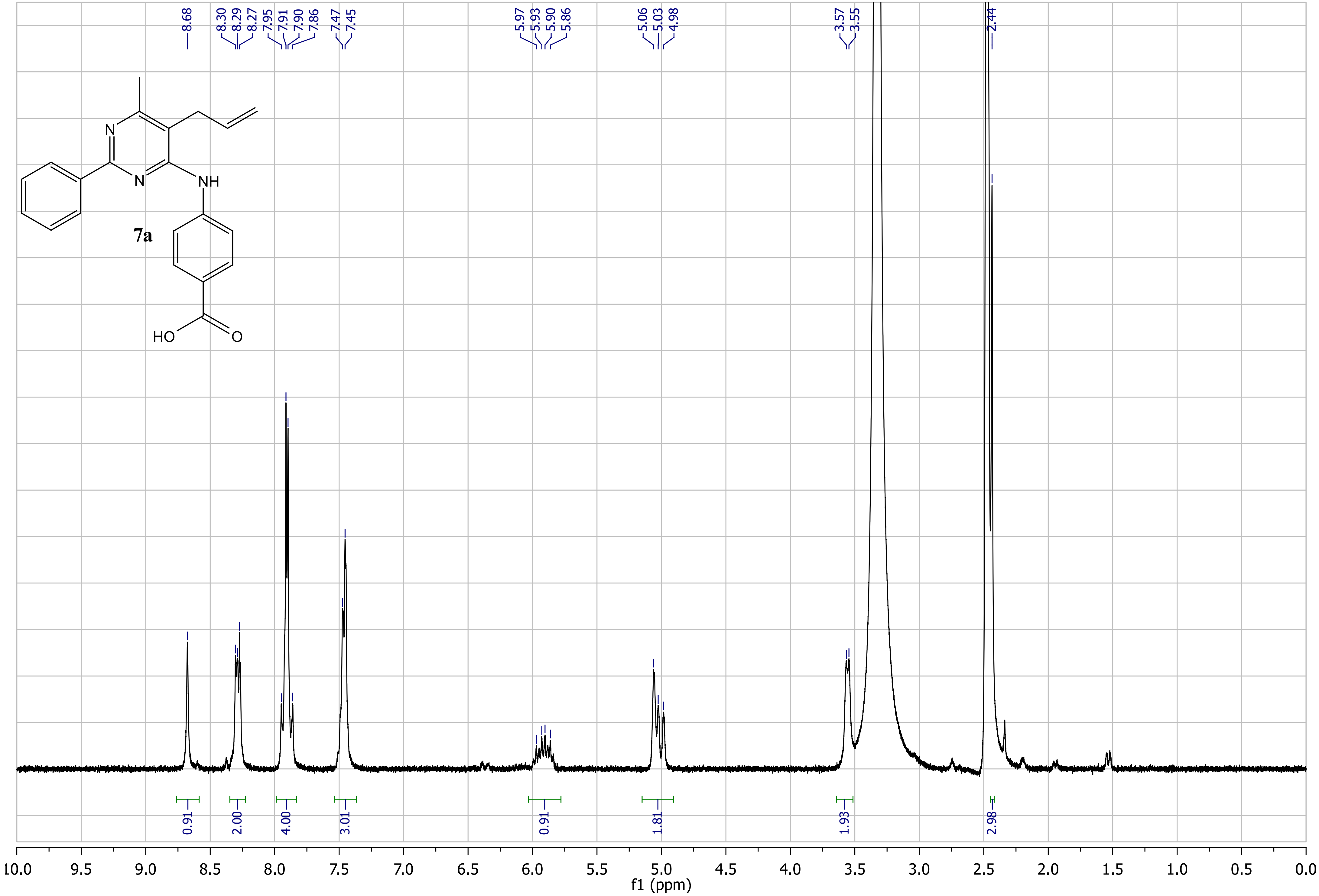

8.68
8.30
8.29
8.27
7.95
7.91
7.90
7.86
7.47
7.45
5.97
5.93
5.90
5.86
5.06
5.03
4.98
3.57
3.55
2.44
N
N
NH
HO
O
7a
0.91
2.00
4.00
3.01
0.91
1.81
1.93
2.98
10.0
9.5
9.0
8.5
8.0
7.5
7.0
6.5
6.0
5.5
5.0
4.5
4.0
3.5
3.0
2.5
2.0
1.5
1.0
0.5
0.0
f1 (ppm)

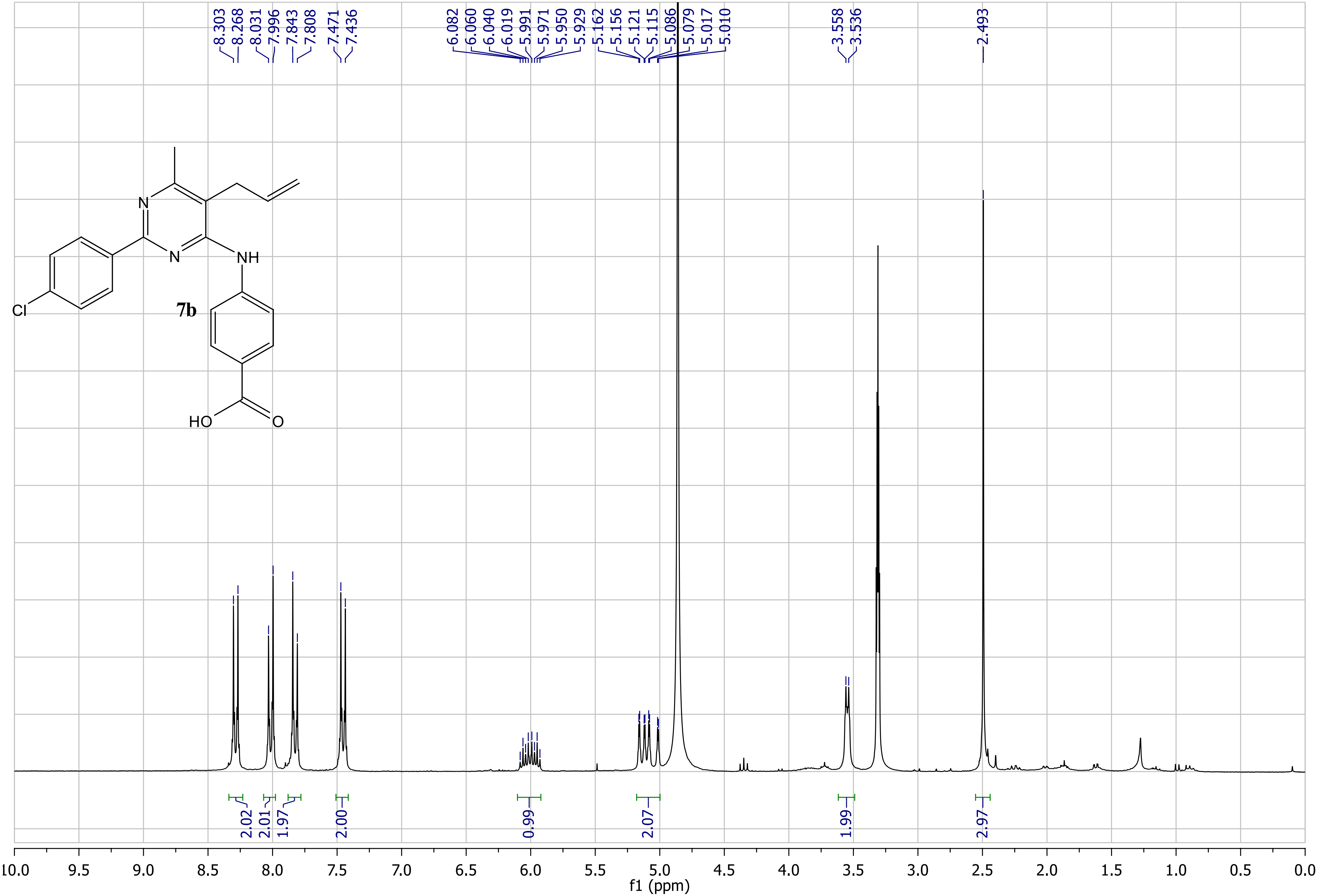
8.303
8.268
8.031
7.996
7.843
7.808
7.471
7.436
6.082
6.060
6.040
6.019
5.991
5.971
5.950
5.929
5.162
5.156
5.121
5.115
5.086
5.079
5.017
5.010
3.558
3.536
2.493
N
N
NH
Cl
7b
HO
O
2.02
2.01
1.97
2.00
0.99
2.07
1.99
2.97
10.0 9.5 9.0 8.5 8.0 7.5 7.0 6.5 6.0 5.5 5.0 4.5 4.0 3.5 3.0 2.5 2.0 1.5 1.0 0.5 0.0
f1 (ppm)

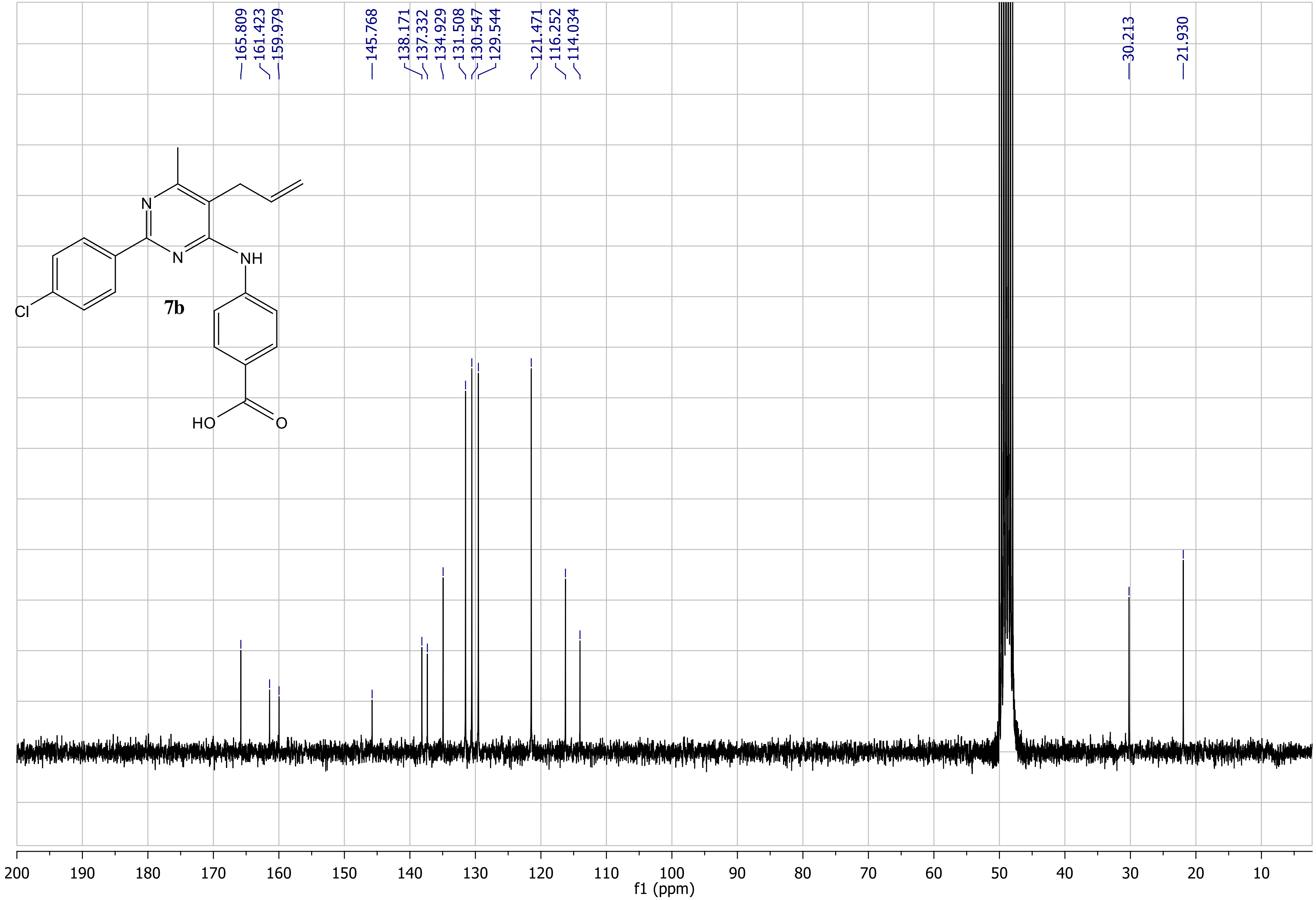

165.809
161.423
159.979
145.768
138.171
137.332
134.929
131.508
130.547
129.544
121.471
116.252
114.034
30.213
21.930
N
N
NH
Cl
7b
HO
O
200 190 180 170 160 150 140 130 120 110 100 90 80 70 60 50 40 30 20 10
f1 (ppm)

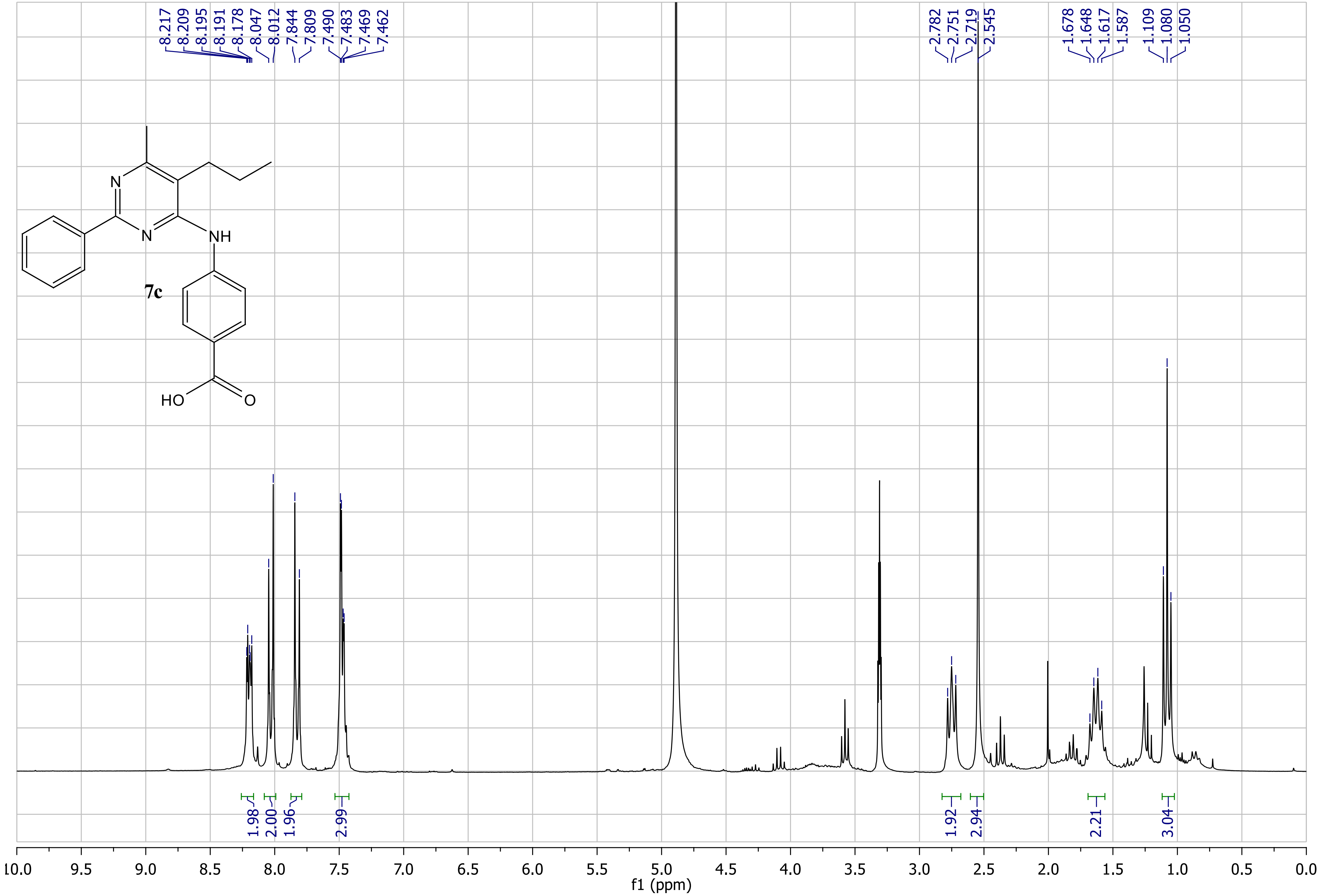
8.217
8.209
8.195
8.191
8.178
8.047
8.012
7.844
7.809
7.490
7.483
7.469
7.462
2.782
2.751
2.719
2.545
1.678
1.648
1.617
1.587
1.109
1.080
1.050
N
N
NH
7c
HO
O
1.98
2.00
1.96
2.99
1.92
2.94
2.21
3.04
10.0
9.5
9.0
8.5
8.0
7.5
7.0
6.5
6.0
5.5
5.0
4.5
4.0
3.5
3.0
2.5
2.0
1.5
1.0
0.5
0.0
f1 (ppm)

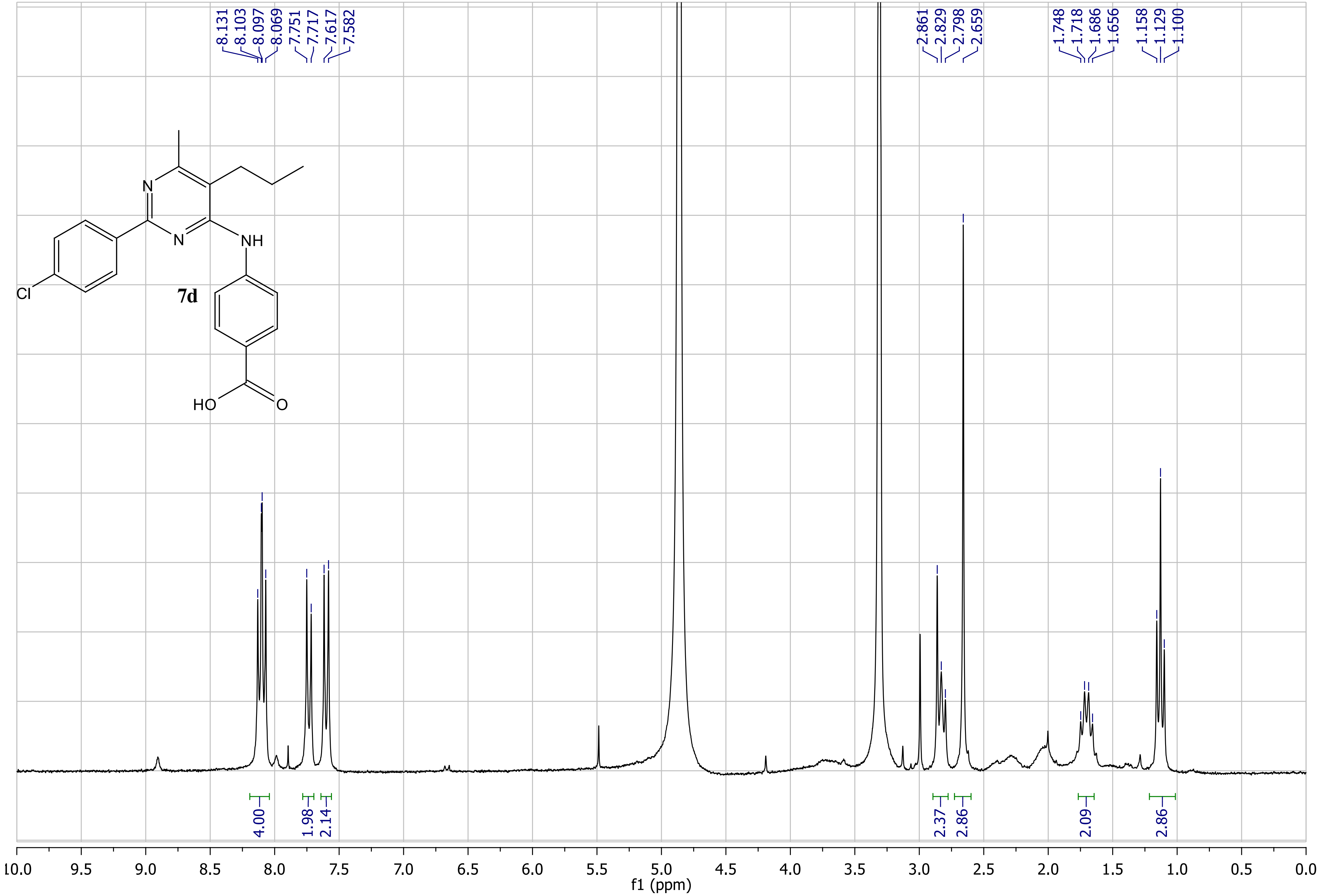

8.131
8.103
8.097
8.069
7.751
7.717
7.617
7.582
2.861
2.829
2.798
2.659
1.748
1.718
1.686
1.656
1.158
1.129
1.100
N
N
NH
Cl
7d
HO
O
4.00
1.98
2.14
2.37
2.86
2.09
2.86
10.0
9.5
9.0
8.5
8.0
7.5
7.0
6.5
6.0
5.5
5.0
4.5
4.0
3.5
3.0
2.5
2.0
1.5
1.0
0.5
0.0
f1 (ppm)

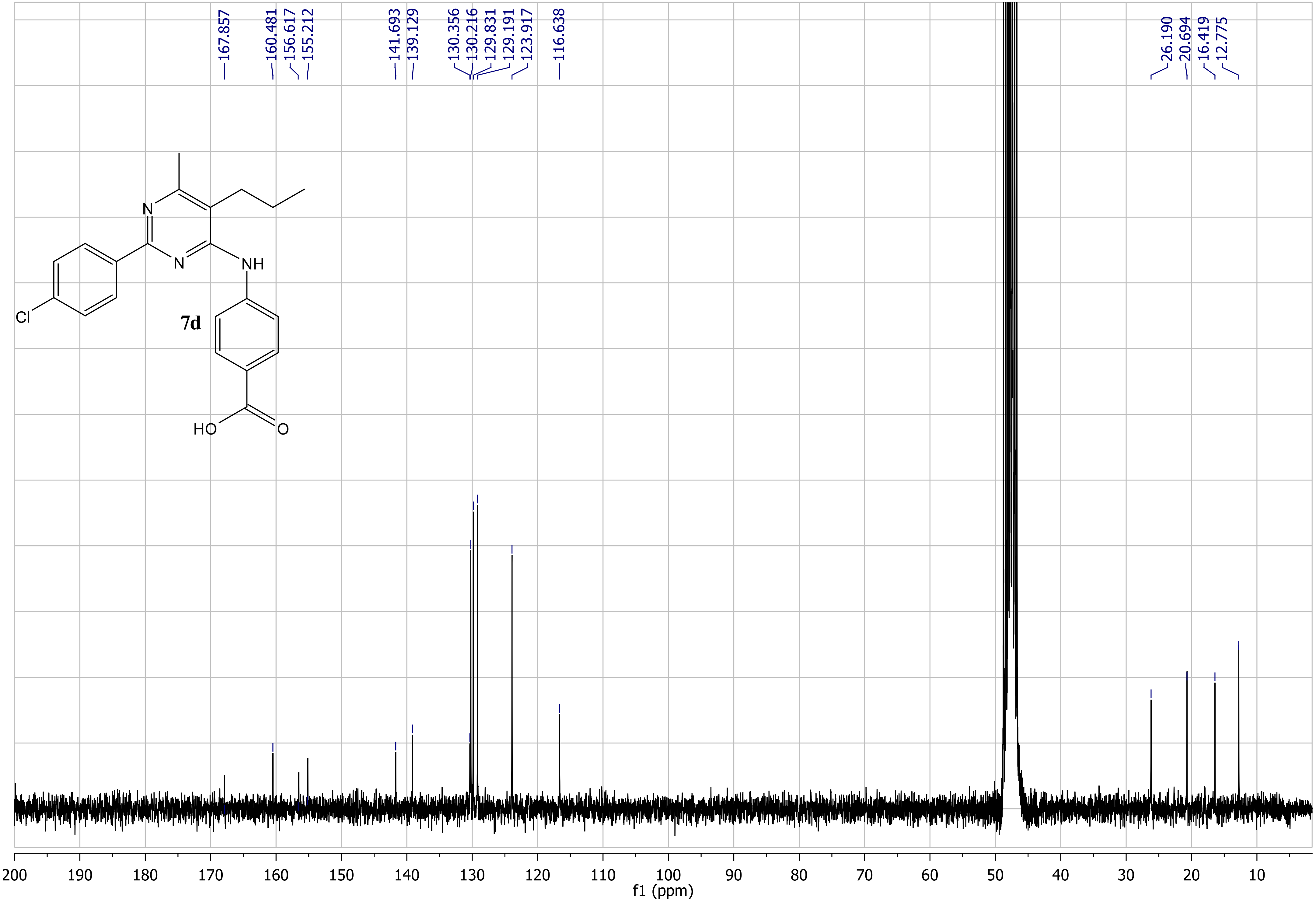

167.857
160.481
156.617
155.212
141.693
139.129
130.356
130.216
129.831
129.191
123.917
116.638
26.190
20.694
16.419
12.775
N
N
NH
Cl
HO
O
7d
200
190
180
170
160
150
140
130
120
110
100
90
80
70
60
50
40
30
20
10
f1 (ppm)

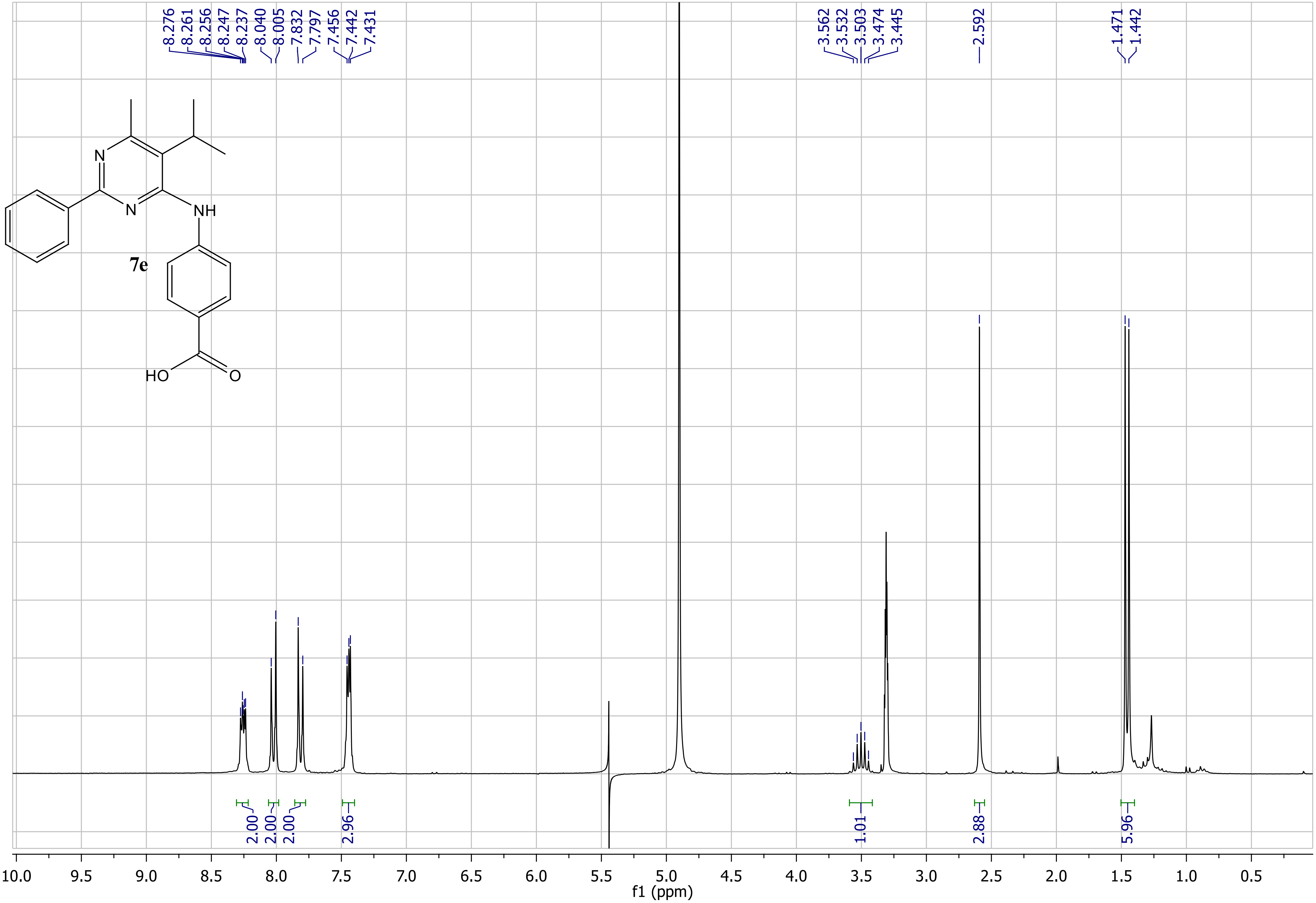
8.276
8.261
8.256
8.247
8.237
8.040
8.005
7.832
7.797
7.456
7.442
7.431
3.562
3.532
3.503
3.474
3.445
2.592
1.471
1.442
N
N
NH
HO
O
7e
2.00
2.00
2.00
2.96
1.01
2.88
5.96
10.0
9.5
9.0
8.5
8.0
7.5
7.0
6.5
6.0
5.5
5.0
4.5
4.0
3.5
3.0
2.5
2.0
1.5
1.0
0.5
f1 (ppm)

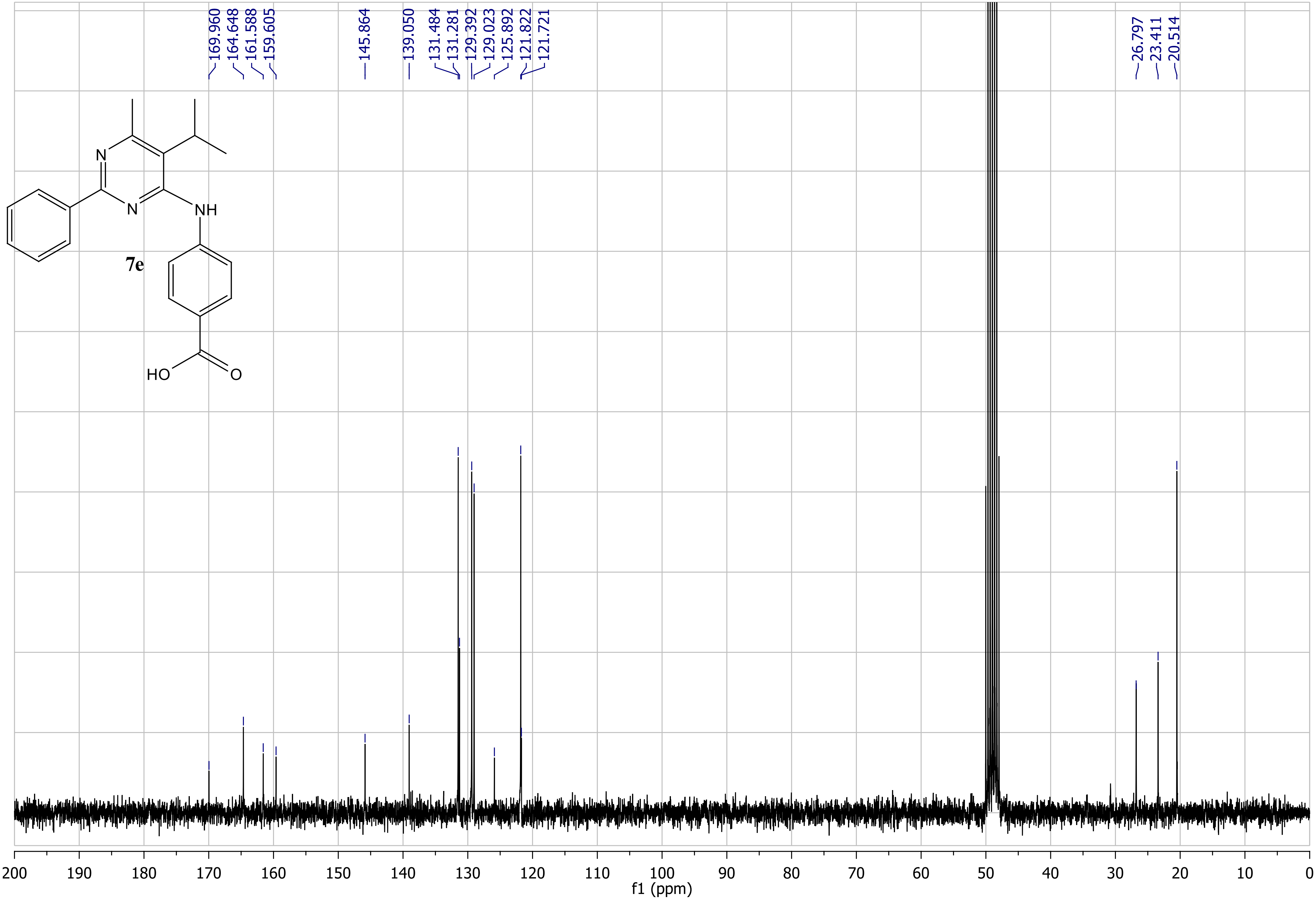
169.960
164.648
161.588
159.605
145.864
139.050
131.484
131.281
129.392
129.023
125.892
121.822
121.721
26.797
23.411
20.514
N
N
NH
HO
O
7e
200
190
180
170
160
150
140
130
120
110
100
90
80
70
60
50
40
30
20
10
0
f1 (ppm)

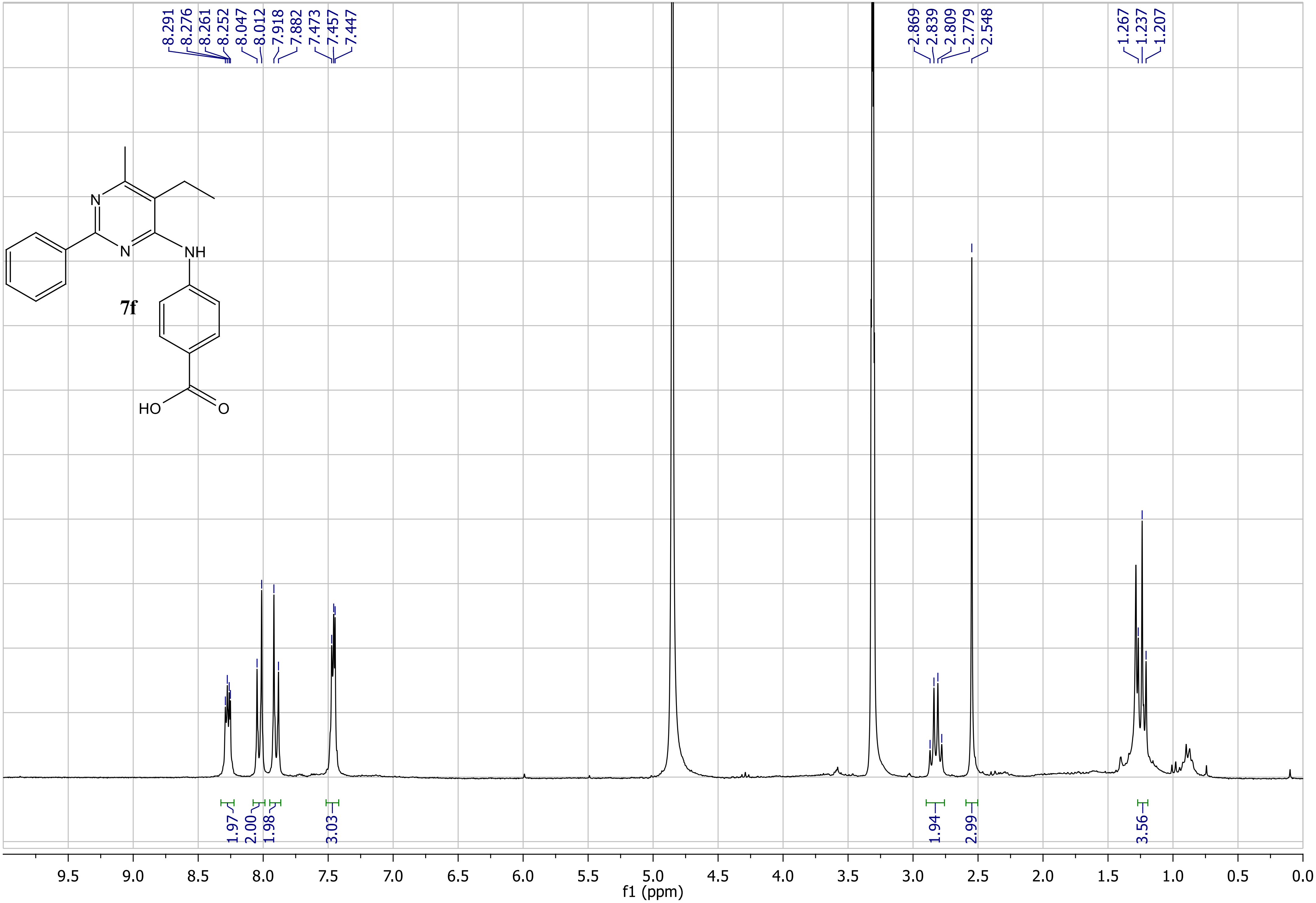
8.291
8.276
8.261
8.252
8.047
8.012
7.918
7.882
7.473
7.457
7.447
2.869
2.839
2.809
2.779
2.548
1.267
1.237
1.207
N
N
NH
7f
HO
O
1.97
2.00
1.98
3.03
1.94
2.99
3.56
9.5
9.0
8.5
8.0
7.5
7.0
6.5
6.0
5.5
5.0
4.5
4.0
3.5
3.0
2.5
2.0
1.5
1.0
0.5
0.0
f1 (ppm)

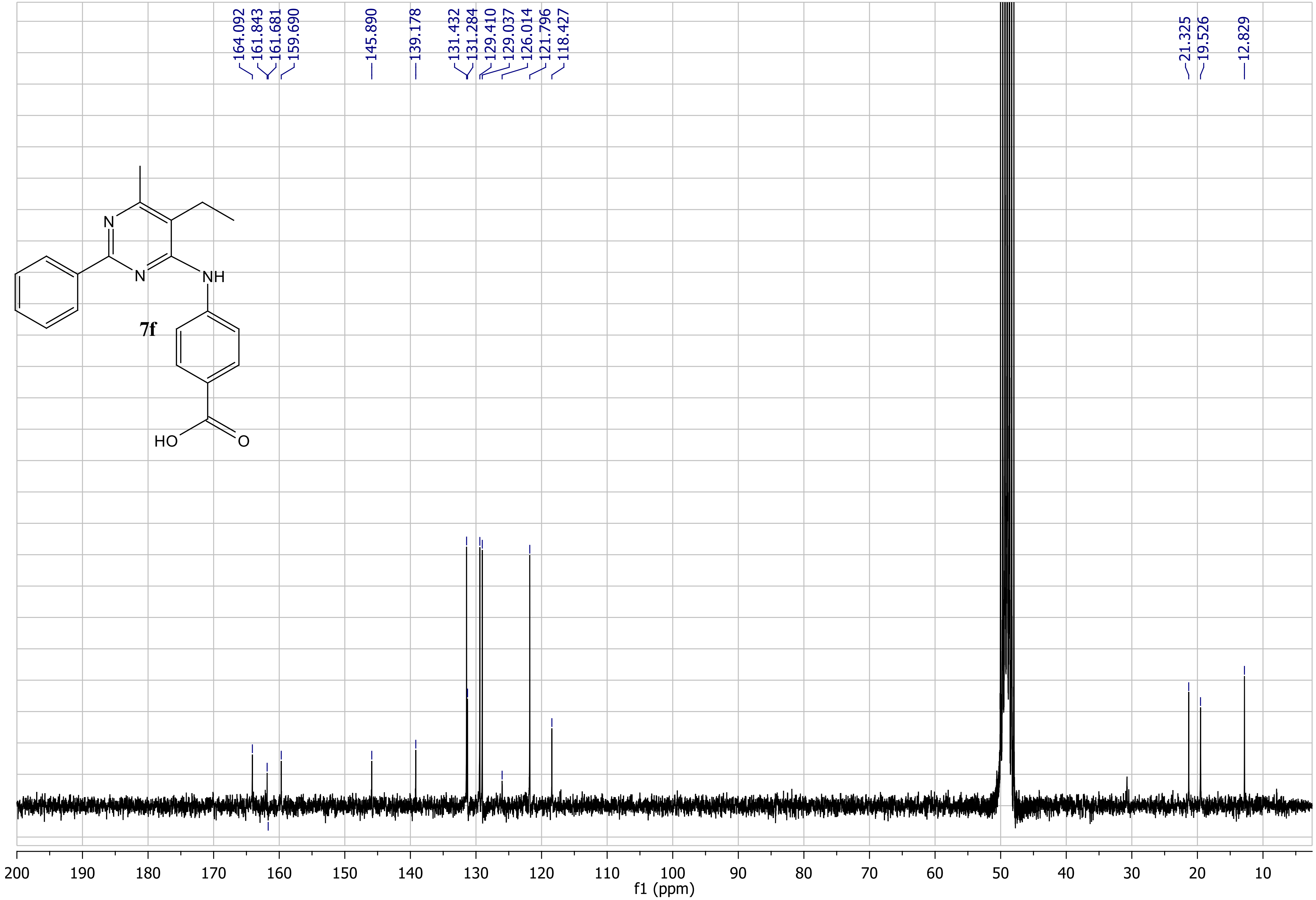
164.092
161.843
161.681
159.690
145.890
139.178
131.432
131.284
129.410
129.037
126.014
121.796
118.427
21.325
19.526
12.829
N
N
NH
HO
O
7f
200 190 180 170 160 150 140 130 120 110 100 90 80 70 60 50 40 30 20 10
f1 (ppm)

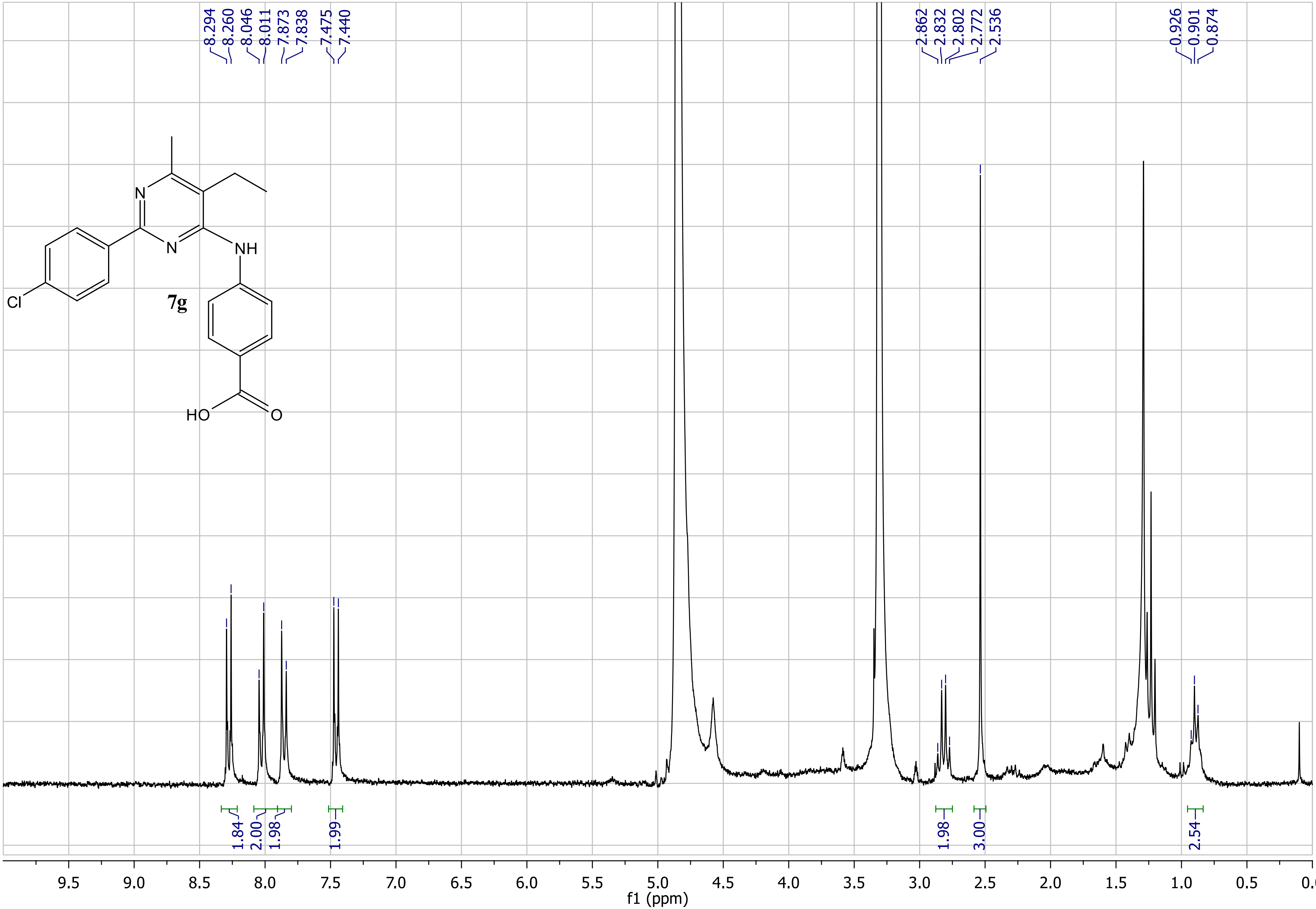
8.294
8.260
8.046
8.011
7.873
7.838
7.475
7.440
2.862
2.832
2.802
2.772
2.536
0.926
0.901
0.874
N
N
NH
Cl
7g
HO
O
1.84
2.00
1.98
1.99
1.98
3.00
2.54
9.5 9.0 8.5 8.0 7.5 7.0 6.5 6.0 5.5 5.0 4.5 4.0 3.5 3.0 2.5 2.0 1.5 1.0 0.5 0.0
f1 (ppm)

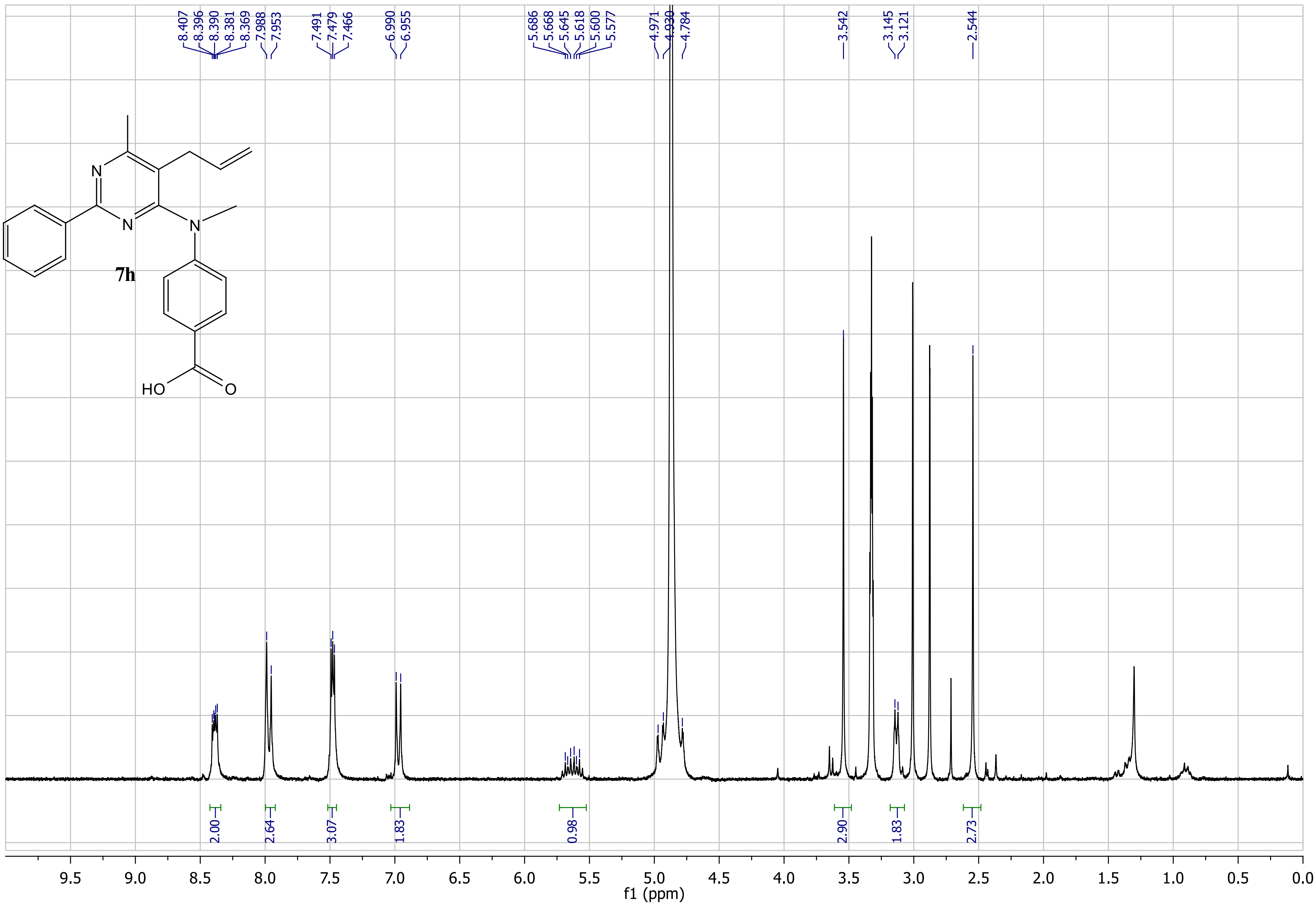
8.407
8.396
8.390
8.381
8.369
7.988
7.953
7.491
7.479
7.466
6.990
6.955
5.686
5.668
5.645
5.618
5.600
5.577
4.971
4.939
4.784
3.542
3.145
3.121
2.544
N
N
N
HO
O
7h
2.00
2.64
3.07
1.83
0.98
2.90
1.83
2.73
9.5
9.0
8.5
8.0
7.5
7.0
6.5
6.0
5.5
5.0
4.5
4.0
3.5
3.0
2.5
2.0
1.5
1.0
0.5
0.0
f1 (ppm)

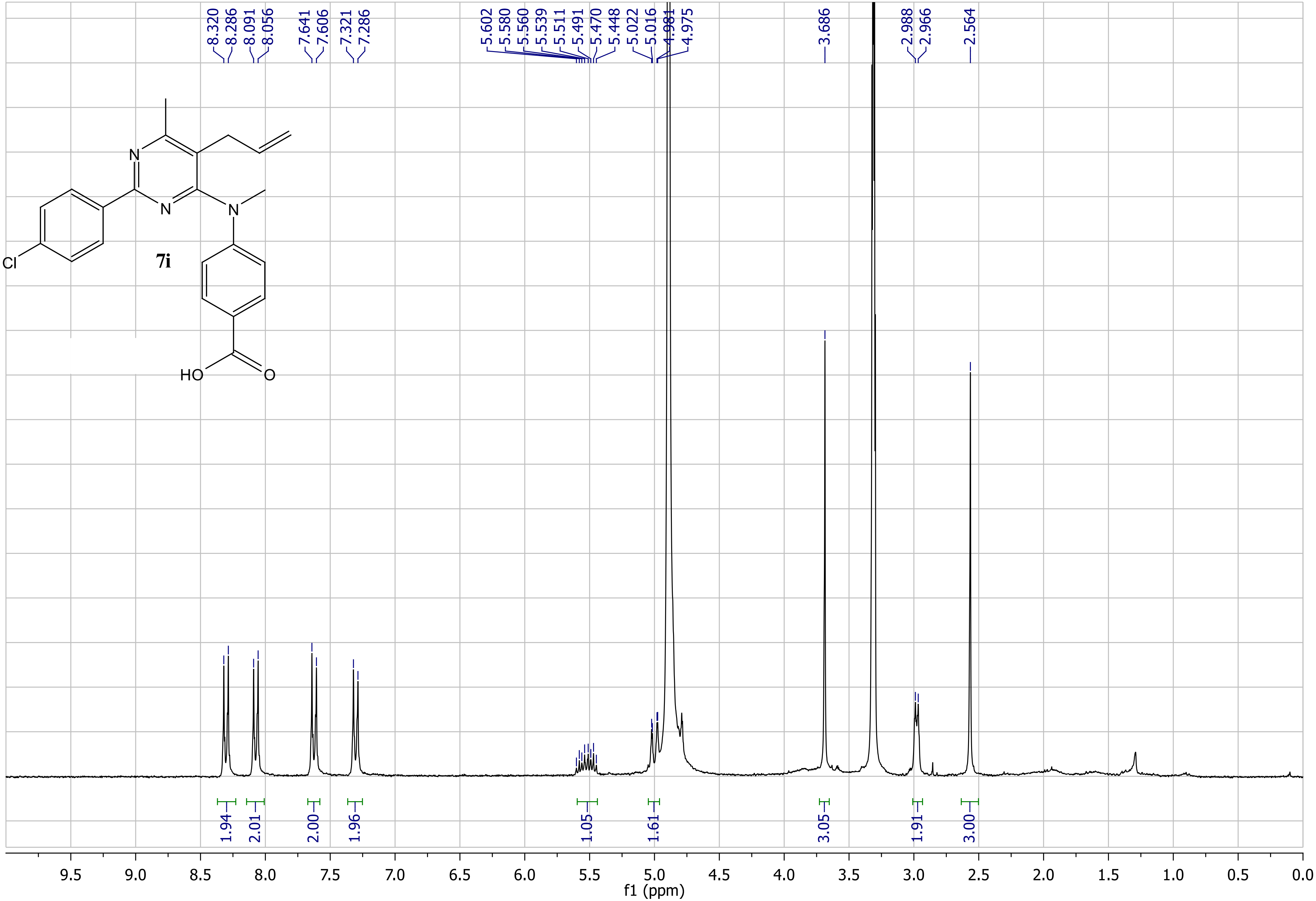
8.320
8.286
8.091
8.056
7.641
7.606
7.321
7.286
5.602
5.580
5.560
5.539
5.511
5.491
5.470
5.448
5.022
5.016
4.981
4.975
3.686
2.988
2.966
2.564
N
N
N
Cl
HO
O
7i
1.94
2.01
2.00
1.96
1.05
1.61
3.05
1.91
3.00
9.5
9.0
8.5
8.0
7.5
7.0
6.5
6.0
5.5
5.0
4.5
4.0
3.5
3.0
2.5
2.0
1.5
1.0
0.5
0.0
f1 (ppm)

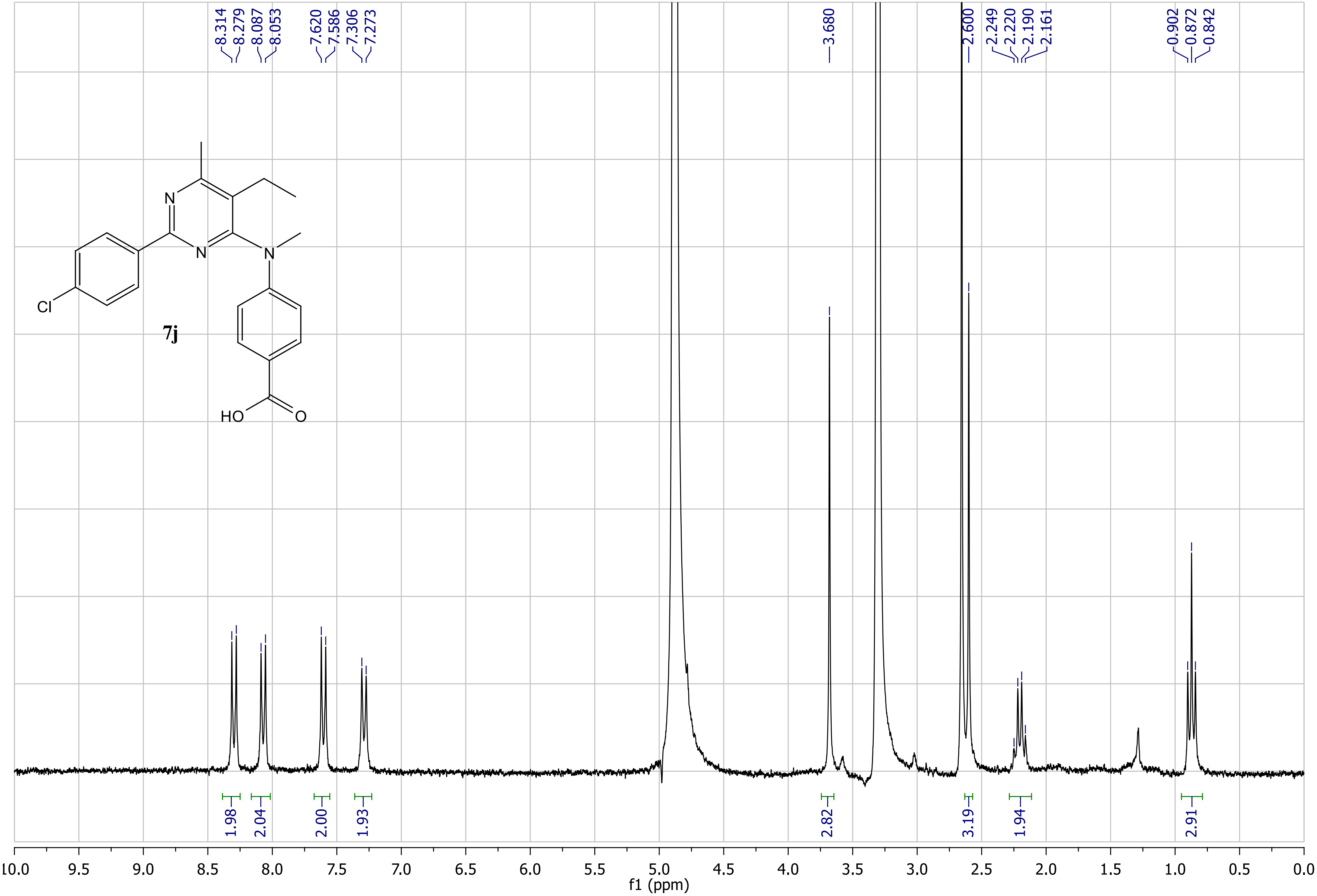

8.314
8.279
8.087
8.053
7.620
7.586
7.306
7.273
3.680
2.600
2.249
2.220
2.190
2.161
0.902
0.872
0.842
N
N
N
Cl
HO
O
7j
1.98
2.04
2.00
1.93
2.82
3.19
1.94
2.91
10.0
9.5
9.0
8.5
8.0
7.5
7.0
6.5
6.0
5.5
5.0
4.5
4.0
3.5
3.0
2.5
2.0
1.5
1.0
0.5
0.0
f1 (ppm)

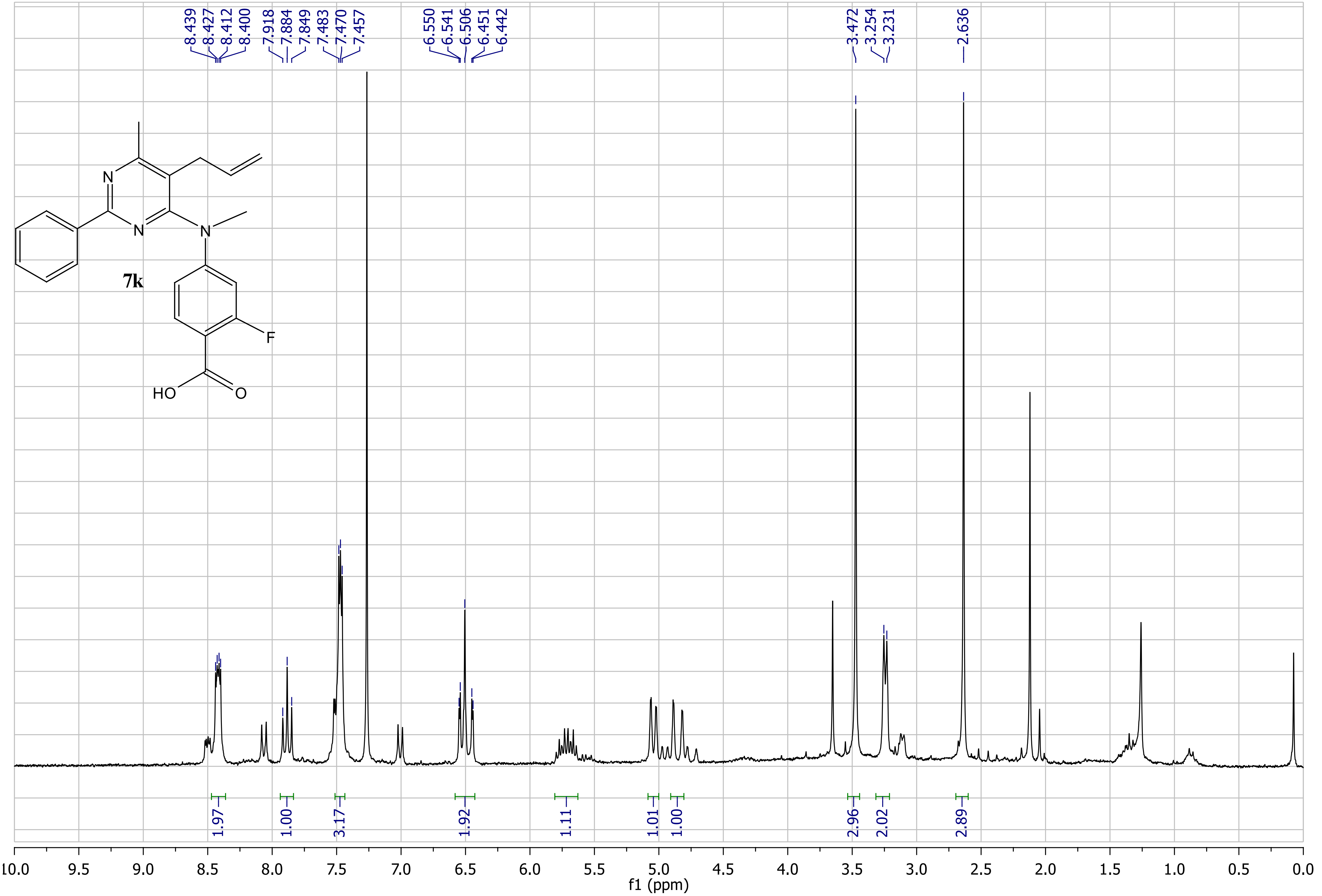
8.439
8.427
8.412
8.400
7.918
7.884
7.849
7.483
7.470
7.457
6.550
6.541
6.506
6.451
6.442
3.472
3.254
3.231
2.636
N
N
N
F
HO
O
7k
1.97
1.00
3.17
1.92
1.11
1.01
1.00
2.96
2.02
2.89
10.0
9.5
9.0
8.5
8.0
7.5
7.0
6.5
6.0
5.5
5.0
4.5
4.0
3.5
3.0
2.5
2.0
1.5
1.0
0.5
0.0
f1 (ppm)

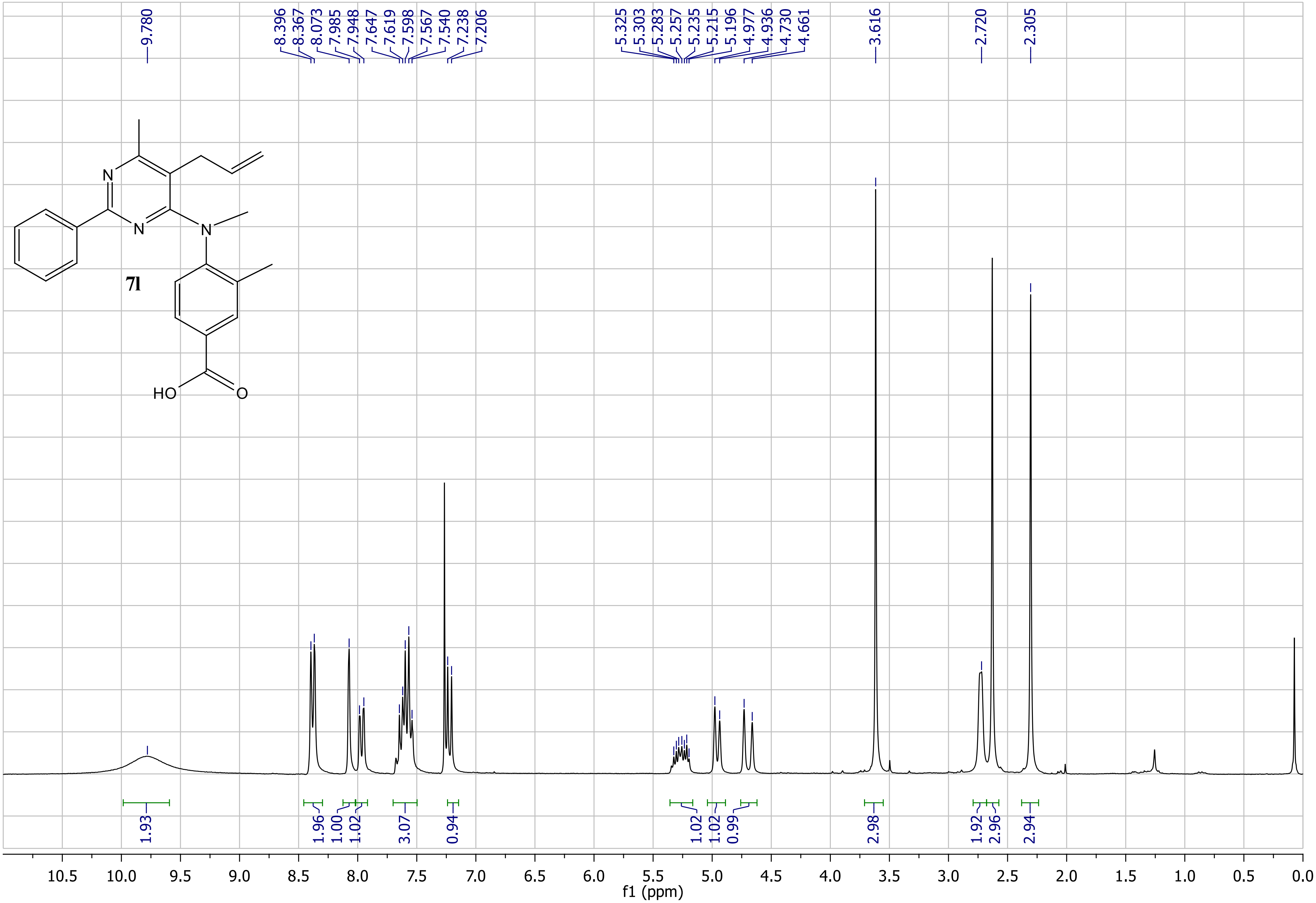
9.780
8.396
8.367
8.073
7.985
7.948
7.647
7.619
7.598
7.567
7.540
7.238
7.206
5.325
5.303
5.283
5.257
5.235
5.215
5.196
4.977
4.936
4.730
4.661
3.616
2.720
2.305
N
N
N
HO
O
7l
1.93
1.96
1.00
1.02
3.07
0.94
1.02
1.02
0.99
2.98
1.92
2.96
2.94
10.5
10.0
9.5
9.0
8.5
8.0
7.5
7.0
6.5
6.0
5.5
5.0
4.5
4.0
3.5
3.0
2.5
2.0
1.5
1.0
0.5
0.0
f1 (ppm)

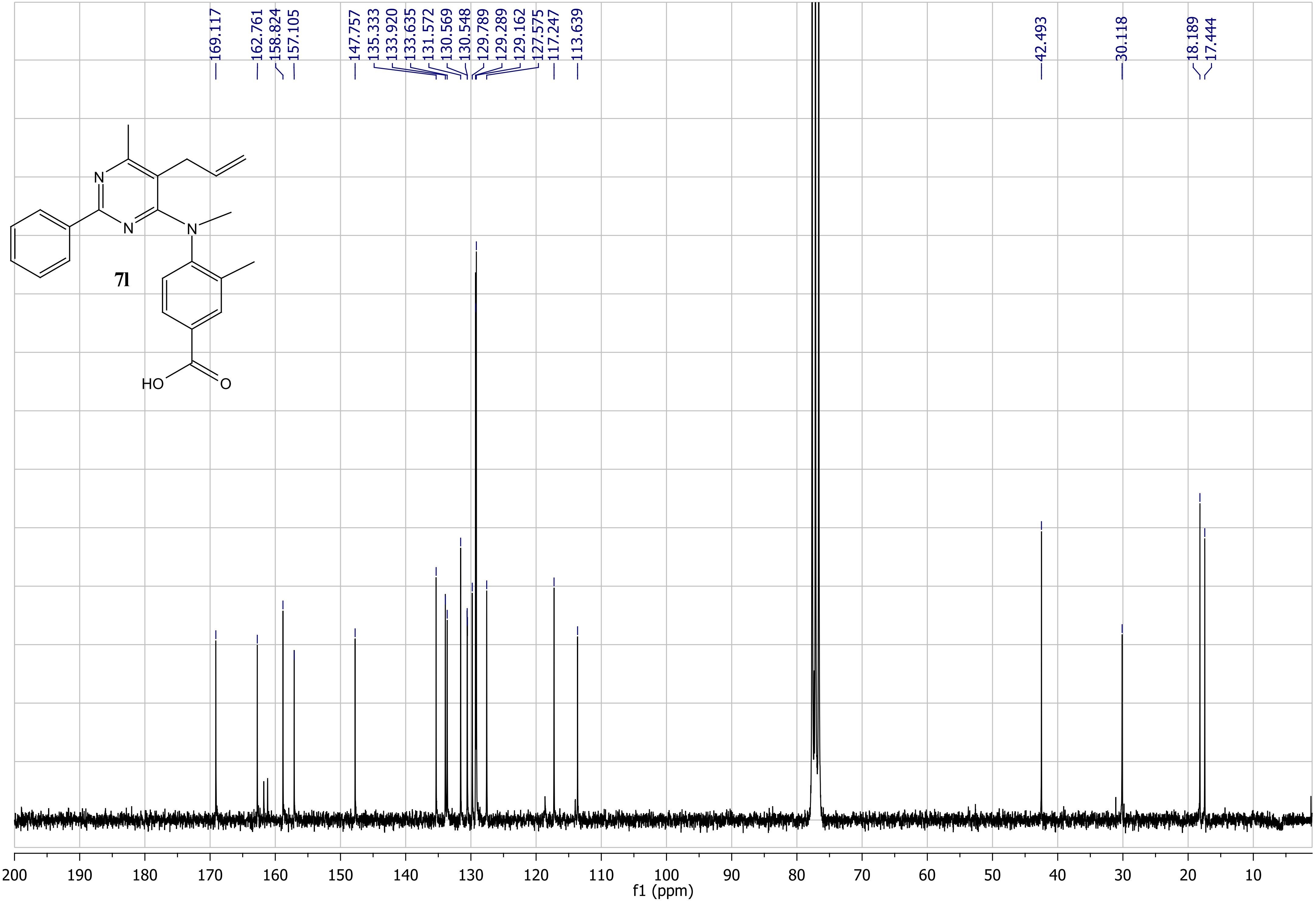
169.117
162.761
158.824
157.105
147.757
135.333
133.920
133.635
131.572
130.569
130.548
129.789
129.289
129.162
127.575
117.247
113.639
42.493
30.118
18.189
17.444
N
N
N
HO
O
7l
200 190 180 170 160 150 140 130 120 110 100 90 80 70 60 50 40 30 20 10
f1 (ppm)

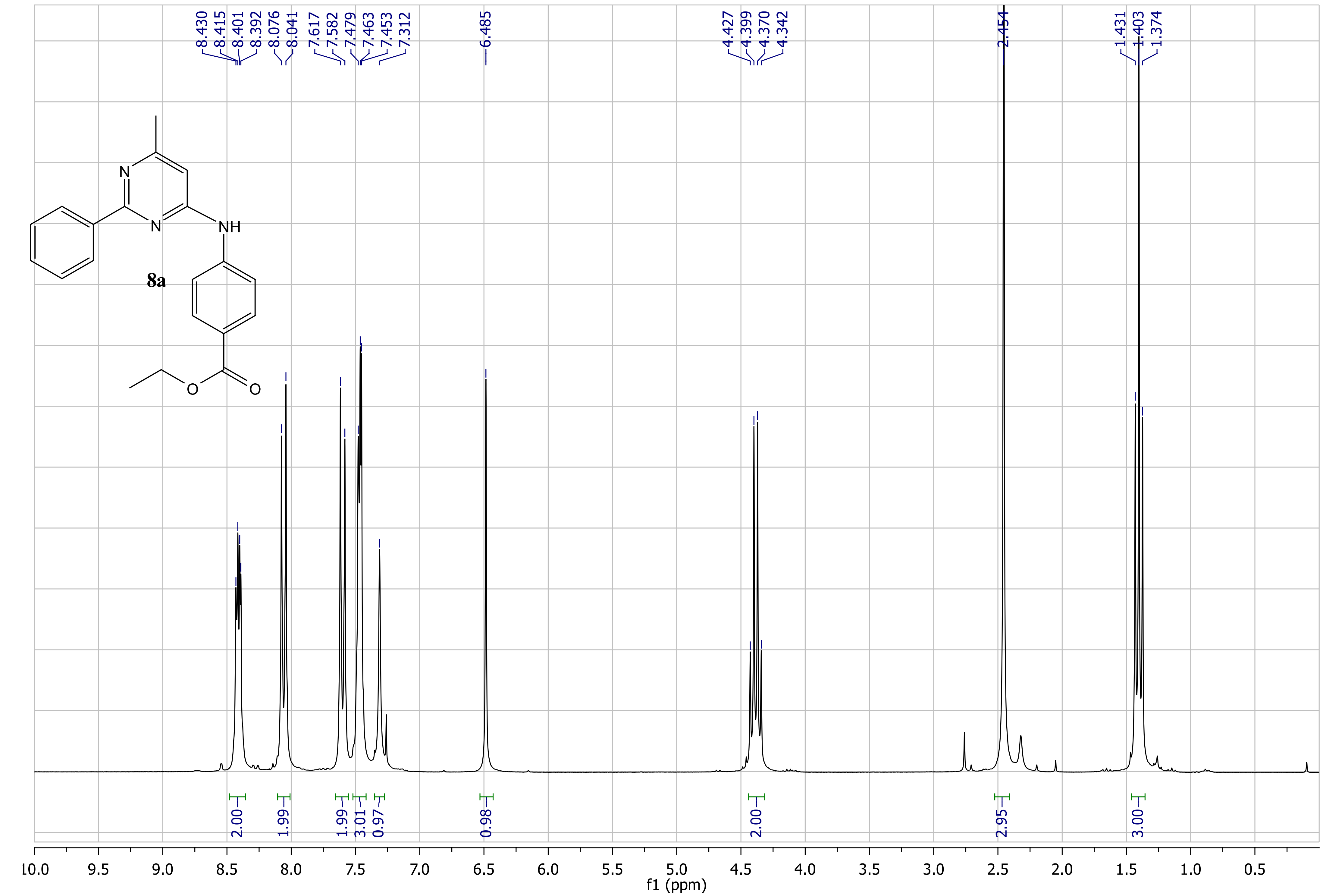
8.430
8.415
8.401
8.392
8.076
8.041
7.617
7.582
7.479
7.463
7.453
7.312
6.485
4.427
4.399
4.370
4.342
2.454
1.431
1.403
1.374
N
N
NH
O
O
8a
2.00
1.99
1.99
3.01
0.97
0.98
2.00
2.95
3.00
10.0
9.5
9.0
8.5
8.0
7.5
7.0
6.5
6.0
5.5
5.0
4.5
4.0
3.5
3.0
2.5
2.0
1.5
1.0
0.5
f1 (ppm)

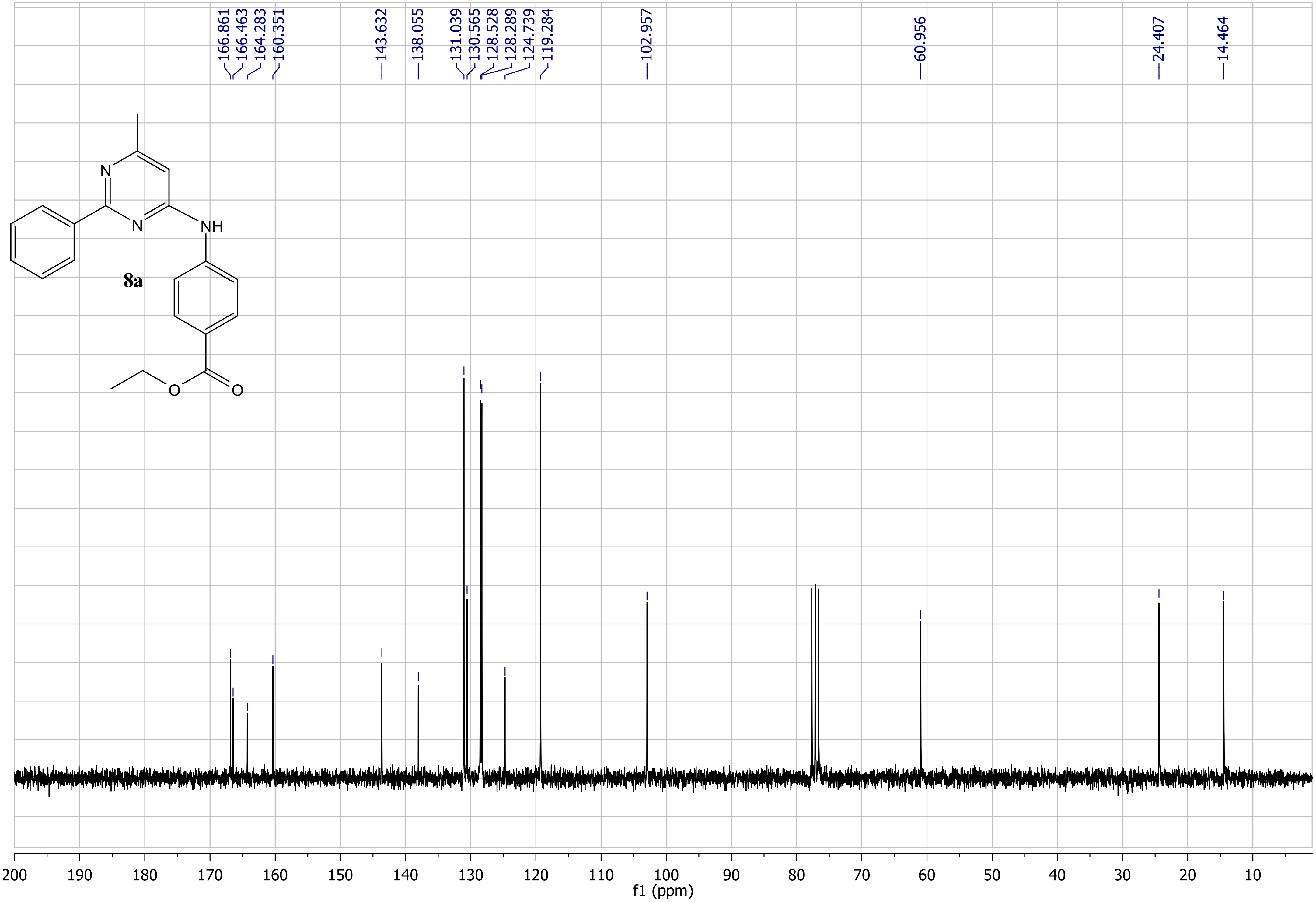
166.861
166.463
164.283
160.351
143.632
138.055
131.039
130.565
128.528
128.289
124.739
119.284
102.957
60.956
24.407
14.464
8a
N
N
NH
O
O
200 190 180 170 160 150 140 130 120 110 100 90 80 70 60 50 40 30 20 10
f1 (ppm)

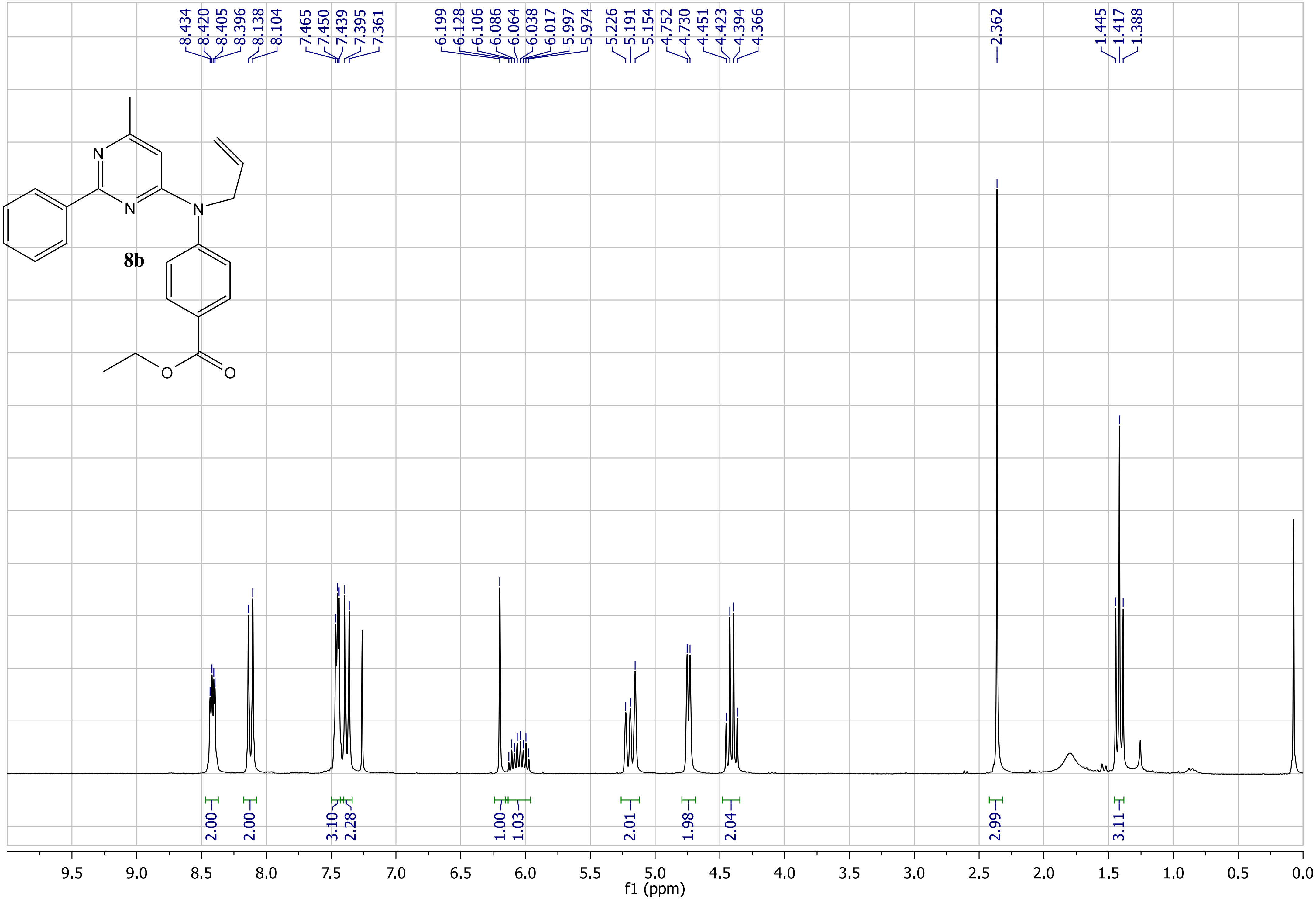
8.434
8.420
8.405
8.396
8.138
8.104
7.465
7.450
7.439
7.395
7.361
6.199
6.128
6.106
6.086
6.064
6.038
6.017
5.997
5.974
5.226
5.191
5.154
4.752
4.730
4.451
4.423
4.394
4.366
2.362
1.445
1.417
1.388
N
N
N
O
O
8b
2.00
2.00
3.10
2.28
1.00
1.03
2.01
1.98
2.04
2.99
3.11
9.5
9.0
8.5
8.0
7.5
7.0
6.5
6.0
5.5
5.0
4.5
4.0
3.5
3.0
2.5
2.0
1.5
1.0
0.5
0.0
f1 (ppm)

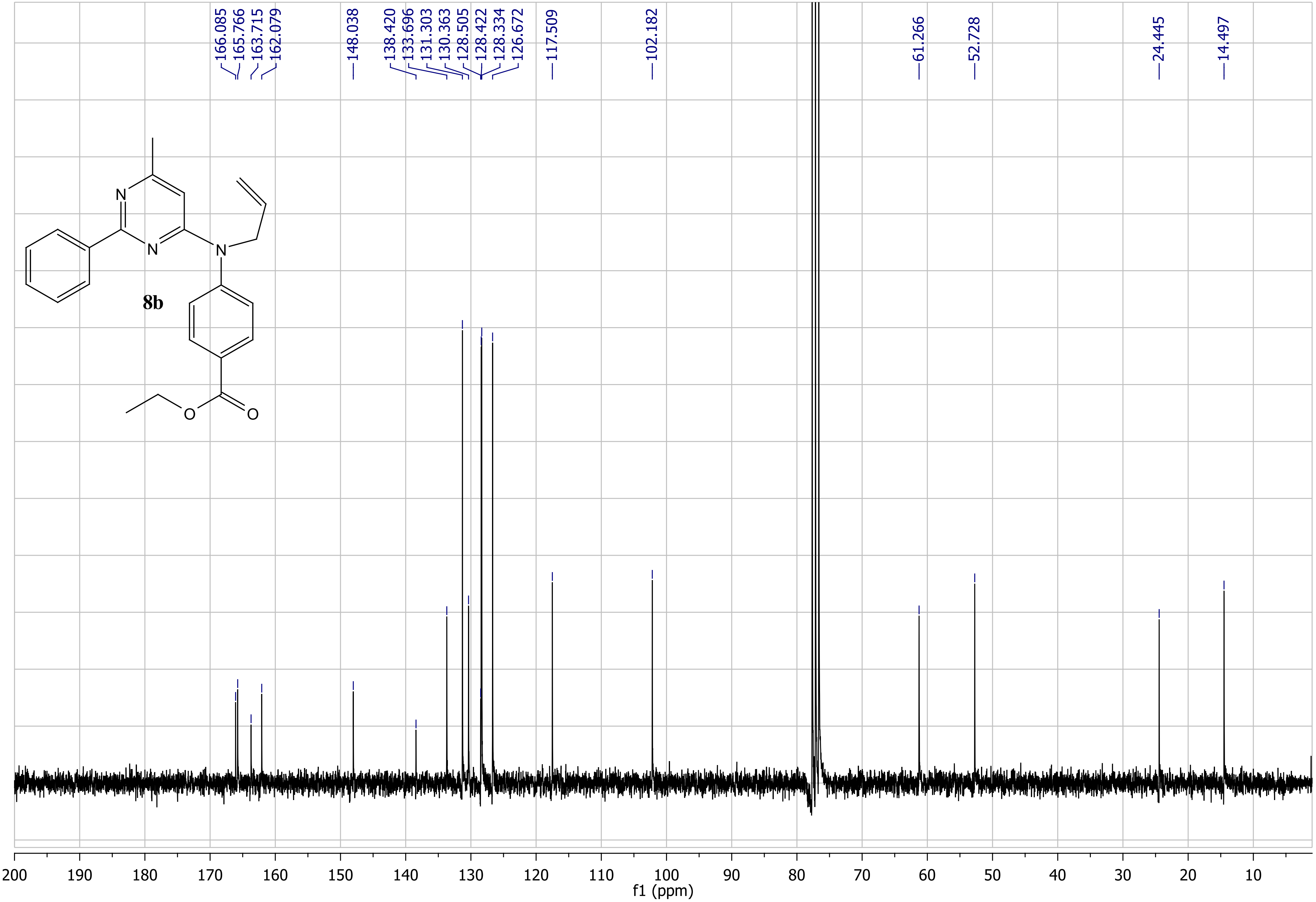
166.085
165.766
163.715
162.079
148.038
138.420
133.696
131.303
130.363
128.505
128.422
128.334
126.672
117.509
102.182
61.266
52.728
24.445
14.497
N
N
N
O
O
8b
200 190 180 170 160 150 140 130 120 110 100 90 80 70 60 50 40 30 20 10
f1 (ppm)

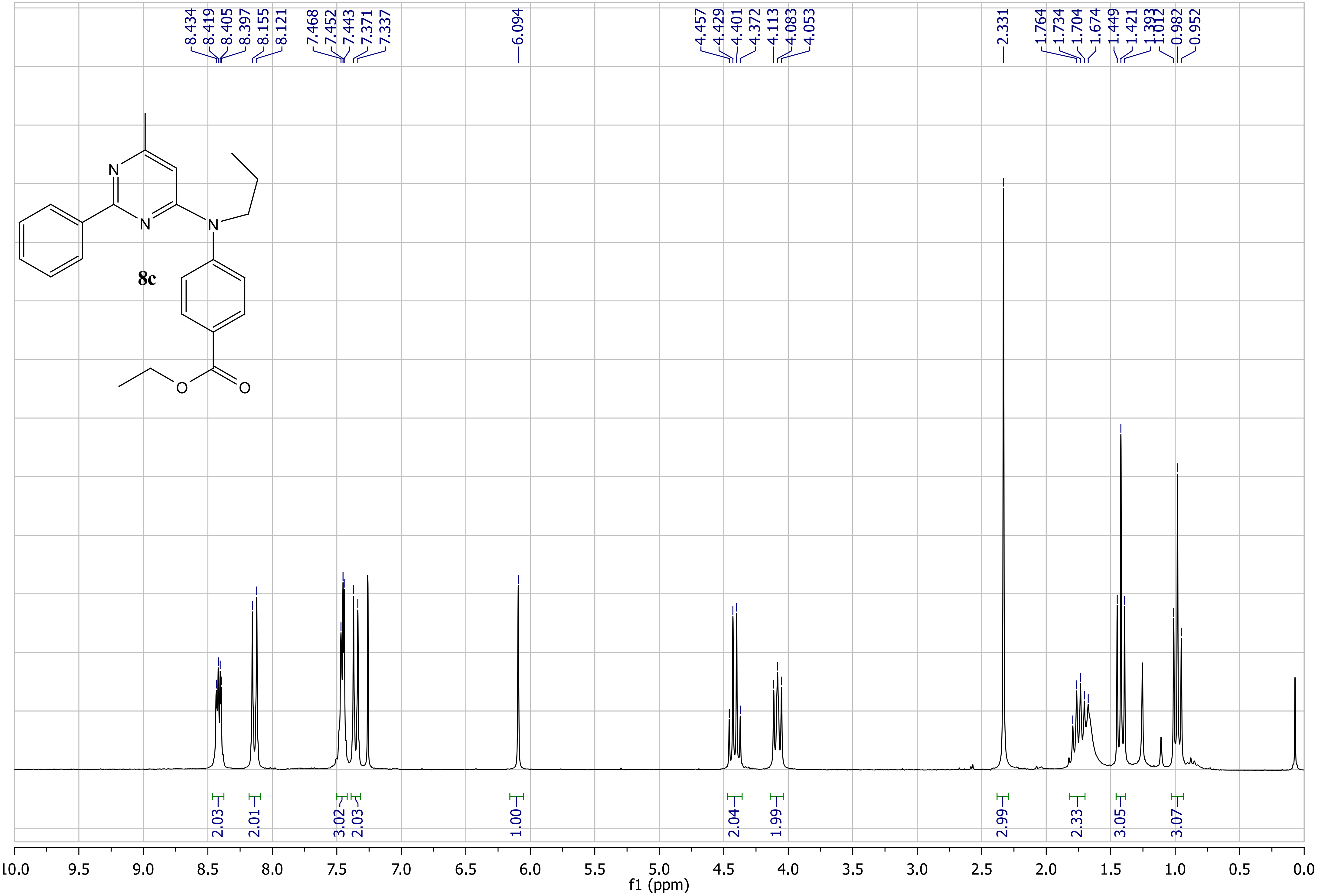
8.434
8.419
8.405
8.397
8.155
8.121
7.468
7.452
7.443
7.371
7.337
6.094
4.457
4.429
4.401
4.372
4.113
4.083
4.053
2.331
1.764
1.734
1.704
1.674
1.449
1.421
1.393
1.012
0.982
0.952
8c
2.03
2.01
3.02
2.03
1.00
2.04
1.99
2.99
2.33
3.05
3.07
10.0 9.5 9.0 8.5 8.0 7.5 7.0 6.5 6.0 5.5 5.0 4.5 4.0 3.5 3.0 2.5 2.0 1.5 1.0 0.5 0.0
f1 (ppm)

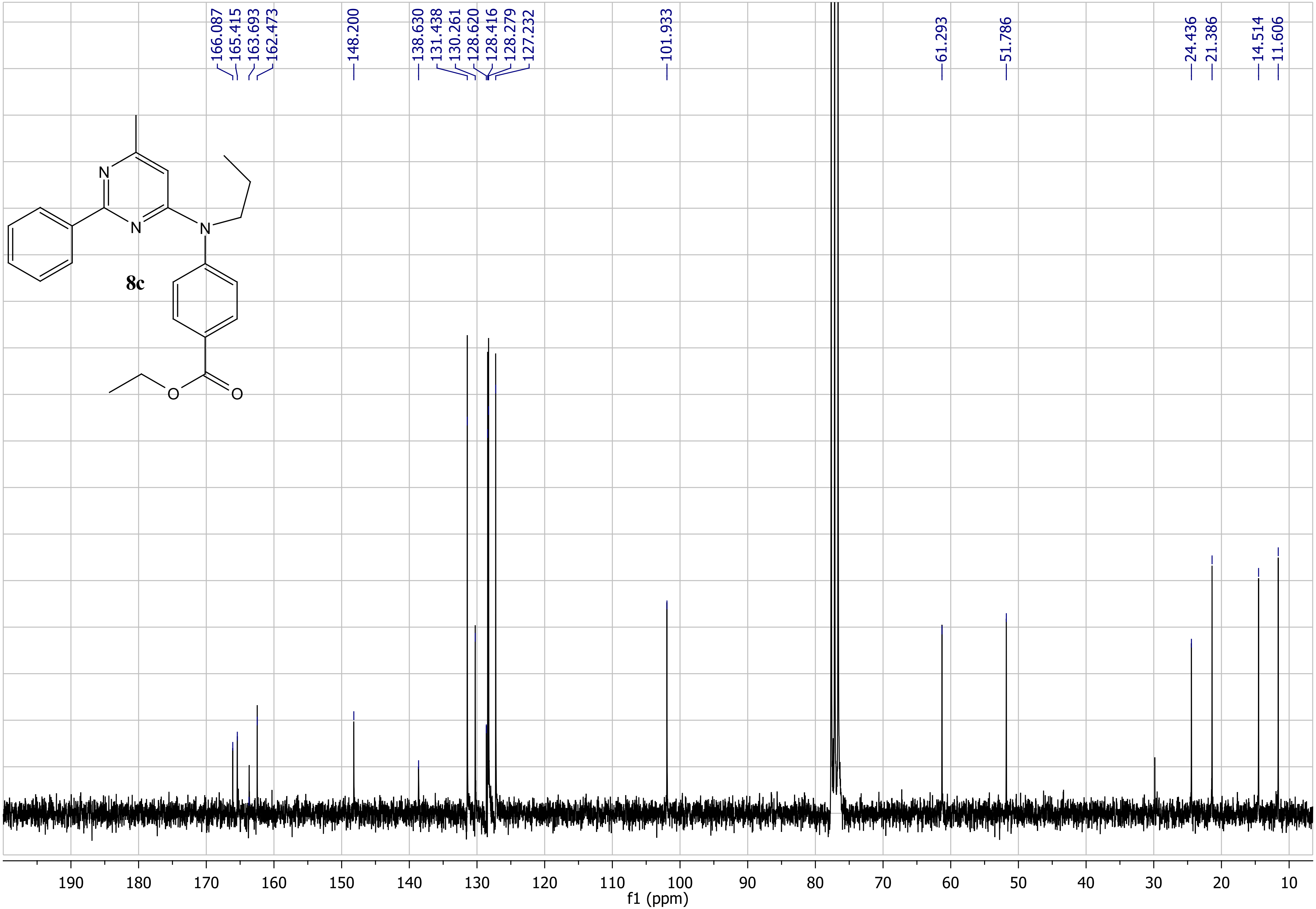

166.087
165.415
163.693
162.473
148.200
138.630
131.438
130.261
128.620
128.416
128.279
127.232
101.933
61.293
51.786
24.436
21.386
14.514
11.606
8c
f1 (ppm)

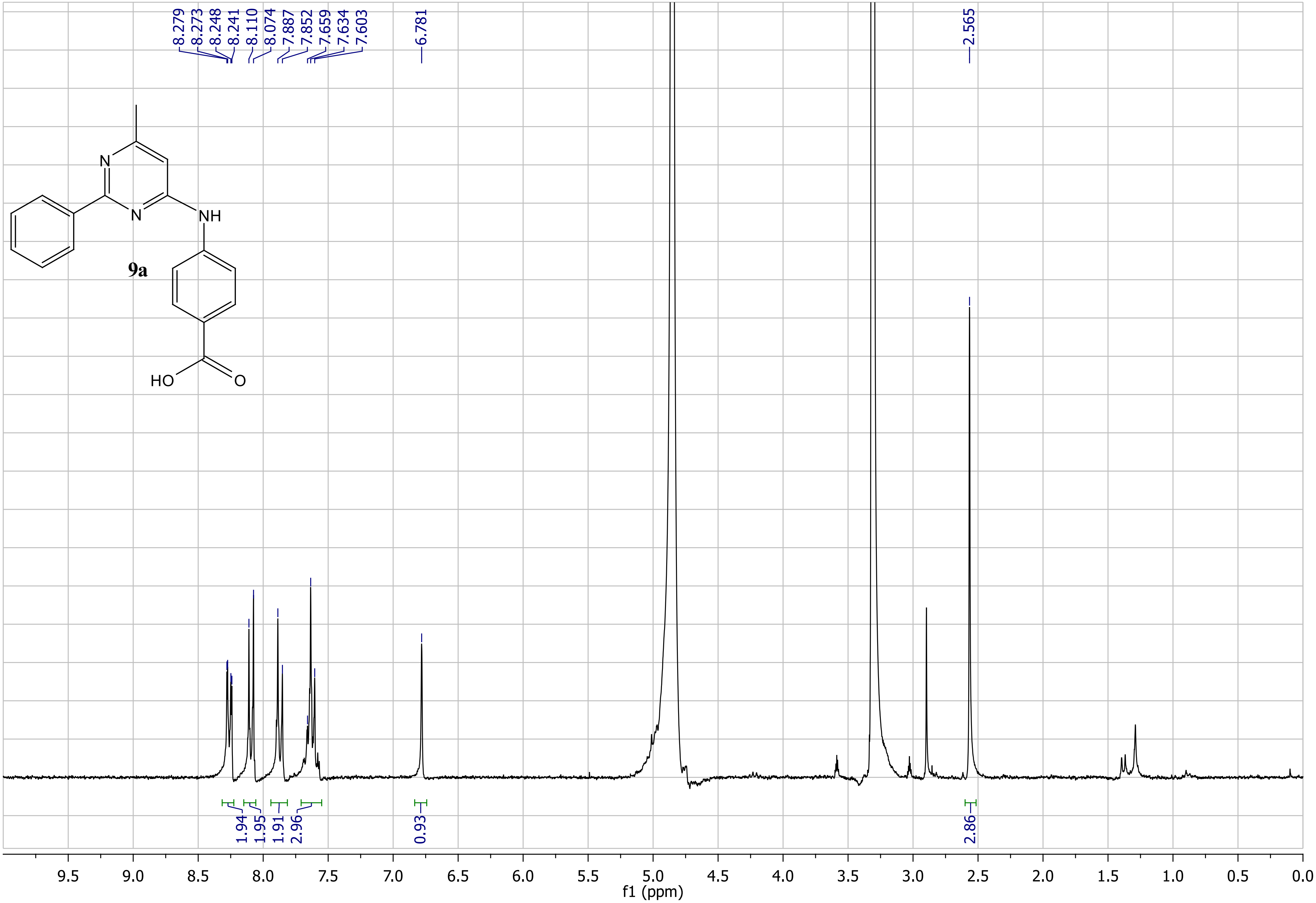
8.279
8.273
8.248
8.241
8.110
8.074
7.887
7.852
7.659
7.634
7.603
6.781
2.565
N
N
NH
HO
O
9a
1.94
1.95
1.91
2.96
0.93
2.86
9.5 9.0 8.5 8.0 7.5 7.0 6.5 6.0 5.5 5.0 4.5 4.0 3.5 3.0 2.5 2.0 1.5 1.0 0.5 0.0
f1 (ppm)

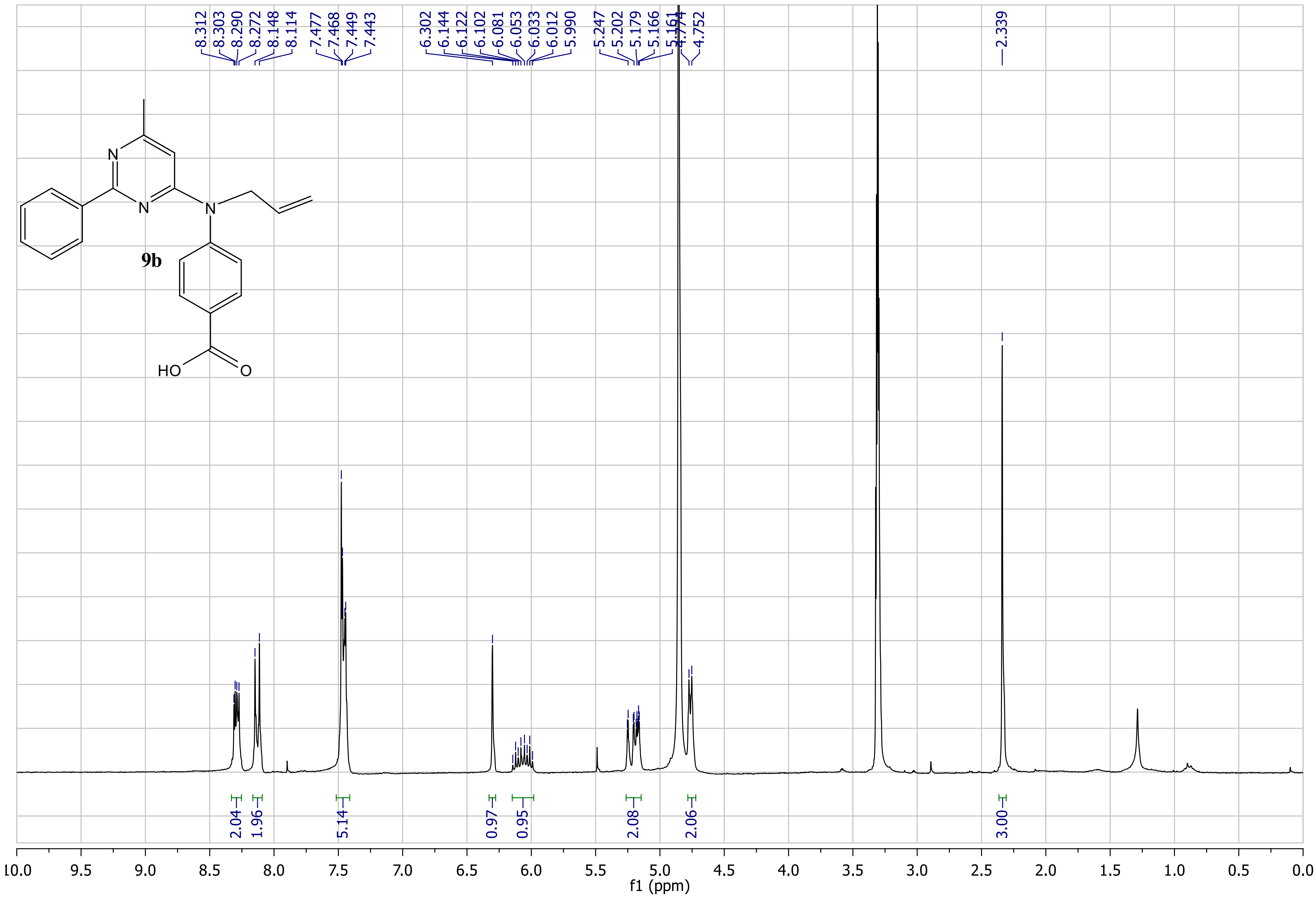

8.312
8.303
8.290
8.272
8.148
8.114
7.477
7.468
7.449
7.443
6.302
6.144
6.122
6.102
6.081
6.053
6.033
6.012
5.990
5.247
5.202
5.179
5.166
5.161
4.774
4.752
2.339
N
N
N
HO
O
9b
2.04
1.96
5.14
0.97
0.95
2.08
2.06
3.00
10.0
9.5
9.0
8.5
8.0
7.5
7.0
6.5
6.0
5.5
5.0
4.5
4.0
3.5
3.0
2.5
2.0
1.5
1.0
0.5
0.0
f1 (ppm)

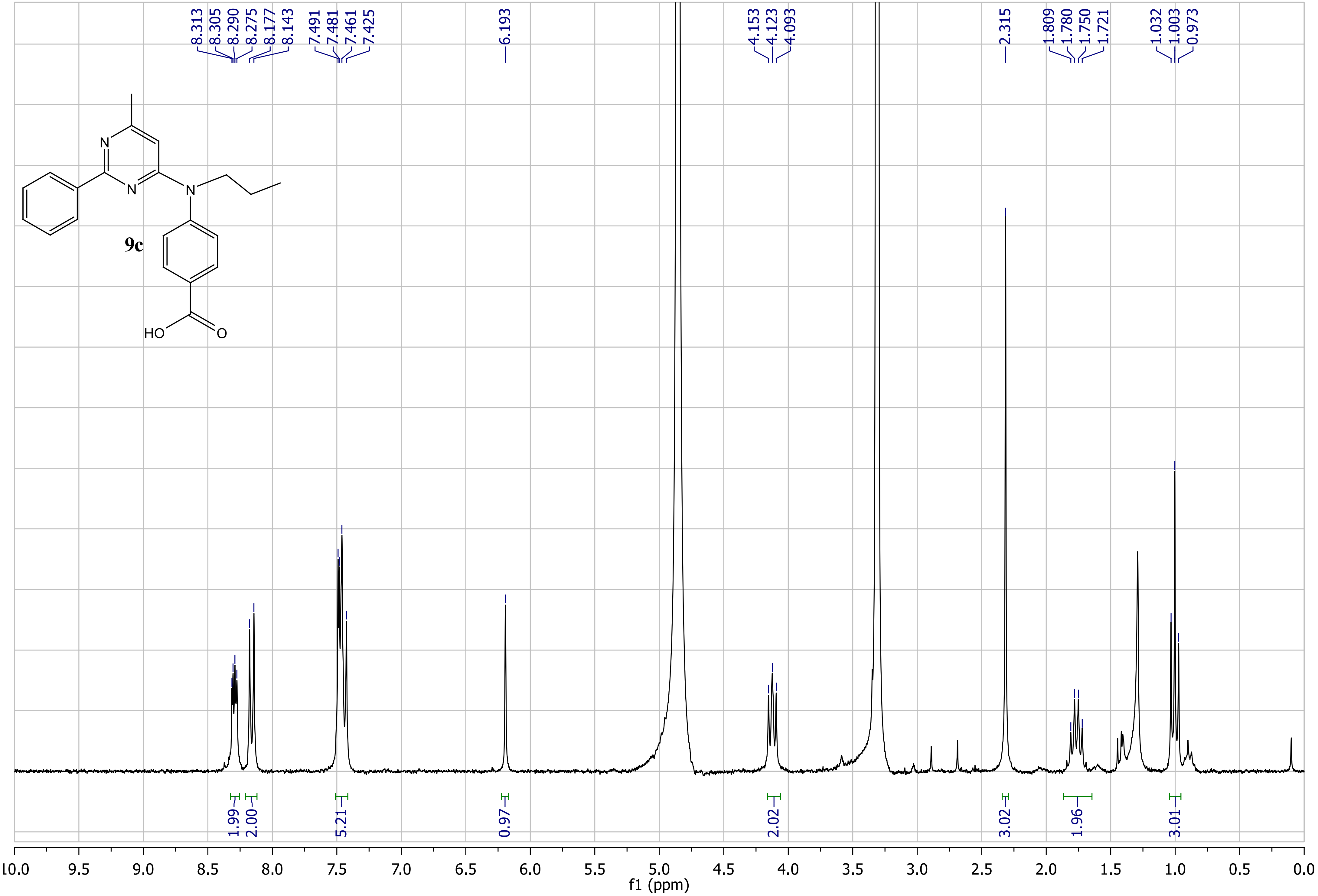
8.313
8.305
8.290
8.275
8.177
8.143
7.491
7.481
7.461
7.425
6.193
4.153
4.123
4.093
2.315
1.809
1.780
1.750
1.721
1.032
1.003
0.973
N
N
N
9c
HO
O
1.99
2.00
5.21
0.97
2.02
3.02
1.96
3.01
10.0
9.5
9.0
8.5
8.0
7.5
7.0
6.5
6.0
5.5
5.0
4.5
4.0
3.5
3.0
2.5
2.0
1.5
1.0
0.5
0.0
f1 (ppm)

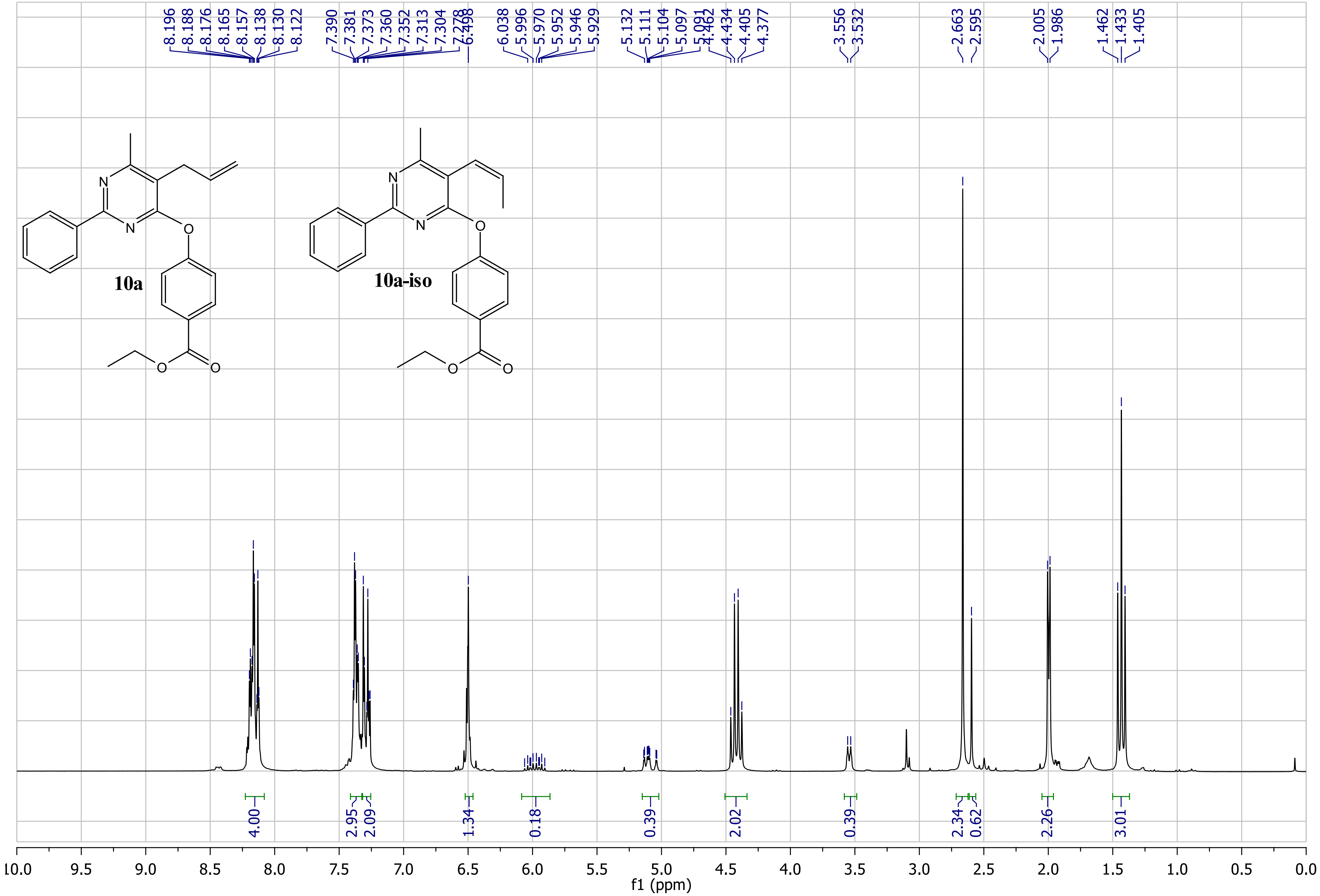
8.196 8.188 8.176 8.165 8.157 8.138 8.130 8.122
7.390 7.381 7.373 7.360 7.352 7.313 7.304 7.278 6.498
6.038 5.996 5.970 5.952 5.946 5.929
5.132 5.111 5.104 5.097 5.091 4.462 4.434 4.405 4.377
3.556 3.532
2.663 2.595
2.005 1.986
1.462 1.433 1.405
N
N
O
O
O
10a
10a-iso
4.00
2.95
2.09
1.34
0.18
0.39
2.02
0.39
2.34
0.62
2.26
3.01
10.0 9.5 9.0 8.5 8.0 7.5 7.0 6.5 6.0 5.5 5.0 4.5 4.0 3.5 3.0 2.5 2.0 1.5 1.0 0.5 0.0
f1 (ppm)

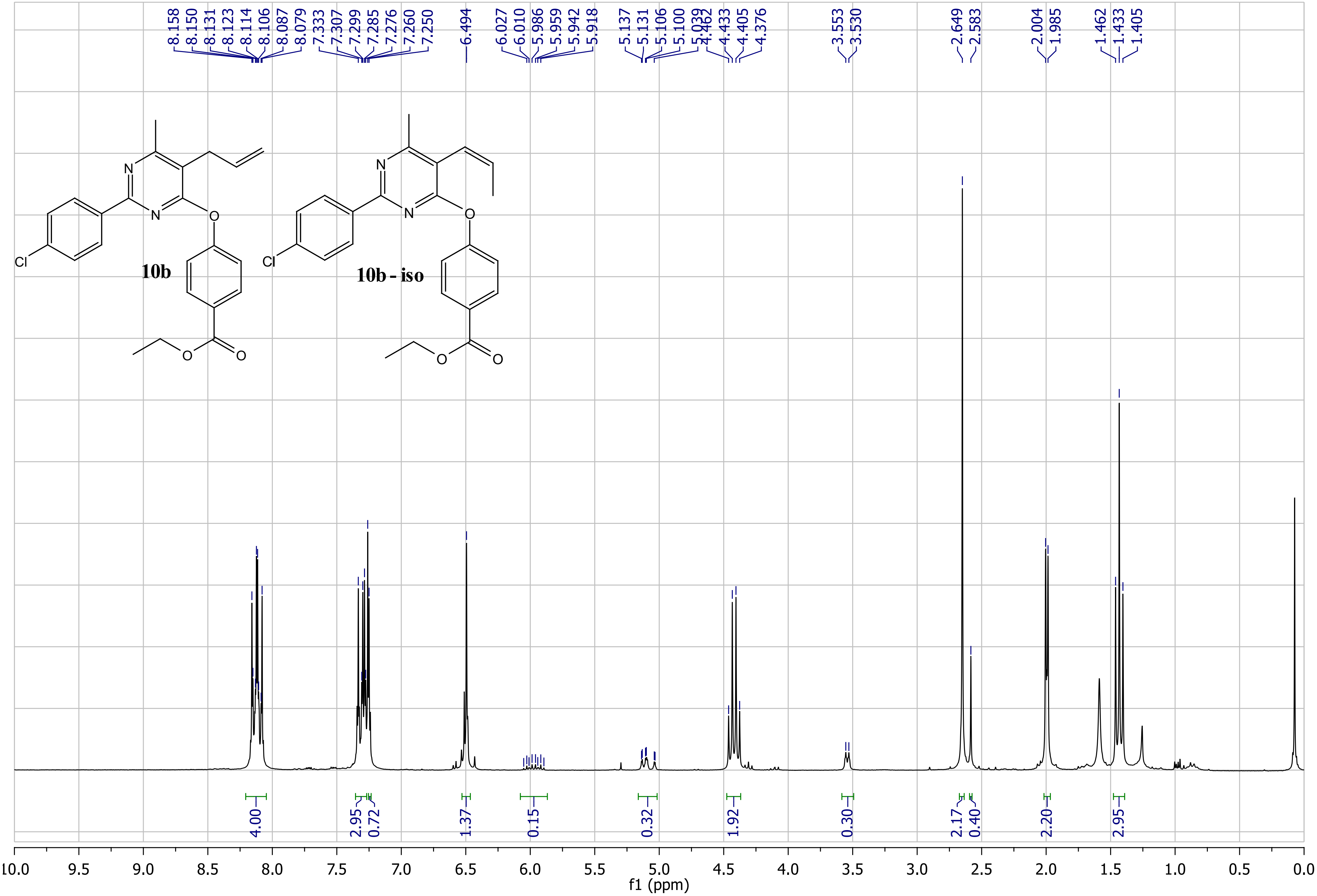
8.158 8.150 8.131 8.123 8.114 8.106 8.087 8.079 7.333 7.307 7.299 7.285 7.276 7.260 7.250 6.494 6.027 6.010 5.986 5.959 5.942 5.918 5.137 5.131 5.106 5.100 5.039 4.462 4.433 4.405 4.376 3.553 3.530 2.649 2.583 2.004 1.985 1.462 1.433 1.405
10b
10b - iso
4.00 2.95 0.72 1.37 0.15 0.32 1.92 0.30 2.17 0.40 2.20 2.95
f1 (ppm)

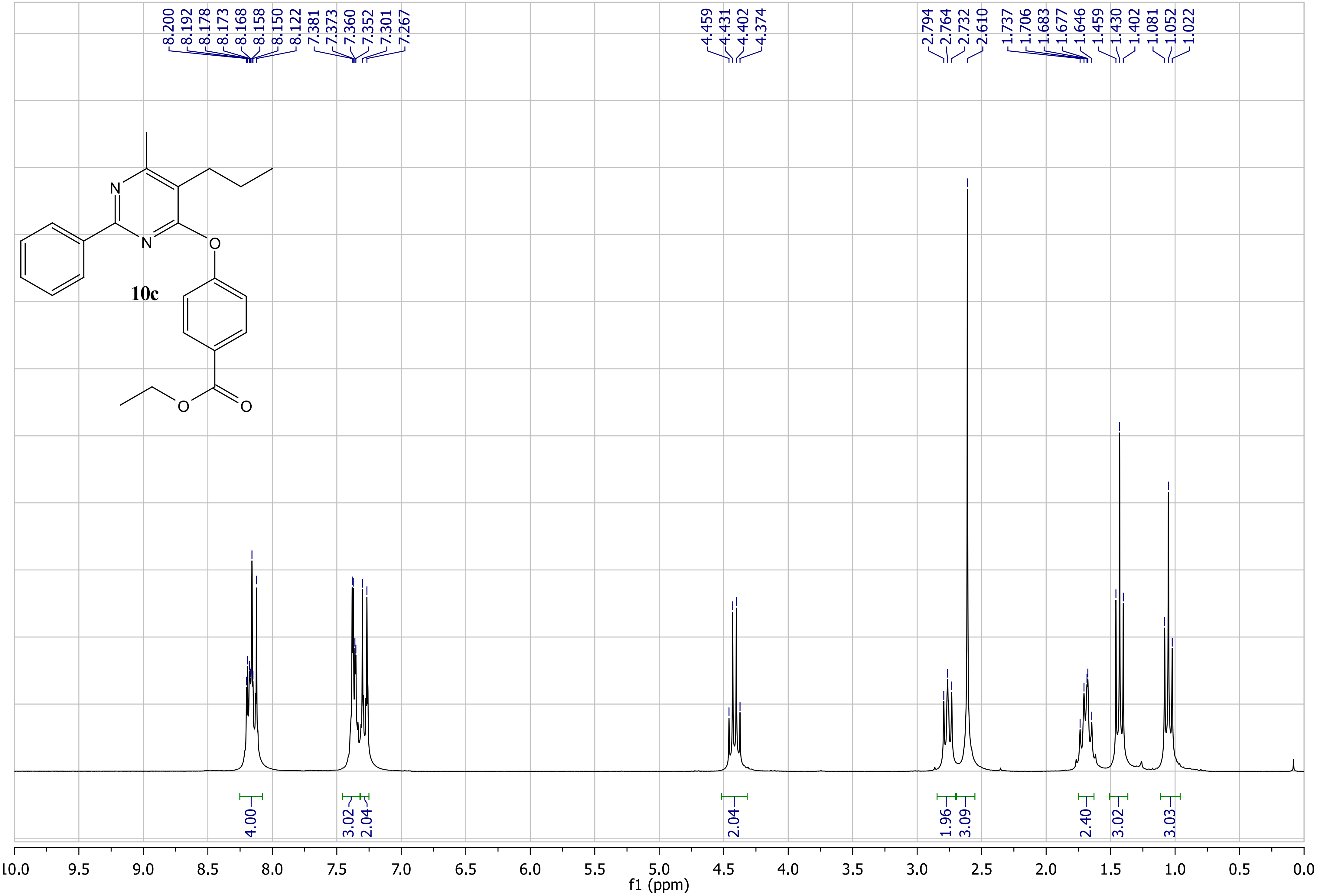
8.200 8.192 8.178 8.173 8.168 8.158 8.150 8.122 7.381 7.373 7.360 7.352 7.301 7.267
4.459 4.431 4.402 4.374
2.794 2.764 2.732 2.610 1.737 1.706 1.683 1.677 1.646 1.459 1.430 1.402 1.081 1.052 1.022
N
N
O
O
O
10c
4.00
3.02
2.04
2.04
1.96
3.09
2.40
3.02
3.03
10.0 9.5 9.0 8.5 8.0 7.5 7.0 6.5 6.0 5.5 5.0 4.5 4.0 3.5 3.0 2.5 2.0 1.5 1.0 0.5 0.0
f1 (ppm)

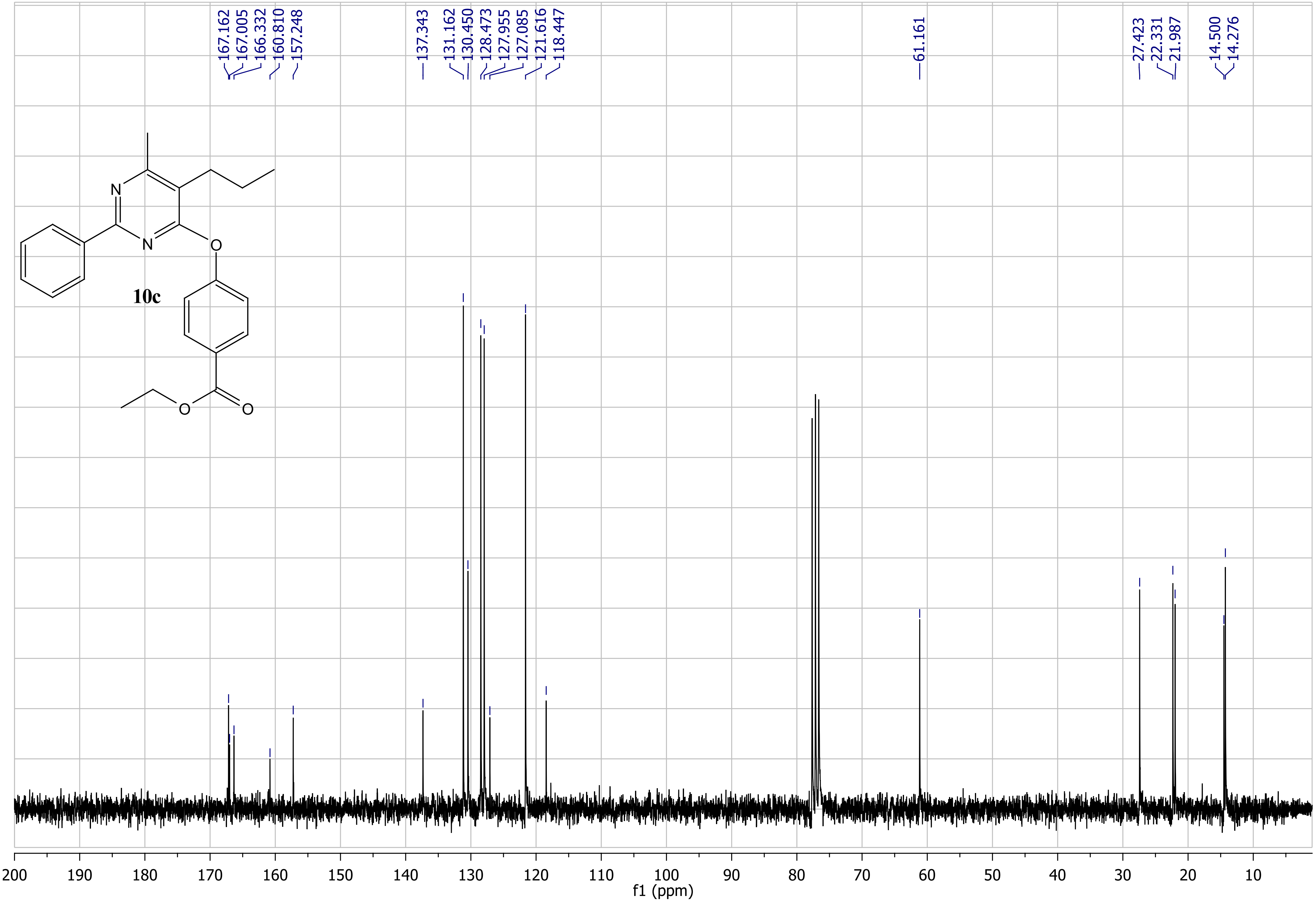

167.162
167.005
166.332
160.810
157.248
137.343
131.162
130.450
128.473
127.955
127.085
121.616
118.447
61.161
27.423
22.331
21.987
14.500
14.276
N
N
O
O
O
10c
200
190
180
170
160
150
140
130
120
110
100
90
80
70
60
50
40
30
20
10
f1 (ppm)

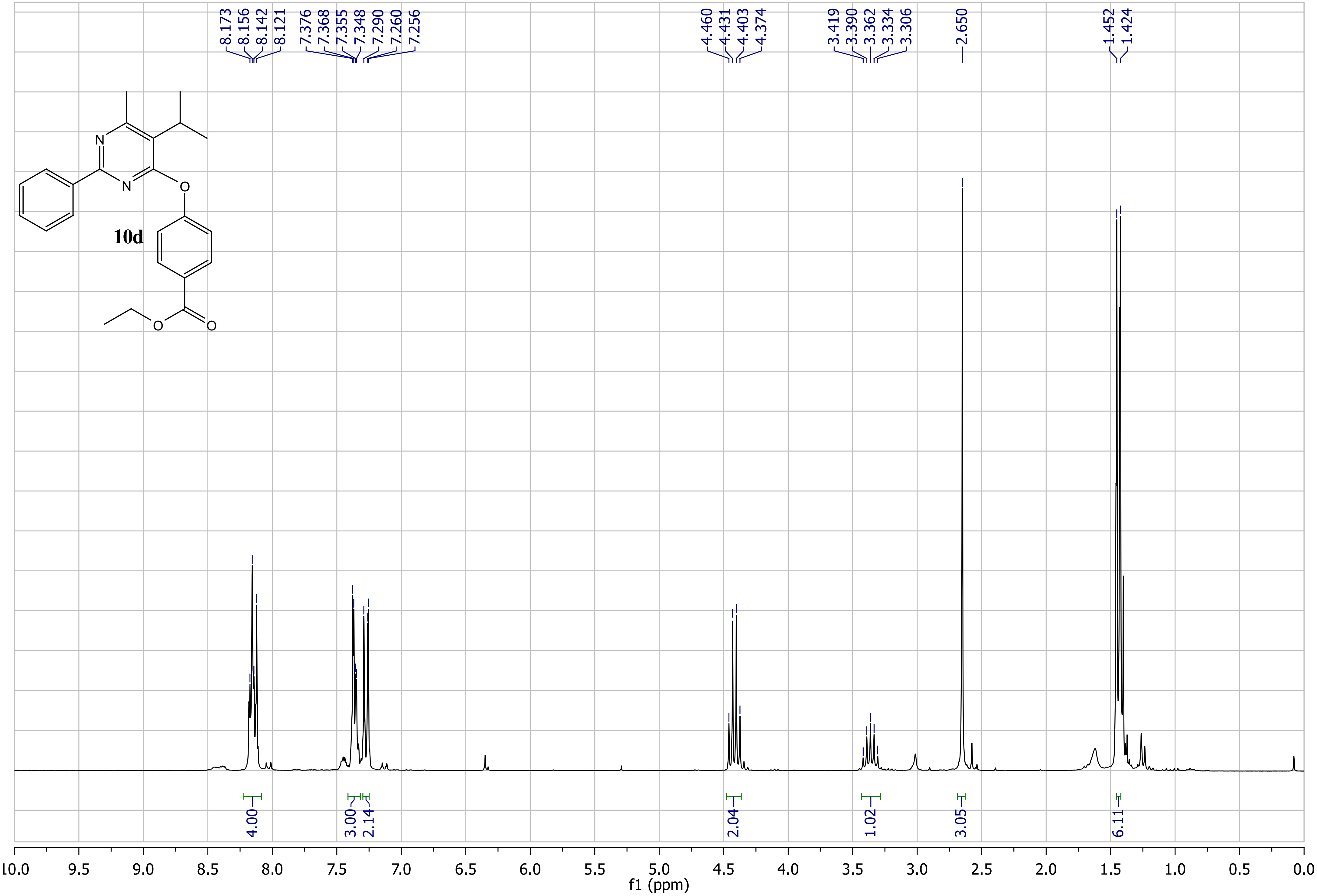
8.173
8.156
8.142
8.121
7.376
7.368
7.355
7.348
7.290
7.260
7.256
4.460
4.431
4.403
4.374
3.419
3.390
3.362
3.334
3.306
2.650
1.452
1.424
N
N
O
10d
O
O
4.00
3.00
2.14
2.04
1.02
3.05
6.11
10.0
9.5
9.0
8.5
8.0
7.5
7.0
6.5
6.0
5.5
5.0
4.5
4.0
3.5
3.0
2.5
2.0
1.5
1.0
0.5
0.0
f1 (ppm)

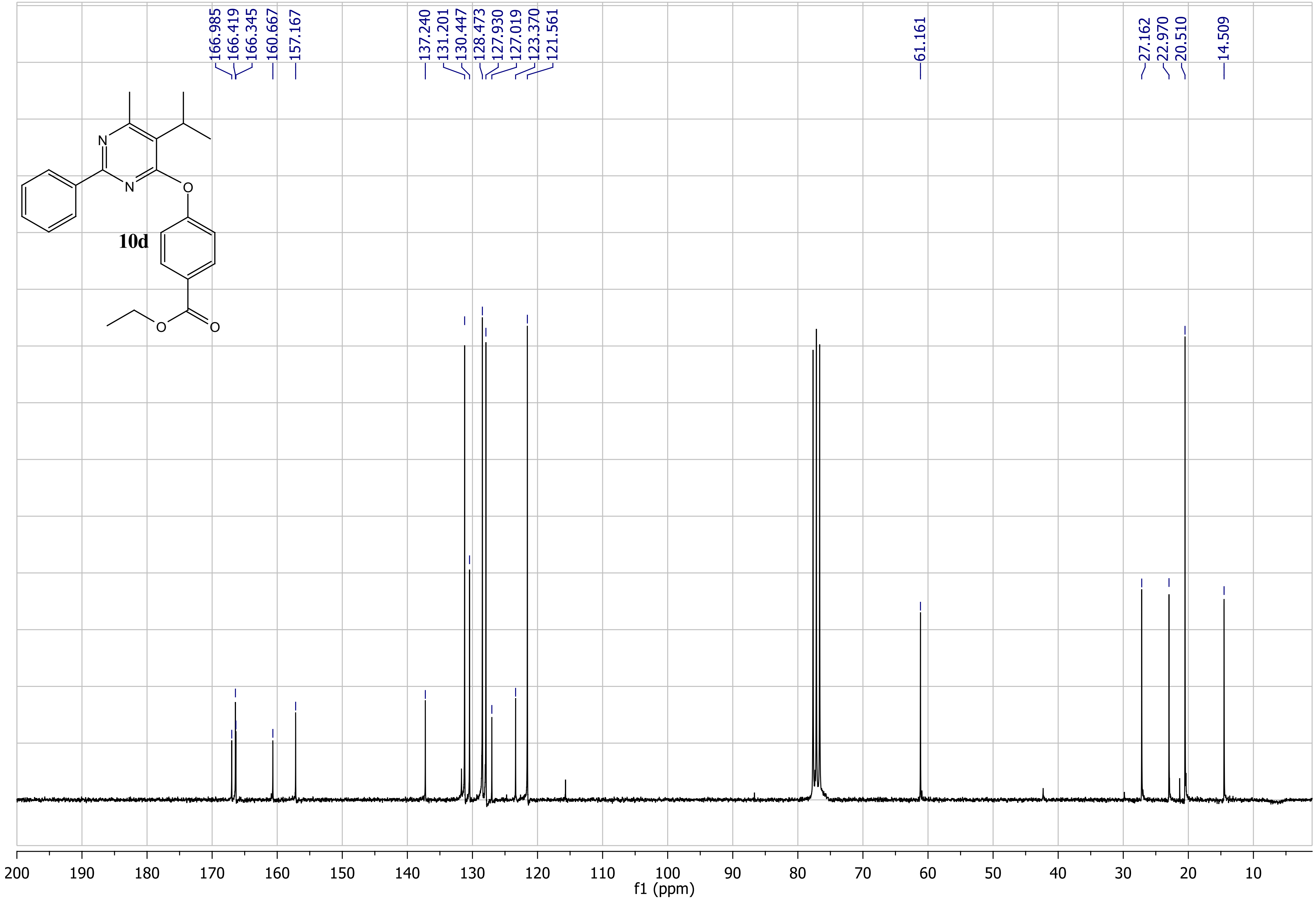
166.985
166.419
166.345
160.667
157.167
137.240
131.201
130.447
128.473
127.930
127.019
123.370
121.561
61.161
27.162
22.970
20.510
14.509
N
N
O
O
O
10d
200
190
180
170
160
150
140
130
120
110
100
90
80
70
60
50
40
30
20
10
f1 (ppm)

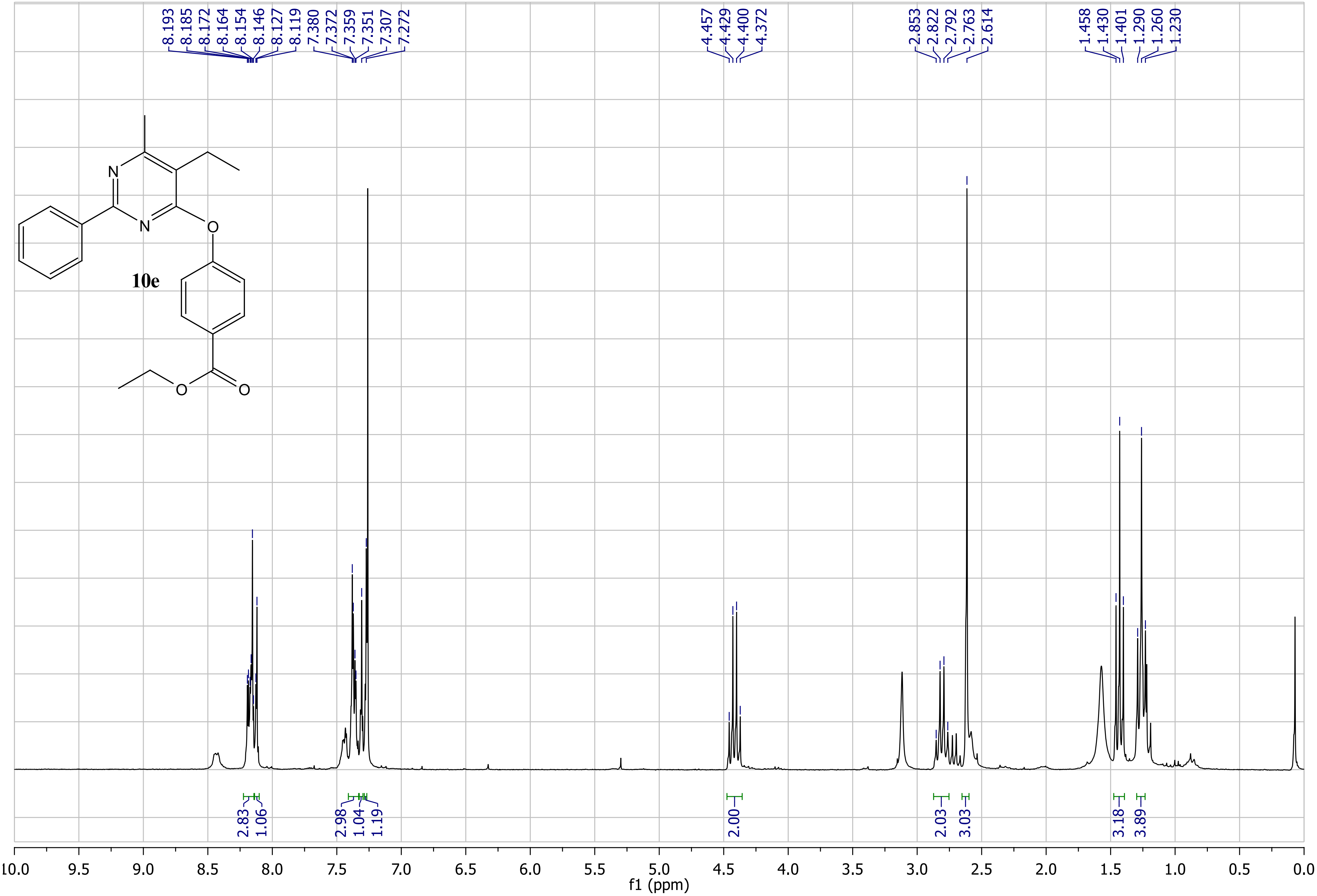

8.193
8.185
8.172
8.164
8.154
8.146
8.127
8.119
7.380
7.372
7.359
7.351
7.307
7.272
4.457
4.429
4.400
4.372
2.853
2.822
2.792
2.763
2.614
1.458
1.430
1.401
1.290
1.260
1.230
N
N
O
O
O
10e
2.83
1.06
2.98
1.04
1.19
2.00
2.03
3.03
3.18
3.89
10.0
9.5
9.0
8.5
8.0
7.5
7.0
6.5
6.0
5.5
5.0
4.5
4.0
3.5
3.0
2.5
2.0
1.5
1.0
0.5
0.0
f1 (ppm)

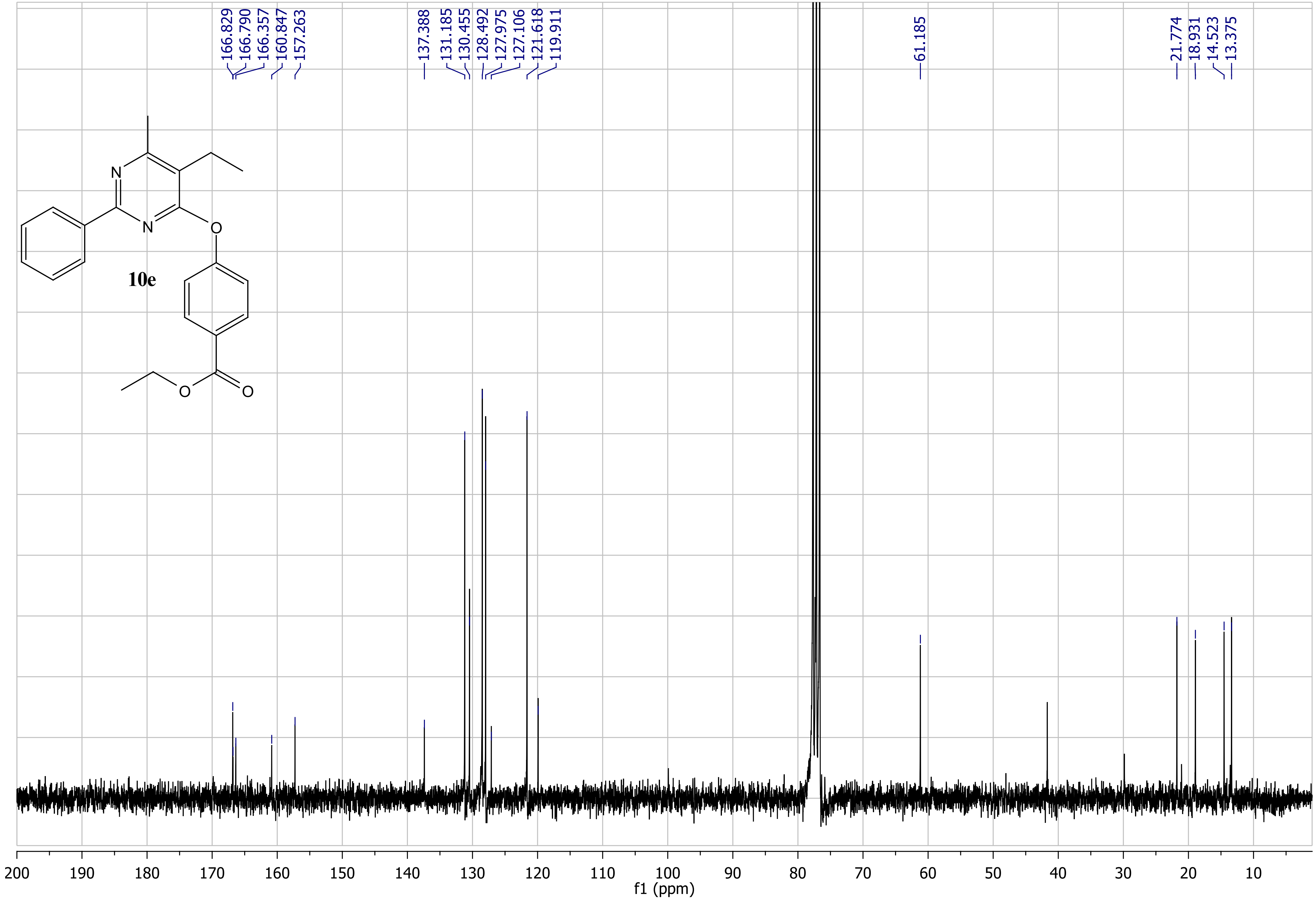
166.829
166.790
166.357
160.847
157.263
137.388
131.185
130.455
128.492
127.975
127.106
121.618
119.911
61.185
21.774
18.931
14.523
13.375
N
N
O
O
O
10e
200
190
180
170
160
150
140
130
120
110
100
90
80
70
60
50
40
30
20
10
f1 (ppm)

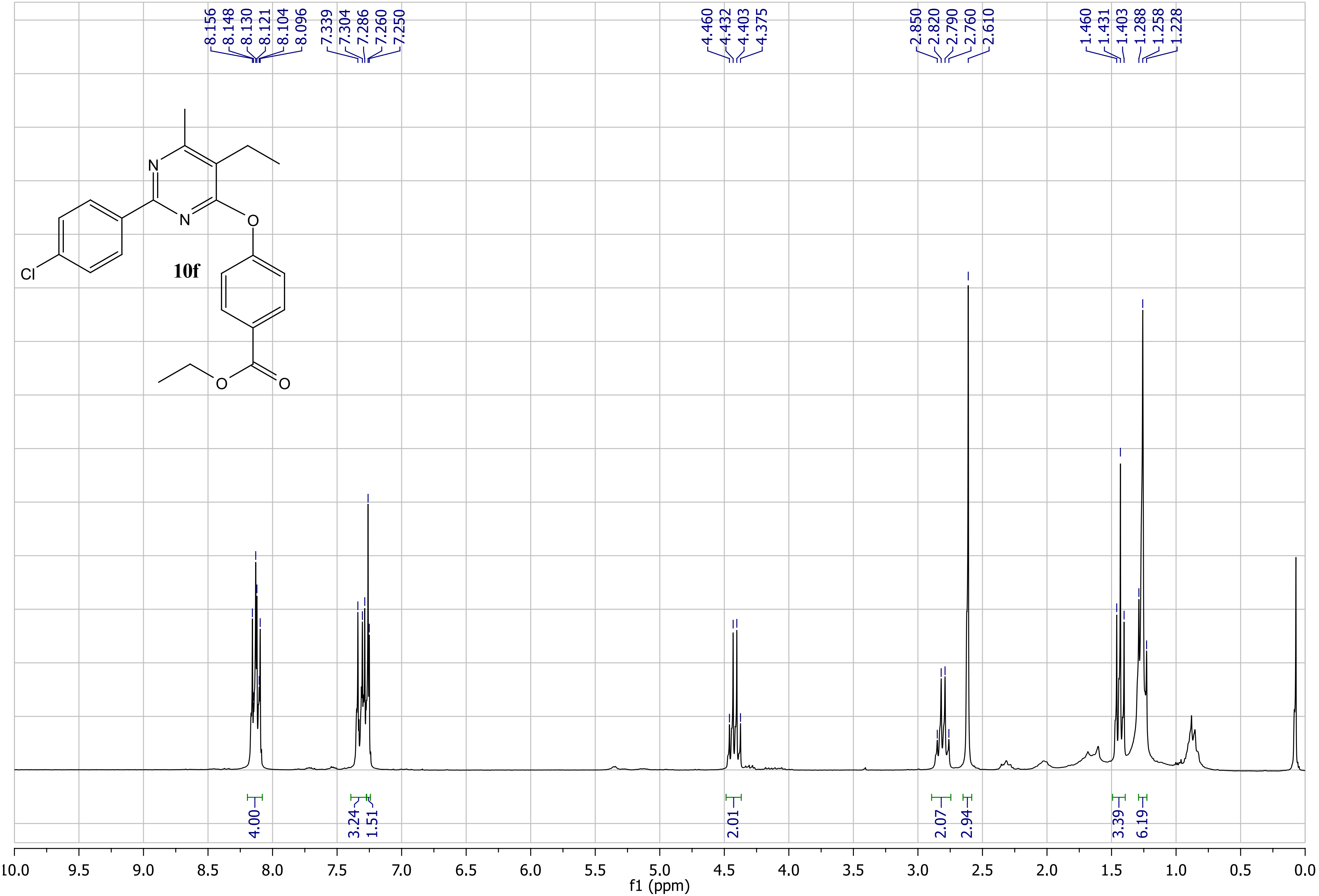
8.156
8.148
8.130
8.121
8.104
8.096
7.339
7.304
7.286
7.260
7.250
4.460
4.432
4.403
4.375
2.850
2.820
2.790
2.760
2.610
1.460
1.431
1.403
1.288
1.258
1.228
N
N
O
O
O
Cl
10f
4.00
3.24
1.51
2.01
2.07
2.94
3.39
6.19
10.0
9.5
9.0
8.5
8.0
7.5
7.0
6.5
6.0
5.5
5.0
4.5
4.0
3.5
3.0
2.5
2.0
1.5
1.0
0.5
0.0
f1 (ppm)

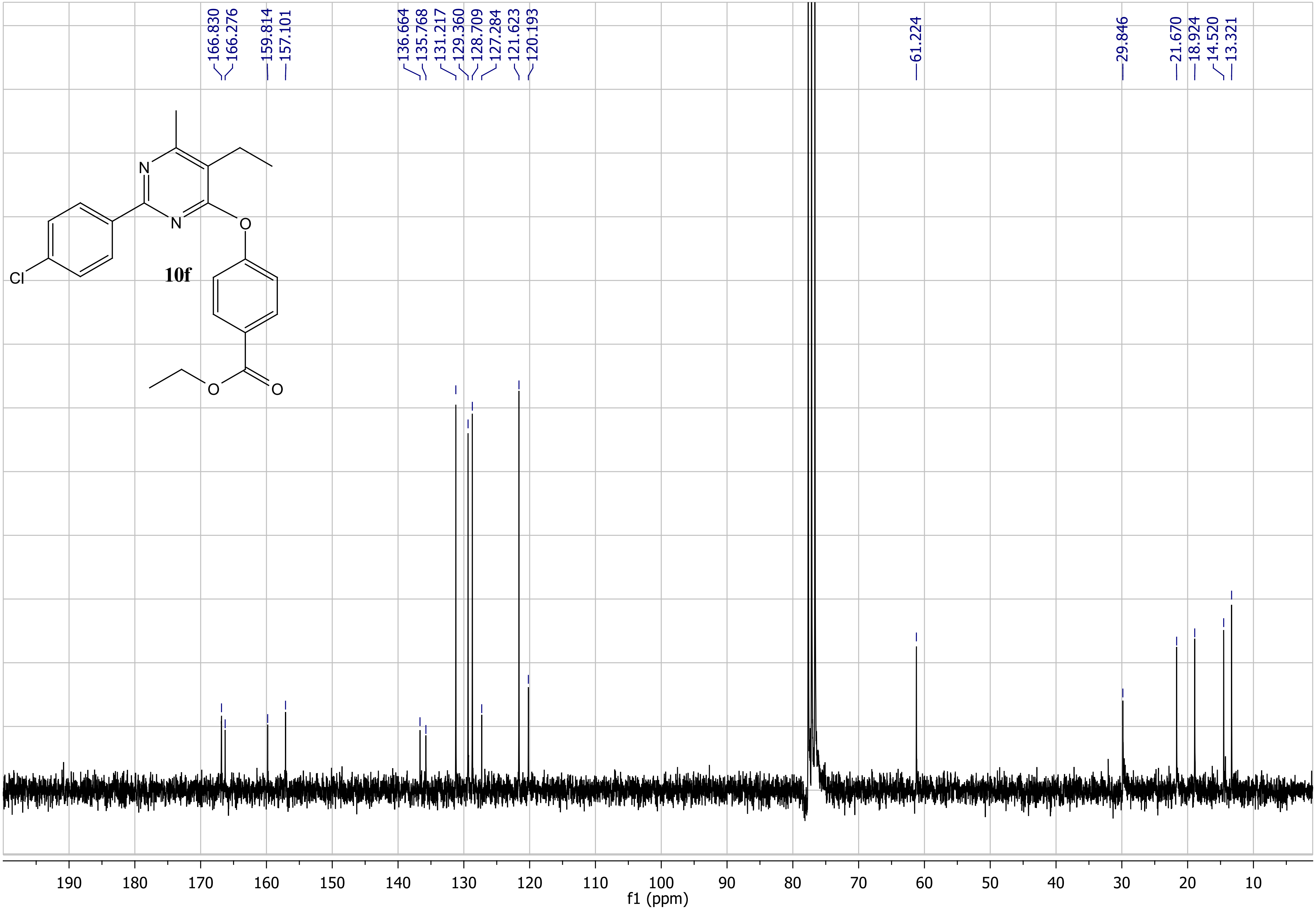
166.830
166.276
159.814
157.101
136.664
135.768
131.217
129.360
128.709
127.284
121.623
120.193
61.224
29.846
21.670
18.924
14.520
13.321
10f
190
180
170
160
150
140
130
120
110
100
90
80
70
60
50
40
30
20
10
f1 (ppm)

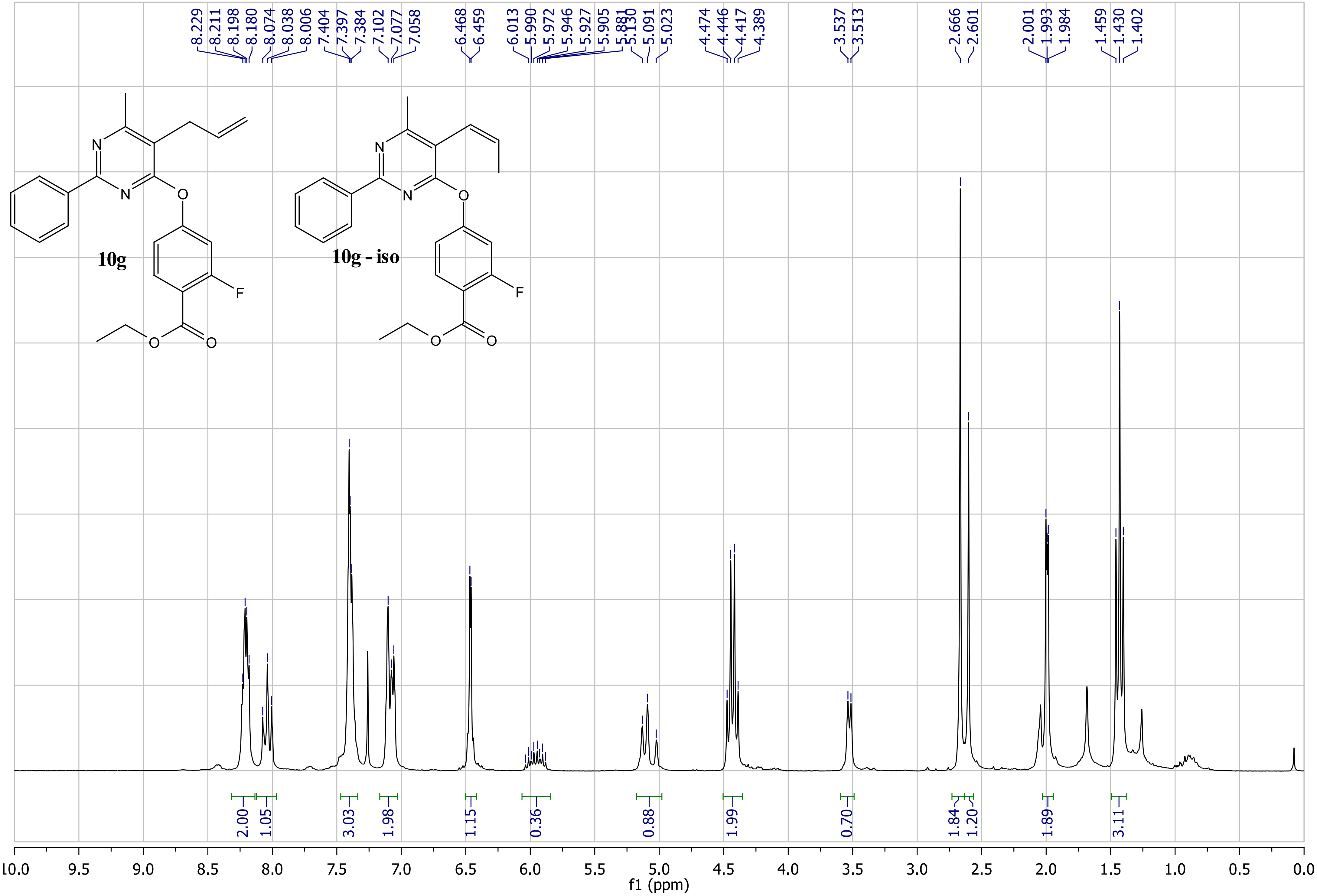
8.229 8.211 8.198 8.180 8.074 8.038 8.006 7.404 7.397 7.384 7.102 7.077 7.058 6.468 6.459 6.013 5.990 5.972 5.946 5.927 5.905 5.881 5.130 5.091 5.023 4.474 4.446 4.417 4.389 3.537 3.513 2.666 2.601 2.001 1.993 1.984 1.459 1.430 1.402
10g
10g - iso
N
N
O
F
O
O
2.00 1.05 3.03 1.98 1.15 0.36 0.88 1.99 0.70 1.84 1.20 1.89 3.11
10.0 9.5 9.0 8.5 8.0 7.5 7.0 6.5 6.0 5.5 5.0 4.5 4.0 3.5 3.0 2.5 2.0 1.5 1.0 0.5 0.0
f1 (ppm)

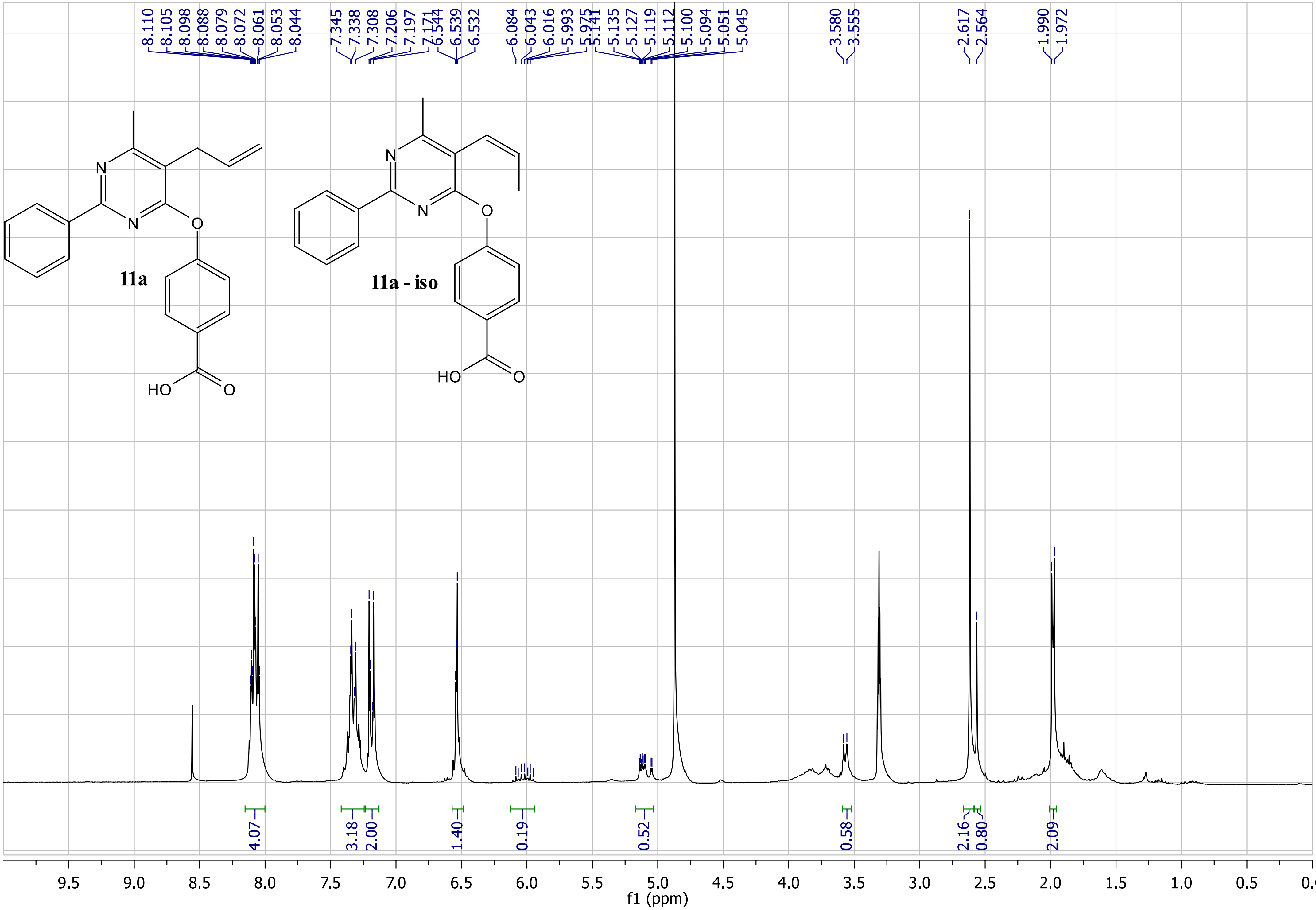
8.110
8.105
8.098
8.088
8.079
8.072
8.061
8.053
8.044
7.345
7.338
7.308
7.206
7.197
7.171
6.544
6.539
6.532
6.084
6.043
6.016
5.993
5.975
5.141
5.135
5.127
5.119
5.112
5.100
5.094
5.051
5.045
3.580
3.555
2.617
2.564
1.990
1.972
N
N
O
HO
O
11a
N
N
O
HO
O
11a - iso
4.07
3.18
2.00
1.40
0.19
0.52
0.58
2.16
0.80
2.09
9.5
9.0
8.5
8.0
7.5
7.0
6.5
6.0
5.5
5.0
4.5
4.0
3.5
3.0
2.5
2.0
1.5
1.0
0.5
0.0
f1 (ppm)

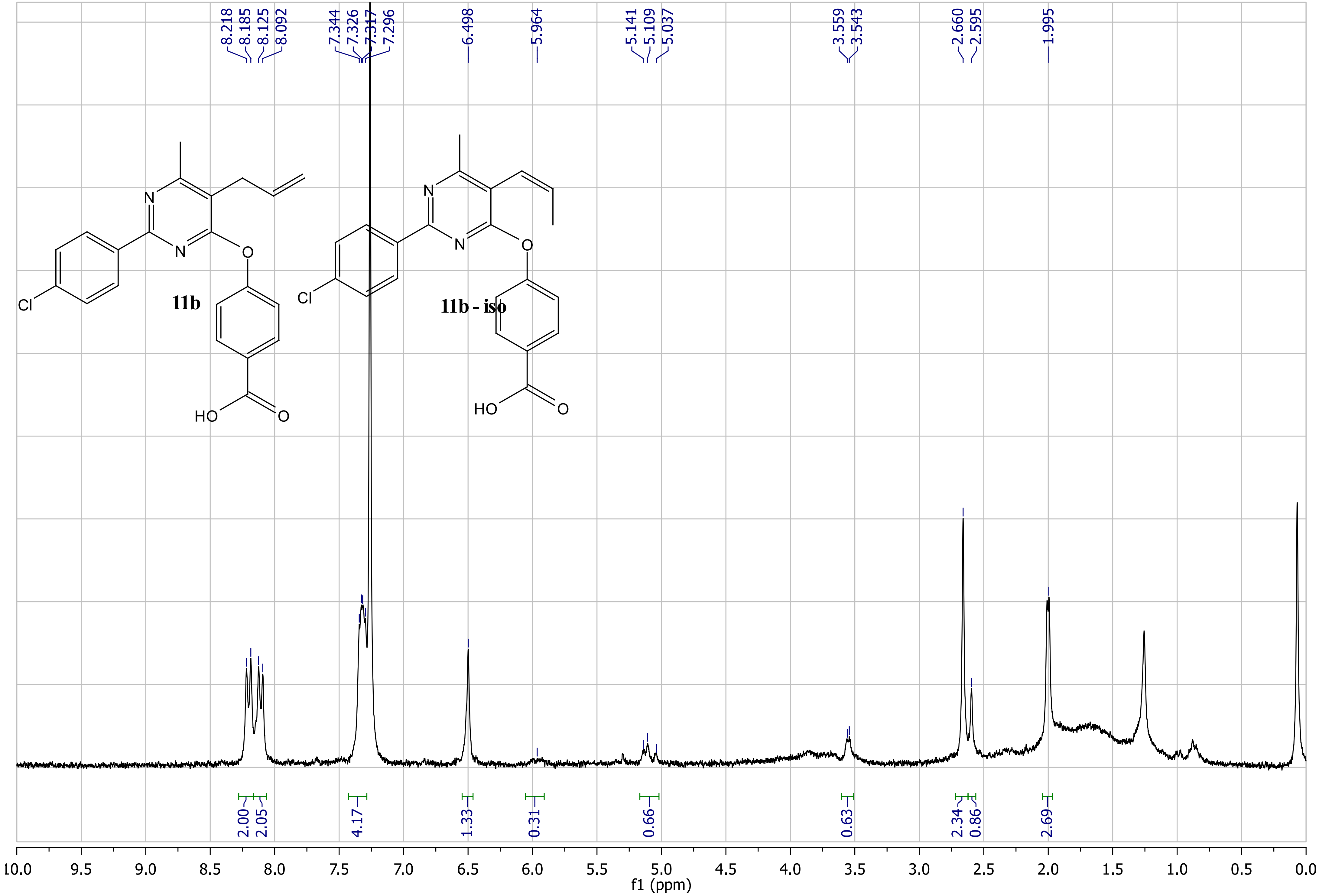
8.218
8.185
8.125
8.092
7.344
7.326
7.317
7.296
6.498
5.964
5.141
5.109
5.037
3.559
3.543
2.660
2.595
1.995
11b
11b - iso
Cl
N
O
HO
2.00
2.05
4.17
1.33
0.31
0.66
0.63
2.34
0.86
2.69
10.0 9.5 9.0 8.5 8.0 7.5 7.0 6.5 6.0 5.5 5.0 4.5 4.0 3.5 3.0 2.5 2.0 1.5 1.0 0.5 0.0
f1 (ppm)

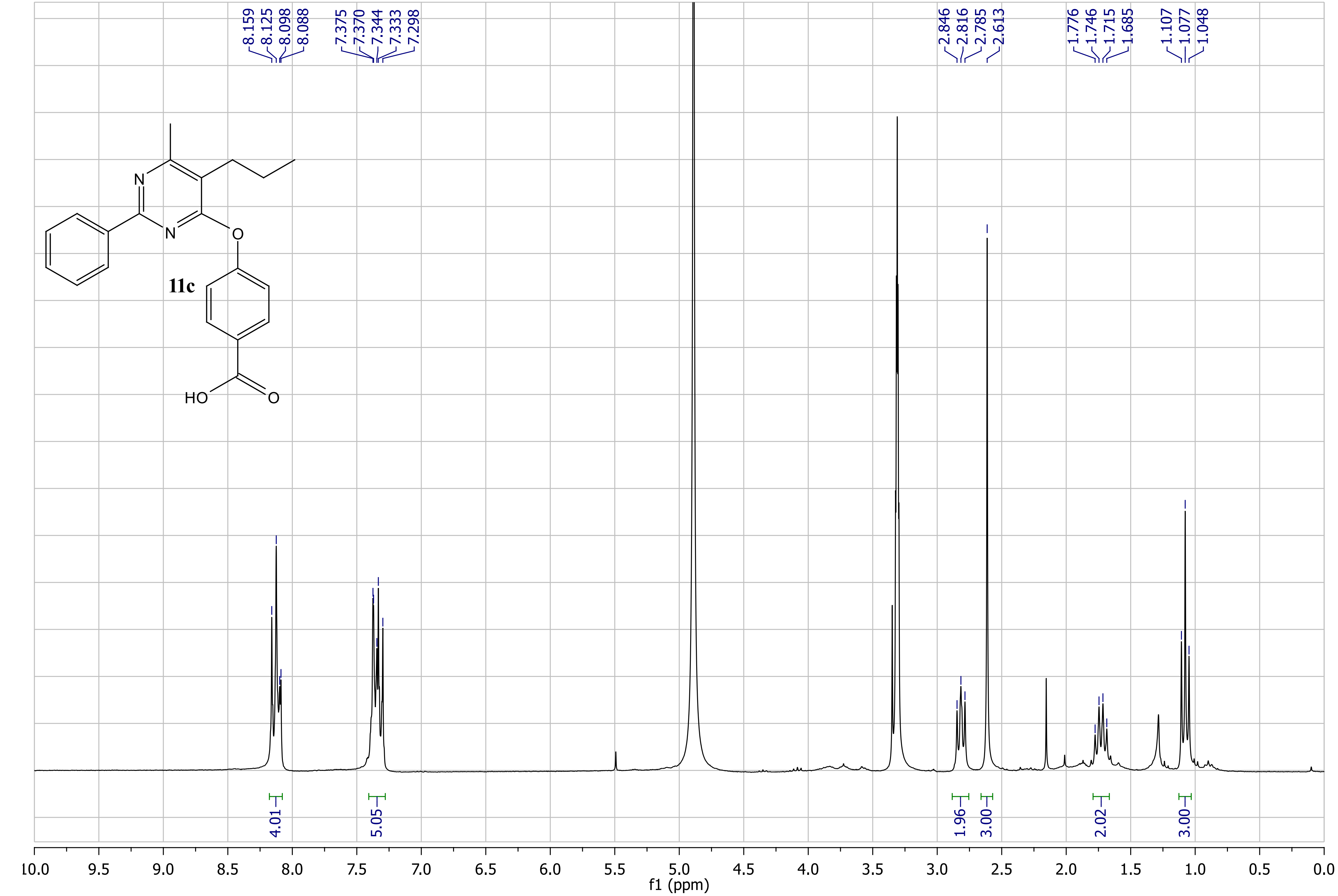
8.159
8.125
8.098
8.088
7.375
7.370
7.344
7.333
7.298
2.846
2.816
2.785
2.613
1.776
1.746
1.715
1.685
1.107
1.077
1.048
N
N
O
HO
O
11c
4.01
5.05
1.96
3.00
2.02
3.00
10.0
9.5
9.0
8.5
8.0
7.5
7.0
6.5
6.0
5.5
5.0
4.5
4.0
3.5
3.0
2.5
2.0
1.5
1.0
0.5
0.0
f1 (ppm)

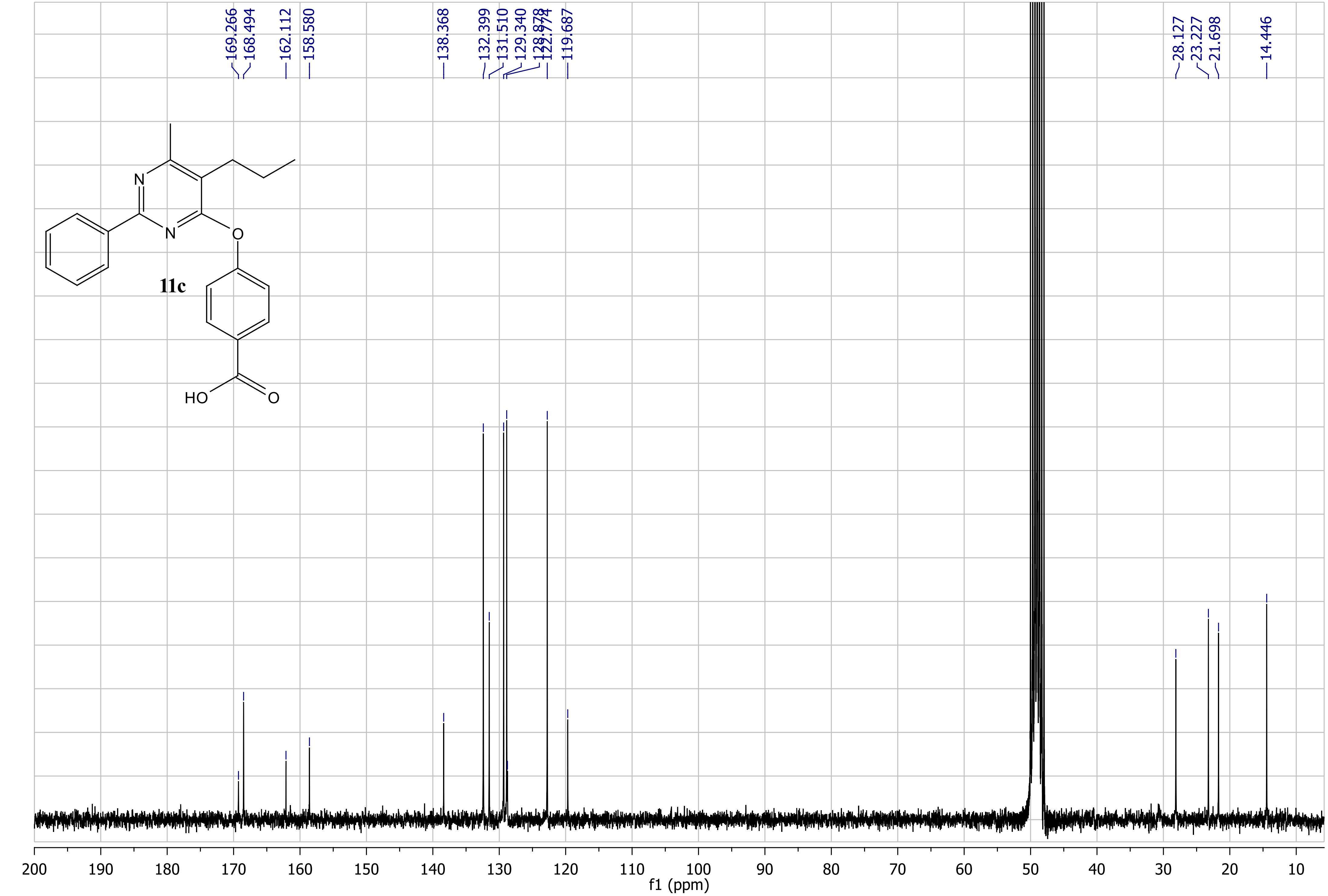

169.266
168.494
162.112
158.580
138.368
132.399
131.510
129.340
122.774
119.687
28.127
23.227
21.698
14.446
N
N
O
11c
HO
O
200
190
180
170
160
150
140
130
120
110
100
90
80
70
60
50
40
30
20
10
f1 (ppm)

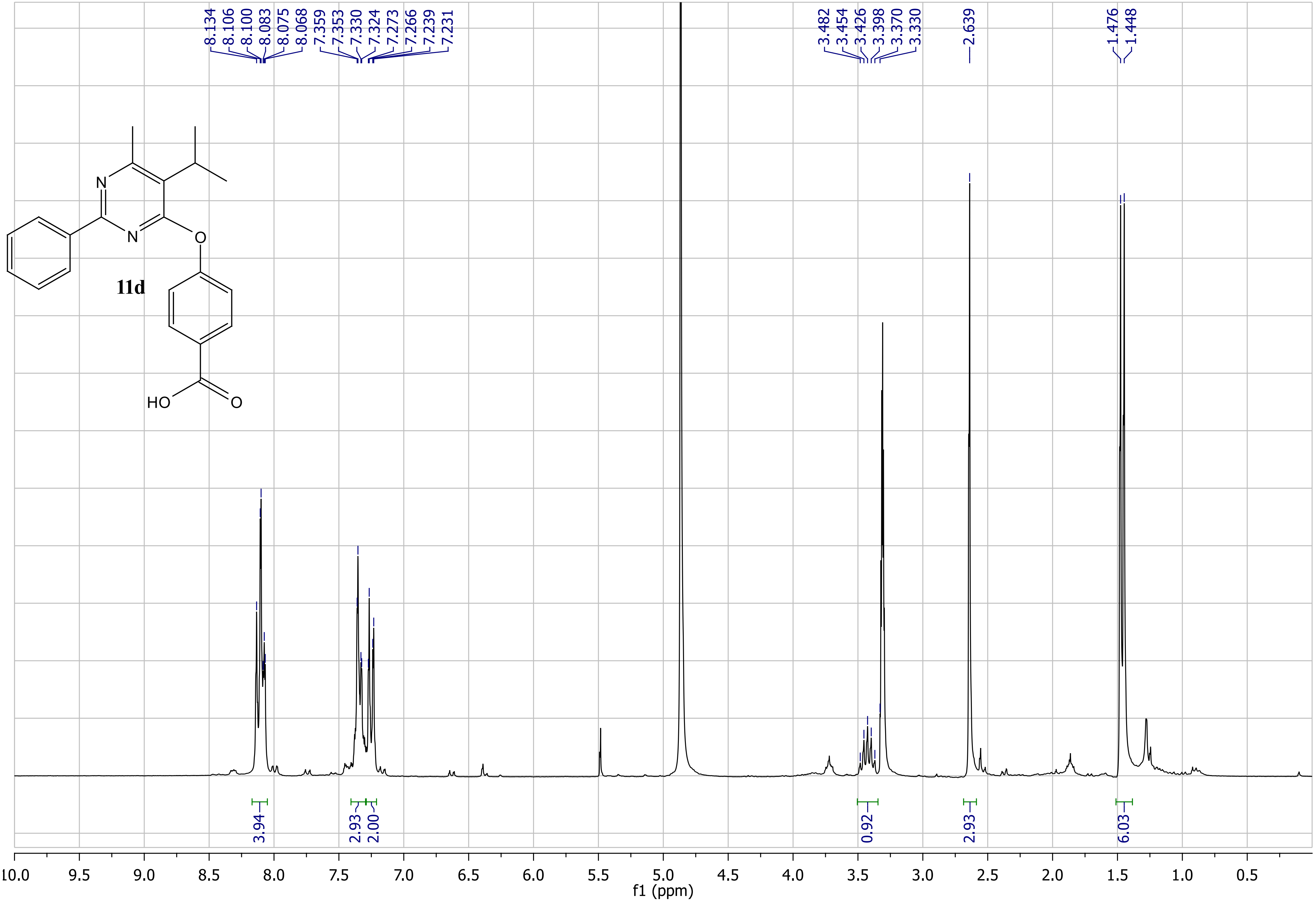
8.134
8.106
8.100
8.083
8.075
8.068
7.359
7.353
7.330
7.324
7.273
7.266
7.239
7.231
3.482
3.454
3.426
3.398
3.370
3.330
2.639
1.476
1.448
N
N
O
HO
O
11d
3.94
2.93
2.00
0.92
2.93
6.03
10.0 9.5 9.0 8.5 8.0 7.5 7.0 6.5 6.0 5.5 5.0 4.5 4.0 3.5 3.0 2.5 2.0 1.5 1.0 0.5
f1 (ppm)

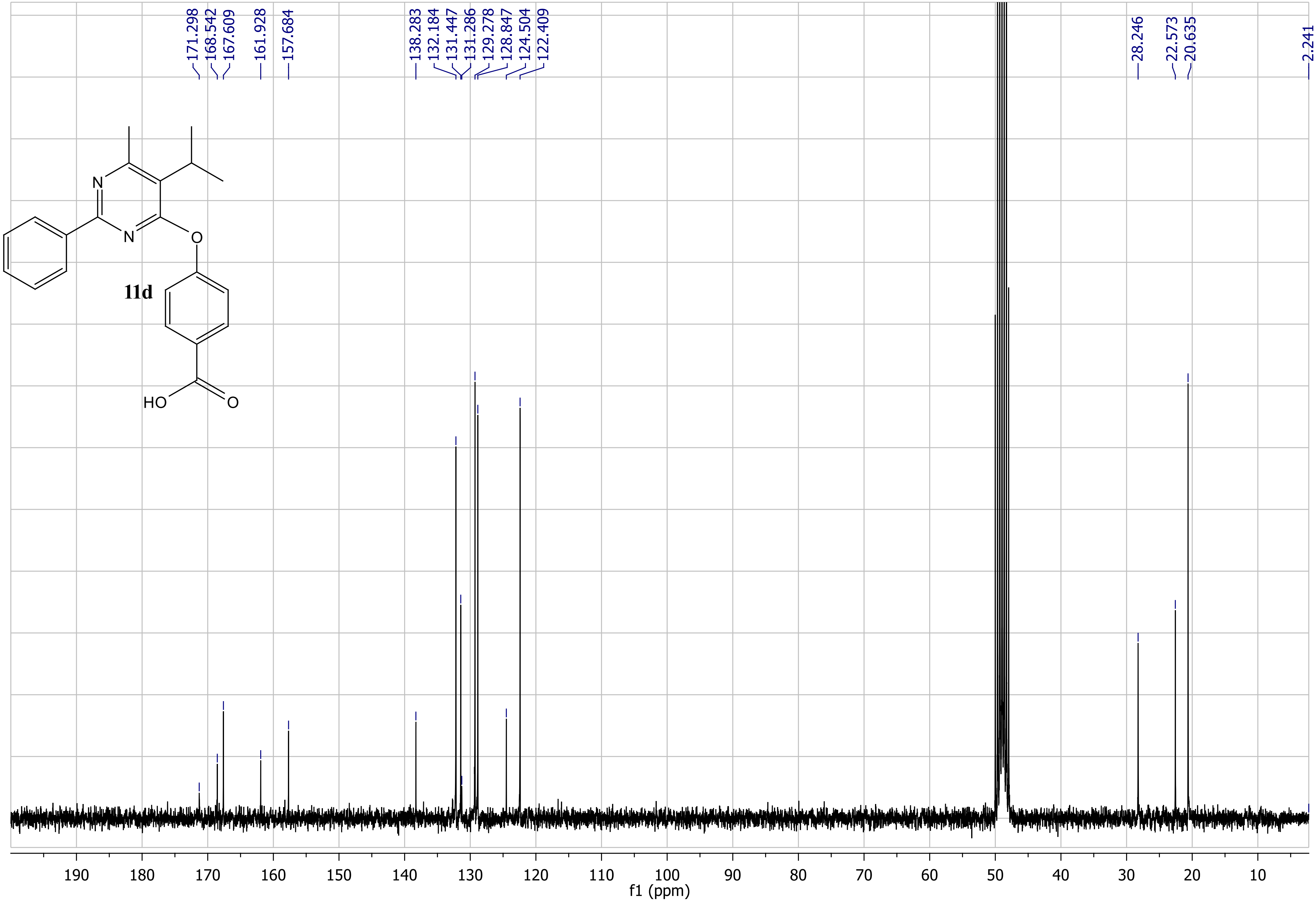
171.298
168.542
167.609
161.928
157.684
138.283
132.184
131.447
131.286
129.278
128.847
124.504
122.409
28.246
22.573
20.635
2.241
11d
N
N
O
HO
O
190 180 170 160 150 140 130 120 110 100 90 80 70 60 50 40 30 20 10
f1 (ppm)

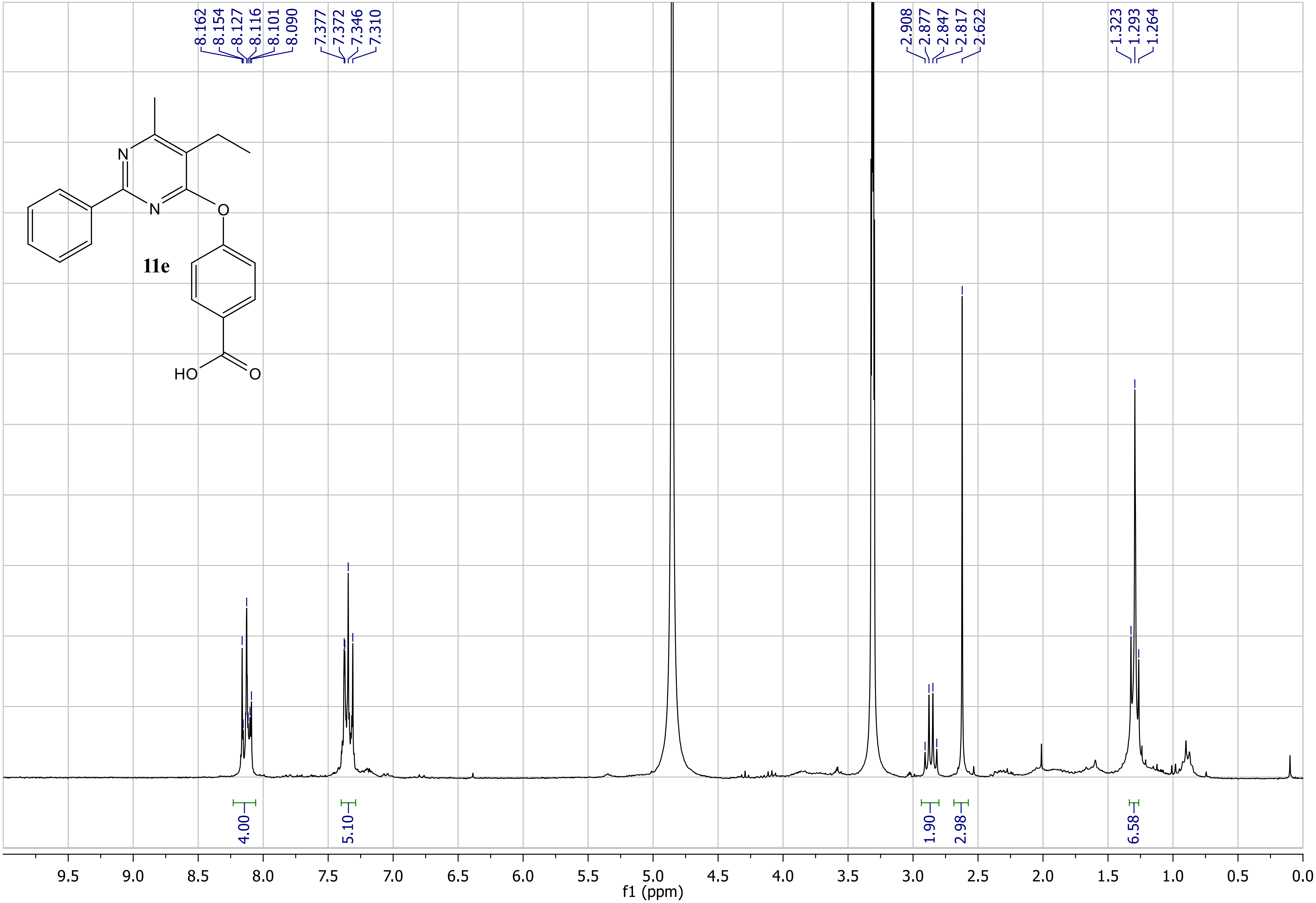

8.162
8.154
8.127
8.116
8.101
8.090
7.377
7.372
7.346
7.310
2.908
2.877
2.847
2.817
2.622
1.323
1.293
1.264
N
N
O
HO
O
11e
4.00
5.10
1.90
2.98
6.58
9.5
9.0
8.5
8.0
7.5
7.0
6.5
6.0
5.5
5.0
4.5
4.0
3.5
3.0
2.5
2.0
1.5
1.0
0.5
0.0
f1 (ppm)

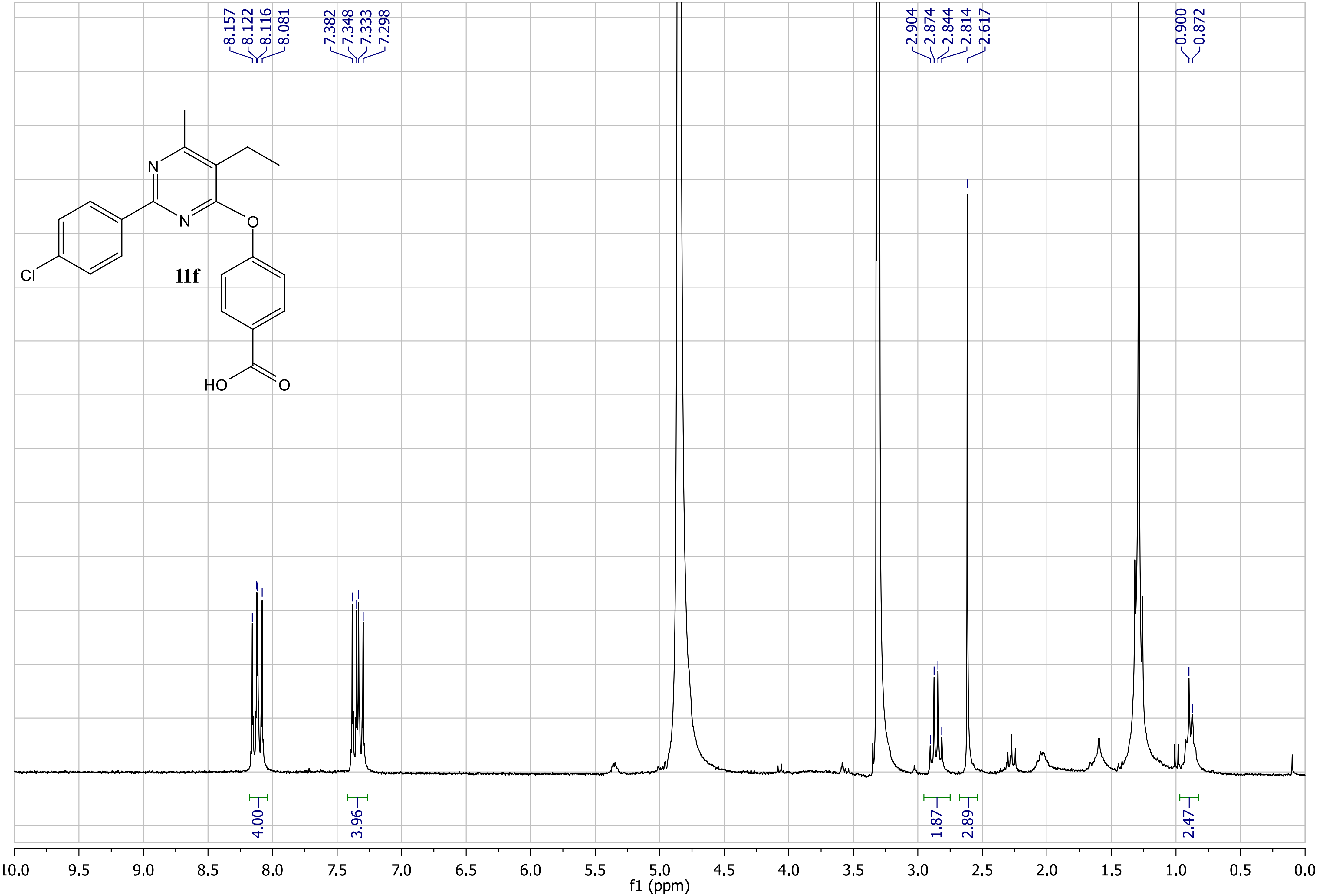

8.157
8.122
8.116
8.081
7.382
7.348
7.333
7.298
2.904
2.874
2.844
2.814
2.617
0.900
0.872
N
N
O
Cl
11f
HO
O
4.00
3.96
1.87
2.89
2.47
10.0
9.5
9.0
8.5
8.0
7.5
7.0
6.5
6.0
5.5
5.0
4.5
4.0
3.5
3.0
2.5
2.0
1.5
1.0
0.5
0.0
f1 (ppm)

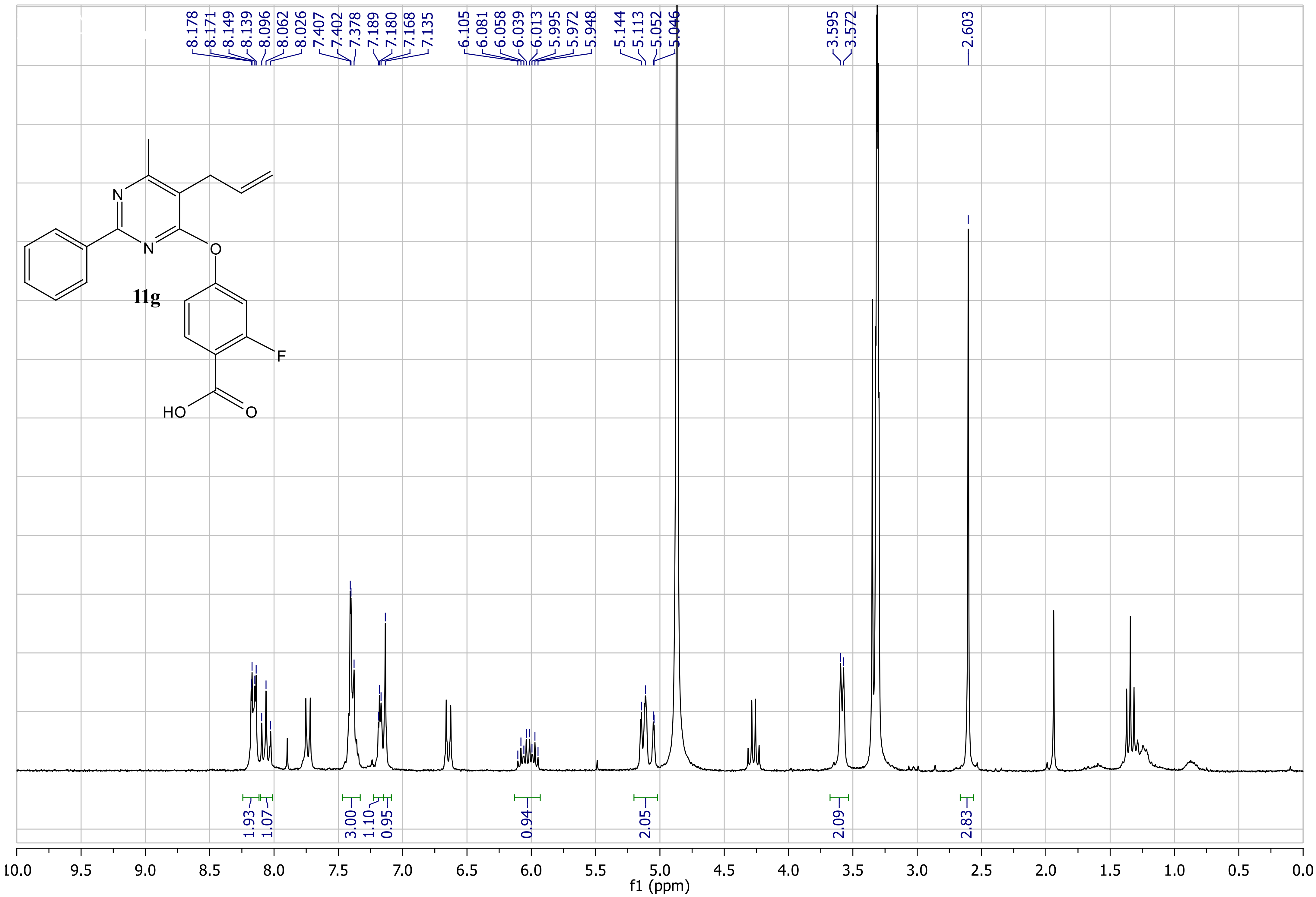
8.178
8.171
8.149
8.139
8.096
8.062
8.026
7.407
7.402
7.378
7.189
7.180
7.168
7.135
6.105
6.081
6.058
6.039
6.013
5.995
5.972
5.948
5.144
5.113
5.052
5.046
3.595
3.572
2.603
N
N
O
F
HO
O
11g
1.93
1.07
3.00
1.10
0.95
0.94
2.05
2.09
2.83
f1 (ppm)

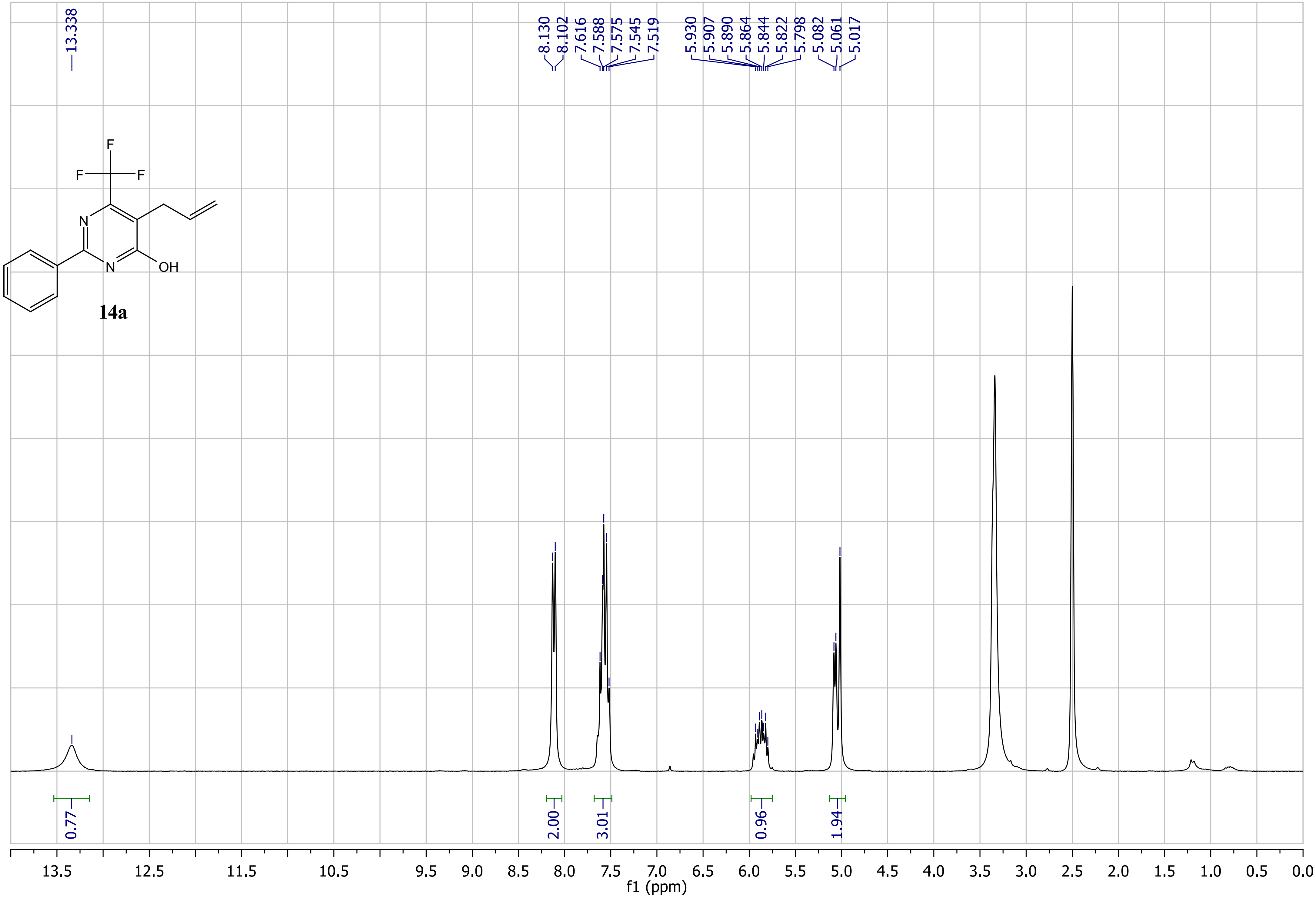
13.338
8.130
8.102
7.616
7.588
7.575
7.545
7.519
5.930
5.907
5.890
5.864
5.844
5.822
5.798
5.082
5.061
5.017
F
F
F
N
N
OH
14a
0.77
2.00
3.01
0.96
1.94
13.5
12.5
11.5
10.5
9.5
9.0
8.5
8.0
7.5
7.0
6.5
6.0
5.5
5.0
4.5
4.0
3.5
3.0
2.5
2.0
1.5
1.0
0.5
0.0
f1 (ppm)

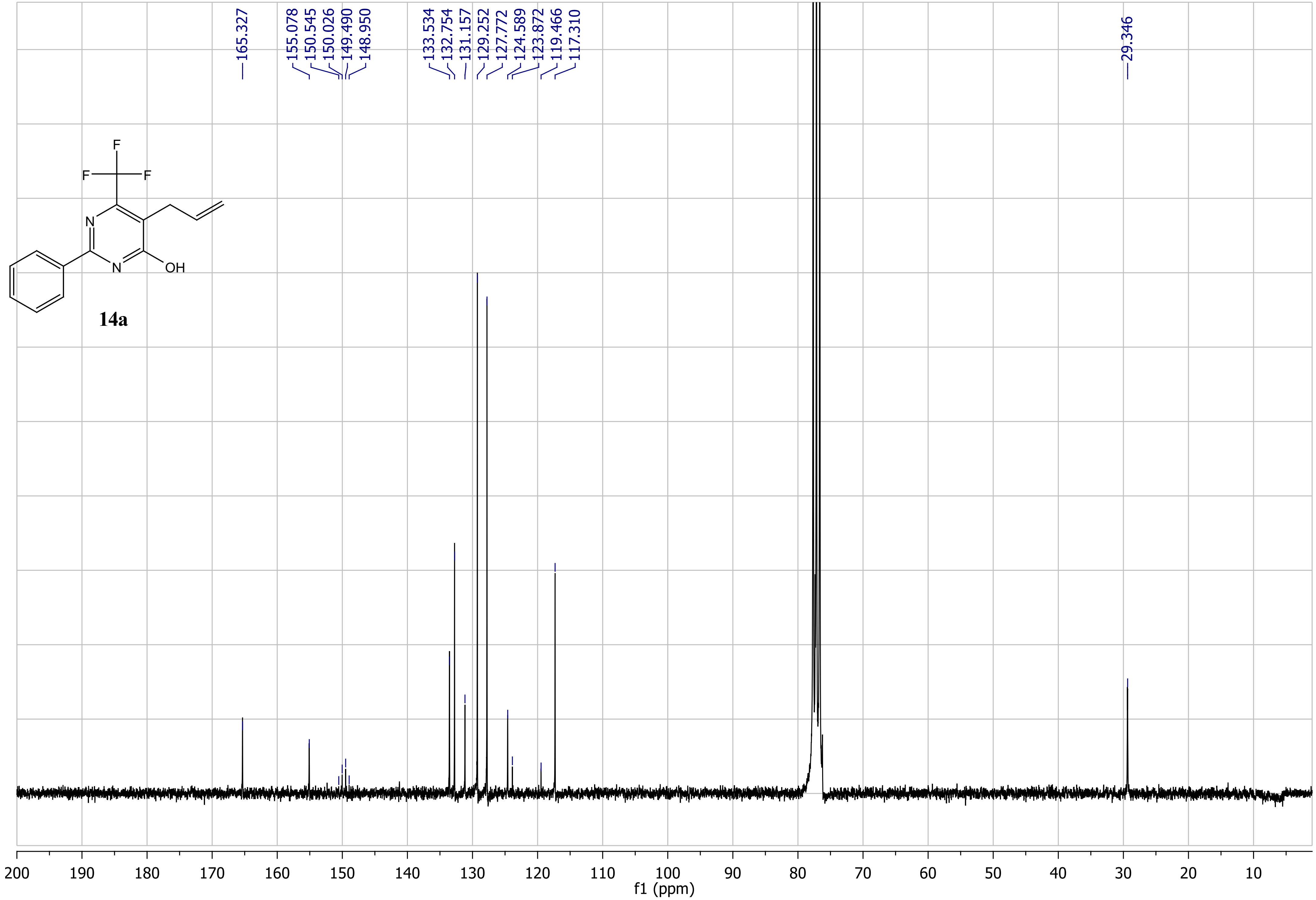

165.327
155.078
150.545
150.026
149.490
148.950
133.534
132.754
131.157
129.252
127.772
124.589
123.872
119.466
117.310
29.346
F
F
F
N
N
OH
14a
200 190 180 170 160 150 140 130 120 110 100 90 80 70 60 50 40 30 20 10
f1 (ppm)

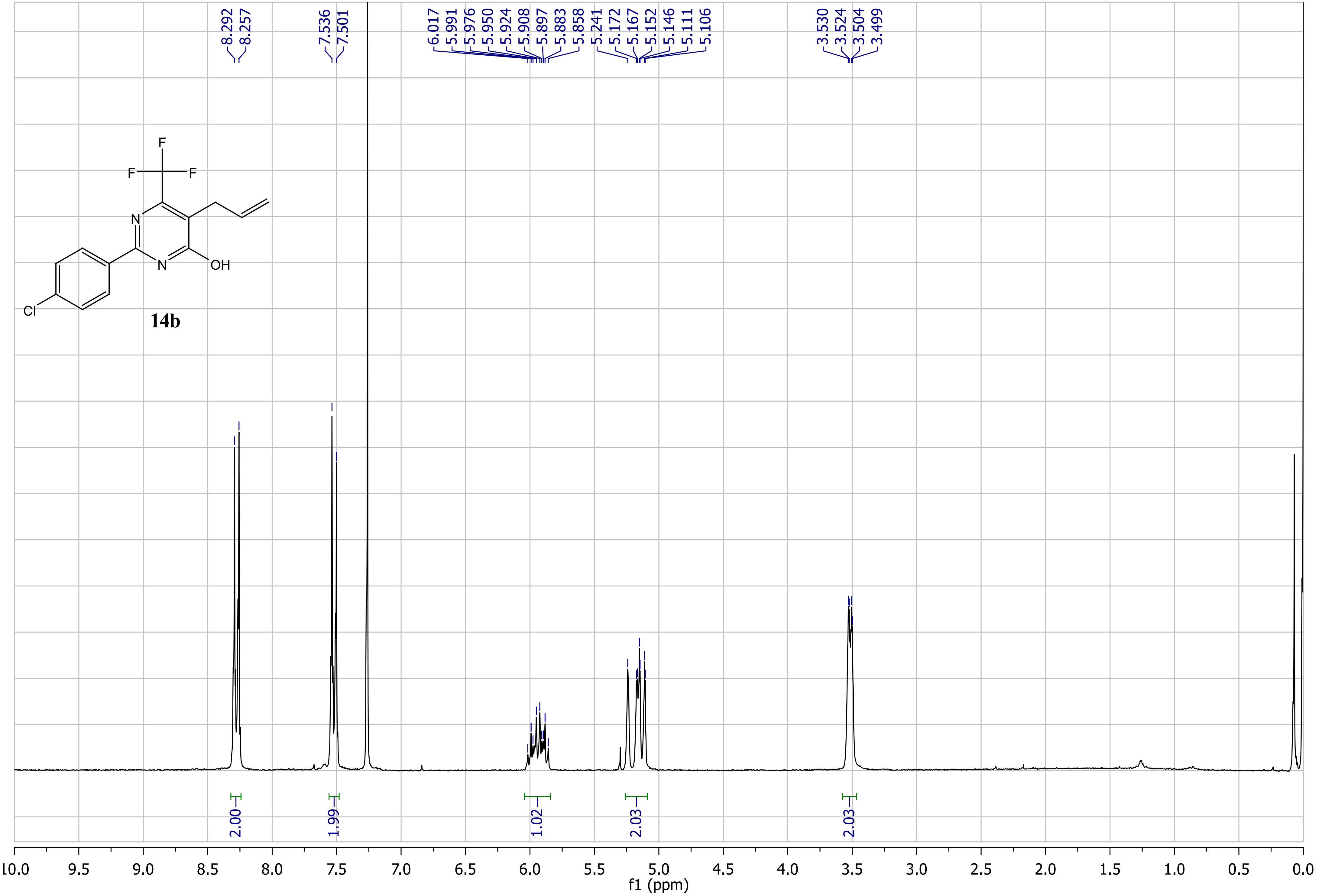
8.292
8.257
7.536
7.501
6.017
5.991
5.976
5.950
5.924
5.908
5.897
5.883
5.858
5.241
5.172
5.167
5.152
5.146
5.111
5.106
3.530
3.524
3.504
3.499
F
F
F
N
N
OH
Cl
14b
2.00
1.99
1.02
2.03
2.03
10.0
9.5
9.0
8.5
8.0
7.5
7.0
6.5
6.0
5.5
5.0
4.5
4.0
3.5
3.0
2.5
2.0
1.5
1.0
0.5
0.0
f1 (ppm)

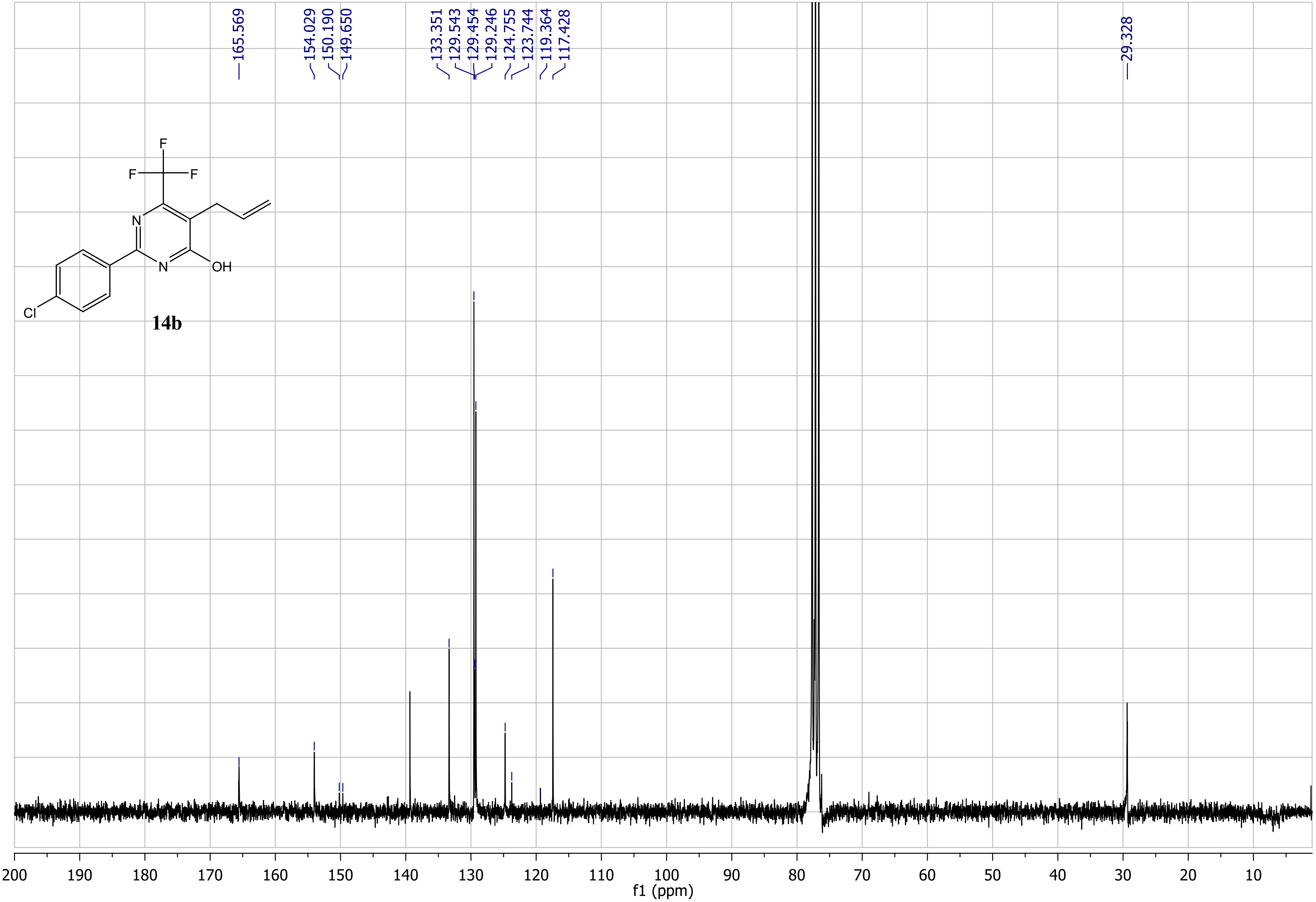

165.569
154.029
150.190
149.650
133.351
129.543
129.454
129.246
124.755
123.744
119.364
117.428
29.328
F
F
F
N
N
OH
Cl
14b
f1 (ppm)
200 190 180 170 160 150 140 130 120 110 100 90 80 70 60 50 40 30 20 10

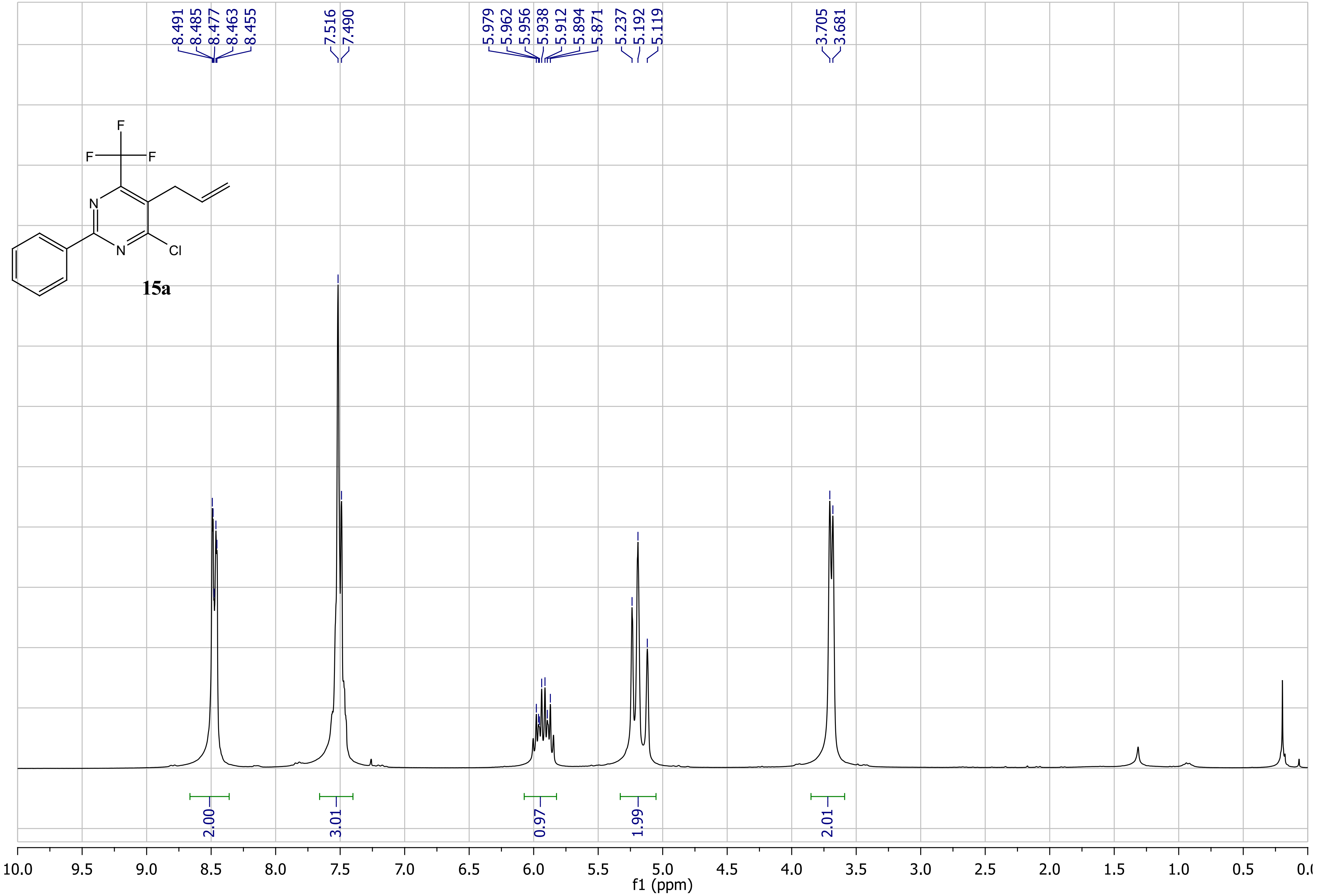
8.491
8.485
8.477
8.463
8.455
7.516
7.490
5.979
5.962
5.956
5.938
5.912
5.894
5.871
5.237
5.192
5.119
3.705
3.681
F
F
F
N
N
Cl
15a
2.00
3.01
0.97
1.99
2.01
10.0
9.5
9.0
8.5
8.0
7.5
7.0
6.5
6.0
5.5
5.0
4.5
4.0
3.5
3.0
2.5
2.0
1.5
1.0
0.5
f1 (ppm)

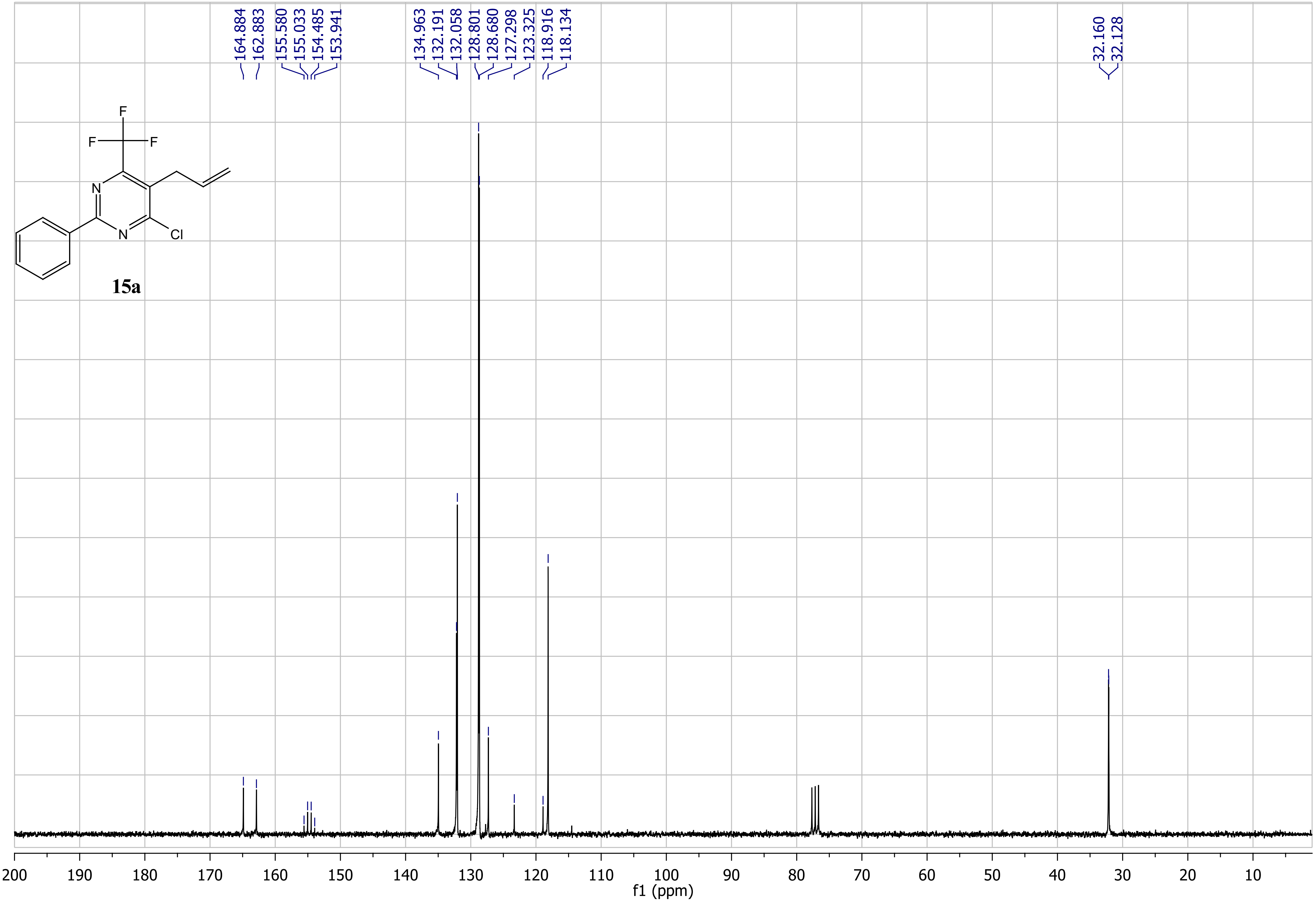
164.884
162.883
155.580
155.033
154.485
153.941
134.963
132.191
132.058
128.801
128.680
127.298
123.325
118.916
118.134
32.160
32.128
F
F
F
N
N
Cl
15a
200
190
180
170
160
150
140
130
120
110
100
90
80
70
60
50
40
30
20
10
f1 (ppm)

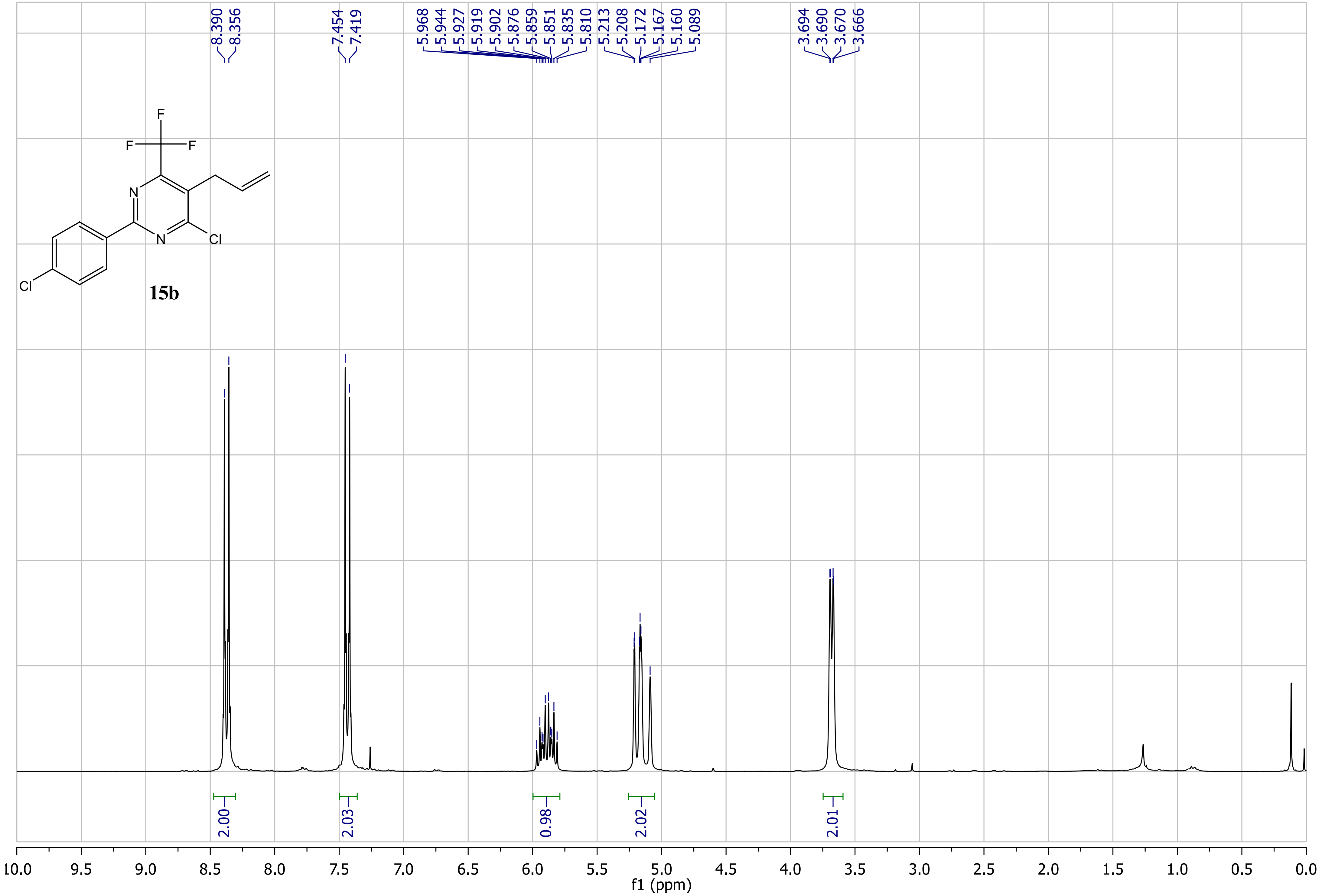
8.390
8.356
7.454
7.419
5.968
5.944
5.927
5.919
5.902
5.876
5.859
5.851
5.835
5.810
5.213
5.208
5.172
5.167
5.160
5.089
3.694
3.690
3.670
3.666
F
F
F
N
N
Cl
Cl
15b
2.00
2.03
0.98
2.02
2.01
10.0
9.5
9.0
8.5
8.0
7.5
7.0
6.5
6.0
5.5
5.0
4.5
4.0
3.5
3.0
2.5
2.0
1.5
1.0
0.5
0.0
f1 (ppm)

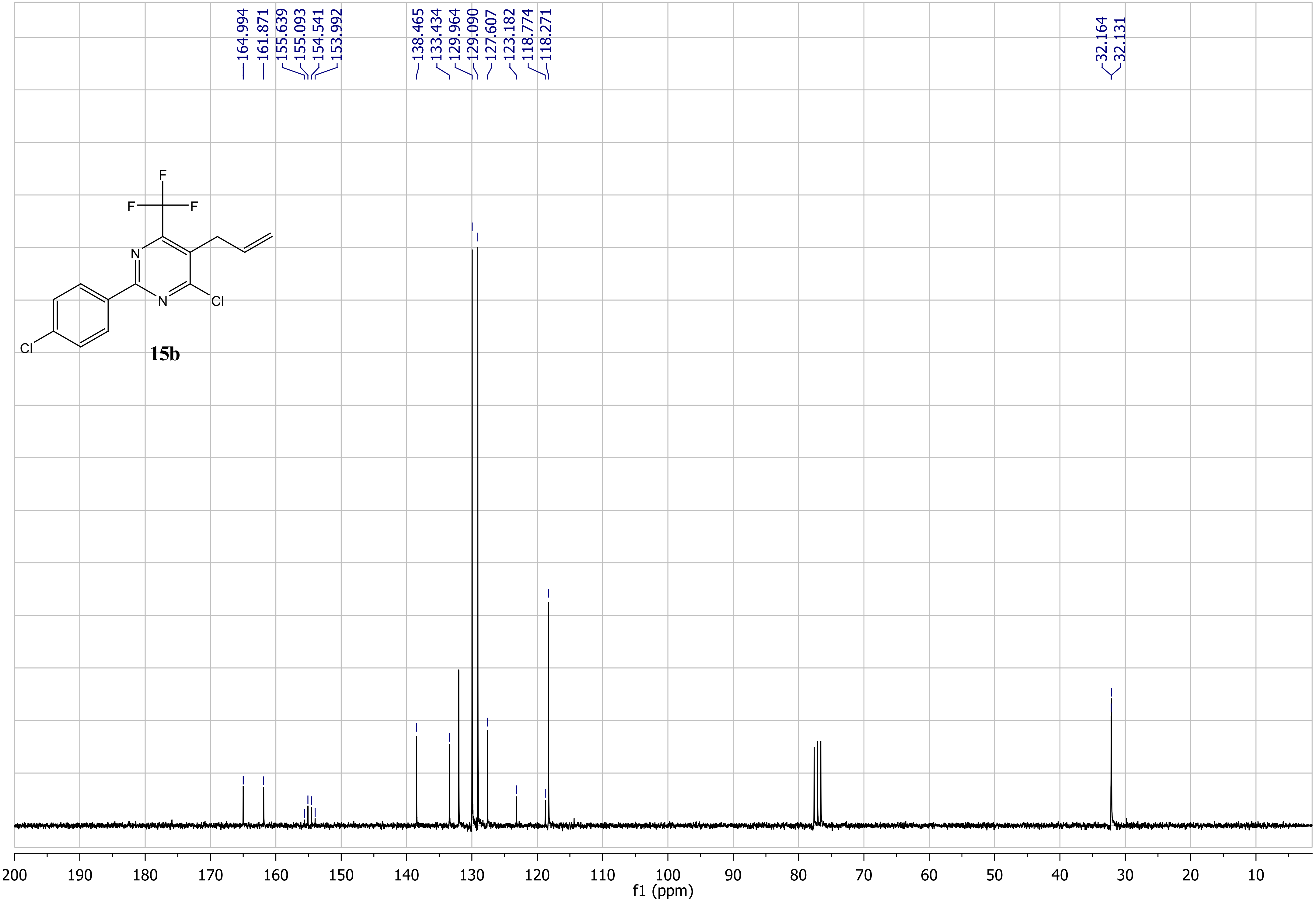
164.994
161.871
155.639
155.093
154.541
153.992
138.465
133.434
129.964
129.090
127.607
123.182
118.774
118.271
32.164
32.131
F
F
F
N
N
Cl
Cl
15b
200 190 180 170 160 150 140 130 120 110 100 90 80 70 60 50 40 30 20 10
f1 (ppm)

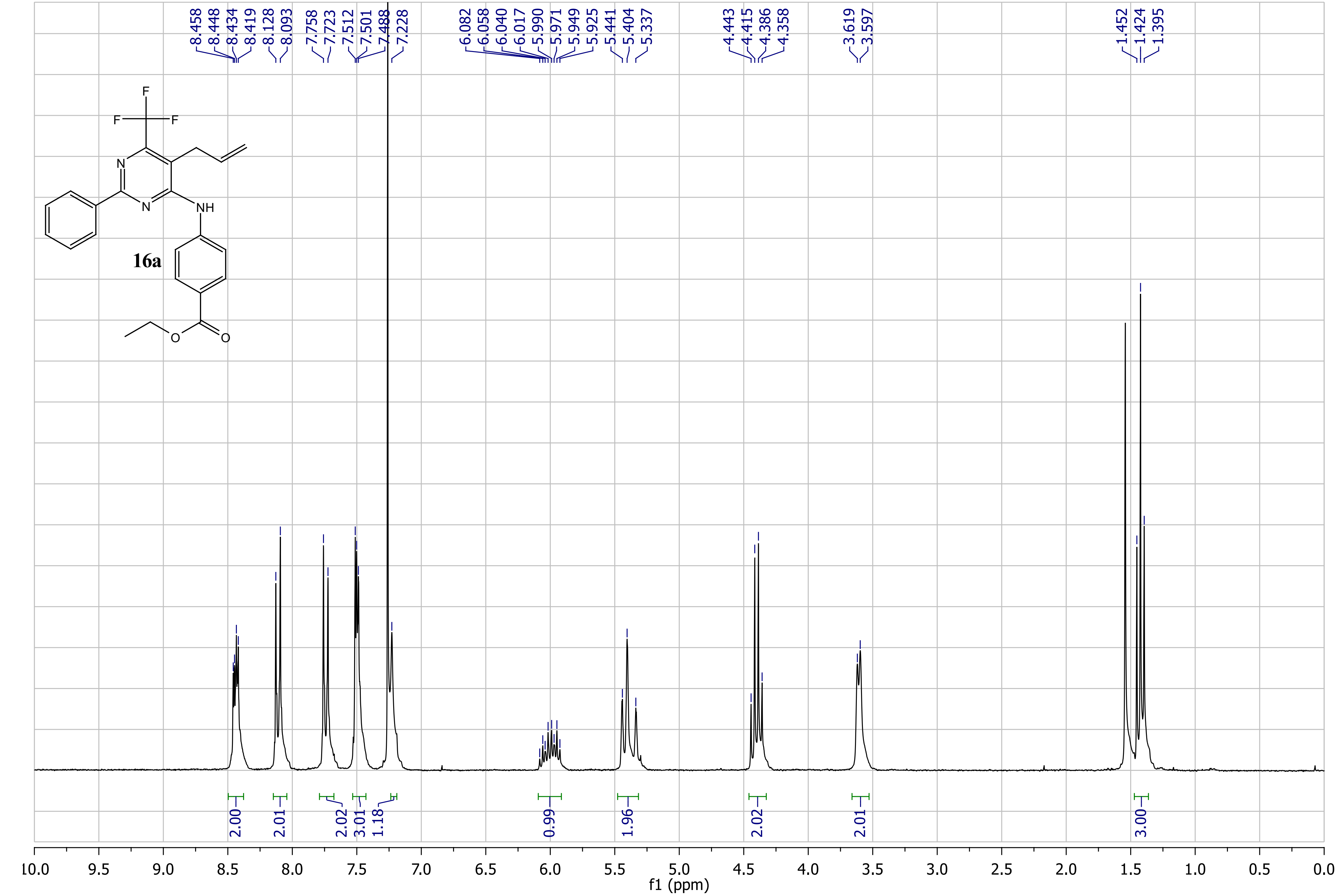
8.458
8.448
8.434
8.419
8.128
8.093
7.758
7.723
7.512
7.501
7.488
7.228
6.082
6.058
6.040
6.017
5.990
5.971
5.949
5.925
5.441
5.404
5.337
4.443
4.415
4.386
4.358
3.619
3.597
1.452
1.424
1.395
16a
2.00
2.01
2.02
3.01
1.18
0.99
1.96
2.02
2.01
3.00
f1 (ppm)

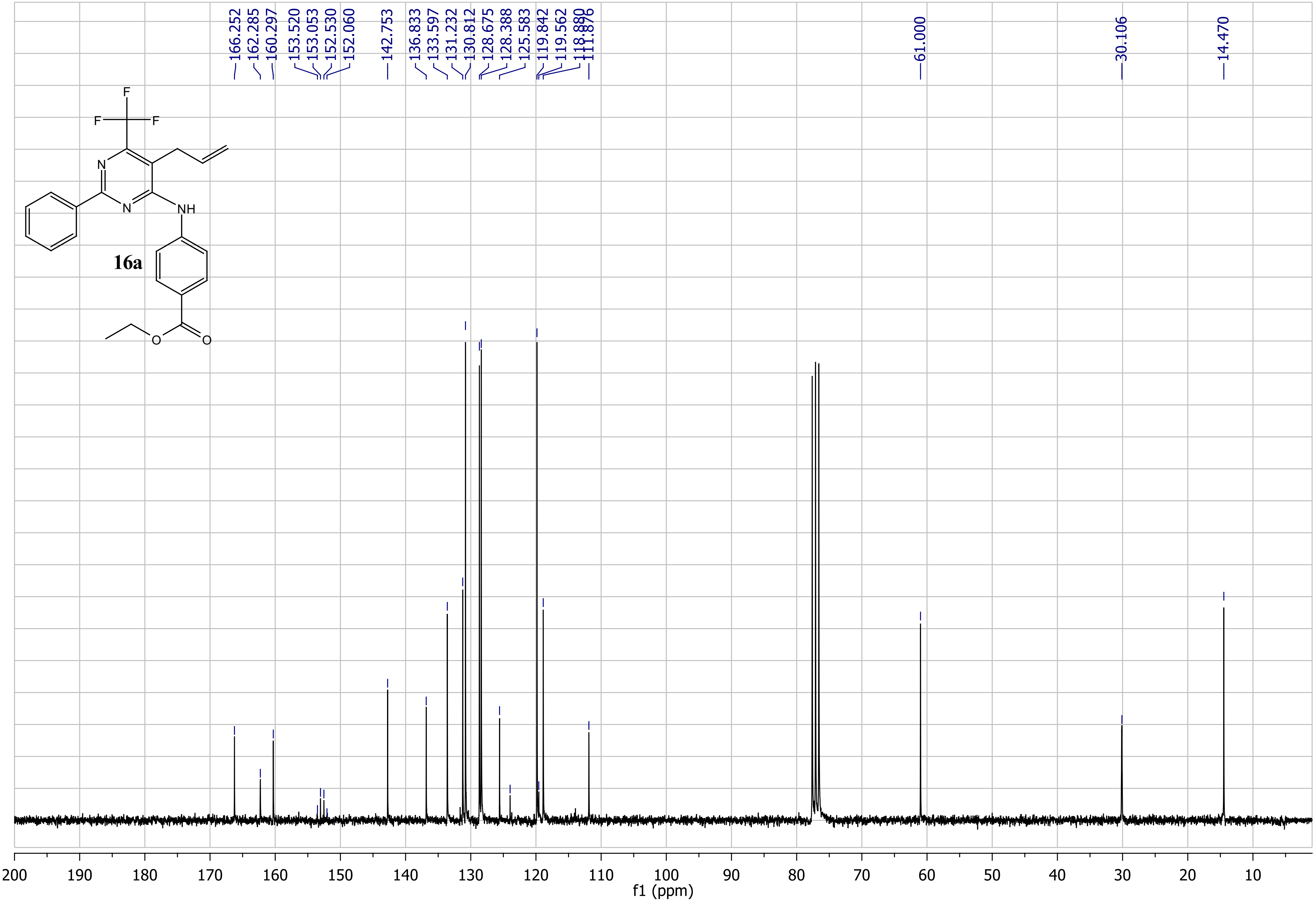
166.252
162.285
160.297
153.520
153.053
152.530
152.060
142.753
136.833
133.597
131.232
130.812
128.675
128.388
125.583
119.842
119.562
118.880
111.876
61.000
30.106
14.470
F
F
F
N
N
NH
O
O
16a
200 190 180 170 160 150 140 130 120 110 100 90 80 70 60 50 40 30 20 10
f1 (ppm)

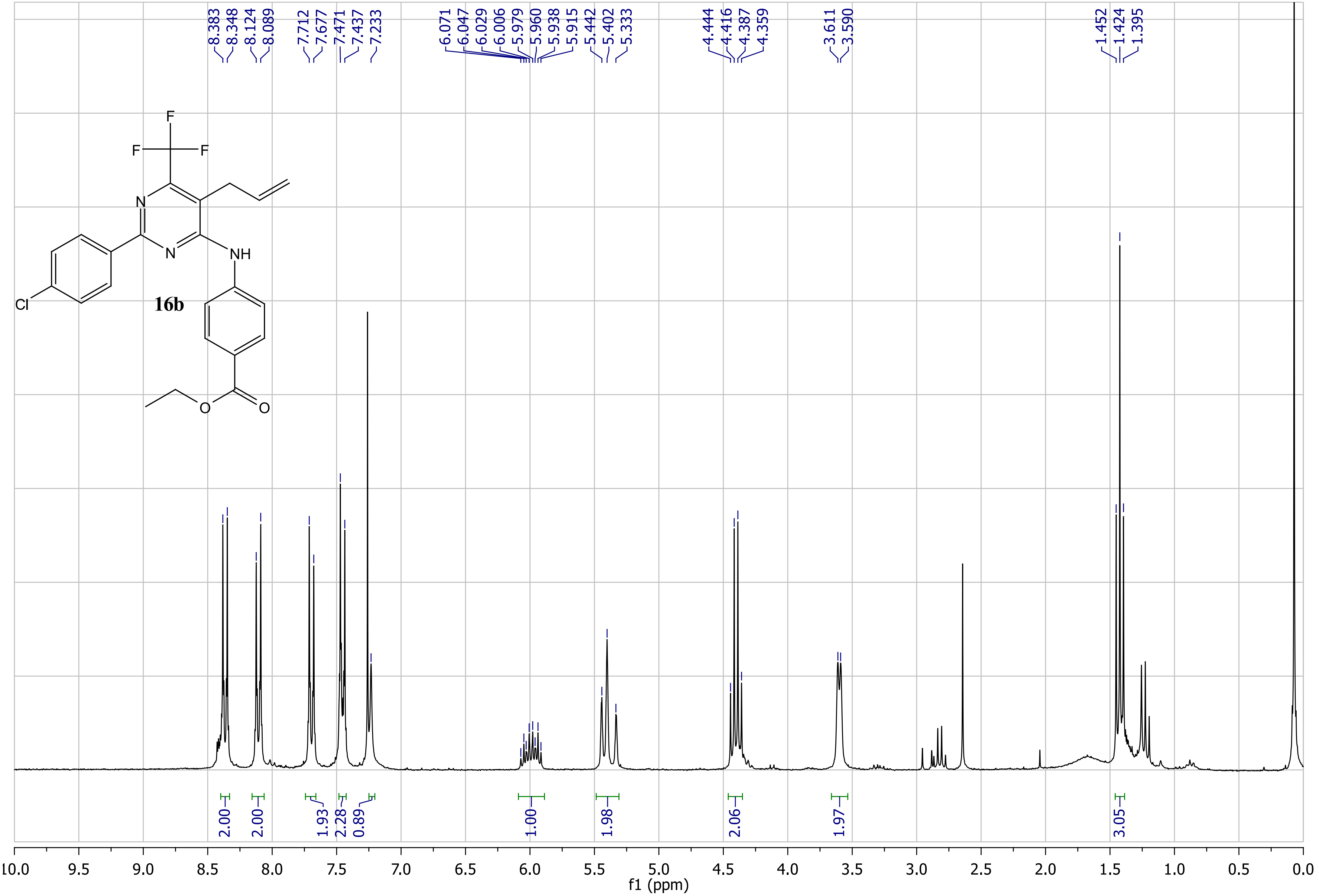
8.383
8.348
8.124
8.089
7.712
7.677
7.471
7.437
7.233
6.071
6.047
6.029
6.006
5.979
5.960
5.938
5.915
5.442
5.402
5.333
4.444
4.416
4.387
4.359
3.611
3.590
1.452
1.424
1.395
16b
2.00
2.00
1.93
2.28
0.89
1.00
1.98
2.06
1.97
3.05
f1 (ppm)

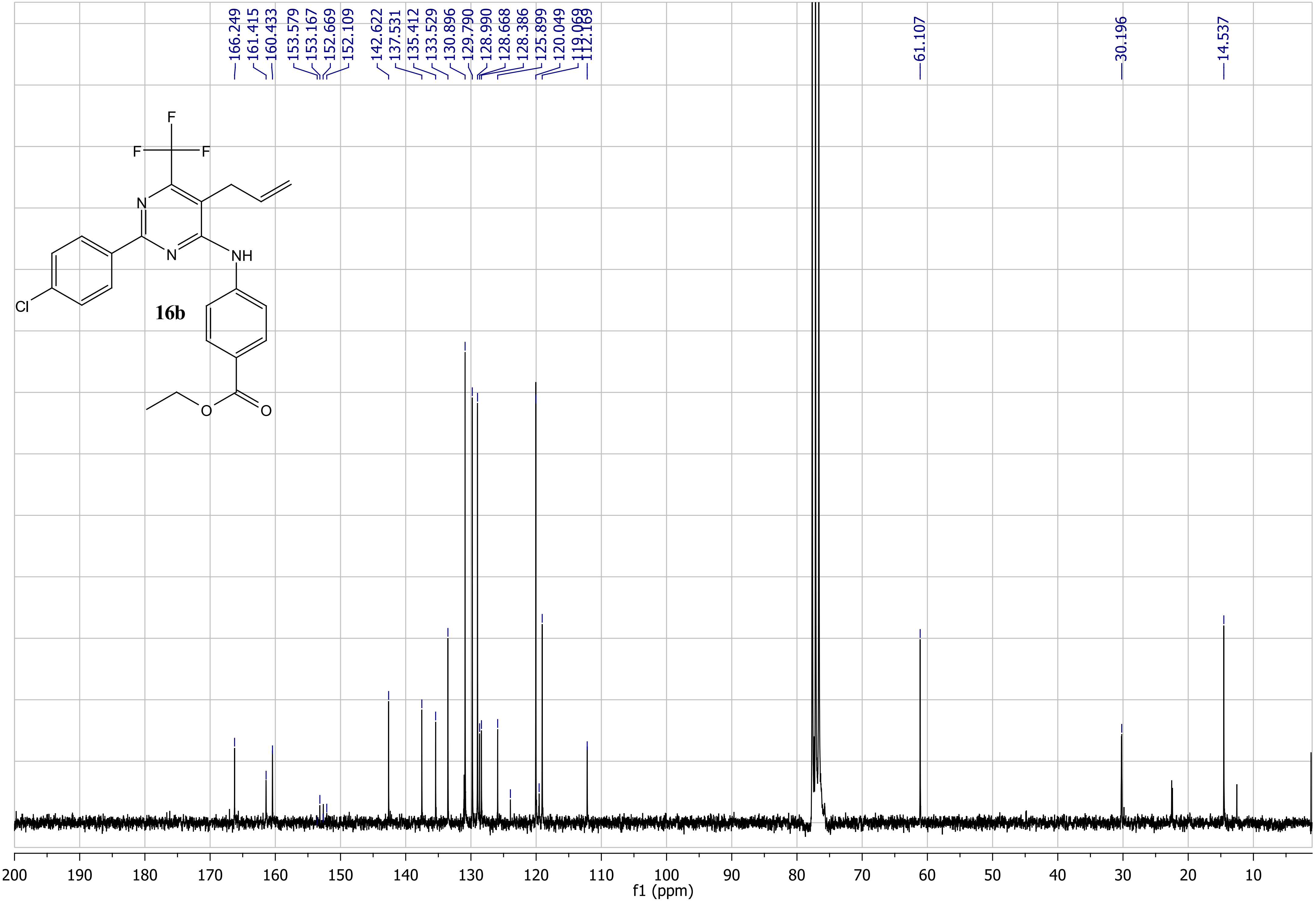
166.249
161.415
160.433
153.579
153.167
152.669
152.109
142.622
137.531
135.412
133.529
130.896
129.790
128.990
128.668
128.386
125.899
120.049
119.069
112.169
61.107
30.196
14.537
F
F
F
N
N
NH
Cl
16b
O
O
200
190
180
170
160
150
140
130
120
110
100
90
80
70
60
50
40
30
20
10
f1 (ppm)

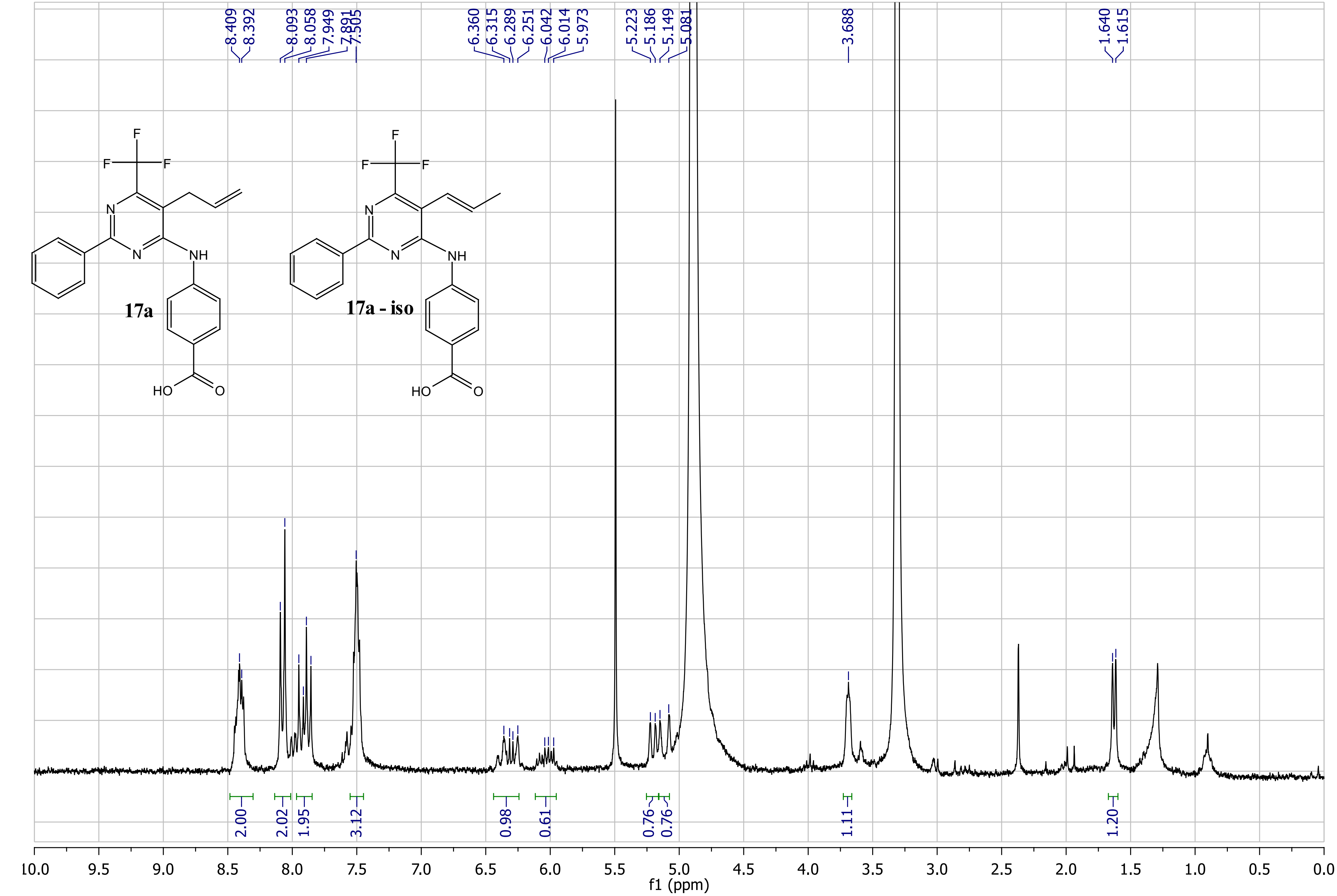
8.409
8.392
8.093
8.058
7.949
7.891
7.505
6.360
6.315
6.289
6.251
6.042
6.014
5.973
5.223
5.186
5.149
5.081
3.688
1.640
1.615
17a
17a - iso
2.00
2.02
1.95
3.12
0.98
0.61
0.76
0.76
1.11
1.20
f1 (ppm)

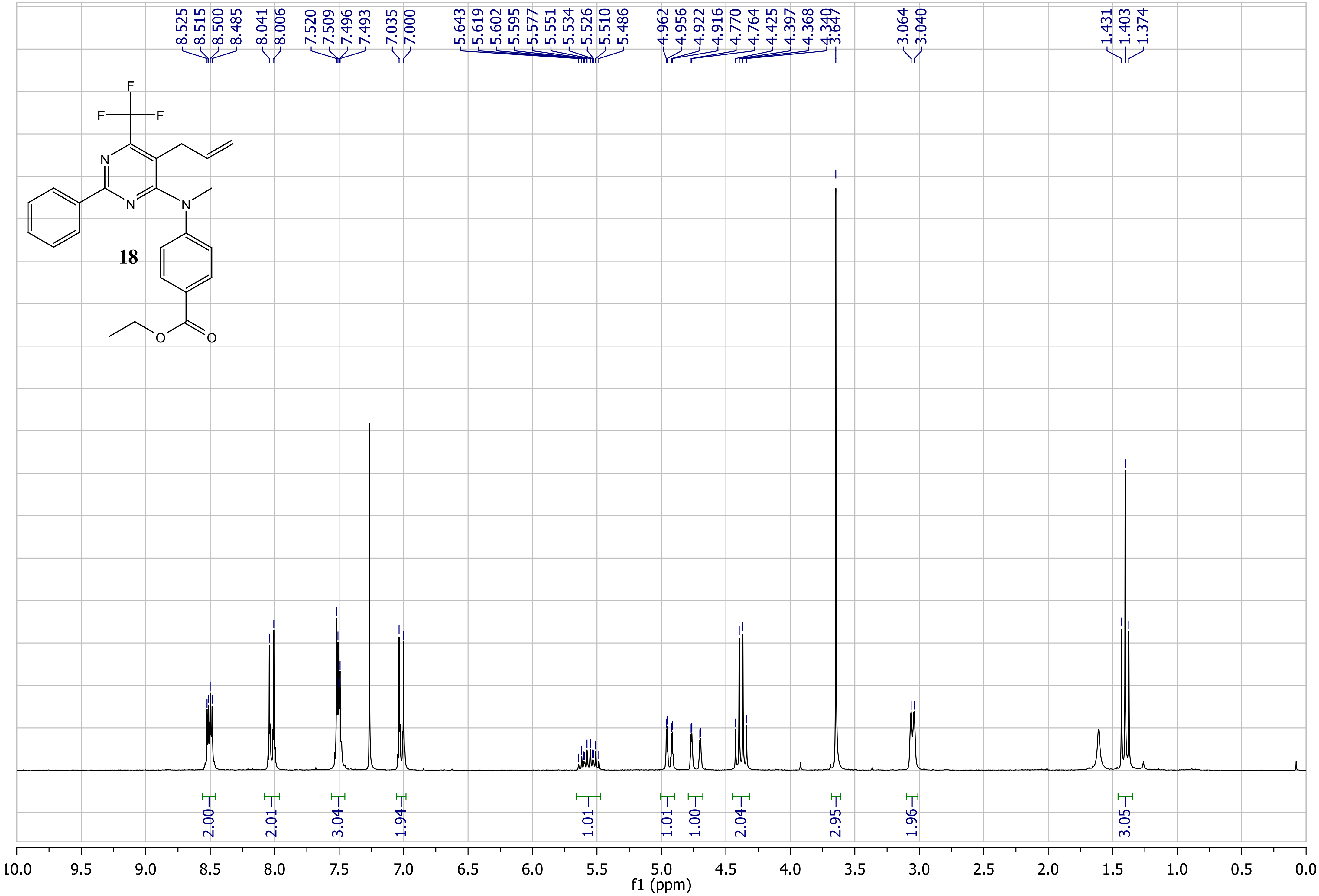
8.525
8.515
8.500
8.485
8.041
8.006
7.520
7.509
7.496
7.493
7.035
7.000
5.643
5.619
5.602
5.595
5.577
5.551
5.534
5.526
5.510
5.486
4.962
4.956
4.922
4.916
4.770
4.764
4.425
4.397
4.368
4.340
3.647
3.064
3.040
1.431
1.403
1.374
F
F
F
N
N
N
O
O
18
2.00
2.01
3.04
1.94
1.01
1.01
1.00
2.04
2.95
1.96
3.05
10.0
9.5
9.0
8.5
8.0
7.5
7.0
6.5
6.0
5.5
5.0
4.5
4.0
3.5
3.0
2.5
2.0
1.5
1.0
0.5
0.0
f1 (ppm)

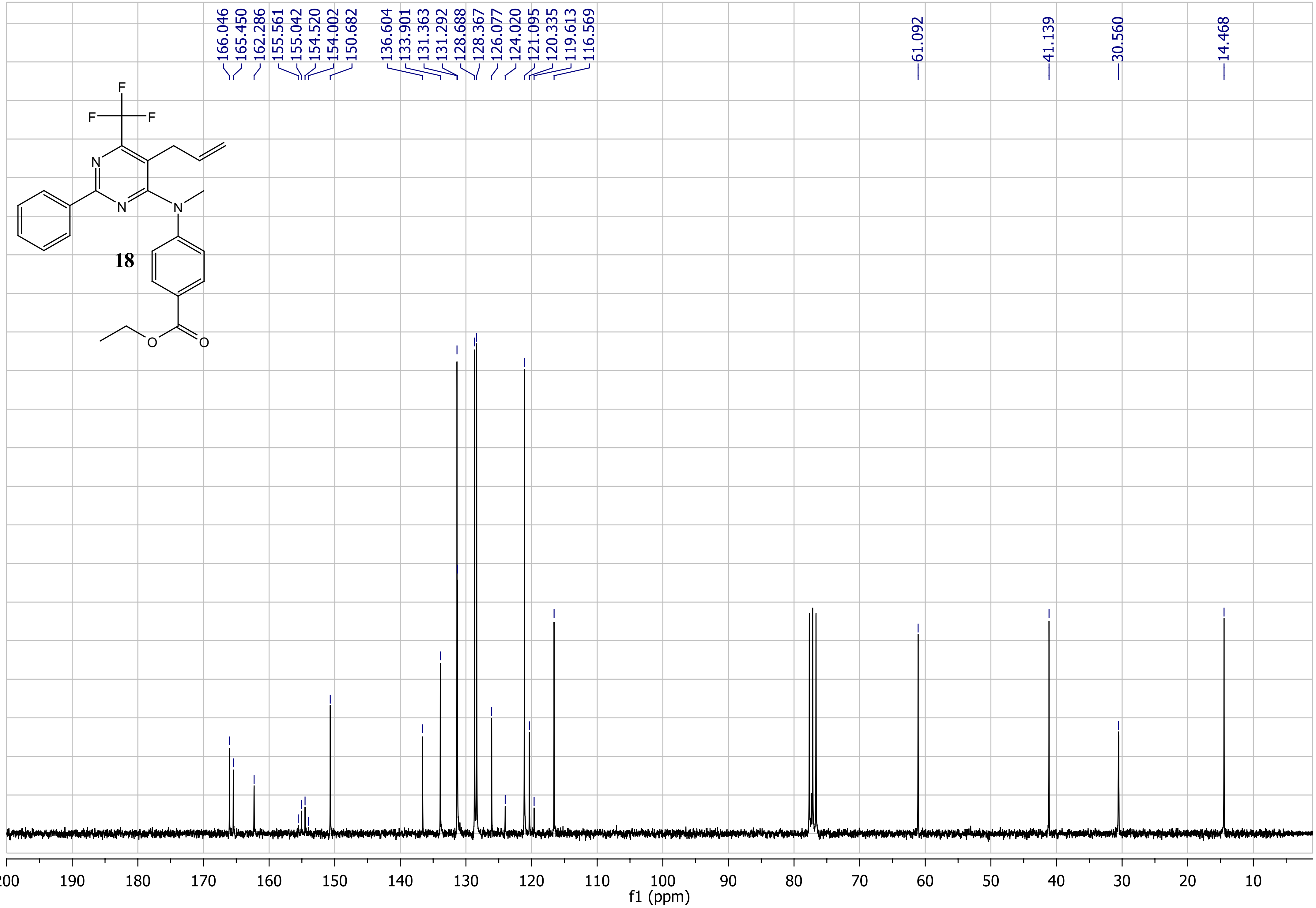
166.046
165.450
162.286
155.561
155.042
154.520
154.002
150.682
136.604
133.901
131.363
131.292
128.688
128.367
126.077
124.020
121.095
120.335
119.613
116.569
61.092
41.139
30.560
14.468
F
F
F
N
N
N
O
O
18
200 190 180 170 160 150 140 130 120 110 100 90 80 70 60 50 40 30 20 10
f1 (ppm)

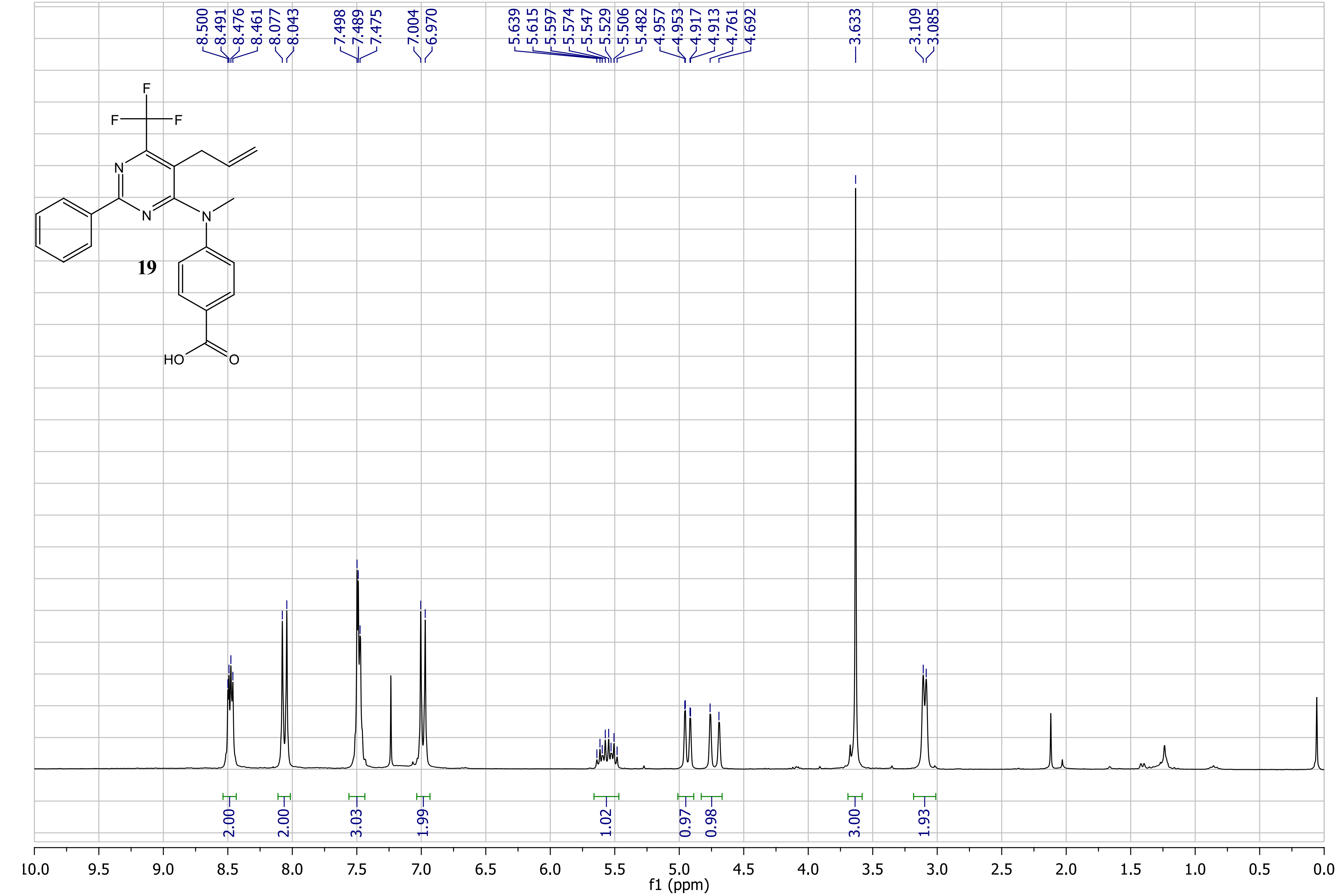

8.500
8.491
8.476
8.461
8.077
8.043
7.498
7.489
7.475
7.004
6.970
5.639
5.615
5.597
5.574
5.547
5.529
5.506
5.482
4.957
4.953
4.917
4.913
4.761
4.692
3.633
3.109
3.085
F
F
F
N
N
N
19
HO
O
2.00
2.00
3.03
1.99
1.02
0.97
0.98
3.00
1.93
10.0
9.5
9.0
8.5
8.0
7.5
7.0
6.5
6.0
5.5
5.0
4.5
4.0
3.5
3.0
2.5
2.0
1.5
1.0
0.5
0.0
f1 (ppm)

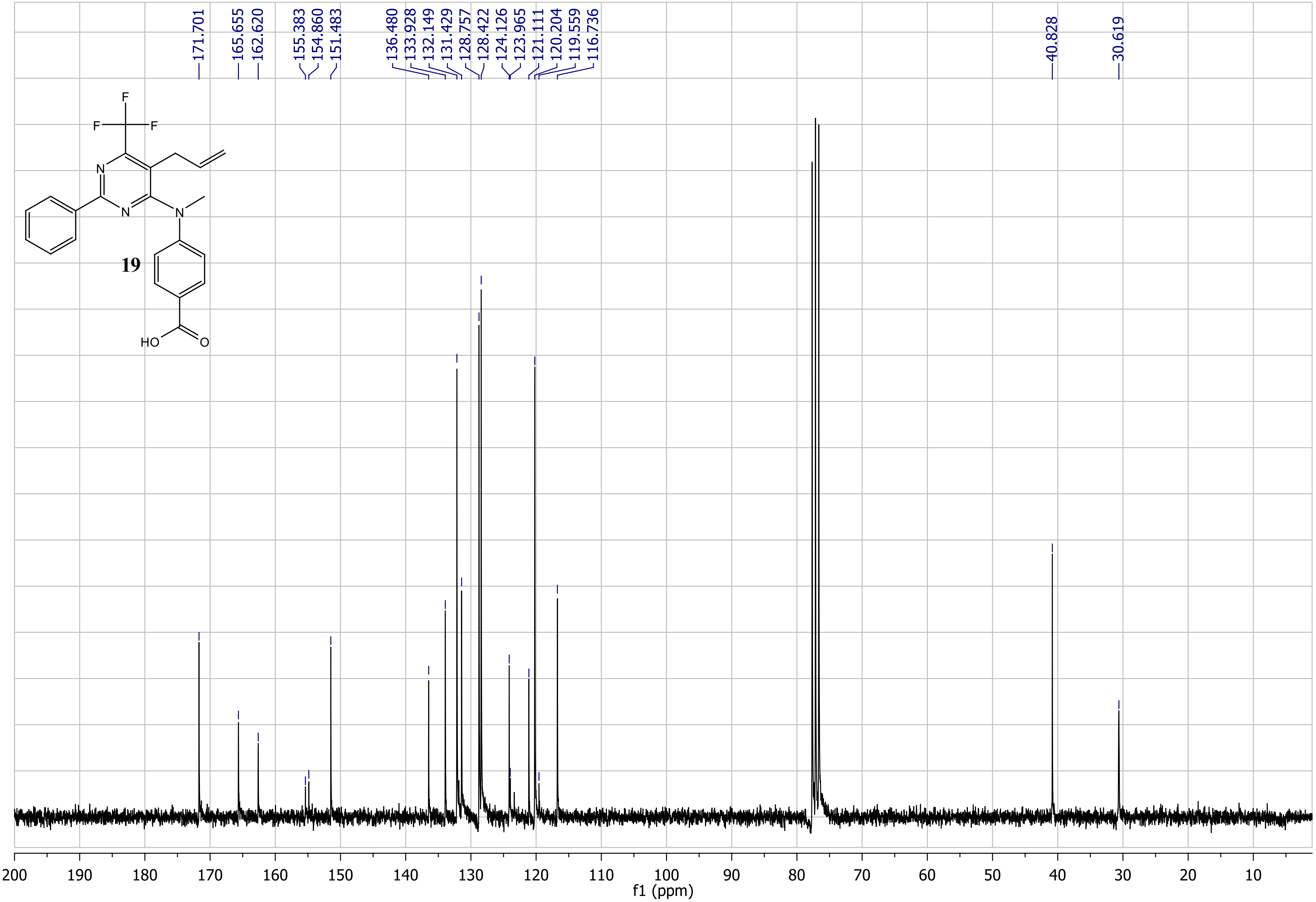
171.701
165.655
162.620
155.383
154.860
151.483
136.480
133.928
132.149
131.429
128.757
128.422
124.126
123.965
121.111
120.204
119.559
116.736
40.828
30.619
19
F
F
F
N
N
N
HO
O
200
190
180
170
160
150
140
130
120
110
100
90
80
70
60
50
40
30
20
10
f1 (ppm)
